# Stereoselective Prenylation of Aryl- and Heteroaryl Halides: γ‑Selective Suzuki–Miyaura Coupling as a Tool for Asymmetric Csp^2^–Csp^3^ Cross-Coupling

**DOI:** 10.1021/acs.joc.6c00410

**Published:** 2026-05-12

**Authors:** Cornelius Pawlowsky, Tim Leipertz, Birgit Henßen, Mona Haase, Jörg Pietruszka

**Affiliations:** † Institute for Bioorganic Chemistry & Bioeconomy Science Center (BioSC), 9170 Heinrich Heine University Düsseldorf in Forschungszentrum Jülich, 52426 Jülich, Germany; ‡ Institute of Bio- and Geosciences (IBG-1: Bioorganic Chemistry) & Bioeconomy Science Center (BioSC), Forschungszentrum Jülich, 52426 Jülich, Germany

## Abstract

The stereoselective preparation of all carbon quaternary stereogenic centers attached to an aromatic core structure is a long-standing challenge for synthetic chemists, due to the sterically encumbered nature of the central carbon atom. Natural products, like teleocidin A or sporochnol A, carrying a branched geranyl chain feature this special and challenging structural motif. Herein, we present a catalytic system that promotes the asymmetric γ-selective Suzuki–Miyaura cross-coupling of aryl halides and allyl boronic acid esters, to form the desired stereogenic center in one step. The γ-selective coupling approach overcomes the need for a preformed stereogenic center, gives selective access to both stereoisomers, and starts with readily available starting materials. In this study we investigate the scope of the γ-selective cross-coupling reaction and showcase its applicability for late-stage derivatization and natural product synthesis, to provide a further asset in the toolbox of asymmetric synthesis.

## Graphical abstract

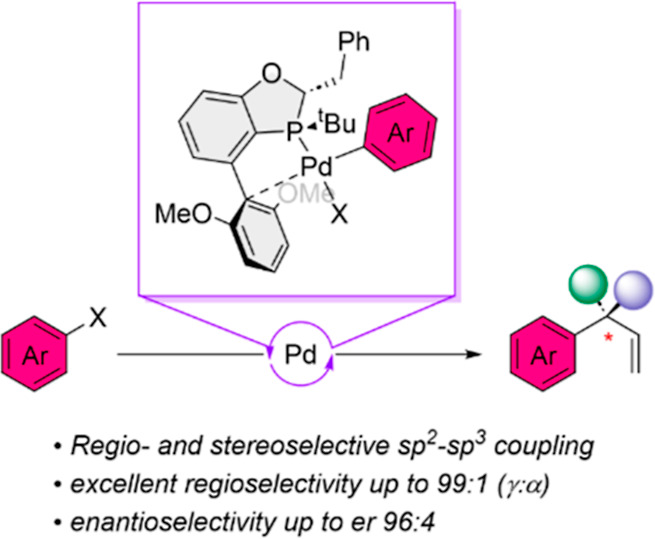

## Introduction

Terpenoids are the largest family of natural products as they can be found in all living organisms, thereby carrying an enormous range of structural diversity interesting for natural product related research.^1^ Prevalent in the primary and secondary metabolism, terpenoids assume all sorts of biological functions, ranging from signal transduction to defense mechanisms against competitors, pathogens, and predators.^2–4^ Drawing inspiration from this plethora of biological activity and structural diversity, medicinal research continues to use terpenoids as lead structures for new active agents, exemplified by the success of taxol and artemisinin.^5–7^

The structural diversity of terpenoids is based upon a number of allylic diphosphates that biosynthetically engage as substrates either in a series of regio- and stereoselective cyclization reactions catalyzed by terpene synthases or intermolecular transfer via prenyltransferases.^8^ Natural products that arise from the latter pathway often carry the known prenyl group either in a linear form, represented by nocardiozine B from *Nocardiopsis* sp. (CMB-M0232) or a branched manner, like the secondary metabolite lansai B from *Streptomyces* sp. (SUC1).^9,10^ A structurally interesting motif arises from the combination of higher order allylic diphosphates and the branched prenyl chain, as it creates an all carbon quaternary stereogenic center, which is traditionally a challenging motif for chemists to establish. Representative natural products inheriting the branched geranyl motif are teleocidin A-1 (**1**) from *Streptomyces mecliocidius*, (+)-(*S*)-sporochnol A (**2**) from *Sporochnus bolleanus*, and (+)-(*R*)-bakuchiol (**3**) from *Psoralea corylifolia* L. (Figure 1).^11–13^

**Figure 1:**
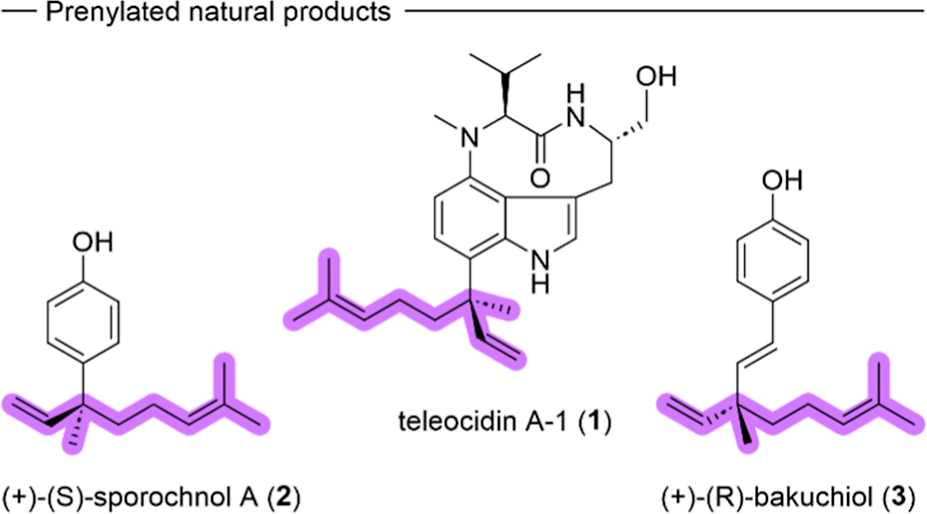
Natural products teleocidin A-1 (**1**), (+)-(*S*)-sporochnol A (**2**), and (+)-(*R*)-bakuchiol (**3**) featuring the branched geranyl side chain.

All three mentioned natural products are potent bioactive molecules, whose activity was at least partially attributed to their chiral terpenoid side chain.^14,15^ While sporochnol A (**2**) exhibits significant feeding deterrence toward herbivorous fishes, bakuchiol (**3**) possesses a wider range of biological activities including antifungal, antibacterial, anti-inflammatory, and anticancer properties.^16,17^ Teleocidin A-1 (**1**) is mostly known for its high inflammatory and toxic effects as well as its ability of activating protein kinase C isozymes, making it a potent tumor promoter.^18,19^

Despite being prevalent in many natural product scaffolds, the selective asymmetric synthesis of all carbon quaternary stereogenic centers still remains challenging. Synthetic asymmetric methods to establish the stereogenic center via direct Csp^2^ to Csp^3^ cross-coupling from non stereogenic starting materials remain underdeveloped to this day. A biomimetic and long-established approach to synthesize the branched geranyl chain is based upon allylic substitution type chemistry utilizing allylic nucleofuges in combination with metal catalysis, instead of the natural allylic phosphates and enzyme catalysis. The Stoltz group established a method delivering the desired branched geranyl chain with high levels of enantio- and regioselectivity based on the combination of iridium and phosphoramidite ligands (Scheme 1A).^20^ Unfortunately, most stereoselective Tsuji–Trost-type approaches rely on the use of softer nucleophiles like malonates or otherwise inherently nucleophilic carbon atoms.^21^ The implementation of harder nucleophiles to directly attach the branched geranyl chain toward arenes has been pioneered by the Bäckvall group using organo-magnesium nucleophiles and copper catalysis.^22^ Since then, similar techniques have been brought forward utilizing indium, boron, or aluminum nucleophiles.^23–25^ Nonetheless, these methods suffer from limited functional group tolerance and the sensitive nature of the metal arene. Hoveyda and co-workers were the first to establish the desired structural motif directly in an enantioselective fashion (Scheme 1B).^25,26^

**Scheme 1:**
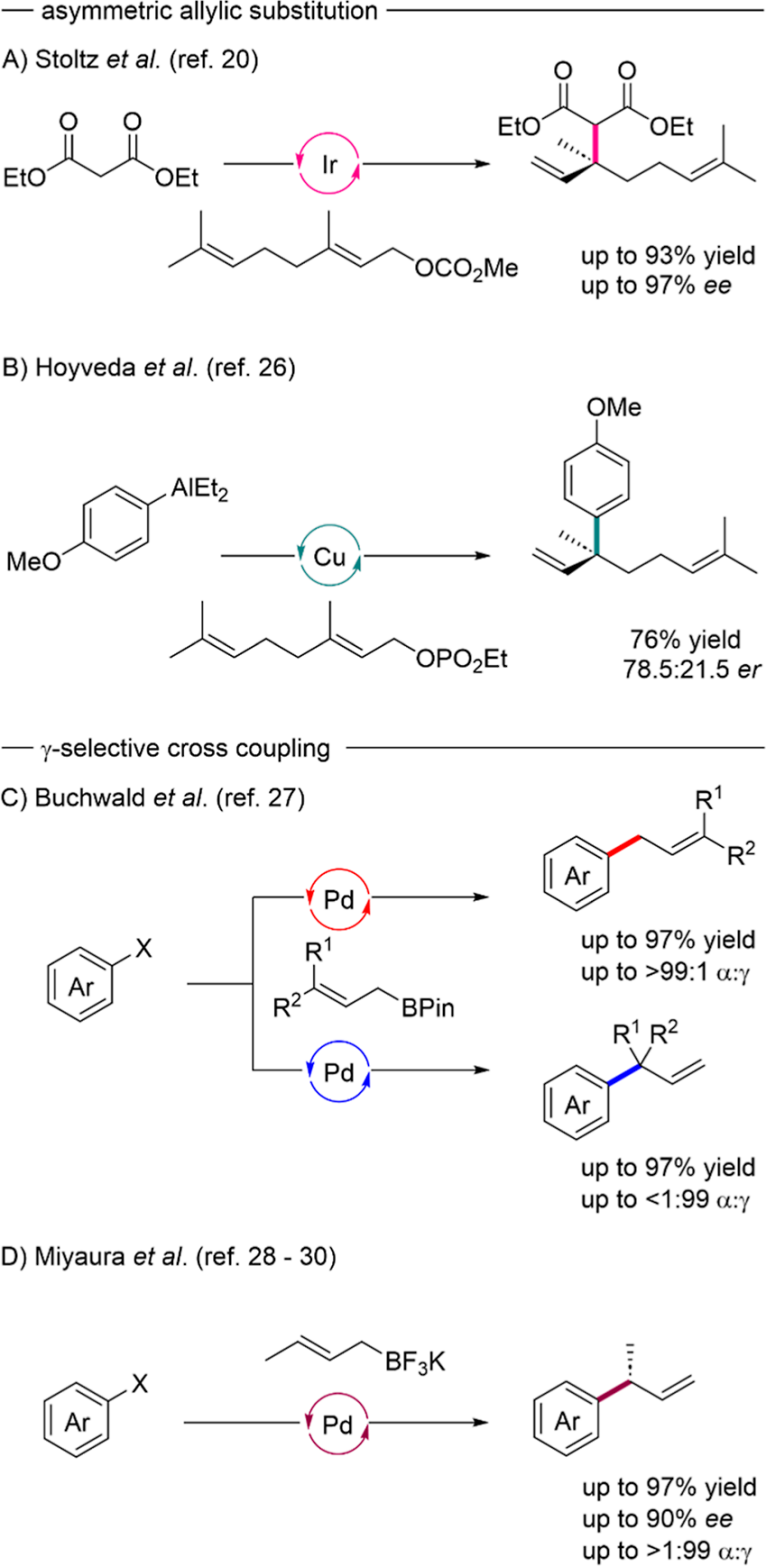
Established Catalytic Approaches toward the Branched Prenyl Chain Motif

The application of allylic nucleophiles in enantioselective synthesis, for example, in the allylation of carbonyl compounds, has been established for just as long as allylic substitution chemistry. When used in a γ-selective cross-coupling type manner, the combination of allylic nucleophiles and electrophilic arenes can provide the same desired branched prenyl motif. Recently, Buchwald and co-workers presented two palladium based catalytic systems that, by choice of the employed ligand, can selectively form either the linear or branched prenyl motif using allylic boronic acid esters in a α/γ-selective Suzuki–Miyaura reaction (Scheme 1C).^27^ The only asymmetric version of the γ-selective Suzuki reaction in literature has been brought forward by the Miyaura group, who used the chiral JosiPhos ligand system to induce stereo- and regioselectivity in the cross-coupling of potassium crotyl trifluoroborates and aryl halides (Scheme 1D).^28–30^

In our efforts to access the branched geranyl motif in a stereoselective fashion, we envisioned that γ-selective Suzuki–Miyaura coupling, based on a suitable catalytic system, could eliminate the drawbacks that allylic substitution type approaches inherit and directly build the desired motif in an asymmetric manner. Herein, we present a catalytic system that allows the direct Csp^2^ to Csp^3^ cross-coupling of allylic boronates and heteroaryl halides and thereby establishes the desired all carbon quaternary stereogenic center in one step via γ-selective coupling. The combination of readily accessible allylic boronates and their robustness compared to other organometal species in conjunction with the mild reaction conditions of our catalytic system should make this approach an interesting new asset in the toolbox of asymmetric synthesis of all carbon quaternary stereogenic centers.

## Results and Discussion

### Reaction Optimization

On the basis of the previous works by the Buchwald and Miyaura groups, we started our research with the hypothesis that a chiral palladium-based catalytic system would be able to regio- and stereoselectively catalyze the coupling between heteroaryl halides and geranyl boronic acid esters. To achieve the desired stereoselective outcome of the coupling reaction, two factors were deemed critical in our starting hypothesis: the first one being control of the configuration of the double bond of the allylic boronate and the second one being directing the faciality of the coordination of the oxidative-addition Pd^II^ species onto the allylic boronate prior to transmetalation, thus achieving either selective coordination of the *re*- or *si*-site of the prochiral allylic boronate.

Following these assumptions, we began investigating the synthesis of a panel of stereodefined allylic boronates from the inexpensive commercially available allylic alcohols geraniol (**A1**) and nerol (**A2**). A variety of different Pd-based catalytic systems were applied to directly convert the allylic alcohol toward the allyl boronic acid ester. While [Pd­(CH_3_CN)_4_]­(BF_4_)_2_ gave complete conversion of the allylic alcohol and the corresponding bisboronate (**BB1** or **BB2**) toward the desired allyl boronic acid ester within 2 h at room temperature, an inseparable mixture of *E* and *Z* allylic boronate was obtained, independent of the identity of the starting allylic alcohol. Application of di-μ-chlorobis­{2-[(dimethylamino)­methyl]­phenyl-C,N} dipalladium­(II) as a catalyst at 60 °C overnight selectively delivers the *E*-allylic boronate independent of the nature of the used alcohol.^31^ H_2_PdCl_4_, which is generated from inexpensive and stable PdCl_2_ in an aqueous solution of hydrogen chloride, emerged as our catalyst of choice and is able to convert the desired allylic alcohol and toward the allylic boronate without loss of stereoinformation or racemization under mild conditions.^32^ Under these conditions the desired allyl boronic acid ester can be directly obtained from the corresponding bis boronate (**BB1** or **BB2**) or via synthesis of the allyl boronic acid and one-pot esterification with the corresponding diol. In that manner we established a panel of four different allyl boronic acid esters (**B1**–**B4**) with varying configurations of the allylic double bond, as well as different steric bulk of the ester functionality (Scheme 2). An advantageous aspect of these allyl boronic acid esters, in comparison to other organometallic species used for example in allylic substitution chemistry, is that these compounds can be handled under aerobic conditions and seemed to be stable indefinitely, when stored dry under argon at −20 °C, during our research.

**Scheme 2:**
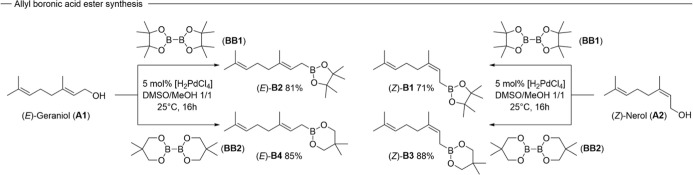
Synthesis of Various Allyl Boronic Acid Esters via Palladium Catalyzed Direct Boronation

With these four different allyl boronic acid esters in hand, we tested the assumption that a chiral catalyst system would be able to preferably coordinate one face of the allylic boronate and therefore lead to a stereoselective reaction. In their work concerning the α/γ-selective Suzuki–Miyaura reaction, Buchwald and co-workers argue that the choice of ligand would influence the reaction mechanism and the rate of the single mechanistic steps, consequently leading to different regioselective outcomes.^27^ They found that SPhos, RuPhos, as well as ligand **L1**, which is structurally derived from SPhos, give almost exclusively the γ-coupling product.^27^ Inspired by this work, we hypothesized that a structurally related but chiral version of these employed ligands would not only be able deliver the desired regioselectivity but would also control the faciality of the coordination of the catalyst species toward the allyl boronic acid ester. To examine our hypothesis and due to our interest in prenylated natural compounds like lansai B, we started investigating the branched geranylation of 5-bromoindole (**4**).^9^ We based our starting conditions on Buchwald’s work using a 1:1 mixture of tetrahydrofuran (THF) and aqueous potassium phosphate (2.5 M), 1.2 equiv of allylic boronate, in combination with 2.5 mol % of [Pd­(allyl)_2_Cl_2_] as palladium source and 10 mol % of the corresponding ligand.^27^ Furthermore, initial testing only provided positive results for allyl boronic acid esters as nucleophiles, whereas the corresponding allylic potassium trifluoroborates did not yield the desired result (not shown). After screening ligands based on structural similarity to **L1** (Table 1, entry 1, er 50:50), we found that *P*-chiral oxaphosphol ligands (**L4**–**L6**) are able to deliver the desired regio- and stereoinduction (entry 4–6), with regioselectivities up to 10:90 (α/γ) toward the branched regioisomer and enantiomeric ratios up to 93:7. Moreover, a sterically bulky substituent in position 2 of the oxaphosphol ring, pointing in the opposite direction of the phosphorus’ *tert*-butyl group, proved critical for effective stereoselection. The axially chiral ligand **L2** and *P*-chiral BIDIME (**L3**) failed to catalyze the reaction efficiently (entries 2/3). Missing stereogenic information concerning position 2 of the oxaphosphol ring (entry 7, **L7**, 68:32 er) led to decreased stereoinduction, whereas a substituent carrying another coordination site diminished the yield (10%) drastically (entry 8).

**Table 1:**
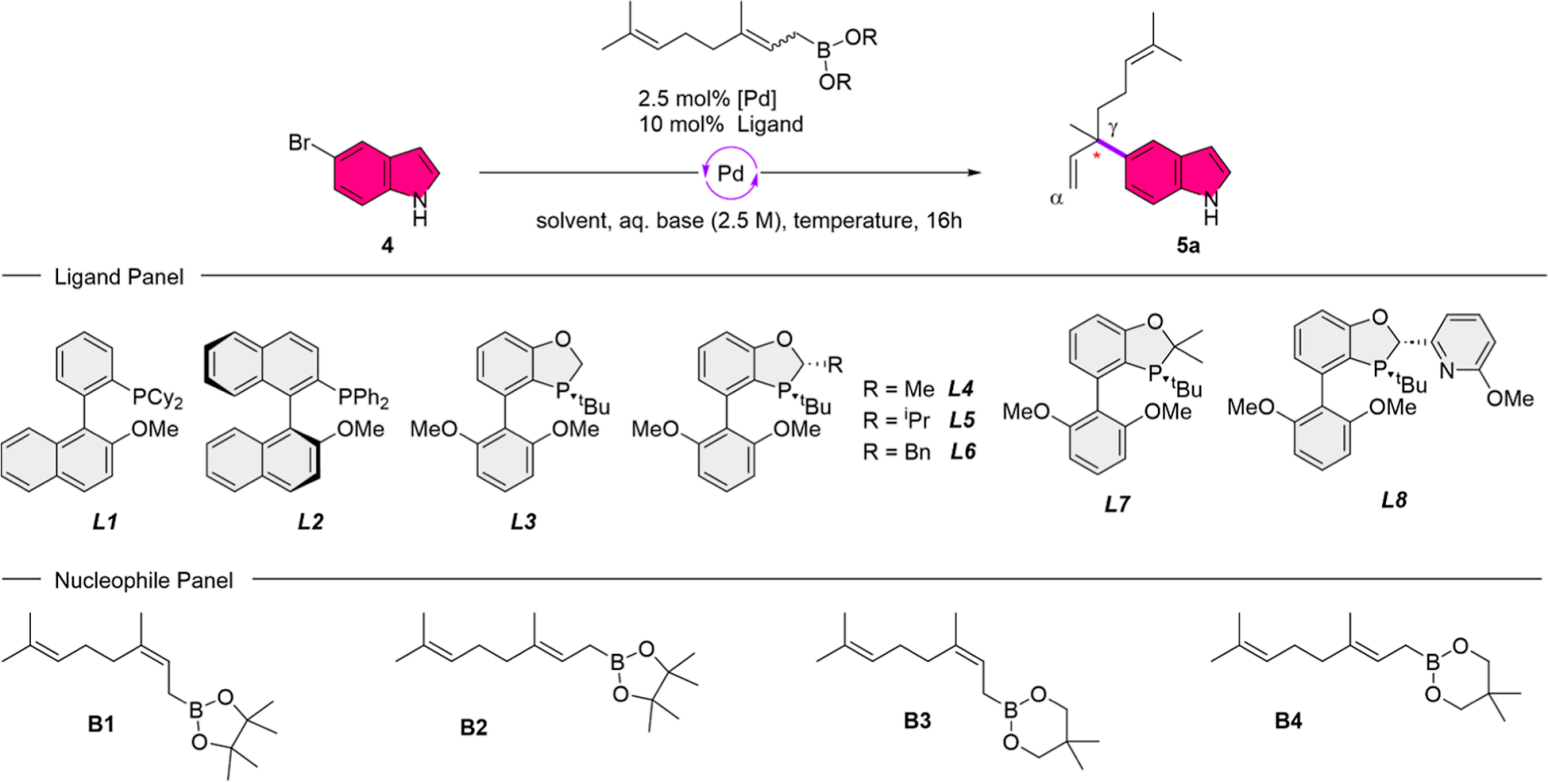
Optimization of the Asymmetric γ-Selective Suzuki–Miyaura Coupling^a^

entry	ligand	nucleophile	temperature (°C)	yield (%)^b^	α/γ^c^	er^d^
1	**L1**	**B1**	40	84	8:92	50:50
2	**L2**	**B1**	40	traces	93:7	-
3	**L3**	**B1**	40	traces	-	-
4	**L4**	**B1**	40	23	42:58	87:13
5	**L5**	**B1**	40	56	14:86	90:10
6	**L6**	**B1**	40	51	10:90	93:7
7	**L7**	**B1**	40	64	18:82	68:32
8	**L8**	**B1**	40	10	78:22	77:23
9	**L5**	**B2**	40	79	13:87	11:89
10	**L5**	**B3**	rt	64	13:87	92:8
11	**L6**	**B3**	rt	85	2:98	95:5
12	**L6**	**B1**	rt	7	88:12	55:45
13	**L6**	**B2**	rt	35	38:62	22:78
14	**L6**	**B4**	rt	78	1:99	7:93

a Standard reaction conditions: 5-Bromoindole (0.2 mmol), allylic boronate (0.24 mmol), [Pd­(allyl)­Cl]_2_ (0.005 mmol), ligand (0.020 mmol), THF (0.4 mL), aq. K_3_PO_4_ (0.4 mL (2.5 M)), 40 °C, 16 h.

b Determined via ^1^H qNMR using 1,3,5-trimethoxybenzene as an internal standard.

c Determined via ^1^H NMR.

d Determined by high-performance liquid chromatography (HPLC) analysis via comparison with an authentic racemic sample.

We further examined the effect of the constitution of the allyl boronic acid ester on the selectivity of the reaction. Interestingly, changing the configuration of the allylic double-bond results in similar levels of stereoselectivity but gives the opposite enantiomer (entry 5/9, 90:10/11:89 er). Thus, both stereoisomers of the desired product are accessible through the same catalytic system. Altering the nature of the boronic acid ester from the pinacol ester (**B1**) toward the less sterically bulky neopentylglycol boronate (**B3**), in combination with a lowered temperature, gave enhanced regioselectivity and very high level of enantioselection (entry 10/11) for ligands **L5** and **L6**, with superior results for **L6** (2:98 α/γ, 95:5 er). Application of the pinacol derived boronic acid ester at room temperature led to unselective reactions (entry 12/13), whereas the reaction via the geranyl neopentyl derived boronate (**B4**, entry 14) gave similar results as **B3**, highlighting the accessibility of both enantiomers. Further optimization concerning the solvent, base, and palladium source, as well as different metal to ligand ratios gave no significant improvement in terms of regio- or stereoselectivity (Supporting Information Section 2).

Based on the proposed mechanistic pathway for the γ-selective cross-coupling reaction by the Buchwald group and our finding, that the two different possible configurations of the allylic boronate lead toward different preferred stereoisomers, we propose an improved mechanistic view for the asymmetric γ-selective cross-coupling reaction (Scheme 3).^27^ In analogy to the previously proposed mechanism, transmetalation of the allylic boronate prevails via the S_E_2́ pathway.^27^ For higher-order prenyl boronates this pathway leads through two different transmetalation transition states **TS**_***R***_ and **TS**_***S***_ toward differently configurated intermediates **I**_***R***_ and **I**_***S***_, which after reductive elimination furnish the two differently configurated stereoisomers (*R*) and (*S*). Evidently, altering the allylic double bond of the starting material between *E* and *Z* leads to different stereoisomers, which indicates that the rate of π–σ–π interconversion *k*_4_/*k*_–4_ from **I**_***R***_ to **I**_***S***_ via the linear transition state **TS**_***L***_ is slower compared to the rate of reductive elimination *k*_3_ and no equilibrium between **I**_***R***_ to **I**_***S***_ reached. Furthermore, our finding suggests that contrary to our starting hypothesis the stereochemical outcome is not merely determined by first coordination of the metal catalyst toward one site of the allylic double bond or equilibration between the sites, but by the identity of the cyclic transmetalation transition state and the rate *k*_2_ of the following transmetalation.

**Scheme 3:**
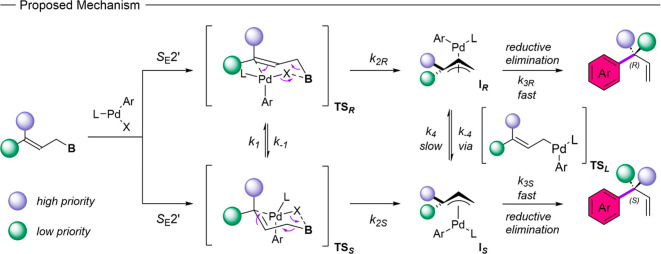
Proposed Mechanism for the Asymmetric γ-Selective Cross-Coupling

### Reaction Scope

To evaluate the generality and synthetic utility of the now established catalytic system, we investigated the scope of the asymmetric γ-selective cross-coupling reaction (Figure 2). We focused our investigation on aryl- and heteroaryl halides bearing different electronic and sterical prerequisites to identify their influence on this type of reaction. Pleasingly, we found that the method generally translated to a wider set of substrates. In nearly every case, with the exception for the pyridine-based heterocycle **5n**, our set of reaction conditions delivered excellent regioselectivity for the branched reaction product. Electronically neutral aryl halides (**5b**, **5f**, **5j**) performed well under the reaction conditions (65–87%), albeit with a lower enantioselection compared to our optimized conditions (∼80:20 er). Interestingly, highly electron-withdrawing substituents like nitro (**5e**) and trifluoromethyl (**5c**) on the aromatic structure had a detrimental effect on stereoselectivity that led up to an almost racemic product (**5e** 54:46 er). To our delight, the electron-donating substituents methoxy (**5d**) and methyl (**5g**) seem to have a positive effect concerning the outcome of our reaction in terms of yield (76–88%) and selectivity (∼86:14 er). Furthermore, we were pleased to see that our finding that the different configurations of allyl boronic acid ester led to different stereoisomers translated to other substrates (**5d**, **5f**) and in these cases both enantiomers could be prepared with similar selectivity. In analogy to the literature known sporochnol precursor **5d** we could assign the stereochemical outcome of the *Z*-allylic boronate as the (*S*)-enantiomer, whereas the *E*-allyl boronic acid ester gave the *R*-enantiomer. Scaling experiments using the sporochnol precursor **5d** (1 mmol/80%, 3 mmol/74%) delivered the same level of stereoselectivity while maintaining an efficient reaction even with a reduced amount of Pd-source (1 mol%) and ligand (4 mol%). Investigating the sterical boundaries of our reaction (**5g**, **5h**, **5i**), we observed that *para*- and *meta*-substitution has no significant influence on the reaction outcome, while *ortho*-substitution (**5i**) leads to a decrease in reaction efficiency and stereoselectivity (48%, 67:33 er). When we tested heteroaryl halides, similar to our optimization substrate containing a different heteroatom (**5l**, **5m**), we obtained similarly efficient reactions, with slightly lowered enantioselectivity. For the electron-deficient pyridine **5n**, regio-as well as stereoselectivity (49:51 α/γ, 73:27 er) were poor, which further underlines the importance of electronic properties of the applied halide. Moreover, we wanted to investigate whether the method also transfers to other allyl boronic acid esters.

**Figure 2:**
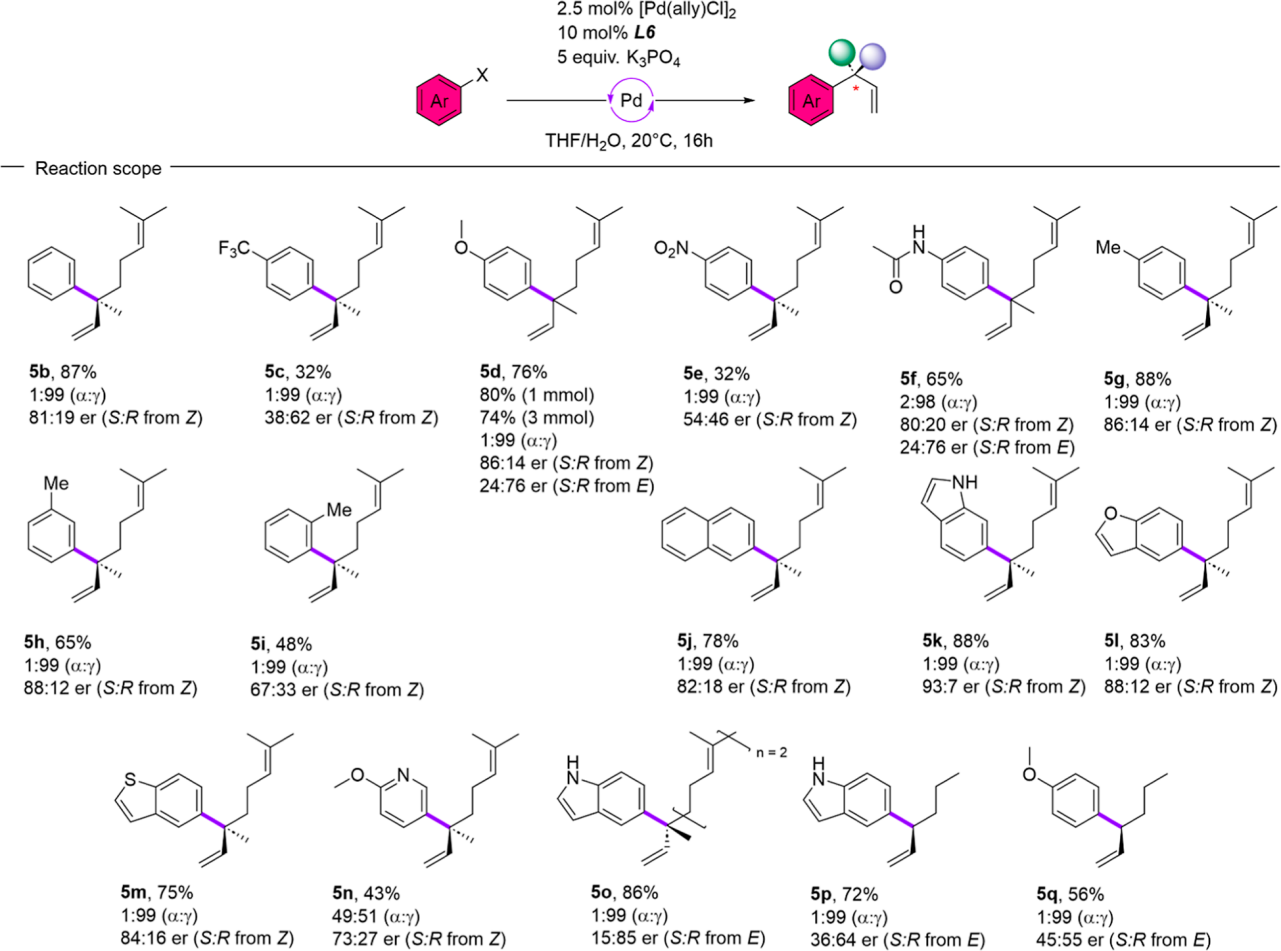
Scope of the asymmetric γ-selective Suzuki–Miyaura cross-coupling reaction.

For that purpose, we prepared the allylic boronates derived from (*E*)-hept-2-en-1-ol (**B5**) and (*E*,*E*)-farnesol (**B6**) (see Supporting Information Section 3). The subsequent γ-selective cross-coupling reactions using these boronates gave a highly efficient and selective reaction (**5q**, 1:99 α/γ, 15:85 er) for the farnesol derived allyl boronic acid ester (**B6**). The heptenol variant (**B5**) delivered unsatisfying results (**5p**, **5q**) concerning the stereoselectivity of the reaction, which might indicate that for efficient energetical discrimination between the diastereomeric transmetalation transition-states, a substituent sterically more demanding than hydrogen is necessary. This suggests that the method is more suited for the establishment of all carbon quaternary stereogenic centers than for stereogenic centers that are less sterically encumbered.

### Natural Product and Analog Synthesis

To further highlight the synthetic utility of our catalytic system, we showcased the applicability of the reaction for natural product synthesis. Consequently, we applied the asymmetric γ-selective cross-coupling conditions onto 4-iodophenol (**6**) (Scheme 4). Delightfully, the reaction proceeded with excellent levels of regioselectivity (1:99 α/γ) and even higher enantioselectivity [96:4 er (*S*)/(*R*)] than we obtained beforehand. In this manner the natural product (+)-(*S*)-sporochnol A (**2**) is accessible in one step from a commercially available starting material using this approach. The usage of an unprotected phenol further showcases the functional group compatibility as well as the beneficial effect of electron donating groups for asymmetric γ-selective cross-coupling. Furthermore, to the best of our knowledge, this represents the shortest and first protective group free total synthesis of (+)-(*S*)-sporochnol A (**2**).

**Scheme 4:**
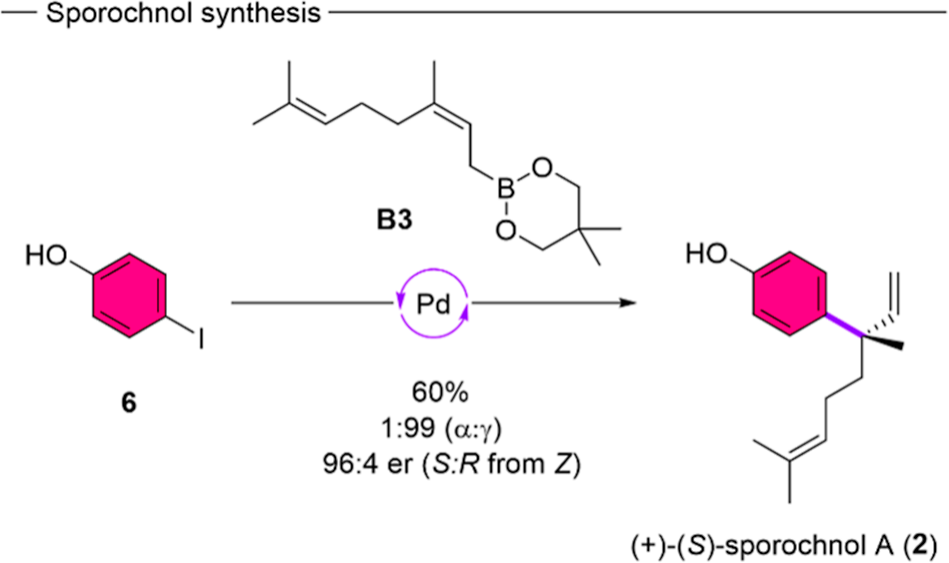
Total Synthesis of Sporochnol

Due to our ongoing interest in prenylated natural products and their biological activity and to further assess the viability of our catalytic system for late-stage functionalization, we aimed to synthesize higher order prenylated analogs of the lansai B scaffold and the synthetic precursor tryptophan–tryptophan diketopiperazine (**7**) (the 2,5-diketopiperazine core is highlighted in green, Scheme 5). Lansai B, which has been isolated from *Streptomyces* sp. (SUC1), is a weakly active agent against breast cancer cell lines (IC_50_ = 15 μg mL^–1^), whereas 2,5-diketopiperazines in general, and especially prenylated diketopiperazines have shown potential for a multitude of pharmaceutical applications.^9,33,34^

**Scheme 5:**
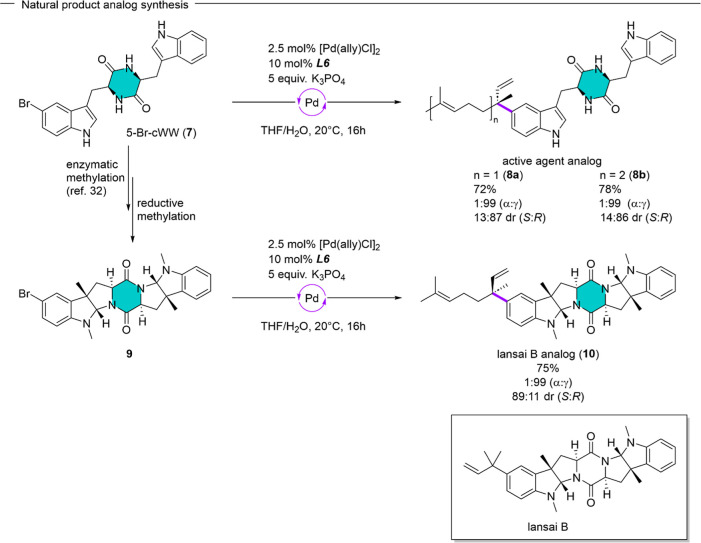
Preparation of Natural Product and Active Agent Analogs via Asymmetric γ-Selective Suzuki–Miyaura Cross-Coupling Methodology

According to our recently disclosed chemoenzymatic procedure we prepared the brominated tryptophan–tryptophan diketopiperazine **7** (5-Br-cWW) (Scheme 5).^35^ Subsequently, we used or catalytic system to introduce the branched geranyl (**8a**) and the branched farnesyl (**8b**) side chain onto the core scaffold. In both cases the γ-selective cross coupling reaction proceeded efficiently (72%/78%), with perfect levels of regioselectivity (1:99 α/γ) and high levels of diastereoselectivity [13:87/14:86 er (*S*)/(*R*)]. After enzymatic C3-methylation and reductive methylation of 5-Br-cWW (**7**), we subjected the pyrrolo-indoline **9** to the reaction conditions to obtain the geranylated lansai B analog **10**, with equally high levels of regio- and stereoselectivity [1:99 α/γ, 89:11 er (*S*)/(*R*)] in a very good yield (75%). This further highlights the utility of our catalytic system for the late-stage derivatization of natural products and their analogs.

## Conclusion

In summary, we established a new catalytic method for the asymmetric γ-selective Suzuki–Miyaura cross-coupling of aryl halides and allylboronic acid esters, which is especially suitable for the direct synthesis of branched heteroaryl-terpenoids. Our palladium-based catalytic system is able to form both stereoisomers of an all-carbon quaternary stereogenic center directly onto the aromatic scaffold in a selective manner, starting from simple and easily accessible starting materials. To the best of our knowledge, our catalytic method represents the first approach for asymmetric γ-selective cross-coupling leading to quaternary stereogenic centers. Moreover, we could demonstrate, that our catalytic method is applicable to a wider range of heteroaryl halides and different allyl boronic acid esters, meaning our methodology can serve as additional asset in the toolbox for asymmetric Csp^2^–Csp^3^ cross-coupling. We could further showcase the synthetic utility of our catalytic system by applying it for natural product synthesis and late-stage derivatization of potential active agents and natural product analogs.

## Methods

### General Procedure for Asymmetric γ-Selective Suzuki–Miyaura Coupling

An oven-dried screw cap vial was charged with the [Pd­(allyl)­Cl]_2_ (5 μmol, 2.5 mol%), the ligand (0.02 mmol, 10 mol%), and the aryl halide (0.2 mmol) under air. The vial was closed using a screw cap with a polytetrafluoroethylene/silicone septa and sealed using parafilm. The vial was evacuated and refilled with nitrogen three times using a needle, piercing through the septum. The allylic boronate (0.24 mmol, 1.2 equiv) dissolved in THF (0.4 mL) was added followed by an aqueous solution (2.5 M, 0.4 mL) of K_3_PO_4_. The reaction mixture was stirred vigorously overnight at room temperature. Afterward the reaction mixture was diluted using ethyl acetate and transferred into a separatory funnel. The aqueous phase was extracted three times using ethyl acetate. The combined organic phase was washed with brine, dried over magnesium sulfate, and concentrated *in vacuo*. Purification of the crude reaction product was achieved via column chromatography.

## Supplementary Material



## Data Availability

The data underlying this study are available in the published article and its Supporting Information.

## Notes

NMR spectra, additional experimental details, materials and methods, including NMR data and HPLC chromatograms (PDF)

## Abbreviations

THF: tetrahydrofuran
er: enantiomeric ratio
dr: diastereomeric ratio

## References

[1] Rudolf J. D., Alsup T. A., Xu B., Li Z. (2021). Bacterial terpenome. Nat. Prod. Rep..

[2] Gershenzon J., Dudareva N. (2007). The function of terpene natural products in the natural
world. Nat. Chem. Biol..

[3] Câmara J. S., Perestrelo R., Ferreira R., Berenguer C. V., Pereira J. A. M., Castilho P. C. (2024). Plant-Derived Terpenoids: A Plethora
of Bioactive Compounds with Several Health Functions and Industrial
Applications—A Comprehensive Overview. Molecules.

[4] Wang Q., Zhao X., Jiang Y., Jin B., Wang L. (2023). Functions
of Representative Terpenoids and Their Biosynthesis Mechanisms in
Medicinal Plants. Biomolecules.

[5] Wang, G. ; Tang, W. ; Bidigare, R. R. Terpenoids As Therapeutic Drugs and Pharmaceutical Agents. In Natural Products: Drug Discovery and Therapeutic Medicine; Zhang, L. , Demain, A. L. , Eds.; Humana Press: Totowa, NJ, 2005; pp 197–227.

[6] Cragg G. M., Pezzuto J. M. (2015). Natural Products as a Vital Source
for the Discovery
of Cancer Chemotherapeutic and Chemopreventive Agents. Med. Princ. Pract..

[7] Shen B. (2015). A New Golden
Age of Natural Products Drug Discovery. Cell.

[8] Xu B., Li Z., Alsup T. A., Ehrenberger M. A., Rudolf J. D. (2021). Bacterial Diterpene
Synthases Prenylate Small Molecules. ACS Catal..

[9] Tuntiwachwuttikul P., Taechowisan T., Wanbanjob A., Thadaniti S., Taylor W. C. (2008). Lansai A–D,
secondary metabolites from Streptomyces
sp. SUC1. Tetrahedron.

[10] Raju R., Piggott A. M., Huang X.-C., Capon R. J. (2011). Nocardioazines:
A Novel Bridged Diketopiperazine Scaffold from a Marine-Derived Bacterium
Inhibits P-Glycoprotein. Org. Lett..

[11] Shen Y.-C., Tsai P. I., Fenical W., Hay M. E. (1992). Secondary metabolite
chemistry of the caribbean marine alga Sporochnus bolleanus: A basis
for herbivore chemical defence. Phytochemistry.

[12] Zhang X., Zhao W., Wang Y., Lu J., Chen X. (2016). The Chemical
Constituents and Bioactivities of Psoralea corylifolia Linn.: A Review. Am. J. Chin. Med..

[13] Takashima M., Sakai H. (1960). A New Toxic Substance, Teleocidin,
Produced by Streptonayces Part
II. Biological Studies of Teleocidin. Bull.
Agric. Chem. Soc. Jpn..

[14] Adarsh
Krishna T. P., Edachery B., Athalathil S. (2022). Bakuchiol
– a natural meroterpenoid: structure, isolation, synthesis
and functionalization approaches. RSC Adv..

[15] Wang S., Liu M., Lewin N. E., Lorenzo P. S., Bhattacharrya D., Qiao L., Kozikowski A. P., Blumberg P. M. (1999). Probing the Binding
of Indolactam-V to Protein Kinase C through Site-Directed Mutagenesis
and Computational Docking Simulations. J. Med.
Chem..

[16] Takahashi M., Shioura Y., Murakami T., Ogasawara K. (1997). The absolute
configuration of (+)-sporochnol A, the fish deterrent from the Caribbean
marine alga Sporochnus bolleanus. Tetrahedron:
Asymmetry.

[17] Mascarenhas-Melo F., Ribeiro M. M., Kahkesh K. H., Parida S., Pawar K. D., Velsankar K., Jha N. K., Damiri F., Costa G., Veiga F., Paiva-Santos A. C. (2024). Comprehensive review of the skin
use of bakuchiol: physicochemical properties, sources, bioactivities,
nanotechnology delivery systems, regulatory and toxicological concerns. Phytochem. Rev..

[18] Cardellina J. H., Marner F.-J., Moore R. E. (1979). Seaweed Dermatitis:
Structure of
Lyngbyatoxin A. Science.

[19] Arcoleo J. P., Weinstein I. B. (1985). Activation of protein kinase C by
tumor promoting phorbol
esters, teleocidin and aplysiatoxin in the absence of added calcium. Carcinogenesis.

[20] Moghadam F. A., Hicks E. F., Sercel Z. P., Cusumano A. Q., Bartberger M. D., Stoltz B. M. (2022). Ir-Catalyzed Asymmetric Allylic Alkylation of Dialkyl
Malonates Enabling the Construction of Enantioenriched All-Carbon
Quaternary Centers. J. Am. Chem. Soc..

[21] Sander L., Müller J. M., Stark C. B. W. (2025). Iridium-Catalyzed Regio- and Enantioselective
Reverse Prenylation of Tryptamines and Other 3-Substituted Indoles. J. Am. Chem. Soc..

[22] Baeckvall J.-E., Persson E. S., Bombrun A. (1994). Regiocontrol in copper-catalyzed
cross coupling of allylic chlorides with aryl Grignard reagents. J. Org. Chem..

[23] Rodríguez D., Sestelo J. P., Sarandeses L. A. (2003). Copper-Catalyzed Regioselective Allylic
Substitution Reactions with Indium Organometallics. J. Org. Chem..

[24] Legros J.-Y., Fiaud J.-C. (1990). Palladium-catalyzed phenylation of allylic acetates
by tetraphenylborate anion. Tetrahedron Lett..

[25] Gao F., McGrath K. P., Lee Y., Hoveyda A. H. (2010). Synthesis of Quaternary
Carbon Stereogenic Centers through Enantioselective Cu-Catalyzed Allylic
Substitutions with Vinylaluminum Reagents. J.
Am. Chem. Soc..

[26] Gao F., Lee Y., Mandai K., Hoveyda A. H. (2010). Quaternary Carbon Stereogenic Centers
through Copper-Catalyzed Enantioselective Allylic Substitutions with
Readily Accessible Aryl- or Heteroaryllithium Reagents and Aluminum
Chlorides. Angew. Chem., Int. Ed..

[27] Yang Y., Buchwald S. L. (2013). Ligand-Controlled
Palladium-Catalyzed Regiodivergent
Suzuki–Miyaura Cross-Coupling of Allylboronates and Aryl Halides. J. Am. Chem. Soc..

[28] Yamamoto Y., Takada S., Miyaura N. (2006). γ-Selective Cross-coupling
of Potassium Allyltrifluoroborates with Aryl and 1-Alkenyl Bromides
Catalyzed by a Pd­(OAc)­2/D-t-BPF Complex. Chem.
Lett..

[29] Yamamoto Y., Takada S., Miyaura N. (2006). Asymmetric
Cross-coupling of Potassium
2-Butenyltrifluoroborates with Aryl and 1-Alkenyl Bromides Catalyzed
by a Pd­(OAc)­2/Josiphos Complex. Chem. Lett..

[30] Yamamoto Y., Takada S., Miyaura N., Iyama T., Tachikawa H. (2009). γ-Selective
Cross-Coupling Reactions of Potassium Allyltrifluoroborates with Haloarenes
Catalyzed by a Pd(0)/D-t-BPF or Pd(0)/Josiphos ((R,S)-CyPF-t-Bu) Complex:
Mechanistic Studies on Transmetalation and Enantioselection. Organometallics.

[31] Dutheuil G., Selander N., Szabó K. J., Aggarwal V. K. (2008). Direct Synthesis
of Functionalized Allylic Boronic Esters from Allylic Alcohols and
Inexpensive Reagents and Catalysts. Synthesis.

[32] Raducan M., Alam R., Szabó K. J. (2012). Palladium-Catalyzed
Synthesis and
Isolation of Functionalized Allylboronic Acids: Selective, Direct
Allylboration of Ketones. Angew. Chem., Int.
Ed..

[33] Martins M. B., Carvalho I. (2007). Diketopiperazines:
biological activity and synthesis. Tetrahedron.

[34] Borthwick A. D. (2012). 2,5-Diketopiperazines:
Synthesis, Reactions, Medicinal Chemistry, and Bioactive Natural Products. Chem. Rev..

[35] Schatton M., Haase M., Tenhaef J., Gronkowsky C., Noack S., Pietruszka J. (2025). Chemoenzymatic Total Synthesis of
Lansai B. Chem. - Eur. J..

