# Supplementary material for: Stereoselective Prenylation of Aryl- and Heteroaryl Halides: γ‑Selective Suzuki–Miyaura Coupling as a Tool for Asymmetric Csp2–Csp3 Cross-Coupling
Source: J Org Chem. 2026 May 12;91(20):6929–37. doi: 10.1021/acs.joc.6c00410 (PMC13200185; doi:10.1021/acs.joc.6c00410)
Supplement: Supplementary file 1 [file jo6c00410_si_001.pdf]

Stereoselective Prenylation of Aryl- and Heteroaryl Halides: γ-selective Suzuki-Miyaura coupling as a tool for asymmetric Csp^2-Csp^3 cross-coupling

Supporting Information

Cornelius Pawlowsky[a,b], Tim Leipertz[b], Birgit Henssen[a,b], Mona Haase[a], Jörg Pietruszka[a,b]

a. Institute for Bioorganic Chemistry & Bioeconomy Science Center (BioSC), Heinrich Heine University Düsseldorf in Forschungszentrum Jülich
b. Institute of Bio- and Geosciences (IBG-1: Bioorganic Chemistry) & Bioeconomy Science Center (BioSC), Forschungszentrum Jülich

Table of Contents

1. General Information S2
   1. Chemicals S2
   2. Analytical Information and devices S2
2. Optimization of reaction conditions S3
3. Synthesis of allyl boronic acid esters S7
   General procedures for allyl boronic acid ester preparation: S7
   Compound characterization S8
4. γ-selective Suzuki-Miyaura coupling S12
   General procedure for asymmetric γ-selective Suzuki-Miyaura coupling: S12
   Compound characterization S12
5. Application in natural product and derivative synthesis S31
6. Biological work S40
7. Copy of NMR spectra S43
8. References S77

1. General Information

1. Chemicals

Chemicals were purchased from Sigma-Aldrich Co., Alfa Aesar GmbH & Co. KG, Merck KGaA or BLD Pharmatech GmbH and used without further purification. Anhydrous dichloromethane, diethyl ether and tetrahydrofuran were taken from the solvent purifier MB SPS-800 by MBraun. Silica gel 60 (0.040 – 0.063 mm, 230 – 400 mesh) for column chromatography was obtained from Macherey Nagel. Anhydrous solid reagents were stored in a desiccator under an atmosphere of N_2. The Ligands ***L1*** – ***L8*** have been purchased from BLD Pharmatech GmbH.

2. Analytical Information and devices

NMR spectra were measured on the spectrometer Bruker Avance/DRX 600 at a frequency of 600 MHz (^{1}H) and 151 MHz (^{13}C) as well as Bruker Avance/DRX 300 at a frequency of 282 MHz (^{19}F), 96 MHz (^{11}B), 300 MHz (^{1}H) and 75.5 MHz (^{13}C) in the indicated solvent. The ^{1}H- and ^{13}C-spectra were referenced to the solvent peak ($CDCl_3$ δ = 7.26 ppm (^{1}H), δ = 77.16 ppm (^{13}C)). Data were evaluated with MNova (MestReNova) version 14.1.2 by Mestrelab Research. Coupling constants J are given in Hz and chemical shifts δ in ppm (parts per million). Multiplicities are abbreviated as the following: singlet (s), broad singlet (brs), doublet (d), triplet (t), quartet (q), multiplet (m). Chiral HPLC analysis was performed using Dionex UltiMate 3000 from Thermo Scientific. High resolution mass spectrometry (HRMS) were measured using a timsTOF Pro 2 (Bruker Daltonics) with electron spray ionization (ESI/TOF analyzer) or an Orbitrap Exploris™ 240 (Thermo Fisher Scientific) with electron ionization (EI/quadrupol analyzer). Low resolution mass spectrometry (MS) was conducted using the expression CMS system by Advion, Inc. in combination with an atmospheric pressure chemical ionization (APCI) or electron spray ionization (ESI). Gas chromatographic mass spectrometry (GC-MS(EI)) was conducted using the Thermo Scientific TRACE 1310 gas chromatograph (ISQ QD Single Quadrupole Mass Spectrometer, Helium). Optical rotation was measured with A.Krüss P8000-TF at 10 °C (c in g/100 ml). Melting points were measured with Stuart Scientific Melting Point Apparatus SMP3. IR spectra were recorded using the SpectrumTwo FT-IR by PerkinElmer with attenuated total reflection (ATR). The absorption bands are given in units of wave numbers (cm^{-1}). Elemental analysis was measured at the Heinrich Heine University Düsseldorf (Elementar, Vario Micro Cube). Cell lysis was performed using an ultrasonic cell disruptor (Branson Sonifier II "Model W-250", Heinemann). The determination of enantiomeric ratios was conducted using chiral HPLC. Unless otherwise noted HPLC was conducted at a column temperature of 20 °C. Reactions that require heating were heated using an oil bath.

2. Optimization of reaction conditions

General procedure for optimization reactions:

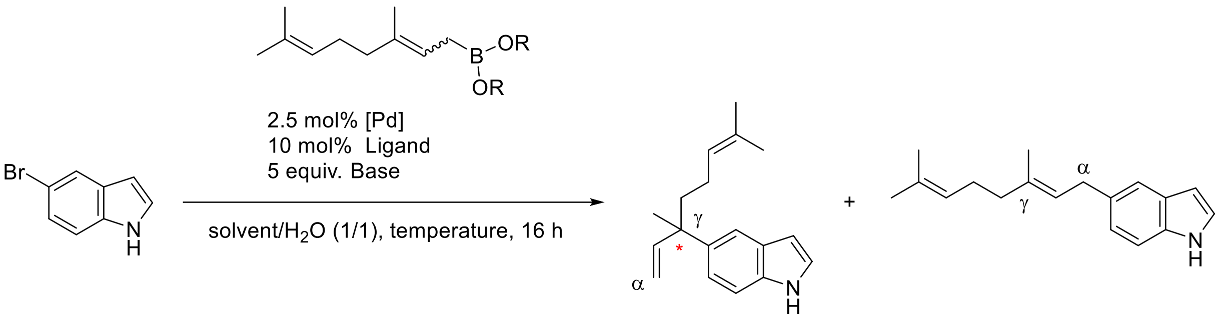

Scheme S1 *Reaction scheme for the optimization of the γ-selective coupling reaction.*

An oven dried screw cap vial was charged with the Pd-source (5 µmol, 2.5 mol%), the ligand (0.02 mmol,10 mol%) and the aryl halide (0.2 mmol, 1 equiv.) under air. The vial was closed using a screw cap with a PTFE/silicone septa and sealed using parafilm®. The vial was evacuated and refilled with nitrogen using a needle, piercing through the septum, three times. The allylic boronate (0.24 mmol, 1.2 equiv.) dissolved in the applied solvent (0.4 mL, 2 ml/mmol) was added followed by an aqueous solution (2.5 M, 0.4 mL, 2 ml/mmol) of the base. The reaction mixture was stirred vigorously over night at the given temperature. Afterwards the reaction mixture was diluted using ethyl acetate and filtered through a short plug of silica eluting with ethyl acetate. The filtrate was concentrated *in vacuo* and 1,3,5-trimethoxybenzene (0.05 mmol) dissolved in deuterated chloroform was added. The crude reaction mixture was analyzed via ^{1}H-qNMR for determination of yield and regioselectivity. Subsequently an aliquot of the sample was taken up to dryness, redissolved in *n*-heptane (final concentration: 1 mg/mL) and analyzed via chiral HPLC for determination of the enantiomeric ratio.

Ligand screening

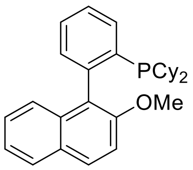

L1

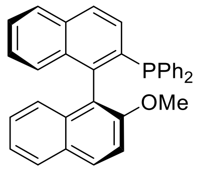

L2

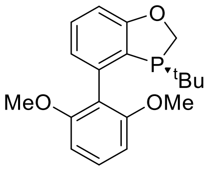

L3

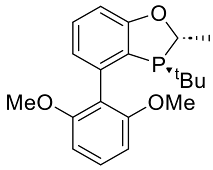

L4

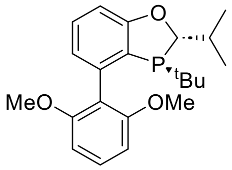

L5

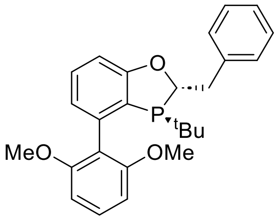

L6

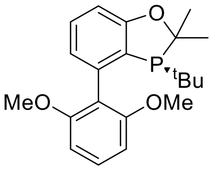

L7

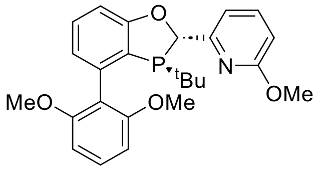

L8

Table S1 *Results of the ligand screening.*

**Entry**	**Ligand**	**Yield (%)**[a]	**α:γ**[b]	**er (ee)**[c]
1	***L1***	84	8:92	-
2	***L2***	traces	93:7[d]	-
3	***L3***	traces	-	-
4	***L4***	23	42:58	8:13 (74%)
5	***L5***	79	13:87	11:89 (78%)
6	***L6***	51	10:90	93:7 (86%)
7	***L7***	64	18:82	68:32 (36%)
8	***L8***	10	78:22	77:23 (54%)

Standard reaction conditions: 5-Bromoindole (0.2 mmol), neryl-Bpin (0.24 mmol), $[Pd(allyl)Cl]_2$ (0.005 mmol), Ligand (0.020 mmol), THF (0.4 mL), aq. K_3PO_4 (0.4 mL (2.5 M)), 40 °C, 16 h. [a]Determined via ^{1}H-qNMR using 1,3,5-trimethoxybenzene as internal standard. [b]Determined via ^{1}H-NMR. [c]determined by HPLC analysis via comparison to an authentic racemic sample

Nucleophile Screening

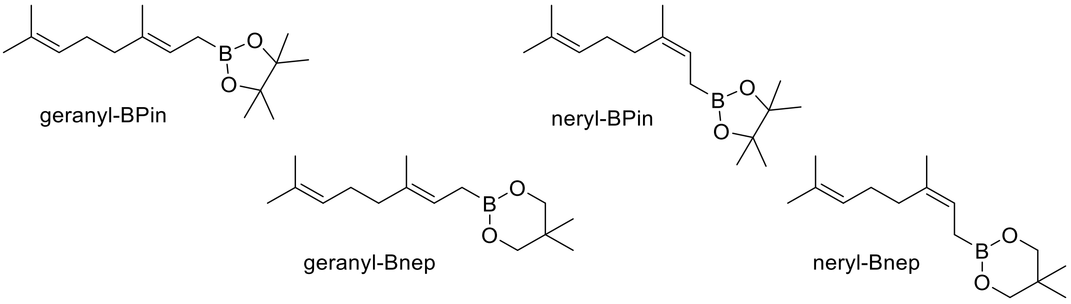

Table S2 Results of the nucleophile screening.

Entry	Nucleophile	Ligand	Temperature	Yield (%)[a]	α:γ[b]	er (ee)[c]
1	geranyl-Bpin	***L5***	40 °C	79	13:87	11:89 (78%)
2	geranyl-Bpin	***L2***	40 °C	41	33:67	42:58 (16%)
3	neryl-Bpin	***L5***	40 °C	56	14:86	90:10 (80%)
4	neryl-BPin	***L6***	40 °C	51	10:90	93:7 (86%)
5	neryl-BPin	***L5***	rt	44	34:66	87:13 (74%)
6	neryl-BPin	***L6***	rt	7	88:12	55:45 (10%)
7	neryl-Bnep	***L5***	40 °C	75	9:91	91:9 (82%)
8	neryl-Bnep	***L6***	40 °C	33	51:49	83:17 (66%)
9	neryl-Bnep	***L5***	rt	64	13:87	92:8 (84%)
10	neryl-Bnep	***L6***	rt	85	2:98	95:5 (90%)
11	geranyl-Bpin	***L6***	rt	35	38:62	78:22
12	geranyl-Bnep	***L6***	rt	78	1:99	7:93

Standard reaction conditions: 5-Bromoindole (0.2 mmol), allylic boronate (0.24 mmol), $[Pd(allyl)Cl]_2$ (0.005 mmol), Ligand (0.020 mmol), THF (0.4 mL), aq. K_3PO_4 (0.4 mL (2.5 M)), 40 °C, 16 h. [a]Determined via ^{1}H-qNMR using 1,3,5-trimethoxybenzene as internal standard. [b]Determined via ^{1}H-NMR. [c]Determined by HPLC analysis via comparison to an authentic racemic sample

Pd-Source Screening

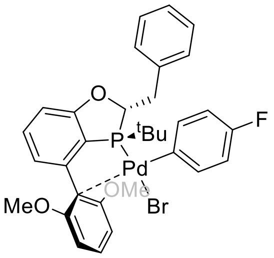

P1

Table S3 Results of the Pd source screening.

Entry	Pd-Source	Ligand	Yield (%)[a]	α:γ[b]	er (ee)[c]
1	***P1***	-	44	11:89	94:6 (88%)

2	***P1***	***L6*** (5 mol%)	38	1:99	92:8 (84%)

Standard reaction conditions: 5-Bromoindole (0.2 mmol), neryl-Bnep (0.24 mmol), Pd-Source (0.005 mmol), Ligand (0.020 mmol), THF (0.4 mL), aq. K_3PO_4 (0.4 mL (2.5 M)), 40 °C, 16 h, [a]determined via ^{1}H-qNMR using 1,3,5-trimethoxybenzene as internal standard. [b]determined via ^{1}H-NMR. [c]determined by HPLC analysis via comparison to an authentic racemic sample

Solvent Screening

Table S4 *Results of the solvent screening.*

**Entry**	**Solvent**	**Yield (%)[a]**	**α:γ[b]**	**er (ee)[c]**
1	THF	85	2:98	95:5 (90%)
2	2-MeTHF	51	1:99	90:5 (90%)
3	CPME	13	1:99	90:5 (90%)
4	^{i}PrOH	10	1:99	86:14 (72%)
5	Cyrene™	traces	-	-
6	DMF	62	8:92	82:18 (64%)
7	Toluene	58	4:96	94:6 (88%)
8	Dioxane	94	1:99	93:7 (86%)

Standard reaction conditions: 5-Bromoindole (0.2 mmol), neryl-Bnep (0.24 mmol), $[Pd(allyl)Cl]_2$ (0.005 mmol), Ligand (0.020 mmol), solvent (0.4 mL), aq. K_3PO_4 (0.4 mL (2.5 M)), 40 °C, 16 h, [a]determined via ^{1}H-qNMR using 1,3,5-trimethoxybenzene as internal standard. [b]determined via ^{1}H-NMR. [c]determined by HPLC analysis via comparison to an authentic racemic sample

Base Screening

Table S5 *Results of the base screening.*

**Entry**	**Base**	**Yield (%)[a]**	**α:γ[b]**	**er (ee)[c]**
1	K_3PO_4	85	2:98	95:5 (90%)
2	K_2CO_3	58	2:98	94:6 (88%)
3	KOH	67	1:99	93:7 (86%)
4	KF	Traces	-	-
5	KOAc	13	1:99	93:7 (86%)

Standard reaction conditions: 5-Bromoindole (0.2 mmol), allylic boronate (0.24 mmol), $[Pd(allyl)Cl]_2$ (0.005 mmol), Ligand (0.020 mmol), THF (0.4 mL), aq. Base (0.4 mL (2.5 M)), 40 °C, 16 h, [a]determined via ^{1}H-qNMR using 1,3,5-trimethoxybenzene as internal standard. [b]determined via ^{1}H-NMR. [c]determined by HPLC analysis via comparison to an authentic racemic sample

3. Synthesis of allyl boronic acid esters

General procedures for allyl boronic acid ester preparation:

General Procedure A

This synthetic procedure was adapted from a procedure published by Aggarwal *et al*.[1] A Schlenk tube was charged with *p*-toluenesulfonic acid hydrate (0.05 equiv.) and di-µ-chlorobis{2-[(di-methylamino)methyl]phenyl-C,N}dipalladium(II) (0.05 equiv.). The tube was evacuated and refilled with nitrogen three times. DMSO and methanol (1:1, 2 mL/mmol allylic alcohol) were added, followed by the corresponding allylic alcohol (1.0 equiv.). Bis(pinacolato)diboron was added (2.0 equiv.) and the mixture was stirred at 50 °C overnight. The mixture was cooled to room temperature and diluted with petrol ether (approx. 20 times of the reaction mixtures volume) filtered through a short plug of Celite® and washed with brine (5x). The resulting organic phase was concentrated in vacuo and purified via column chromatography.

General Procedure B

This synthetic procedure was adapted from a procedure published by Szabo *et al.*[2] A round bottom flask was charged with tetrahydroxydiboron (1.2 equiv.). The flask was evacuated and refilled with nitrogen (3x). DMSO and methanol (1:1, 2 mL/mmol allylic alcohol) were added, followed by the corresponding allylic alcohol (1.0 equiv.). The mixture was stirred vigorously for 15 minutes, after which an aqueous solution of H_2PdCl_4 (5 mol%, 0.33 M) was added dropwise. The mixture was stirred vigorously at room temperature until complete consumption of the allylic alcohol. To the reaction mixture pinacol (2 equiv.) was added and the mixture was stirred vigorously for another 2 hours at room temperature. Afterwards the mixture was diluted using petrol ether (approx. 20 times of the reaction mixtures volume), filtered through a short plug of Celite® and washed with brine (5x). The resulting organic phase was concentrated in vacuo and purified via column chromatography.

General Procedure C

This synthetic procedure was adapted from a procedure published by Szabo *et al.*[2] A round bottom flask was charged with bis(neopentylglycolato)diboron (1.2 equiv.). The flask was evacuated and refilled with nitrogen (3x). DMSO and methanol (1:1, 2 mL/mmol allylic alcohol) were added, followed by the corresponding allylic alcohol (1.0 equiv.). The mixture was stirred vigorously for 15 minutes, after which an aqueous solution of H_2PdCl_4 (5 mol%, 0.33 M) was added dropwise. The mixture was stirred vigorously until complete consumption of the allylic alcohol. Afterwards the mixture was diluted using petrol ether (approx. 20 times of the reaction mixtures volume), filtered through a short plug of Celite® and washed with brine (5x). The resulting organic phase was concentrated in vacuo and purified via column chromatography.

Preparation of Pd catalyst H_2PdCl_4

This synthetic procedure was adapted from a procedure published by Szabo *et al.*[3] A screw cap vial was charged with $PdCl_2$ (1.0 equiv., 0.5 mmol, 88.7 mg), closed with a screw cap and sealed with parafilm®. The vial was evacuated and refilled with nitrogen (3x), after which water (1.38 mL, 2.76 mL/mmol) was added. The mixture was stirred vigorously and concentrated hydrochloric acid (37% in water, 3 equiv., 0.12 mL) was added dropwise. The mixture was stirred and heated to 70 °C for one hour, after which it was used as catalyst for further reactions.

Compound characterization

(*Z*)-2-(3,7-dimethylocta-2,6-dien-1-yl)-4,4,5,5-tetramethyl-1,3,2-dioxaborolane (B1)

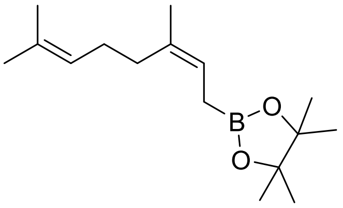

The title compound was prepared according to the general procedure B using the allylic alcohol nerol (5 mmol). The crude product was purified using column chromatography (silica gel; pentane:Et_2O 98:2) to afford the allylic boronate (*Z*)-2-(3,7-dimethylocta-2,6-dien-1-yl)-4,4,5,5-tetramethyl-1,3,2-dioxaborolane (937 mg, 3.55 mmol, 71%) as a colorless oil. The analytical data is in accordance with the published literature.[4] The title compound was also prepared according to general procedure C (71%).

^{1}H NMR ($CDCl_3$, 600 MHz) δ 5.21 (1H, td, *J* = 7.7, 1.6 Hz), 5.10 (1H, dddt, *J* = 6.9, 5.5, 2.9, 1.4 Hz), 2.05 – 1.95 (4H, m), 1.66 (3H, q, *J* = 1.4 Hz), 1.65 (3H, s), 1.58 (3H, s), 1.21 (12H, s)

^{13}C{^{1}H} NMR ($CDCl_3$, 151 MHz) δ 135.3, 131.4, 124.6, 119.2, 83.2, 31.9, 26.5, 25.8, 24.8, 23.6, 17.7

^{11}B NMR ($CDCl_3$, 96 MHz) δ 33.2

MS (APCI): m/z calc. for $C_{16}H_{29}BO_2$+H^+ 265.2; found 265.1

(*E*)-2-(3,7-dimethylocta-2,6-dien-1-yl)-4,4,5,5-tetramethyl-1,3,2-dioxaborolane (B2)

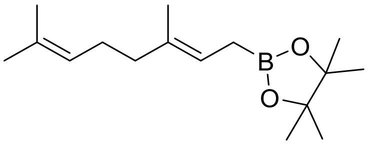

The title compound was prepared according to the general procedure A using the allylic alcohol geraniol (5 mmol). The crude product was purified using column chromatography (silica gel; pentane:Et_2O 98:2) to afford the allylic boronate (*E*)-2-(3,7-dimethylocta-2,6-dien-1-yl)-4,4,5,5-tetramethyl-1,3,2-dioxaborolane (1.070 g, 4.05 mmol, 81%) as a colorless oil. The analytical data is in accordance with the published literature.[1] The title compound was also prepared according to general procedure C (81%).

^{1}H NMR ($CDCl_3$, 600 MHz) δ 5.22 (1H, tq, *J* = 7.6, 1.2 Hz), 5.07 (1H, ddp, *J* = 7.0, 5.7, 1.4 Hz), 2.07 – 1.92 (4H, m), 1.67 – 1.62 (3H, m), 1.59 – 1.53 (6H, m), 1.21 (12H, s)

^{13}C{^{1}H} NMR ($CDCl_3$, 151 MHz) δ 135.1, 131.1, 124.6, 118.6, 83.1, 77.4, 39.8, 26.9, 25.8, 25.1, 24.8, 17.8, 16.0

^{11}B NMR ($CDCl_3$, 96 MHz) δ 33.0

MS (APCI): m/z calc. for $C_{16}H_{29}BO_2$+H^+ 265.2; found 265.1

(*Z*)-2-(3,7-dimethylocta-2,6-dien-1-yl)-5,5-dimethyl-1,3,2-dioxaborinane (B3)

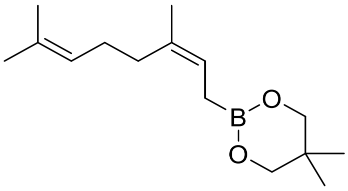

The title compound was prepared according to the general procedure C using the allylic alcohol nerol (5 mmol). The crude product was purified using column chromatography (silica gel; pentane:Et_2O 96:4) to afford the allylic boronate (*Z*)-2-(3,7-dimethylocta-2,6-dien-1-yl)-5,5-dimethyl-1,3,2-dioxaborinane (1.100 g, 4.40 mmol, 88%) as a colorless oil.

^{1}H NMR ($CDCl_3$, 600 MHz) δ 5.28 – 5.23 (1H, m), 5.16 – 5.10 (1H, m), 3.59 (4H, s), 2.07 – 1.99 (4H, m), 1.70 (3H, s), 1.68 (3H, s), 1.61 (3H, s), 1.56 (2H, d, *J* = 7.9 Hz), 0.95 (6H, d, *J* = 0.8 Hz)

^{13}C{^{1}H} NMR ($CDCl_3$, 151 MHz) δ 134.7, 131.4, 124.8, 120.4, 72.3, 32.0, 31.8, 26.6, 25.9, 23.7, 22.0, 17.8

^{11}B NMR ($CDCl_3$, 96 MHz) δ 29.4

HRMS (EI): m/z calc. for $C_{15}H_{27}BO_2$+H^+ 250.2099; found 250.2109

MS (APCI): m/z calc. for $C_{15}H_{27}BO_2$+H^+ 251.2; found 251.3

IR (ATR, film) ṽ [cm^{-1}] = 2962, 2928, 1477, 1415, 1376, 1297, 1254, 1175, 1070, 1008, 813, 667.

(*E*)-2-(3,7-dimethylocta-2,6-dien-1-yl)-5,5-dimethyl-1,3,2-dioxaborinane (B4)

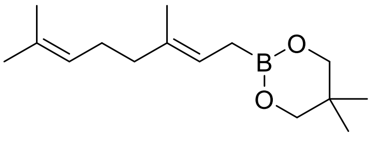

The title compound was prepared according to the general procedure C using the allylic alcohol geraniol (5 mmol). The crude product was purified using column chromatography (silica gel; pentane:Et_2O 96:4) to afford the allylic boronate (*E*)-2-(3,7-dimethylocta-2,6-dien-1-yl)-5,5-dimethyl-1,3,2-dioxaborinane (1.063 g, 4.25 mmol, 85%) as a colorless oil.

^{1}H NMR ($CDCl_3$, 600 MHz) δ 5.25 (1H, t, *J* = 7.7 Hz), 5.13 – 5.07 (1H, m), 3.59 (4H, s), 2.13 – 1.93 (4H, m), 1.67 (3H, s), 1.59 (6H, s), 1.54 (2H, d, *J* = 7.7 Hz), 0.95 (6H, s)

$^{13}C\{^1H\}$ NMR ($CDCl_3$, 151 MHz) δ 134.4, 131.2, 124.7, 119.8, 72.3, 40.0, 27.0, 25.8, 22.0, 17.8, 16.0

^{11}B NMR ($CDCl_3$, 96 MHz) δ 29.7

HRMS (EI): m/z calc. for $C_{15}H_{27}BO_2+H^+$ 250.2099; found 250.2109

MS (APCI): m/z calc. for $C_{15}H_{27}BO_2+H^+$ 251.2; found 251.3

IR (ATR, film) ṽ [cm^{-1}] = 2962, 1477, 1416, 1376, 1296, 1254, 1175, 1070, 1008, 813, 666.

(*E*)-2-(hept-2-en-1-yl)-5,5-dimethyl-1,3,2-dioxaborinane (B5)

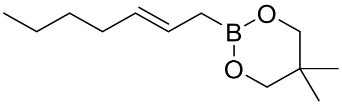

The title compound was prepared according to the general procedure C using the allylic alcohol (*E*)-hept-2-en-1-ol (5 mmol). The crude product was purified using column chromatography (silica gel; pentane:Et_2O 99:1) to afford the allylic boronate (*E*)-2-(hept-2-en-1-yl)-5,5-dimethyl-1,3,2-dioxaborinane (788 mg, 3.75 mmol, 75%) as a colorless oil.

^1H NMR ($CDCl_3$, 600 MHz) δ 5.45 (1H, dt, *J* = 15.0, 7.9 Hz), 5.35 (1H, dt, *J* = 15.0, 6.7 Hz), 3.59 (4H, d, *J* = 2.5 Hz), 1.97 (2H, q, *J* = 7.0 Hz), 1.57 (2H, d, *J* = 7.5 Hz), 1.35 – 1.23 (4H, m), 0.95 (6H, d, *J* = 2.7 Hz), 0.91 – 0.84 (3H, m)

$^{13}C\{^1H\}$ NMR ($CDCl_3$, 151 MHz) δ 130.4, 126.0, 72.3, 32.6, 32.1, 31.8, 22.4, 22.0, 14.1

^{11}B NMR ($CDCl_3$, 96 MHz) δ 29.6

HRMS (ESI): m/z calc. for $C_{12}H_{23}BO_2+H^+$ 210.1785; found 210.1793

MS (APCI): m/z calc. for $C_{12}H_{23}BO_2+H^+$ 211.2; found 211.2

IR (ATR, film) ṽ [cm^{-1}] = 2958, 2929, 1477, 1416, 1376, 1289, 1253, 1176, 964, 813.

5,5-dimethyl-2-((2*E*,6*E*)-3,7,11-trimethyldodeca-2,6,10-trien-1-yl)-1,3,2-dioxaborinane (B6)

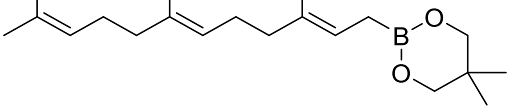

The title compound was prepared according to the general procedure A using the allylic alcohol (*E*,*E*)-farnesol (5 mmol). The crude product was purified using column chromatography (silica gel; pentane:Et_2O 96:4) to afford allylic boronate 5,5-dimethyl-2-((2*E*,6*E*)-3,7,11-trimethyldodeca-2,6,10-trien-1-yl)-1,3,2-dioxaborinane (1.244 g, 3.91 mmol, 78%) as a slightly yellowish oil.

1H NMR (CDCl3, 600 MHz) δ 5.26 (1H, tq, *J* = 7.7, 1.3 Hz), 5.10 (2H, ddddt, *J* = 15.5, 7.1, 5.8, 2.9, 1.4 Hz), 3.59 (4H, s), 2.10 – 1.94 (8H, m), 1.72 – 1.65 (3H, m), 1.63 – 1.57 (9H, m), 1.55 (2H, d, *J* = 7.9 Hz), 0.95 (6H, s)

$^{13}C\{^1H\}$ NMR ($CDCl_3$, 151 MHz) δ 134.7, 134.3, 131.2, 124.5 (d, *J* = 3.1 Hz), 119.7, 72.1, 39.9, 39.7, 31.7, 26.8, 26.8, 25.7, 21.8, 17.7, 16.0, 15.9

^{11}B NMR ($CDCl_3$, 96 MHz) δ 29.3

HRMS (ESI): m/z calc. for $C_{20}H_{35}BO_2+H^+$ 318.2728; found 318.2722

MS (APCI): m/z calc. for $C_{20}H_{35}BO_2+H^+$ 319.3; found 319.4

IR (ATR, film) ṽ [cm^{-1}] = 2927, 1477, 1415, 1376, 1295, 1254, 1175.

4. γ-Selective Suzuki-Miyaura coupling

General procedure for asymmetric γ-selective Suzuki-Miyaura coupling:

An oven dried screw cap vial was charged with the $[Pd(allyl)Cl]_2$ (5 µmol, 2.5 mol%), the ligand (0.02 mmol,10 mol%) and the aryl halide (0.2 mmol) under air. The vial was closed using a screw cap with a PTFE/silicone septa and sealed using parafilm®. The vial was evacuated and refilled with nitrogen using a needle, piercing through the septum. The allylic boronate (0.24 mmol, 1.2 equiv.) dissolved in THF (0.4 mL) was added followed by an aqueous solution (2.5 M, 0.4 mL) of K_3PO_4. The reaction mixture was stirred vigorously over night at room temperature. Afterwards the reaction mixture was diluted using ethyl acetate and transferred into a separatory funnel. The aqueous phase was extracted three times using ethyl acetate. The combined organic phase was washed brine, dried over magnesium sulfate and concentrated *in vacuo*. Purification of the crude reaction product was achieved via column chromatography using the automated chromatography system Biotage Isolera 4.

Compound characterization

(*S*)-5-(3,7-dimethylocta-1,6-dien-3-yl)-1*H*-indole (5a)

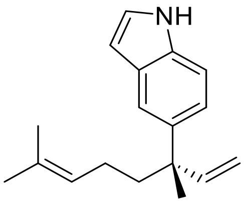

The title compound was prepared from 5-bromoindole (39.2 mg, 0.2 mmol) according to the general procedure for asymmetric γ-selective Suzuki-Miyaura coupling using (*Z*)-2-(3,7-dimethylocta-2,6-dien-1-yl)-5,5-dimethyl-1,3,2-dioxaborinane as allylic boronate. The crude product was purified using column chromatography (silica gel; petrol ether:ethyl acetate 100:0 to 98:2) to afford the title compound (43 mg, 0.17 mmol, 85%) as a colorless oil. The enantiomeric ratio was determined to be 95:5 (*S*:*R*) following HPLC-analysis comparing to an authentic racemic sample (HPLC: Phenomenex Lux 5µm Amylose-1 250 x 4.6 mm, *n*-heptane/^{i}PrOH 85/15, 0.5mL/min).

^{1}H NMR ($CDCl_3$, 600 MHz) δ 8.07 (1H, s), 7.59 (1H, dd, *J* = 1.9, 0.9 Hz), 7.33 (1H, dt, *J* = 8.6, 0.8 Hz), 7.21 – 7.15 (2H, m), 6.52 (1H, ddd, *J* = 3.1, 2.0, 1.0 Hz), 6.11 (1H, dd, *J* = 17.5, 10.7 Hz), 5.13 – 5.05 (3H, m), 1.96 – 1.72 (4H, m), 1.66 (3H, s), 1.52 (3H, s), 1.44 (3H, s)

^{13}C{^{1}H} NMR ($CDCl_3$, 151 MHz) δ 148.1, 139.1, 134.3, 131.3, 127.9, 125.1, 124.3, 121.7, 118.3, 111.2, 110.7, 102.9, 44.3, 41.5, 25.8, 25.6, 23.6, 17.7

HRMS (ESI): m/z calc. for $C_{18}H_{23}N+H^+$ 254.1903; found 254.1903

IR (ATR, film) ṽ [cm^{-1}] = 3413, 2965, 2922, 1633, 1470, 1412, 1373, 1319,01152, 1093, 1065, 1001, 911, 804, 765, 725, 606.

$[\propto]_D^{20} = +8.6$ (c=1, $CHCl_3$)

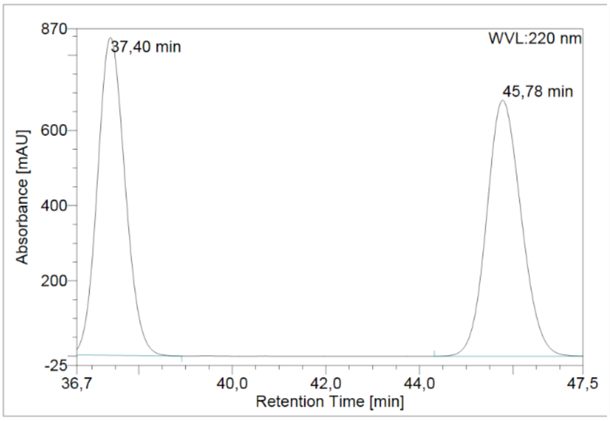

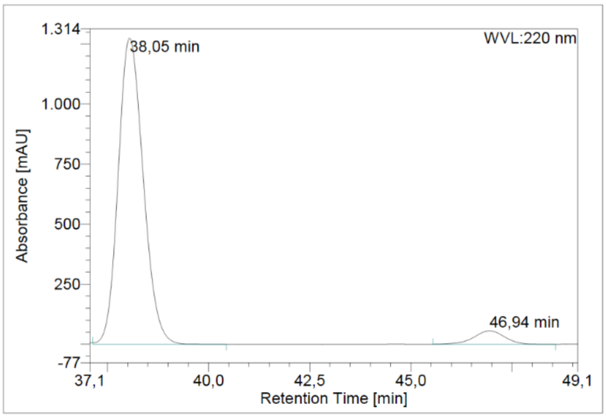

No.	Ret.Time min	Peak Name	Height mAU	Area mAU*min	Rel.Area %	Amount	Type
1	37,40	n.a.	844,960	578,095	49,76	n.a.	BMB*
2	45,78	n.a.	680,670	583,609	50,24	n.a.	BMB
Total:			1525,630	1161,705	100,00	0,000	

No.	Ret.Time min	Peak Name	Height mAU	Area mAU*min	Rel.Area %	Amount	Type
1	38,05	n.a.	1275,286	914,600	94,82	n.a.	MB*
2	46,94	n.a.	56,251	49,945	5,18	n.a.	BMB*
Total:			1331,537	964,545	100,00	0,000	

(*S*)-(3,7-dimethylocta-1,6-dien-3-yl)benzene (5b)

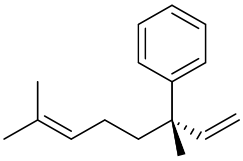

The title compound was prepared from bromobenzene (21 µL, 0.2 mmol) according to the general procedure for asymmetric γ-selective Suzuki-Miyaura coupling using (*Z*)-2-(3,7-dimethylocta-2,6-dien-1-yl)-5,5-dimethyl-1,3,2-dioxaborinane as allylic boronate. The crude product was purified using column chromatography (silica gel; petrol ether:ethyl acetate 100:0) to afford the title compound (37.2 mg, 0.17 mmol, 87%) as a colorless oil. The enantiomeric ratio was determined to be 81:19 (*S*:*R*) following HPLC-analysis comparing to an authentic racemic sample (HPLC: Phenomenex Lux 5µm Cellulose-3 250 x 4.6 mm, water/acetonitrile 10/90, 0.5mL/min).

^1H NMR ($CDCl_3$, 600 MHz) δ 7.38 – 7.29 (4H, m), 7.22 – 7.17 (1H, m), 6.05 (1H, ddd, *J* = 17.5, 10.8, 0.8 Hz), 5.15 – 5.05 (3H, m), 1.92 – 1.68 (4H, m), 1.67 (3H, s), 1.53 (3H, s), 1.40 (3H, s)

$^{13}C\{^1H\}$ NMR ($CDCl_3$, 151 MHz) δ 147.6, 147.1, 131.5, 128.2, 126.7, 125.9, 124.8, 111.9, 44.4, 41.3, 25.8, 25.0, 23.4, 17.7

HRMS (EI): m/z calc. for $C_{16}H_{22}^+$ 214.1721; found 214.1721

IR (ATR, film) ṽ [cm^{-1}] = 2967, 2923, 1634, 1600, 1493, 1444, 1374, 1075, 1030, 912, 833, 763, 698, 535.

$[\propto]_D^{20} = +2.0$ (c = 0.1, CH_3CN)

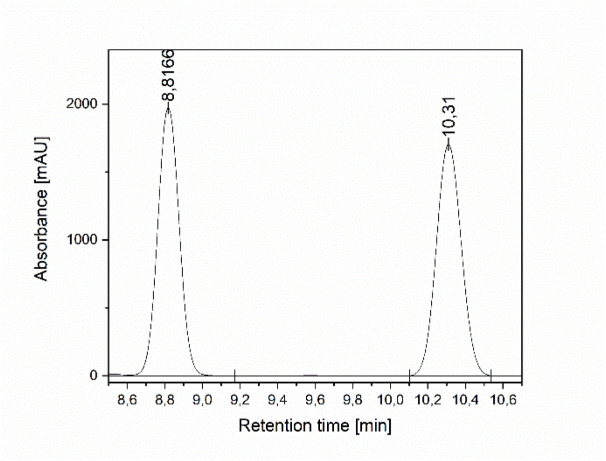

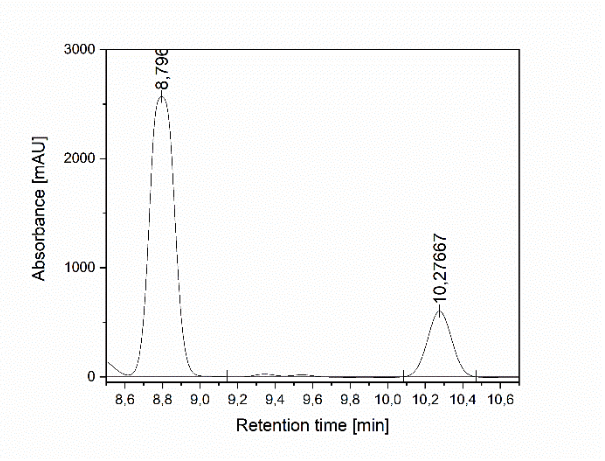

		Retention Time	Area			Height	Relative Area
		min	mAU*min	n.a.		mAU	%
	1	8.817	260.8448	n.a.	M *	1969.663	49.56
	2	10.31	265.4744	n.a.	BMB*	1707.65	50.44
Total:			526.3193	0		3677.313	100

		Retention Time	Area			Height	Relative Area
		min	mAU*min	n.a.		mAU	%
	1	8.797	395.9036	n.a.	M *	2569.463	81.01
	2	10.277	92.8317	n.a.	BMB*	606.858	18.99
Total:			488.7353	0		3176.321	100

(*S*)-1-(3,7-dimethylocta-1,6-dien-3-yl)-4-(trifluoromethyl)benzene (5c)

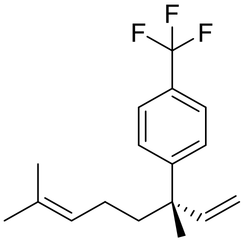

The title compound was prepared from 1-bromo-4-trifluormethylbenzene (28 µL, 0.2 mmol) according to the general procedure for asymmetric γ-selective Suzuki-Miyaura coupling using (*Z*)-2-(3,7-dimethylocta-2,6-dien-1-yl)-5,5-dimethyl-1,3,2-dioxaborinane as allylic boronate. The crude product was purified using column chromatography (silica gel; petrol ether:ethyl acetate 100:0) to afford the title compound (18.1 mg, 0.06 mmol, 32%) as a colorless oil. The enantiomeric ratio was determined to be 38:62 (*S*:*R*) following HPLC-analysis comparing to an authentic racemic sample (HPLC: Phenomenex Lux 5µm Amylose-2 250 x 4.6 mm, water/acetonitrile 40/60, 0.5mL/min).

1H NMR (CDCl3, 600 MHz) δ 7.57 – 7.54 (2H, m), 7.45 – 7.42 (2H, m), 6.01 (1H, dd, *J* = 17.5, 10.7 Hz), 5.15 (1H, dd, *J* = 10.8, 1.2 Hz), 5.09 – 5.05 (2H, m), 1.88 – 1.68 (4H, m), 1.66 (3H, s), 1.51 (3H, s), 1.40 (3H, s)

$^{13}C\{^{1}H\}$ NMR ($CDCl_3$, 151 MHz) δ 151.8, 146.1, 131.8, 127.2, 125.1 (q, *J* = 3.8 Hz), 124.4, 112.8, 44.7, 41.2, 25.8, 25.0, 23.4, 17.7

^{19}F NMR ($CDCl_3$, 282 MHz) δ -62.3

HRMS (EI): m/z calc. for $C_{17}H_{21}F_3^+$ 282.1595; found 282.1594

IR (ATR, film) ṽ [cm^{-1}] = 2926, 1617, 1413, 1326, 1165, 1125, 1079, 1066, 1015, 918, 840.

$[\propto]_{D}^{20} = +3.0$ (c = 0.1, CH_3CN)

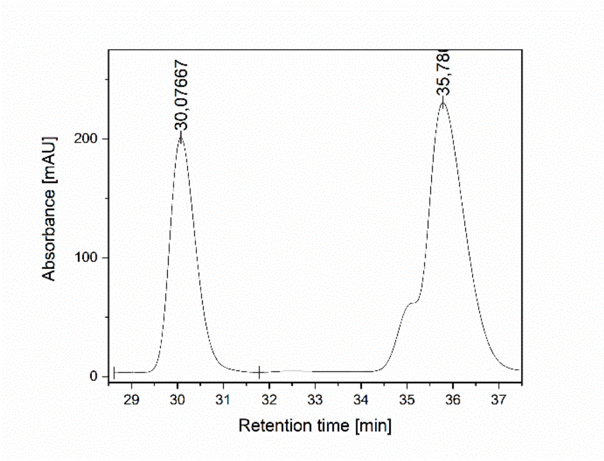

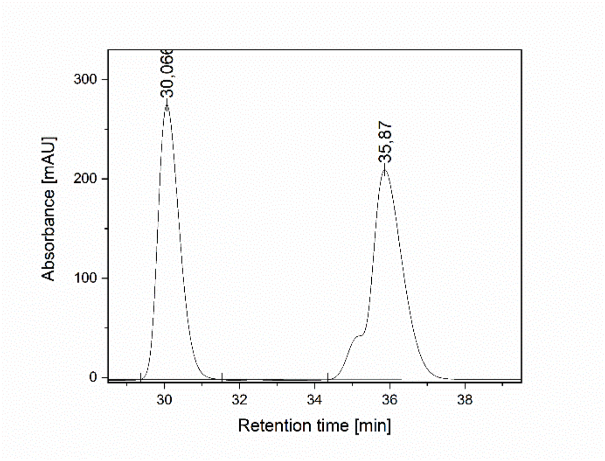

		Retention Time	Area			Height	Relative Area
		min	mAU*min	n.a.		mAU	%
	1	30.067	185.3388	n.a.	BMB*	277.483	49.07
	2	35.87	192.3346	n.a.	MB*	211.422	50.93
Total:			377.6735	0		488.906	100

		Retention Time	Area			Height	Relative Area
		min	mAU*min	n.a.		mAU	%
	1	30.077	129.9778	n.a.	BMB*	197.736	38.22
	2	35.787	210.0759	n.a.	MB*	226.273	61.78
Total:			340.0537	0		424.009	100

(*S*)-1-(3,7-dimethylocta-1,6-dien-3-yl)-4-methoxybenzene (5d)

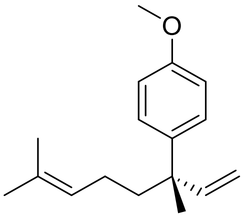

Both enantiomers of the title compound were prepared from 4-bromoanisole (25 µL, 0.2 mmol) according to the general procedure for asymmetric γ-selective Suzuki-Miyaura coupling using (*Z*)-2-(3,7-dimethylocta-2,6-dien-1-yl)-5,5-dimethyl-1,3,2-dioxaborinane and (*E*)-2-(3,7-dimethylocta-2,6-dien-1-yl)-5,5-dimethyl-1,3,2-dioxaborinane as allylic boronate. The crude product was purified using column chromatography (silica gel; petrol ether:ethyl acetate 100:0 to 98:2) to afford the title compound (37.3 mg, 0.15 mmol, 75% (0.2 mmol scale); 195.3 mg 0.80 mmol, 80% (1 mmol scale); 542.1 mg, 2.22 mmol, 74% (3 mmol scale)) as a colorless oil. The analytical data is in accordance with the published literature.[5] The enatiomeric ratios were determined to be 86:14 (*S*:*R*) from the *Z*-boronate and 24:76 (*S*:*R*) from the *E*-boronate following HPLC analysis and peak deconvolution in comparison to a racemic sample (HPLC: Chiralcel ODH 250 x 4.6 mm, n-heptane/^{i}PrOH 99.9/0.1, 0.3mL/min, 15 °C).

^{1}H NMR ($CDCl_3$, 600 MHz) δ 7.25 – 7.22 (2H, m), 6.86 – 6.82 (2H, m), 6.01 (1H, dd, *J* = 17.5, 10.7 Hz), 5.11 – 5.06 (2H, m), 5.02 (1H, dd, *J* = 17.5, 1.3 Hz), 3.79 (3H, s), 1.90 – 1.67 (4H, m), 1.66 (3H, s), 1.52 (3H, s), 1.36 (3H, s).

$^{13}C\{^1H\}$ NMR ($CDCl_3$, 151 MHz) δ 157.7, 147.4, 139.7, 131.4, 127.7, 124.9, 113.5, 111.6, 55.4, 43.8, 41.3, 25.8, 25.2, 23.5, 17.7.

MS (EI): m/z calc. for $C_{17}H_{24}O^+$ 244.18; found 244.21

IR (ATR, film) ṽ [cm^{-1}] = 2966, 2926, 1609, 1510, 1463, 1372, 1293, 1248, 1181, 1111, 1037, 912, 827, 648, 543.

$[\propto]_D^{20} = +2.6$ (c=1, $CHCl_3$) for *R* enantiomer

$[\propto]_D^{20} = -2.3$ (c=1, $CHCl_3$) for *S* enantiomer

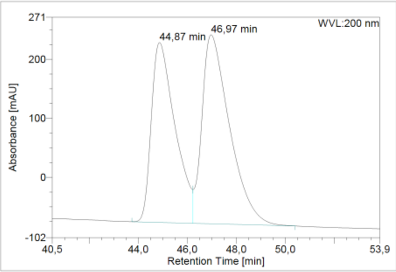

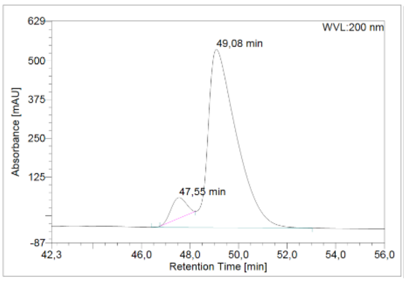

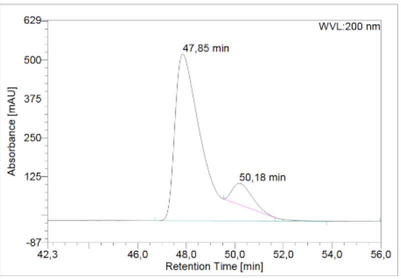

No.	Ret.Time min	Peak Name	Height mAU	Area mAU*min	Rel.Area %	Amount	Type
1	11,07	n.a.	9,470	3,383	0,44	n.a.	BMB
2	44,87	n.a.	304,918	342,729	44,13	n.a.	BM
3	46,97	n.a.	320,478	419,537	54,01	n.a.	MB
4	55,30	n.a.	8,944	11,061	1,42	n.a.	BMB
Total:			643,810	776,710	100,00	0,000	

No.	Ret.Time min	Peak Name	Height mAU	Area mAU*min	Rel.Area %	Amount	Type
1	47,55	n.a.	66,203	47,285	5,43	n.a.	Ru
2	49,08	n.a.	574,773	822,940	94,57	n.a.	BMB
Total:			640,976	870,225	100,00	0,000	

No.	Ret.Time min	Peak Name	Height mAU	Area mAU*min	Rel.Area %	Amount	Type
1	11,10	n.a.	10,509	3,829	0,47	n.a.	BMB
2	47,85	n.a.	536,857	731,068	89,13	n.a.	BMB
3	50,18	n.a.	68,486	65,183	7,95	n.a.	Rd
4	57,34	n.a.	15,857	20,141	2,46	n.a.	BMB
Total:			631,710	820,221	100,00	0,000	

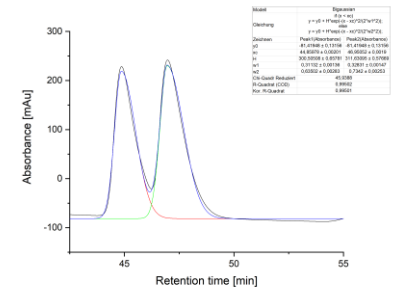

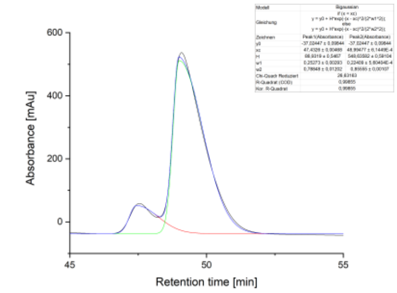

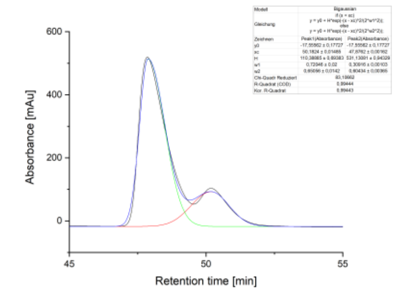

	Area mAU*min	n.a.	Height mAU	Relative Area %
1	356.38485		300.50503	46.20
2	414.94965		311.63077	53.80
Total:	771.33450	0		

	Area mAU*min	n.a.	Height mAU	Relative Area %
1	116.0383		88.93184	13.52
2	742.27449		548.62694	86.48
Total:	858.31279	0		

	Area mAU*min	n.a.	Height mAU	Relative Area %
1	608.0418		531.13071	76.22
2	189.66764		110.38884	23.78
Total:	797.70944			

(*S*)-1-(3,7-dimethylocta-1,6-dien-3-yl)-4-nitrobenzene (5e)

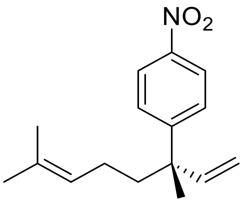

The title compound was prepared from 1-bromo-4-nitrobenzene (40.4 mg, 0.2 mmol) according to the general procedure for asymmetric γ-selective Suzuki-Miyaura coupling using (*Z*)-2-(3,7-dimethylocta-2,6-dien-1-yl)-5,5-dimethyl-1,3,2-dioxaborinane as allylic boronate. The crude product was purified using column chromatography (silica gel; petrol ether:ethyl acetate 99:1 to 96:4) to afford the title compound (17.9 mg, 0.064 mmol, 32%) as a yellowish oil. The enantiomeric ratio was determined to be 46:54 (*S*:*R*) following HPLC-analysis comparing to an authentic racemic sample (HPLC: Phenomenex Lux 5µm Cellulose-3 250 x 4.6 mm, water/acetonitrile 40/60, 0.5mL/min).

^{1}H NMR ($CDCl_3$, 600 MHz) δ 8.17 – 8.13 (2H, m), 7.52 – 7.44 (2H, m), 6.01 (1H, dd, *J* = 17.5, 10.7 Hz), 5.19 (1H, dd, *J* = 10.7, 1.0 Hz), 5.09 (1H, dd, *J* = 17.5, 1.0 Hz), 5.07 – 5.03 (1H, m), 1.89 – 1.66 (5H, m), 1.65 (3H, s), 1.50 (3H, s), 1.42 (3H, s)

$^{13}C\{^1H\}$ NMR ($CDCl_3$, 151 MHz) δ 155.5, 145.5, 127.8, 124.1, 123.4, 113.4, 45.1, 41.2, 25.9, 25.0, 23.3, 17.7

HRMS (ESI): m/z calc. for $C_{16}H_{21}NNaO_2+H^+$ 282.1464; found 282.1462

IR (ATR, film) ṽ [cm^{-1}] = 2968, 2924, 1596, 1518, 1454, 1412, 1344, 1110, 1073, 1013, 919, 853, 757, 726, 702.

$[\propto]_D^{20} = 0.0$ (c = 0.1, CH_3CN)

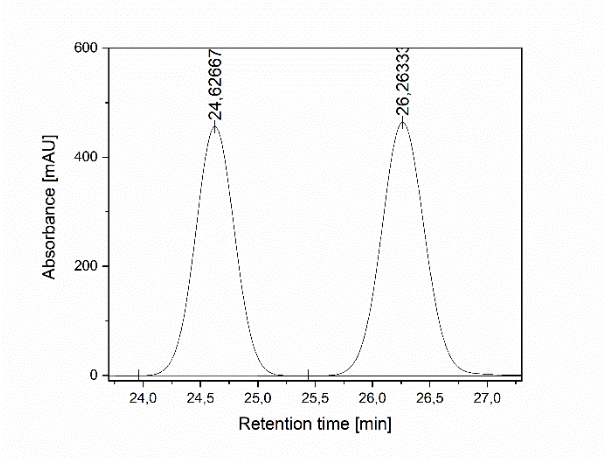

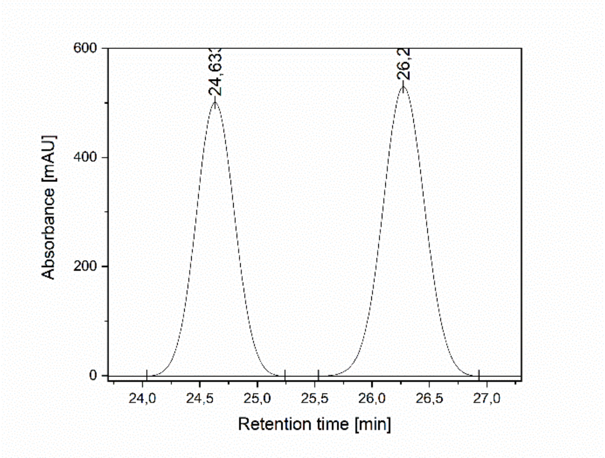

		Retention Time	Area			Height	Relative Area
		min	mAU*min	n.a.		mAU	%
	1	24.627	185.3688	n.a.	M *	456.845	47.15
	2	26.263	207.8057	n.a.	M *	464.933	52.85
Total:			393.1745	0		921.777	100

		Retention Time	Area			Height	Relative Area
		min	mAU*min	n.a.		mAU	%
	1	24.633	204.2876	n.a.	BM *	502.829	46.45
	2	26.273	235.5059	n.a.	MB*	531.721	53.55
Total:			439.7935	0		1034.549	100

(*S*)-*N*-(4-(3,7-dimethylocta-1,6-dien-3-yl)phenyl)acetamide (5f)

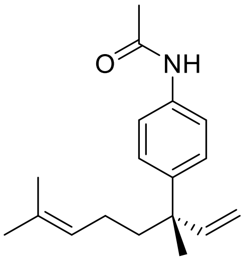

Both enantiomers of the title compound were prepared from 4-bromoacetanilid (42.8 mg, 0.2 mmol) according to the general procedure for asymmetric γ-selective Suzuki-Miyaura coupling using (*Z*)-2-(3,7-dimethylocta-2,6-dien-1-yl)-5,5-dimethyl-1,3,2-dioxaborinane and (*E*)-2-(3,7-dimethylocta-2,6-dien-1-yl)-5,5-dimethyl-1,3,2-dioxaborinane as allylic boronate. The crude product was purified using column chromatography (silica gel; petrol ether:ethyl acetate 85:15 to 70:30) to afford the title compound (35.2 mg, 0.13 mmol, 65%) as a colorless oil. The analytical data is in accordance with the published literature.[6] The enantiomeric ratio was determined to be 80:20 (*S*:*R*) from the *Z*-boronate and 24:76 (*S*:*R*) from the *E*-boronate following HPLC-analysis comparing to an authentic racemic sample (HPLC: Phenomenex Lux 5µm Amylose-1 250 x 4.6 mm, n-heptane/^{i}PrOH 75/25, 0.3mL/min).

^{1}H NMR ($CDCl_3$, 600 MHz) δ 7.30 – 7.27 (2H, m), 7.13 – 7.10 (2H, m), 5.86 (1H, dd, *J* = 17.5, 10.7 Hz), 4.96 – 4.91 (2H, m), 4.89 (1H, dd, *J* = 17.5, 1.3 Hz), 2.02 (3H, s), 1.75 – 1.53 (4H, m), 1.51 (3H, s), 1.37 (3H, s), 1.21 (3H, s)

^{13}C{^{1}H} NMR ($CDCl_3$, 151 MHz) δ 168.5, 147.0, 143.6, 135.7, 131.5, 127.3, 124.7, 119.8, 111.9, 44.1, 41.2, 25.8, 25.0, 24.6, 23.4, 17.7

HRMS (ESI): m/z calc. for $C_{18}H_{25}NO+H^+$ 272.2009; found 272.2009

IR (ATR, film) ṽ [cm^{-1}] = 2967, 2921, 1512, 1451, 1374, 1019, 911, 814, 725, 534.

$[\propto]_D^{20} = +7.6$ (c=1, $CHCl_3$) for *R* enantiomer

$[\propto]_D^{20} = -1.9$ (c=1, $CHCl_3$) for *S* enantiomer

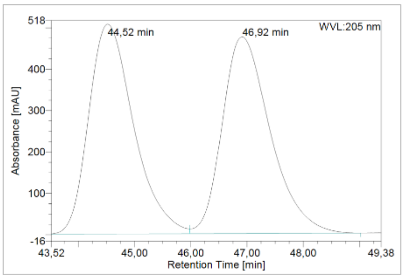

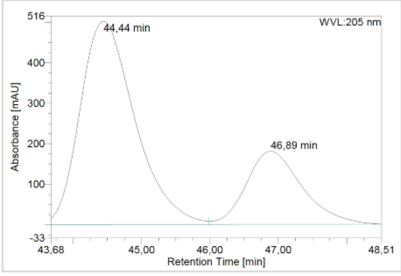

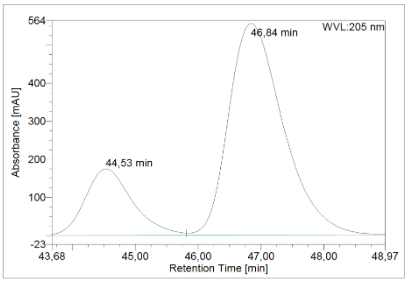

No.	Ret.Time min	Peak Name	Height mAU	Area mAU*min	Rel.Area %	Amount	Type
1	44,52	n.a.	507,109	469,993	49,99	n.a.	BM
2	46,92	n.a.	475,326	470,155	50,01	n.a.	MB
Total:			982,436	940,148	100,00	0,000	

No.	Ret.Time min	Peak Name	Height mAU	Area mAU*min	Rel.Area %	Amount	Type
1	44,44	n.a.	501,819	466,276	70,78	n.a.	BM
2	46,89	n.a.	180,594	170,851	25,94	n.a.	M
3	49,40	n.a.	25,597	21,606	3,28	n.a.	MB
Total:			708,010	658,732	100,00	0,000	

No.	Ret.Time min	Peak Name	Height mAU	Area mAU*min	Rel.Area %	Amount	Type
1	44,53	n.a.	174,720	153,174	21,46	n.a.	BM *
2	46,84	n.a.	555,714	560,709	78,54	n.a.	MB*
Total:			730,434	713,883	100,00	0,000	

(*S*)-1-(3,7-dimethylocta-1,6-dien-3-yl)-4-methylbenzene (5g)

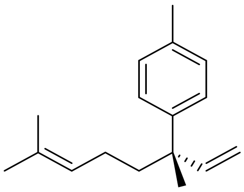

The title compound was prepared from 4-bromotoluene (34.2 mg, 0.2 mmol) according to the general procedure for asymmetric γ-selective Suzuki-Miyaura coupling using (*Z*)-2-(3,7-dimethylocta-2,6-dien-1-yl)-5,5-dimethyl-1,3,2-dioxaborinane as allylic boronate. The crude product was purified using column chromatography (silica gel; petrol ether 100:0) to afford the title compound (40.5 mg, 0.18 mmol, 88%) as a colorless oil. The enantiomeric ratio was determined to be 86:14 (*S*:*R*) following HPLC-analysis comparing to an authentic racemic sample (HPLC: Phenomenex Lux 5µm Cellulose-3 250 x 4.6 mm, water/acetonitrile 10/90, 0.5mL/min).

^{1}H NMR ($CDCl_3$, 600 MHz) δ 7.23 – 7.20 (2H, m), 7.14 – 7.11 (2H, m), 6.03 (1H, dd, *J* = 17.5, 10.7 Hz), 5.13 – 5.08 (2H, m), 5.05 (1H, dd, *J* = 17.5, 1.4 Hz), 2.33 (3H, s), 1.91 – 1.68 (4H, m), 1.67 (3H, s), 1.53 (3H, s), 1.37 (3H, s)

^{13}C{^{1}H} NMR ($CDCl_3$, 151 MHz) δ 147.2, 144.6, 135.3, 131.4, 128.9, 126.6, 124.9, 111.7, 44.1, 41.2, 25.8, 25.1, 23.5, 21.0, 17.7

HRMS (EI): m/z calc. for $C_{17}H_{24}H^+$ 228.1878; found 228.1877

IR (ATR, film) ṽ [cm^{-1}] = 3299, 2967, 2924, 1663, 1601, 1537, 1513, 1443, 1405, 1371, 1320, 1264, 1018, 913, 830, 754, 543.

$[\propto]_D^{20} = +3.0$ (c=1, $CHCl_3$)

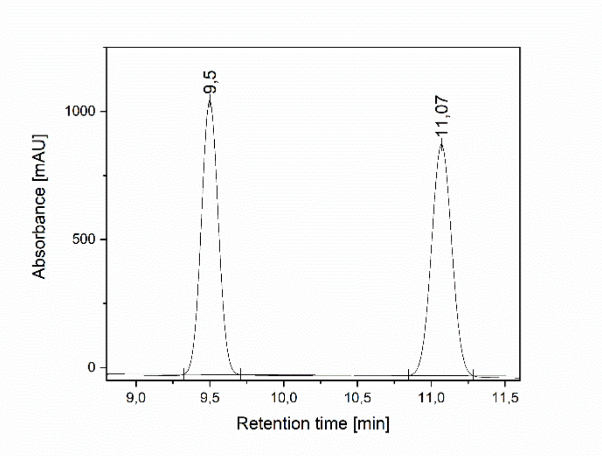

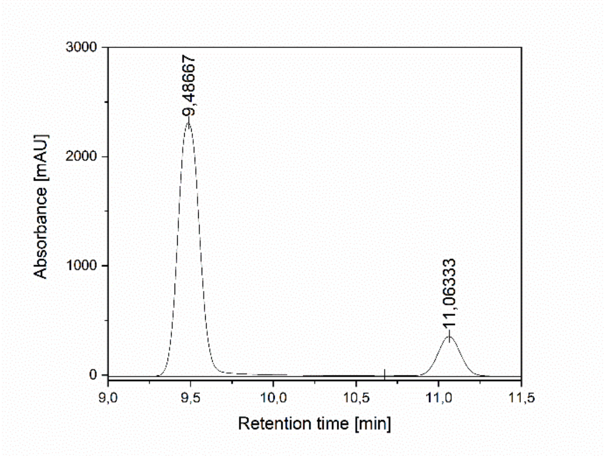

	Retention Time	Area			Height	Relative Area
	min	mAU*min	n.a.		mAU	%
1	9.487	353.1507	n.a.	BMB*	2317.1	85.92
2	11.063	57.8635	n.a.	BMB*	360.876	14.08
Total:		411.0142	0		2677.976	100

	Retention Time	Area			Height	Relative Area
	min	mAU*min	n.a.		mAU	%
1	9.487	353.1507	n.a.	BMB*	2317.1	85.92
2	11.063	57.8635	n.a.	BMB*	360.876	14.08
Total:		411.0142	0		2677.976	100

(*S*)-1-(3,7-dimethylocta-1,6-dien-3-yl)-3-methylbenzene (5h)

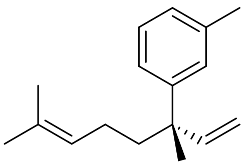

The title compound was prepared from 3-bromotoluene (24 µL, 0.2 mmol) according to the general procedure for asymmetric γ-selective Suzuki-Miyaura coupling using (*Z*)-2-(3,7-dimethylocta-2,6-dien-1-yl)-5,5-dimethyl-1,3,2-dioxaborinane as allylic boronate. The crude product was purified using column chromatography (silica gel; petrol ether:ethyl acetate 100:0) to afford the title compound (29.6 mg, 0.13 mmol, 65%) as a colorless oil. The enantiomeric ratio was determined to be 88:12 (*S*:*R*) following HPLC-analysis comparing to an authentic racemic sample (HPLC: Phenomenex Lux 5µm Cellulose-3 250 x 4.6 mm, water/acetonitrile 30/70, 0.5mL/min).

^1H NMR ($CDCl_3$, 600 MHz) δ 7.21 (1H, dd, *J* = 8.6, 7.3 Hz), 7.18 – 7.11 (2H, m), 7.05 – 6.99 (1H, m), 6.05 (1H, dd, *J* = 17.5, 10.7 Hz), 5.15 – 5.04 (3H, m), 2.36 (3H, s), 1.92 – 1.70 (4H, m), 1.68 (3H, s), 1.55 (3H, s), 1.39 (3H, s)

$^{13}C\{^1H\}$ NMR ($CDCl_3$, 151 MHz) δ 147.6, 147.1, 137.6, 131.4, 128.1, 127.5, 126.6, 124.9, 123.8, 111.7, 44.3, 41.2, 25.8, 25.0, 23.5, 21.8, 17.7

HRMS (EI): m/z calc. for $C_{17}H_{24}H^+$ 228.1878; found 228.1877

IR (ATR, film) ṽ [cm^{-1}] = 2966, 2920, 1605, 1452, 1374, 1000, 911, 783, 705.

$[\propto]_D^{20} = -64.0$ (c = 0.1, CH_3CN)

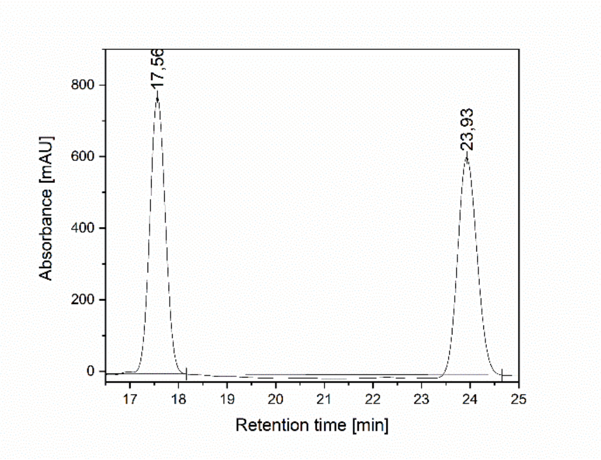

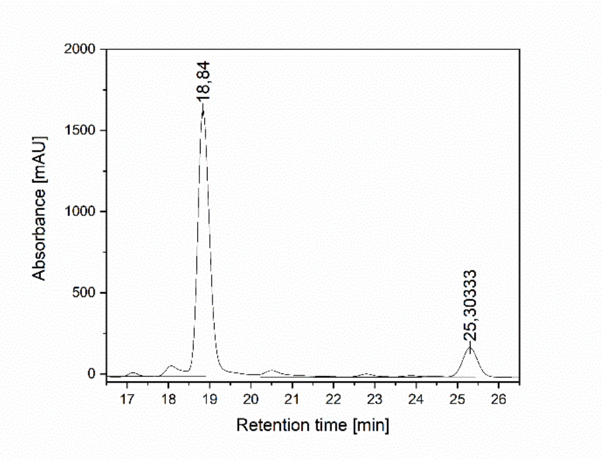

		Retention Time	Area			Height	Relative Area
		min	mAU*min	n.a.		mAU	%
1		17.567	289.1313	n.a.	MB*	776.319	50.11
2		23.93	287.823	n.a.	BMB*	614.814	49.89
Total:			576.9544	0		1391.133	100

		Retention Time	Area			Height	Relative Area
		min	mAU*min	n.a.		mAU	%
1		18.84	551.0054	n.a.	M *	1642.324	87.69
2		25.303	77.3154	n.a.	BMB*	181.055	12.31
Total:			628.3209	0		1823.379	100

(*S*)-1-(3,7-dimethylocta-1,6-dien-3-yl)-2-methylbenzene (5i)

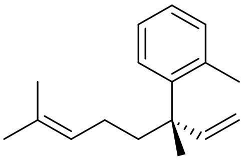

The title compound was prepared from 2-iodotoluene (31 µL, 0.2 mmol) according to the general procedure for asymmetric γ-selective Suzuki-Miyaura coupling using (*Z*)-2-(3,7-dimethylocta-2,6-dien-1-yl)-5,5-dimethyl-1,3,2-dioxaborinane as allylic boronate. The crude product was purified using column chromatography (silica gel; petrol ether:ethyl acetate 100:0) to afford the title compound (21.9 mg, 0.09 mmol, 48%) as a colorless oil. The enantiomeric ratio was determined to be 66:34 (*S*:*R*) following HPLC-analysis comparing to an authentic racemic sample (HPLC: Phenomenex Lux 5µm Cellulose-3 250 x 4.6 mm, water/acetonitrile 40/60, 0.5mL/min).

^1H NMR ($CDCl_3$, 600 MHz) δ 7.32 (1H, dd, *J* = 7.6, 1.7 Hz), 7.17 – 7.08 (3H, m), 6.07 (1H, dd, *J* = 17.6, 10.7 Hz), 5.08 (1H, tdq, *J* = 7.1, 2.9, 1.5 Hz), 5.04 (1H, dd, *J* = 10.7, 1.3 Hz), 4.96 – 4.90 (1H, m), 2.38 (3H, s), 2.04 – 1.97 (1H, m), 1.87 (1H, tt, *J* = 12.5, 6.1 Hz), 1.72 (1H, ddd, *J* = 13.2, 12.1, 4.4 Hz), 1.67 – 1.64 (3H, m), 1.64 – 1.57 (1H, m), 1.50 (3H, s), 1.44 (3H, s)

$^{13}C\{^1H\}$ NMR ($CDCl_3$, 151 MHz) δ 147.8, 144.3, 137.3, 132.5, 131.4, 127.5, 126.3, 125.6, 124.9, 111.5, 45.3, 39.6, 27.1, 25.8, 23.5, 22.8, 17.7

HRMS (EI): m/z calc. for $C_{17}H_{24}H^+$ 228. 1878; found 228.1877

IR (ATR, film) ṽ [cm^{-1}] = 2966, 2915, 1449, 1376, 1053, 910, 758, 741, 728.

$[\propto]_D^{20} = 0.0$ (c = 0.1, CH_3CN)

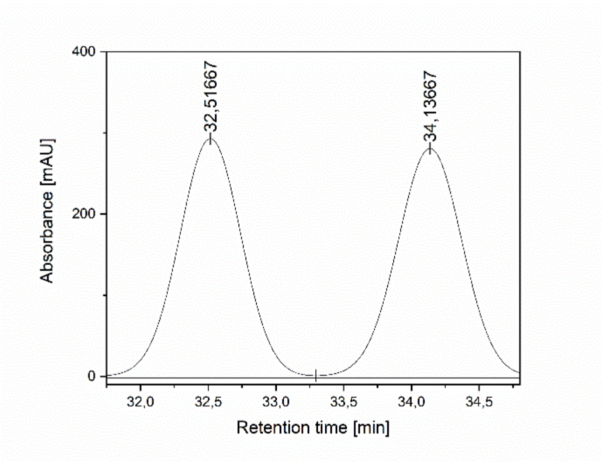

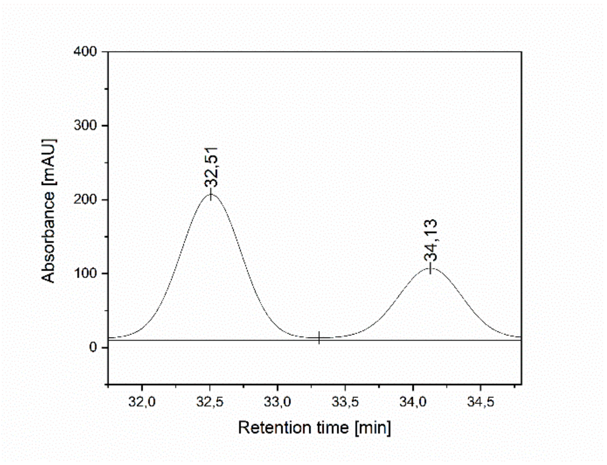

		Retention Time	Area			Height	Relative Area
		min	mAU*min	n.a.		mAU	%
	1	32.517	162.1246	n.a.	BM *	292.918	50.07
	2	34.137	161.6649	n.a.	M *	280.478	49.93
Total:			323.7895	0		573.397	100

		Retention Time	Area			Height	Relative Area
		min	mAU*min	n.a.		mAU	%
	1	32.51	106.6056	n.a.	BM *	194.499	66.13
	2	34.13	54.5898	n.a.	M *	94.493	33.87
Total:			161.1954	0		288.993	100

(*S*)-2-(3,7-dimethylocta-1,6-dien-3-yl)naphthalene (5j)

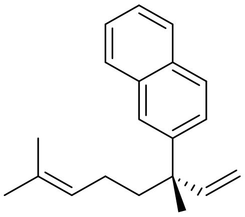

The title compound was prepared from 2-bromonaphthalene (41.4 mg, 0.2 mmol) according to the general procedure for asymmetric γ-selective Suzuki-Miyaura coupling using (*Z*)-2-(3,7-dimethylocta-2,6-dien-1-yl)-5,5-dimethyl-1,3,2-dioxaborinane as allylic boronate. The crude product was purified using column chromatography (silica gel; petrol ether:ethyl acetate 100:0) to afford the title compound (41.2 mg, 0.16 mmol, 78%) as a colorless oil. The enantiomeric ratio was determined to be 18:82 (*S*:*R*) following HPLC-analysis comparing to an authentic racemic sample (HPLC: Phenomenex Lux 5µm Cellulose-3 250 x 4.6 mm, water/acetonitrile 45/55, 0.5mL/min).

^{1}H NMR ($CDCl_3$, 600 MHz) δ 7.83 – 7.77 (3H, m), 7.76 – 7.71 (1H, m), 7.48 (1H, dd, *J* = 8.6, 1.9 Hz), 7.47 – 7.42 (2H, m), 6.12 (1H, dd, *J* = 17.5, 10.7 Hz), 5.16 (1H, dd, *J* = 10.7, 1.4 Hz), 5.14 – 5.07 (2H, m), 1.99 – 1.73 (4H, m), 1.69 – 1.64 (3H, m), 1.51 (3H, s), 1.49 (3H, s)

^{13}C{^{1}H} NMR ($CDCl_3$, 151 MHz) δ 147.0, 145.0, 133.5, 132.0, 131.6, 128.1, 127.7, 127.5, 125.9, 125.9, 125.5, 124.9, 124.8, 112.2, 44.6, 41.1, 25.8, 25.1, 23.5, 17.7

HRMS (EI): m/z calc. for $C_{20}H_{24}^{+}$ 264.1878; found 264.1874

IR (ATR, film) $\tilde{\nu}$ [cm^{-1}] = 3053, 2967, 2919, 1733, 1591, 1502, 1375, 912, 810, 744, 474.

$[\propto]_D^{20} = +40.0$ (c = 0.1, CH_3CN)

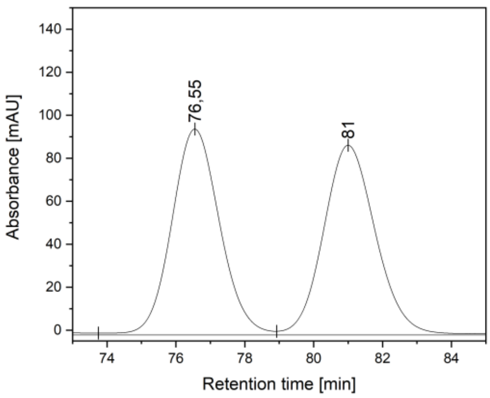

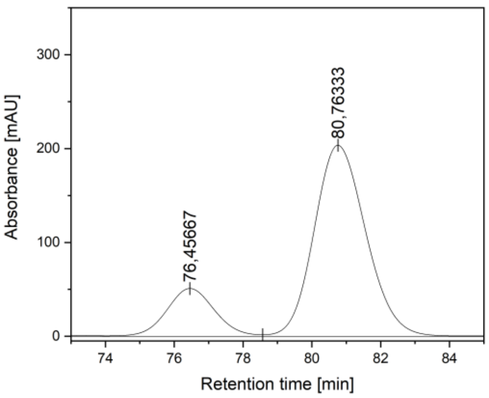

		Retention time	Area			Height	Relative Area
		min	mAU*min	n.a.		mAU	%
	1	76.55	150.7925	n.a.	BM *	94.952	50
	2	81	150.7959	n.a.	MB*	87.555	50
Total:			301.5883	0		182.507	100

		Retention time	Area			Height	Relative Area
		min	mAU*min	n.a.		mAU	%
	1	76.457	80.4938	n.a.	BM *	50.441	18.58
	2	80.763	352.7052	n.a.	MB*	203.175	81.42
Total:			433.199	0		253.617	100

(*S*)-6-(3,7-dimethylocta-1,6-dien-3-yl)-1H-indole (5k)

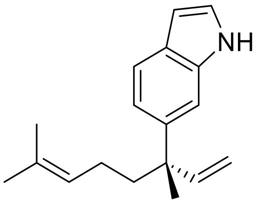

The title compound was prepared from 6-bromoindole (39.2 mg, 0.2 mmol) according to the general procedure for asymmetric γ-selective Suzuki-Miyaura coupling using (*Z*)-2-(3,7-dimethylocta-2,6-dien-1-yl)-5,5-dimethyl-1,3,2-dioxaborinane as allylic boronate. The crude product was purified using column chromatography (silica gel; petrol ether:ethyl acetate 100:0 to 98:2) to afford the title compound (44.6 mg, 0.18 mmol, 88%) as a colorless oil. The enantiomeric ratio was determined to be 93:7 (*S*:*R*) following HPLC-analysis comparing to an authentic racemic sample (HPLC: Phenomenex Lux 5µm Cellulose-3 250 x 4.6 mm, water/acetonitrile 10/90, 0.5mL/min).

^{1}H NMR ($CDCl_3$, 600 MHz) δ 8.06 (1H, s), 7.57 (1H, dd, *J* = 8.4, 0.8 Hz), 7.35 (1H, dt, *J* = 1.7, 0.8 Hz), 7.17 (1H, dd, *J* = 3.2, 2.3 Hz), 7.13 (1H, dd, *J* = 8.4, 1.6 Hz), 6.51 (1H, ddd, *J* = 3.1, 2.0, 1.0 Hz), 6.11 (1H, dd, *J* = 17.5, 10.8 Hz), 5.13 – 5.05 (3H, m), 1.95 – 1.73 (4H, m), 1.66 (3H, s), 1.52 (3H, s), 1.44 (3H, s)

^{13}C{^{1}H} NMR ($CDCl_3$, 151 MHz) δ 147.7, 141.8, 136.2, 131.4, 125.9, 125.0, 124.1, 120.3, 119.4, 111.4, 109.0, 102.4, 44.5, 41.5, 25.8, 25.5, 23.6, 17.7

HRMS (ESI): m/z calc. for $C_{18}H_{23}N+H^+$ 254.1903; found 254.1905

IR (ATR, film) ṽ [cm^{-1}] = 3414, 2966, 2921, 1624, 1452, 1374, 1345, 1319, 1093, 1000, 912, 811, 766, 721, 651, 609.

$[\propto]_D^{20} = +10.0$ (c = 0.1, CH_3CN)

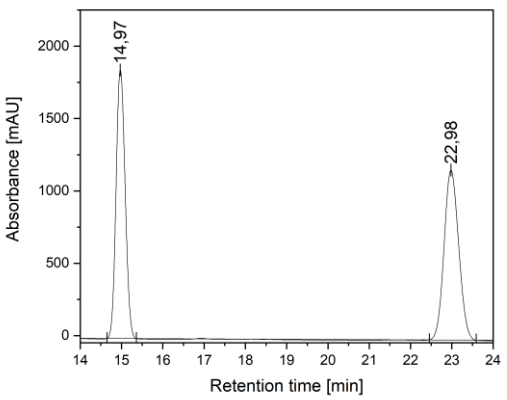

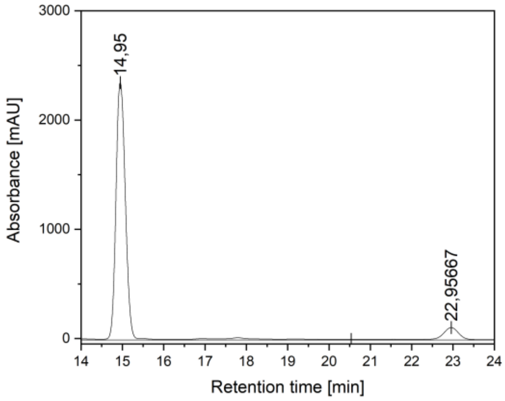

		Retention time	Area			Height	Relative Area
		min	mAU*min	n.a.		mAU	%
1		14.97	474.8589	n.a.	BM *	1859.909	49.5
2		22.98	484.4373	n.a.	BM *	1177.571	50.5
Total:			959.2962	0		3037.481	100

		Retention time	Area			Height	Relative Area
		min	mAU*min	n.a.		mAU	%
1		14.95	611.0299	n.a.	BM *	2348.841	93.18
2		22.957	44.748	n.a.	BMB*	109.592	6.82
Total:			655.7778	0		2458.433	100

(*S*)-5-(3,7-dimethylocta-1,6-dien-3-yl)benzofuran (5l)

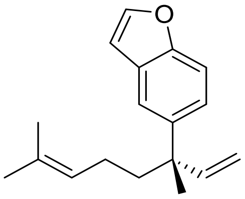

The title compound was prepared from 5-bromobenzofuran (25 µL, 0.2 mmol) according to the general procedure for asymmetric γ-selective Suzuki-Miyaura coupling using (*Z*)-2-(3,7-dimethylocta-2,6-dien-1-yl)-5,5-dimethyl-1,3,2-dioxaborinane as allylic boronate. The crude product was purified using column chromatography (silica gel; petrol ether:ethyl acetate 100:0) to afford the title compound (42.5 mg, 0.17 mmol, 83%) as a colorless oil. The enantiomeric ratio was determined to be 88:12 (*S*:*R*) following HPLC-analysis comparing to an authentic racemic sample (HPLC: Phenomenex Lux 5µm Cellulose-3 250 x 4.6 mm, water/acetonitrile 45/55, 0.5mL/min).

^{1}H NMR ($CDCl_3$, 600 MHz) δ 7.59 (1H, d, *J* = 2.1 Hz), 7.54 (1H, d, *J* = 1.9 Hz), 7.45 – 7.40 (1H, m), 7.28 (1H, dd, *J* = 8.7, 2.0 Hz), 6.73 (1H, dd, *J* = 2.2, 1.0 Hz), 6.08 (1H, ddd, *J* = 17.5, 10.8, 1.1 Hz), 5.14 – 5.05 (3H, m), 1.93 – 1.71 (4H, m), 1.66 (3H, s), 1.51 (3H, s), 1.44 (3H, s)

^{13}C{^{1}H} NMR ($CDCl_3$, 151 MHz) δ 153.5, 147.5, 145.1, 142.2, 131.5, 127.3, 124.8, 123.6, 119.0, 111.7, 110.9, 106.9, 44.4, 41.6, 25.8, 25.6, 23.5, 17.7

HRMS (ESI): m/z calc. for $C_{18}H_{22}O+H^+$ 255.1743; found 255.1738

IR (ATR, film) ṽ [cm^{-1}] = 2967, 2922, 1634, 1466, 1373, 1266, 1133, 1111, 1031, 913, 884, 810, 766, 736, 682.

$[\alpha]_D^{20} = -12.0$ (c = 0.1, CH_3CN)

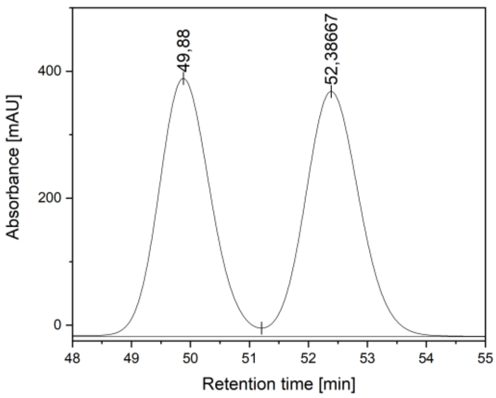

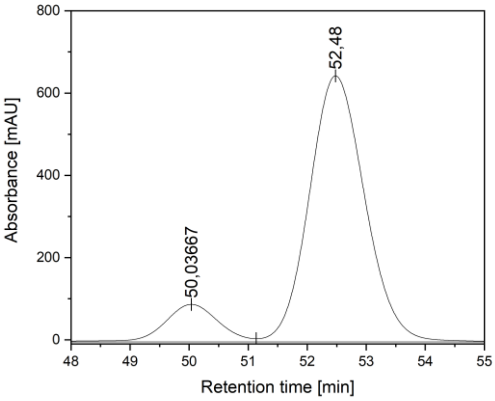

		Retention time	Area			Height	Relative Area
		min	mAU*min	n.a.		mAU	%
1		49.88	414.3383	n.a.	BM *	405.571	49.94
2		52.387	415.3986	n.a.	MB*	385.447	50.06
Total:			829.7369	0		791.018	100

		Retention time	Area			Height	Relative Area
		min	mAU*min	n.a.		mAU	%
1		50.037	91.6671	n.a.	BM *	89.618	11.57
2		52.48	700.5735	n.a.	MB*	645.41	88.43
Total:			792.2407	0		735.028	100

(*S*)-5-(3,7-dimethylocta-1,6-dien-3-yl)benzothiophene (5m)

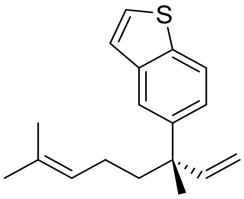

The title compound was prepared from 5-bromobenzothiophen (42.6 mg, 0.2 mmol) according to the general procedure for asymmetric γ-selective Suzuki-Miyaura coupling using (*Z*)-2-(3,7-dimethylocta-2,6-dien-1-yl)-5,5-dimethyl-1,3,2-dioxaborinane as allylic boronate. The crude product was purified using column chromatography (silica gel; petrol ether:ethyl acetate 100:0) to afford the title compound (40.8 mg, 0.15 mmol, 75%) as a colorless oil. The enantiomeric ratio was determined to be 84:16 (*S*:*R*) following HPLC-analysis comparing to an authentic racemic sample (HPLC: Phenomenex Lux 5µm Cellulose-3 250 x 4.6 mm, water/acetonitrile 45/55, 0.5mL/min).

^{1}H NMR ($CDCl_3$, 600 MHz) δ 7.73 (1H, dt, *J* = 8.5, 0.7 Hz), 7.69 (1H, d, *J* = 1.9 Hz), 7.34 (1H, d, *J* = 5.4 Hz), 7.27 (1H, dd, *J* = 8.5, 1.9 Hz), 7.23 (1H, dd, *J* = 5.4, 0.8 Hz), 6.02 (1H, dd, *J* = 17.5, 10.7 Hz), 1.86 – 1.68 (4H, m), 1.58 (3H, d, *J* = 1.4 Hz), 1.49 (4H, s), 1.44 (3H, d, *J* = 1.4 Hz)

^{13}C{^{1}H} NMR ($CDCl_3$, 151 MHz) δ 147.2, 143.9, 139.9, 137.4, 131.5, 126.4 (d, *J* = 3.4 Hz), 124.8, 124.2, 123.9, 122.1, 121.5, 112.0, 44.5, 41.4, 25.8, 25.4, 23.5, 17.7

HRMS (ESI): m/z calc. for $C_{18}H_{22}S+H^+$ 271.1515; found 271.1521

IR (ATR, film) ṽ [cm^{-1}] = 3075, 2971, 2859, 2923, 1636, 1442, 912, 811, 702, 702.

$[\propto]_D^{20} = +5.0$ (c = 0.1, CH_3CN)

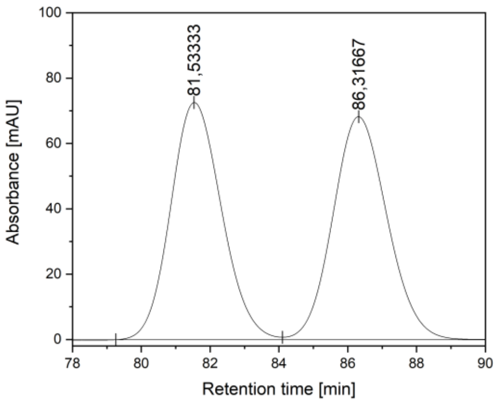

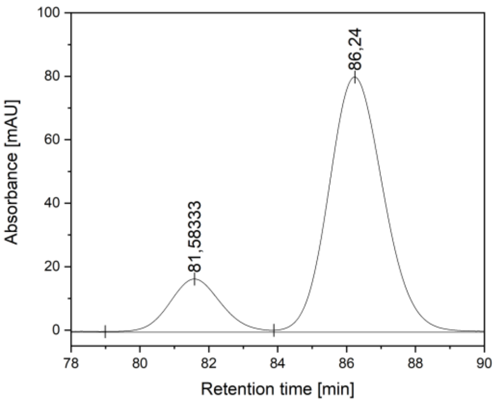

		Retention time	Area			Height	Relative Area
		min	mAU*min	n.a.		mAU	%
	1	81.533	125.5888	n.a.	BM *	72.732	49.87
	2	86.317	125.8849	n.a.	M *	68.417	49.99
Total:			251.821	0		141.413	100

		Retention time	Area			Height	Relative Area
		min	mAU*min	n.a.		mAU	%
	1	81.583	28.1826	n.a.	BM *	16.534	16.02
	2	86.24	147.7613	n.a.	MB*	80.156	83.98
Total:			175.9439	0		96.69	100

(*S*)-5-(3,7-dimethylocta-1,6-dien-3-yl)-2-methoxypyridine (5n)

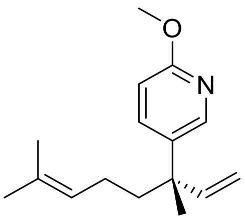

The title compound was prepared from 5-bromo-2-methoxypyridine (37.6 mg, 0.2 mmol) according to the general procedure for asymmetric γ-selective Suzuki-Miyaura coupling using (*Z*)-2-(3,7-dimethylocta-2,6-dien-1-yl)-5,5-dimethyl-1,3,2-dioxaborinane as allylic boronate. The crude product was purified using column chromatography (silica gel; petrol ether:ethyl acetate 100:0 to 98:2) to afford the title compound (21.2 mg, 0.09 mmol, 43%) as a colorless oil. The enantiomeric ratio was determined to be 73:27 (*S*:*R*) following HPLC-analysis comparing to an authentic racemic sample (HPLC: Phenomenex Lux 5μm Cellulose-3 250 x 4.6 mm, water/acetonitrile 40/60, 0.5mL/min).

^{1}H NMR ($CDCl_3$, 600 MHz) δ 8.10 (1H, dd, *J* = 2.6, 0.8 Hz), 7.52 (1H, dd, *J* = 8.7, 2.7 Hz), 6.69 (1H, dd, *J* = 8.7, 0.8 Hz), 5.98 (1H, dd, *J* = 17.5, 10.7 Hz), 5.11 (1H, dd, *J* = 10.7, 1.2 Hz), 5.06 (1H, tp, *J* = 7.0, 1.4 Hz), 5.02 (1H, dd, *J* = 17.5, 1.2 Hz), 3.92 (3H, s), 1.82 – 1.67 (4H, m), 1.65 (3H, s), 1.51 (3H, s), 1.37 (3H, s)

^{13}C{^{1}H} NMR ($CDCl_3$, 151 MHz) δ 162.5, 146.4, 144.8, 138.0, 135.4, 131.7, 124.5, 112.5, 110.3, 53.6, 42.6, 41.1, 25.8, 24.9, 23.3, 17.7

HRMS (ESI): m/z calc. for $C_{16}H_{23}NO+H^{+}$ 246.1852; found 246.1851

IR (ATR, film) ṽ [cm^{-1}] = 2967, 1604, 1493, 1379, 1288, 1029.

$[\propto]_{D}^{20} = +3.0$ (c = 0.1, CH_3CN)

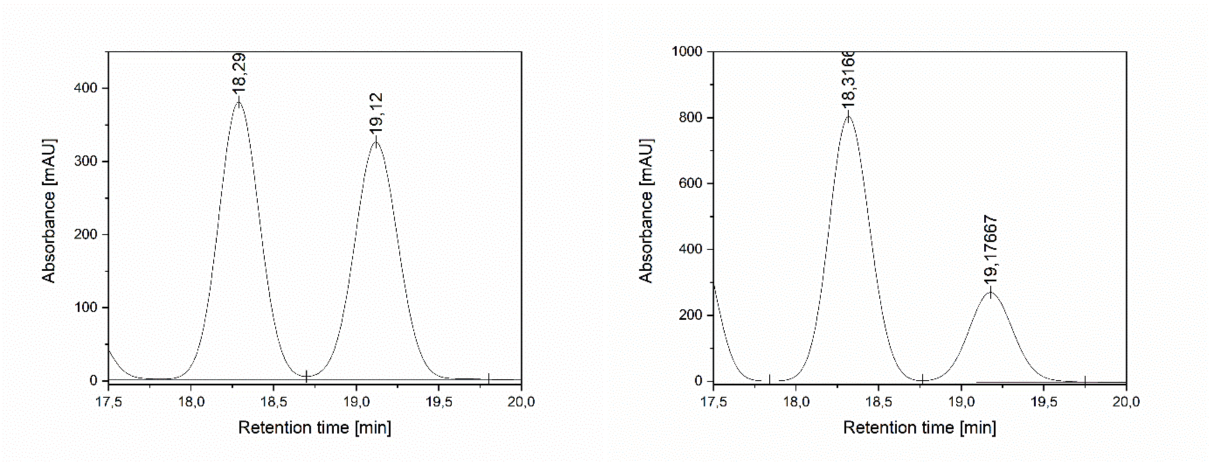

		Retention Time	Area			Height	Relative Area
		min	mAU*min	n.a.		mAU	%
	1	18.29	113.928	n.a.	M *	380.294	52.34
	2	19.12	103.7265	n.a.	M *	325.585	47.66
Total:			217.6546	0		705.879	100

		Retention Time	Area			Height	Relative Area
		min	mAU*min	n.a.		mAU	%
	1	18.317	241.6696	n.a.	M *	806.173	73.22
	2	19.177	88.3831	n.a.	MB*	272.608	26.78
Total:			330.0527	0		1078.781	100

(*S,E*)-5-(3,7,11-trimethyldodeca-1,6,10-trien-3-yl)-1*H*-indole (5o)

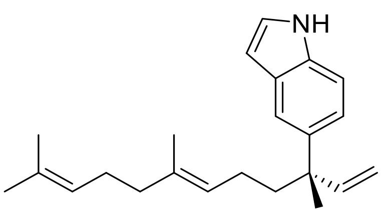

The title compound was prepared from 5-bromoindole (39.2 mg, 0.2 mmol) according to the general procedure for asymmetric γ-selective Suzuki-Miyaura coupling using 5,5-dimethyl-2-((2*E*,6*E*)-3,7,11-trimethyldodeca-2,6,10-trien-1-yl)-1,3,2-dioxaborinane as allylic boronate. The crude product was purified using column chromatography (silica gel; petrol ether:ethyl acetate 100:0 to 98:2) to afford the title compound (55.6 mg, 0.17 mmol, 86%) as a colorless oil. The enantiomeric ratio was determined to be 85:15 (*S*:*R*) following HPLC-analysis comparing to an authentic racemic sample (HPLC: Phenomenex Lux 5µm Cellulose-3 250 x 4.6 mm, water/acetonitrile 45/55, 0.5mL/min).

^{1}H NMR ($CDCl_3$, 600 MHz) δ 8.05 (1H, s), 7.63 (1H, d, *J* = 1.8, 0.9 Hz), 7.33 (1H, dt, *J* = 8.6, 0.9 Hz), 7.23 (1H, dd, *J* = 8.6, 1.8 Hz), 7.17 (1H, dd, *J* = 3.2, 2.4 Hz), 6.54 (1H, ddd, *J* = 3.1, 2.0, 0.9 Hz), 6.15 (1H, dd, *J* = 17.5, 10.8 Hz), 5.19 – 5.08 (4H, m), 2.13 – 1.77 (8H, m), 1.72 (3H, s), 1.63 (3H, s), 1.55 (3H, s), 1.49 (3H, s)

^{13}C{^{1}H} NMR ($CDCl_3$, 151 MHz) δ 148.1, 139.0, 134.9, 134.2, 131.4, 127.9, 125.0, 124.6, 124.3, 121.6, 118.3, 111.2, 110.7, 102.8, 44.3, 41.6, 39.8, 26.9, 25.8, 25.6, 23.5, 17.8, 16.1

HRMS (ESI): m/z calc. for $C_{23}H_{31}N+H^+$ 322.2529; found 322.2528

IR (ATR, film) ṽ [cm^{-1}] = 3413, 2965, 2916, 1633, 1470, 1412, 1374, 1318, 1251, 1094, 1064, 1000, 910, 803, 764, 723, 681, 605.

$[\propto]_D^{20} = -11.0$ (c = 0.1, CH_3CN)

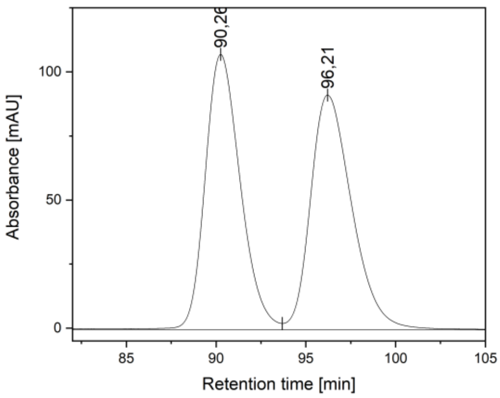

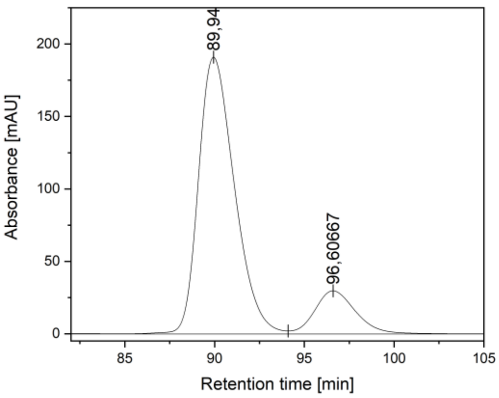

		Retention time	Area			Height	Relative Area
		min	mAU*min	n.a.		mAU	%
	1	90.26	241.128	n.a.	BM *	107.09	49.94
	2	96.21	241.6817	n.a.	MB*	91.26	50.06
Total:			482.8098	0		198.349	100

		Retention time	Area			Height	Relative Area
		min	mAU*min	n.a.		mAU	%
	1	89.94	442.3854	n.a.	BM *	190.972	84.93
	2	96.607	78.5191	n.a.	MB*	29.713	15.07
Total:			520.9045	0		220.684	100

(*S*)-5-(hept-1-en-3-yl)-1H-indole (5p)

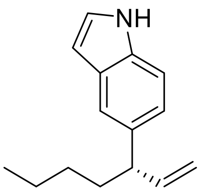

The title compound was prepared from 5-bromoindole (39.2 mg, 0.2 mmol) according to the general procedure for asymmetric γ-selective Suzuki-Miyaura coupling using (*E*)-2-(hept-2-en-1-yl)-5,5-dimethyl-1,3,2-dioxaborinane as allylic boronate. The crude product was purified using column chromatography (silica gel; petrol ether:ethyl acetate 100:0 to 98:2) to afford the title compound (30.7 mg, 0.14 mmol, 72%) as a colorless oil. The enantiomeric ratio was determined to be 36:64 (*S*:*R*) following HPLC-analysis comparing to an authentic racemic sample (HPLC: Chiralcel ODH 250 x 4.6 mm, n-heptane/^{i}PrOH 75/25, 0.5mL/min).

^{1}H NMR ($CDCl_3$, 600 MHz) δ 8.07 (1H, s), 7.46 (1H, dd, *J* = 1.6, 0.8 Hz), 7.33 (1H, dt, *J* = 8.3, 0.9 Hz), 7.18 (1H, dd, *J* = 3.2, 2.4 Hz), 7.05 (1H, dd, *J* = 8.4, 1.7 Hz), 6.55 – 6.47 (1H, m), 6.04 (1H, ddd, *J* = 17.5, 10.2, 7.5 Hz), 5.04 (1H, dt, *J* = 17.2, 1.5 Hz), 5.00 (1H, ddd, *J* = 10.2, 1.9, 1.0 Hz), 3.33 (1H, q, *J* = 7.5 Hz), 1.86 – 1.71 (2H, m), 1.41 – 1.12 (4H, m), 0.92 – 0.82 (3H, m)

^{13}C{^{1}H} NMR ($CDCl_3$, 151 MHz) δ 143.8, 136.3, 134.7, 128.2, 124.4, 122.3, 119.3, 113.2, 111.0, 102.6, 50.1, 35.7, 30.0, 22.9, 14.2

HRMS (ESI): m/z calc. for $C_{15}H_{19}N+H^+$ 214.1590; found 214.1592

IR (ATR, film) ṽ [cm^{-1}] = 3413, 2956, 2927, 2857, 1635, 1473, 1414, 1342, 1090, 993, 910, 803, 764, 725, 606.

$[\propto]_D^{20} = -4.0$ (c = 0.1, CH_3CN)

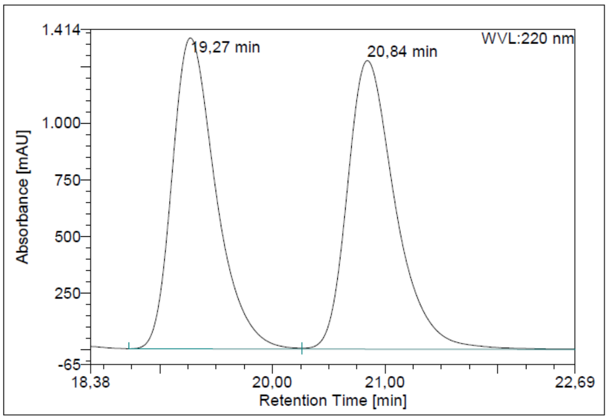

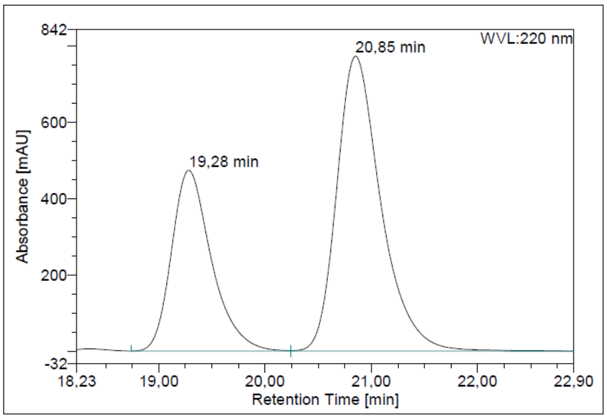

No.	Ret.Time min	Peak Name	Height mAU	Area mAU*min	Rel.Area %	Amount	Type
1	19,27	n.a.	1372,308	616,034	49,44	n.a.	BM *
2	20,84	n.a.	1273,574	630,092	50,56	n.a.	MB*
Total:			2645,882	1246,125	100,00	0,000	

No.	Ret.Time min	Peak Name	Height mAU	Area mAU*min	Rel.Area %	Amount	Type
1	19,28	n.a.	472,534	205,679	35,60	n.a.	BM *
2	20,85	n.a.	771,394	372,151	64,40	n.a.	MB*
Total:			1243,928	577,830	100,00	0,000	

(*R*)-1-(hept-1-en-3-yl)-4-methoxybenzene (5q)

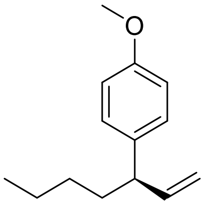

The title compound was prepared from 4-bromoanisole (25 µL, 0.2 mmol) according to the general procedure for asymmetric γ-selective Suzuki-Miyaura coupling using (*E*)-2-(hept-2-en-1-yl)-5,5-dimethyl-1,3,2-dioxaborinane as allylic boronate. The crude product was purified using column chromatography (silica gel; petrol ether:ethyl acetate 100:0) to afford the title compound (22.8 mg, 0.11 mmol, 56%) as a colorless oil. The enantiomeric ratio was determined to be 45:55 (*S*:*R*) following HPLC-analysis comparing to an authentic racemic sample (HPLC: Daicel Chiralpak IC 250 x 4.6 mm, n-heptane/^{i}PrOH 99.5/0.5, 0.5mL/min, 10°C).

^{1}H NMR ($CDCl_3$, 600 MHz) δ 7.13 – 7.08 (2H, m), 6.89 – 6.81 (2H, m), 5.98 – 5.88 (1H, m), 5.00 (1H, ddd, *J* = 7.0, 1.8, 1.1 Hz), 4.98 (1H, d, *J* = 1.1 Hz), 3.79 (4H, s), 3.19 (1H, q, *J* = 7.6 Hz), 1.73 – 1.61 (2H, m), 1.35 – 1.24 (3H, m), 1.18 (1H, dtdd, *J* = 13.0, 10.0, 6.5, 4.4 Hz), 0.87 (3H, t, *J* = 7.2 Hz)

^{13}C{^{1}H} NMR ($CDCl_3$, 151 MHz) δ 158.0, 143.1, 136.9, 128.6, 113.9, 113.6, 55.4, 49.1, 35.3, 29.9, 22.8, 14.2

HRMS (EI): m/z calc. for $C_{14}H_{20}O^+$ 204.1514; found 204.1521

IR (ATR, film) ṽ [cm^{-1}] = 2955.8, 2928.4, 2858.1, 1610.6, 1510.4, 1464.8, 1301.2, 1246.2, 1177.0, 1038.4, 993.3, 911.0, 828.1, 655.9, 540.2.

$[\alpha]_D^{20} = +3.8$ (c=1, $CHCl_3$)

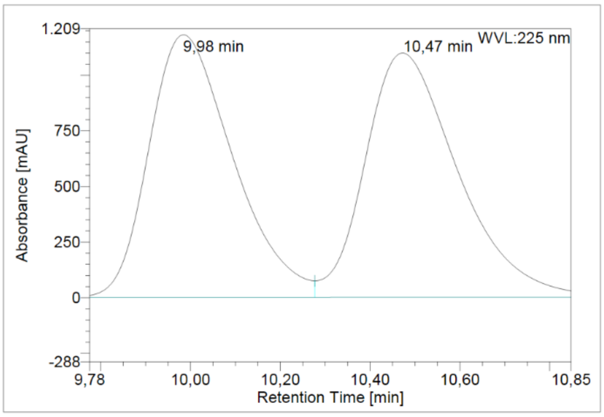

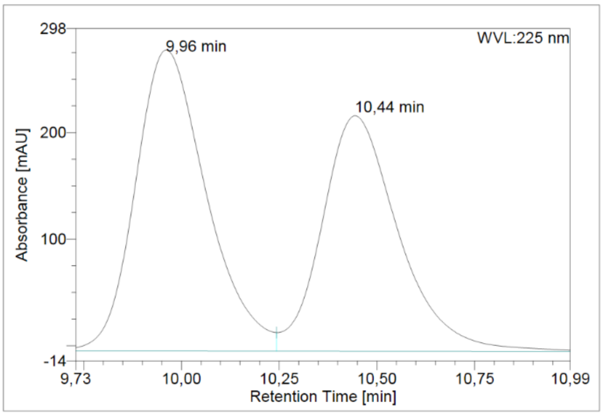

No.	Ret.Time min	Peak Name	Height mAU	Area mAU*min	Rel.Area %	Amount	Type
1	9,98	n.a.	1180,355	255,934	49,26	n.a.	BM
2	10,47	n.a.	1098,015	263,582	50,74	n.a.	MB
Total:			2278,369	519,516	100,00	0,000	

No.	Ret.Time min	Peak Name	Height mAU	Area mAU*min	Rel.Area %	Amount	Type
1	9,96	n.a.	282,523	59,526	54,26	n.a.	BM
2	10,44	n.a.	220,910	50,186	45,74	n.a.	MB
Total:			503,433	109,712	100,00	0,000	

(+)-(*S*)-Sporochnol (2)

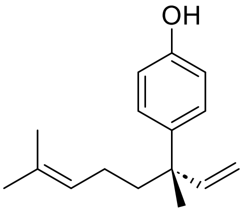

The title compound was prepared from 4-iodophenol (44.0 mg, 0.2 mmol) according to the general procedure for asymmetric γ-selective Suzuki-Miyaura coupling using (*Z*)-2-(3,7-dimethylocta-2,6-dien-1-yl)-5,5-dimethyl-1,3,2-dioxaborinane as allylic boronate. The crude product was purified using column chromatography (silica gel; petrol ether:ethyl acetate 95:5 to 80:20) to afford (+)-(*S*)-Sporochnol (27.6 mg, 0.12 mmol, 60%) as a colorless oil. The analytical data is in accordance with the published literature.[7] The enatiomeric ratio was determined to be 96:4 (*S*:*R*) following HPLC-analysis comparing to an authentic racemic sample (HPLC: Phenomenex Lux 5µm Cellulose-3 250 x 4.6 mm, water/acetonitrile 30/70, 0.5mL/min).

^{1}H NMR ($CDCl_3$, 600 MHz) δ 7.20 – 7.16 (2H, m), 6.80 – 6.72 (2H, m), 6.00 (1H, dd, *J* = 17.5, 10.7 Hz), 5.12 – 4.99 (3H, m), 1.87 – 1.66 (4H, m), 1.66 (3H, s), 1.51 (3H, s), 1.34 (3H, s)

^{13}C{^{1}H} NMR ($CDCl_3$, 151 MHz) δ 153.6, 147.3, 139.9, 131.5, 128.0, 124.9, 115.0, 111.6, 43.8, 41.4, 25.8, 25.2, 23.4, 17.7

MS (EI): m/z calc. for $C_{16}H_{22}O^{+}$ 231.17; found 231.18

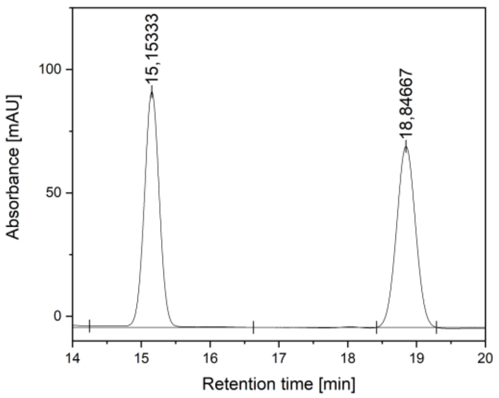

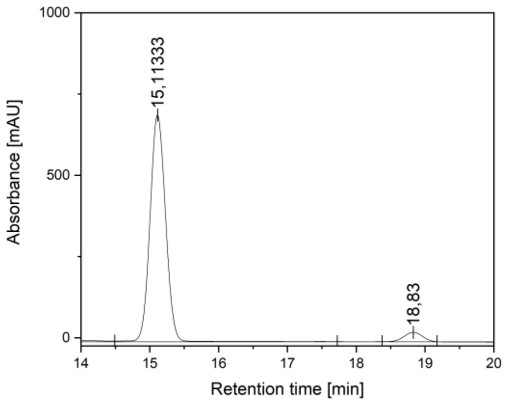

		Retention time	Area			Height	Relative Area
		min	mAU*min	n.a.		mAU	%
1		15.14	23.6532	n.a.	BMB*	95.019	49.87
2		18.833	23.773	n.a.	BMB*	73.671	50.13
Total:			47.4262	0		168.69	100

		Retention time	Area			Height	Relative Area
		min	mAU*min	n.a.		mAU	%
1		15.1	179.2481	n.a.	BMB*	695.274	95.55
2		18.817	8.5396	n.a.	BMB*	28.136	4.45
Total:			187.7877	0		723.41	100

5. Application in natural product and derivative synthesis

(*S*)-5-Bromo-tryptophan (11)

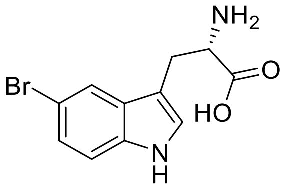

The title compound was prepared according to the procedure from Romney *et al.*[8] The cells containing the overexpressed protein were prepared in line with the description in Chapter 6 (Biological work). The freshly prepared cells (5 g) were suspended in 25 mL of 50 mM potassium phosphate buffer (pH 8) containing 1.3 mg of pyridoxal phosphate and were lysed using an ultrasonic cell disruptor three times for 10-minutes at 35–40% amplitude. The cell debris was removed via centrifugation (4 °C, 10,000 × rcf, 20 minutes) and decantation of the supernatant. The crude lysate was heated to 70°C for 30 minutes, after which further centrifugation (4 °C, 10,000 × rcf, 20 minutes) yielded the supernatant as purified lysate.

A 500 mL Schott bottle was charged with 5-bromo indole (1.32 g, 6.75 mmol, 1 equiv.), serine (851 mg, 8.1 mmol, 1.2 equiv.) and PLP (83 mg, 0.05 mol%). Subsequently, DMSO (6.75 mL, 5% (v/v)), the purified lysate (25 mL) and 100mM potassium phosphate buffer (pH 8) (103 mL, 0.05 M final concentration concerning the indole) were added and the reaction mixture was shaken for 24 hours at 65°C/180 rpm. The mixture was allowed to reach room temperature and was then cooled at 0°C for an hour. The crystallized product was removed via filtration, washed with ice cooled water and ethyl acetate and dried *in vacuo*. (*S*)-5-Bromo-tryptophan (1.66 g, 5.9 mmol, 87%) was received as a slightly off white solid. The analytical data is in accordance with the published literature.[8]

^{1}H NMR (MeOD, 600 MHz) δ 7.91 (1H, d, *J* = 1.9 Hz), 7.29 (1H, d, *J* = 8.6 Hz), 7.23 (1H, s), 7.21 (1H, dd, *J* = 8.6, 1.9 Hz), 3.82 (1H, dd, *J* = 9.1, 4.1 Hz), 3.45 (1H, ddd, *J* = 15.3, 4.2, 0.9 Hz), 3.14 (1H, dd, *J* = 15.3, 9.1 Hz)

^{13}C{^{1}H} NMR (MeOD, 151 MHz) δ 174.2, 137.0, 130.4, 126.7, 125.5, 122.1, 114.1, 113.4, 109.5, 56.7, 28.2

MS (APCI): m/z calc. for $C_{11}H_{12}N_2O_2Br+H^+$: 283.1; found: 283.1

m.p.: 283 °C - 284 °C

(3*S*,6*S*)-3-((1H-indol-3-yl)methyl)-6-((5-bromo-1H-indol-3-yl)methyl)piperazine-2,5-dione (7)

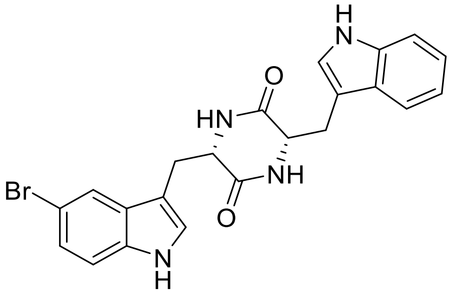

The title compound was prepared based on the procedure from Haase *et al.* over three steps.[9] An oven dried Schlenk flask was charged with (*S*)-5-Bromo-tryptophan (1.5 g, 5.3 mmol, 1 equiv.). Anhydrous MeOH (53 mL, 0.1 M concerning the tryptophan) was added and the resulting suspension was cooled to 0°C. Thionyl chloride (1.15 mL, 15.9 mmol, 3 equiv.) was added dropwise, after which rhe reaction mixture was heated to 60°C overnight. The mixture was cooled to 0°C and the reaction was quenched by the addition of water (20 mL). Solid sodium bicarbonate was added until the aqueous phase reached ph 8. The aqueous phase was extracted three times using ethyl acetate. The combined organic phase was dried over magnesium sulphate and concentrated *in vacuo* to give the crude tryptophan methyl ester.

An oven dried Schlenk flask was charged with Boc-Trp-OH (1.774 g, 5.83 mmol, 1.1 equiv.), after which anhydrous THF (27.5 mL, 0.2 M concerning the tryptophan) was added and the resulting suspension was cooled to 0°C. HCTU (2.412 g, 5.83 mmol, 1.1 equiv.) was added as a solid in one portion, followed by the dropwise addition of triethylamine (0.95 mL, 6.89 mmol, 1.3 equiv.).After stirring the resulting mixture for half an hour at 0°C, the crude tryptophan methyl ester (dissolved in anhydrous THF, 27.5 mL) was added dropwise. The reaction mixture was allowed to warm to room temperature and stirred for three hours. The reaction solution was diluted using ethyl acetate (to approximately 200 mL total volume) and transferred into a separatory funnel. The resulting organic phase was washed to times with aq. NaOH (1 M), two times with aq. HCl (1 M) and brine, dried over magnesium sulphate and concentrated in vacuo. The crude dipeptide was used directly in the next step without further purification.

In a round bottom flask, the crude dipeptide from was redissolved in anhydrous dichloromethane (10 mL) and cooled to 0°C. Trifluoracetic acid (5 mL) was added dropwise and the solution was allowed to warm to room temperature. The reaction solution was stirred at room temperature for 2 hours, after which the volatiles were removed *in vacuo* to yield the crude Boc-deprotected dipeptide trifluoracetic acid salt. The crude product was dissolved in methanolic ammonia (7*N* NH_3 in MeOH) and stirred at 60°C for 4 hours. The volatiles were removed *in vacuo* and purification of the solid residue via column chromatography (ethyl acetate) gave (3*S*,6*S*)-3-((1H-indol-3-yl)methyl)-6-((5-bromo-1H-indol-3-yl)methyl)piperazine-2,5-dione (further referred to as 5-Br-cWW) (1.55 g, 3.4 mmol, 65%) as a white amorphous solid. The analytical data is in accordance with the published literature.[9]

^{1}H NMR (MeOD, 600 MHz) δ 7.68 (1H, s), 7.44 (1H, d, *J* = 8.0 Hz), 7.32 (1H, d, *J* = 8.0 Hz), 7.27 – 7.15 (2H, m), 7.11 (1H, t, *J* = 7.6 Hz), 7.03 (1H, t, *J* = 7.6 Hz), 6.64 (1H, s), 6.40 (1H, s), 4.04 (2H, d, *J* = 33.7 Hz), 2.96 (1H, d, *J* = 14.5 Hz), 2.83 (1H, d, *J* = 14.5 Hz), 2.29 (1H, dd, *J* = 14.6, 7.3 Hz), 2.02 (1H, dd, *J* = 14.8, 7.8 Hz)

$^{13}C\{^1H\}$ NMR (MeOD, 151 MHz) δ 169.7, 138.1, 136.7, 130.4, 128.7, 127.5, 125.7, 125.3, 122.6, 122.2, 120.2, 119.7, 114.1, 113.3, 112.5, 109.6, 109.4, 57.1, 56.6, 31.4, 31.2

MS (APCI): m/z calc. for $C_{22}H_{20}N_4O_2Br+H^+$: 452.3; found: 452.3

m.p.: 179 °C - 180 °C

(3S,6S)-3-((1H-indol-3-yl)methyl)-6-((5-((R)-3,7-dimethylocta-1,6-dien-3-yl)-1H-indol-3-yl)methyl)piperazine-2,5-dione (8a)

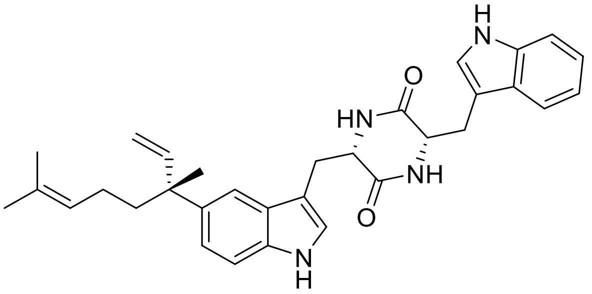

The title compound was prepared from 5-Br-cWW (50 mg, 0.11 mmol) according to the general procedure for asymmetric γ-selective Suzuki-Miyaura coupling using (*E*)-2-(3,7-dimethylocta-2,6-dien-1-yl)-5,5-dimethyl-1,3,2-dioxaborinane as allylic boronate. The crude product was purified using column chromatography (silica gel; petrol ether:acetone 50:50) to afford the title compound (40.3 mg, 0.079 mmol, 72%) as a colorless amorphous solid. The diastereomeric ratio was determined to be 13:87 (*S*:*R*) concerning the newly generated stereogenic centre, following analysis of the obtained ^{1}H-NMR-spectra comparing to the spectra obtained from a 50:50 mixture of the two diastereomers. The 50:50 diastereomeric mixture was obtained in the same manner as the title compound, with the exception that the general procedure for asymmetric γ-selective Suzuki-Miyaura coupling was performed with SPhos as (achiral) ligand.

^{1}H NMR (MeOD, 600 MHz) δ 7.54 – 7.46 (2H, m), 7.32 (1H, dt, *J* = 8.2, 0.9 Hz), 7.26 – 7.21 (1H, m), 7.10 (2H, ddt, *J* = 11.4, 7.0, 1.5 Hz), 7.04 – 7.00 (1H, m), 6.52 (1H, s), 6.34 (1H, s), 6.17 – 6.11 (1H, m), 5.15 – 5.00 (3H, m), 4.05 (2H, dddd, *J* = 17.2, 7.5, 3.8, 1.1 Hz), 2.96 – 2.89 (2H, m), 2.21 (1H, dd, *J* = 14.4, 7.1 Hz), 2.09 (1H, dd, *J* = 14.3, 7.5 Hz), 1.91 – 1.82 (2H, m), 1.82 – 1.72 (2H, m), 1.62 (3H, s), 1.46 (3H, s), 1.44 (3H, s)

$^{13}C\{^1H\}$ NMR (MeOD, 151 MHz) δ 169.7, 169.7, 149.4, 139.3, 138.1, 136.4, 131.7, 128.6, 128.3, 126.2, 126.1, 125.9, 122.5, 122.1, 120.1, 119.7, 117.2, 112.4, 111.9, 111.4, 109.5, 109.4, 56.8, 56.7, 45.3, 42.7, 31.5 (d, *J* = 5.2 Hz), 31.4, 26.1, 25.8, 24.6, 20.8, 17.6

HRMS (ESI): m/z calc. for $C_{32}H_{36}N_4O_2+H^+$ 509.2911; found 509.2905

IR (ATR, film) ṽ [cm^{-1}] = 3324, 2969, 2915, 1671, 1453, 1323, 1257, 1088, 914, 744

$[\alpha]_D^{20} = -86.0$ (c = 0.1, CH_3CN)

m.p.: 171 °C - 184 °C

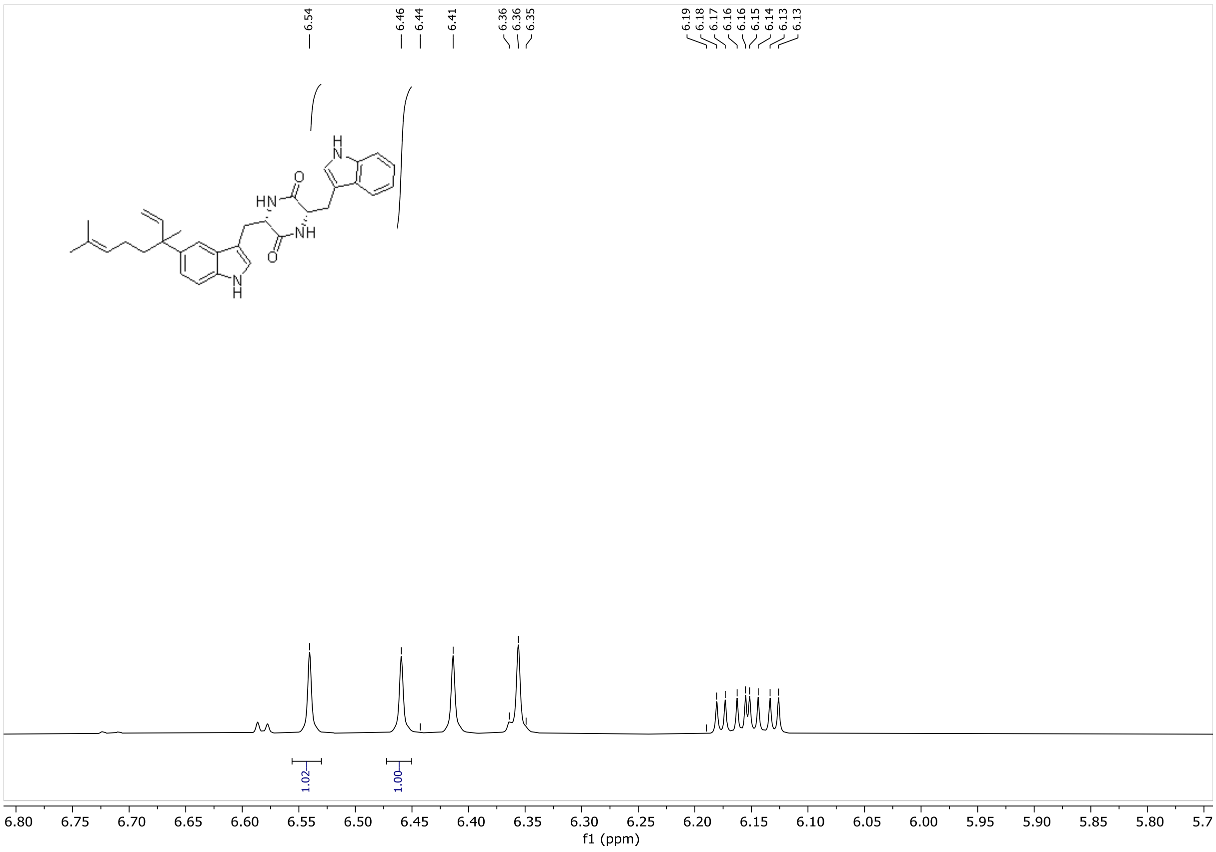

*Figure S1 Cutout from the ^{1}H-NMR of a 50:50 diastereomeric mixture of Compound **8a.***

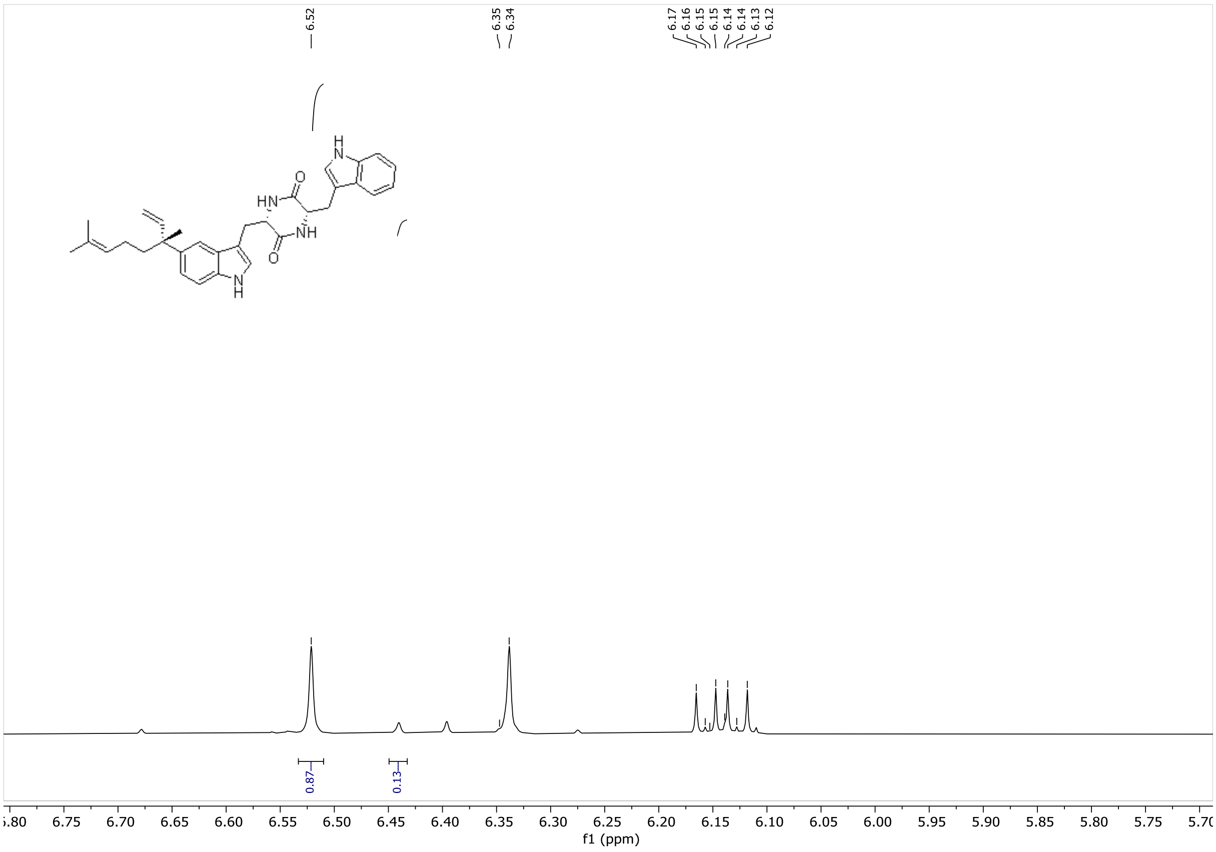

*Figure S2 Cutout from the ^{1}H-NMR of diastereomerically enriched Compound **8a**.*

(3S,6S)-3-((1H-indol-3-yl)methyl)-6-((5-((R,E)-3,7,11-trimethyldodeca-1,6,10-trien-3-yl)-1H-indol-3-yl)methyl)piperazine-2,5-dione (8b)

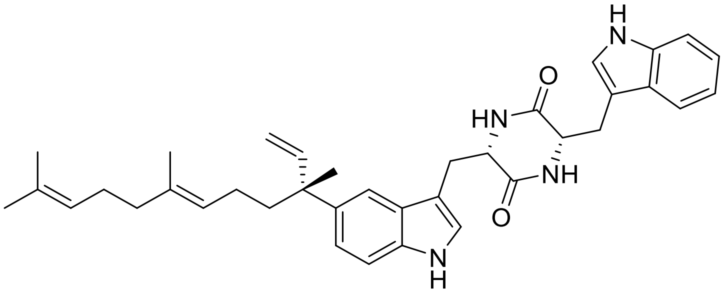

The title compound was prepared from 5-Br-cWW (50 mg, 0.11 mmol) according to the general procedure for asymmetric γ-selective Suzuki-Miyaura coupling using 5,5-dimethyl-2-((2*E*,6*E*)-3,7,11-trimethyldodeca-2,6,10-trien-1-yl)-1,3,2-dioxaborinane as allylic boronate. The crude product was purified using column chromatography (silica gel; petrol ether:acetone 50:50) to afford the title compound (49.6 mg, 0.086 mmol, 78%) as a colorless amorphous solid. The diastereomeric ratio was determined to be 14:86 (*S*:*R*) concerning the newly generated stereogenic centre, following analysis of the obtained ^{1}H-NMR-spectra comparing to the spectra obtained from a 50:50 mixture of the two diastereomers. The 50:50 diastereomeric mixture was obtained in the same manner as the title compound, with the exception that the general procedure for asymmetric γ-selective Suzuki-Miyaura coupling was performed with SPhos as (achiral) ligand.

^{1}H NMR (MeOD, 600 MHz) δ 7.55 – 7.45 (2H, m), 7.31 (1H, d, *J* = 8.2, 1.2 Hz), 7.24 (1H, dd, *J* = 8.6, 2.6 Hz), 7.14 – 7.07 (2H, m), 7.02 (1H, ddd, *J* = 8.1, 7.0, 1.1 Hz), 6.53 (1H, s), 6.33 (1H, s), 6.15 (1H, ddd, *J* = 17.0, 10.8, 6.1 Hz), 5.16 – 4.99 (4H, m), 4.05 (2H, dddd, *J* = 18.1, 7.6, 3.9, 1.2 Hz), 2.93 (2H, dq, *J* = 14.4, 3.5 Hz), 2.21 (1H, td, *J* = 14.5, 7.2 Hz), 2.08 (1H, dd, *J* = 14.5, 7.5 Hz), 2.01 (2H, q, *J* = 7.5 Hz), 1.95 – 1.72 (6H, m), 1.64 (3H, s), 1.57 (3H, s), 1.47 – 1.42 (6H, m)

^{13}C{^{1}H} NMR (MeOD, 151 MHz) δ 168.3, 168.2, 148.1, 137.9, 136.7, 135.1, 134.1, 130.6, 127.3, 126.9, 124.9, 124.8, 124.5, 124.1, 121.1, 120.7, 118.7, 118.3, 115.8, 111.1, 110.5, 110.0, 108.1, 108.0, 55.4, 55.3, 44.0, 41.4, 39.4, 30.1, 30.0, 26.4, 24.7, 24.5, 23.1, 16.4, 14.6

HRMS (ESI): m/z calc. for $C_{37}H_{44}N_4O_2$+H+ 577.3537; found 577.3530

IR (ATR, film) ṽ [cm^{-1}] = 3318, 2964, 2921, 2855, 1670, 1456, 1323, 1088, 909, 743.

$[\alpha]_D^{20} = -81.0$ (c = 0.1, CH_3CN)

m.p.: 167 °C - 169 °C

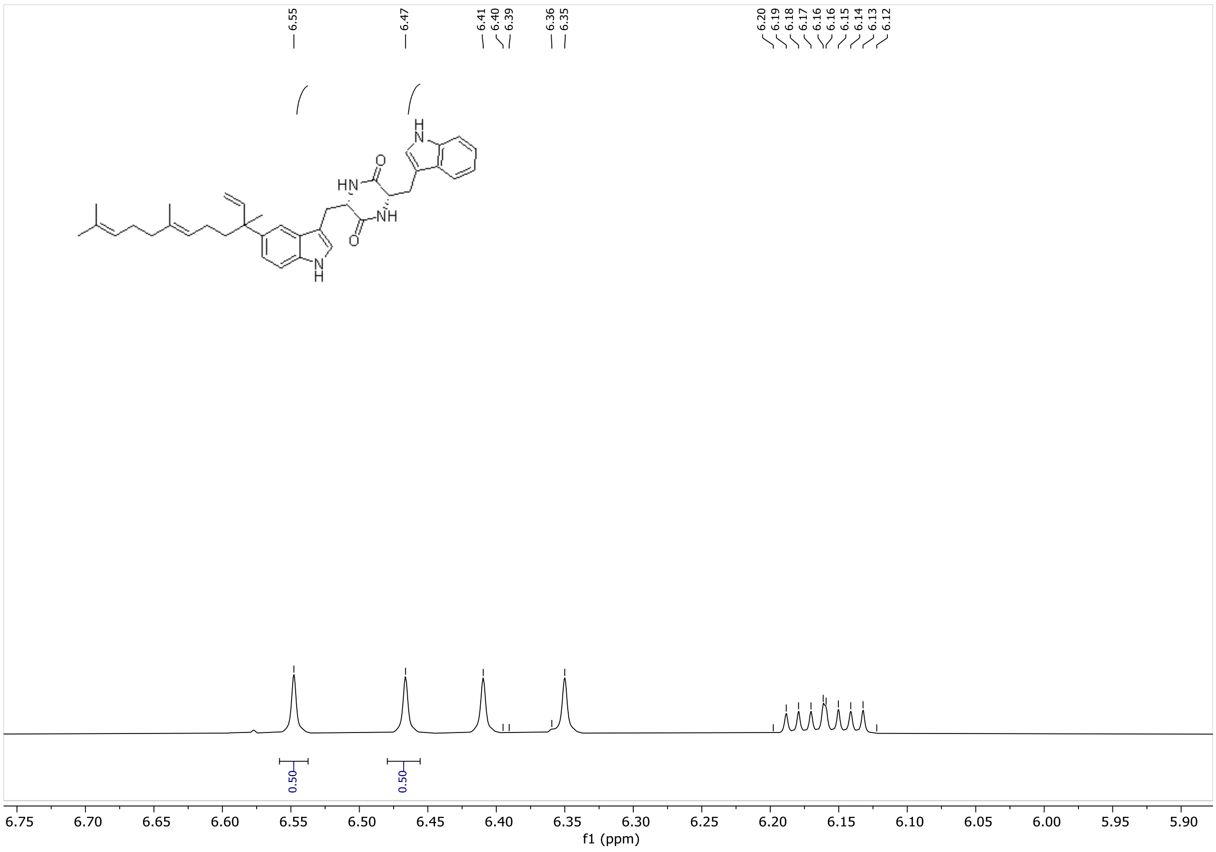

*Figure S3 Cutout from the ^{1}H-NMR of a 50:50 diastereomeric mixture of Compound **8b**.*

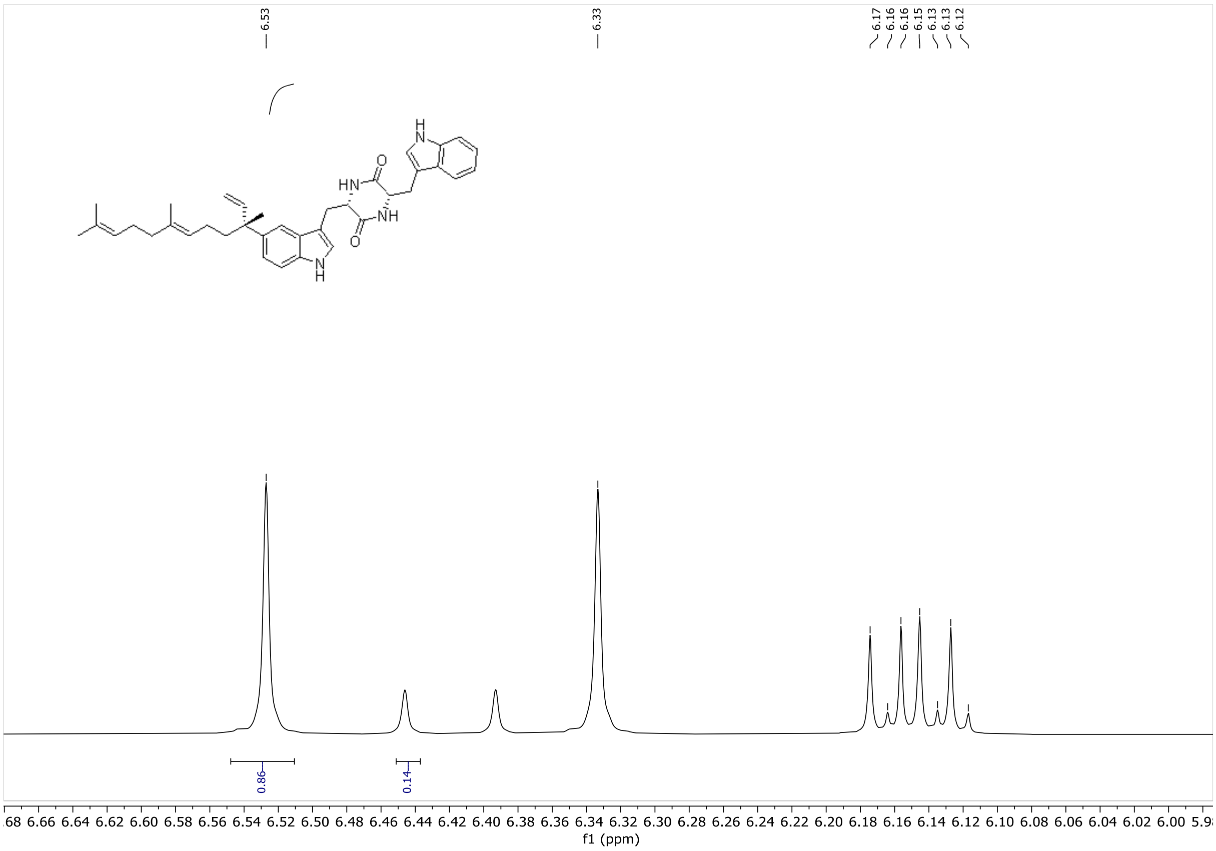

*Figure S4 Cutout from the 1H-NMR of diastereomerically enriched Compound **8b**.*

(3*S*,6*S*)-3-((1H-indol-3-yl)methyl)-6-((5-bromo-1H-indol-3-yl)methyl)piperazine-2,5-dione (9)

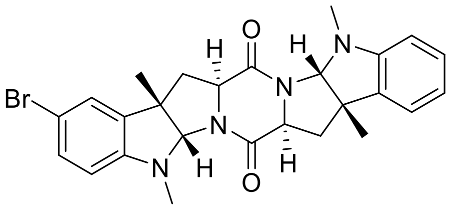

The title compound was prepared from 5-Br-cWW (100 mg, 0.22 mmol) over two steps. The first step was conducted according to the procedure of Haase *et al.*[9] The cells containing the overexpressed protein were prepared in line with the description in Chapter 6 (Biological work). The freshly prepared cells were resuspended in reaction buffer (50 mM KPi, pH 8) and lysed using an ultrasonic cell disruptor for two 10-minute sessions at 35–40% amplitude. Following lysis, the cell debris was removed by centrifugation (4 °C, 10,000 × rcf, 20 minutes), and the supernatant was collected as the lysate. The lysate was incubated washed Protino® Ni-NTA agarose suspension at 4 °C for 40 minutes with gentle end-over-end mixing. After removing the supernatant, the enzyme-bound beads were washed with washing solution (50 mM KPi, pH 8, 40 mM imidazole) for 10 minutes at 4 °C. Residual imidazole was removed by two additional washes with reaction buffer (50 mM KPi, pH 8).

The Ni-NTA-bound enzyme was transferred to a Schott bottle and the desired volume was adjusted with buffer (1 mM). The reaction mixture was prepared by adding 5-Br-cWW substrate (dissolved in DMSO, 1 mM 5-Br-cWW final concentration) and S-adenosylmethionine disulfate tosylate (3 mM final concentration) in KPi buffer (50 mM, pH 8). The reaction proceeded at 40 °C with gentle shaking at 300 rpm. After 48 hours, the Ni-NTA resin was removed by filtration. The product was extracted three times with ethyl acetate, dried over magnesium sulphate, and concentrated under reduced pressure. The product of the first step was purified via column chromatography (ethyl acetate).

A round bottom flask was charged with the product of the first step. The solid was dissolved in acetonitrile (1 mL) and the mixture was cooled to 0°C. Subsequently, formaldehyde (71 µL, 0.88 mmol, 4 equiv. 37% in MeOH), acetic acid (50 µL, 0.88 mmol, 4 equiv.) and sodium cyanoborohydride (55.3 mg, 0.88 mmol, 4 equiv.) are added successively. The mixture is stirred at 0°C for two hours, after which the reaction is quenched using aq. NaOH (1 M, 5 mL). The resulting mixture is extracted three times using ethyl acetate, dried over magnesium sulphate and dried *in vacuo*. The crude product is purified via coloumn chromatography to deliver (3*S*,6*S*)-3-((1H-indol-3-yl)methyl)-6-((5-bromo-1H-indol-3-yl)methyl)piperazine-2,5-dione () as white amorphous solid. The analytical data is in accordance with the published literature.[9]

^{1}H NMR (CDCl$_3$, 600 MHz) δ 7.17 (1H, dd, *J* = 8.3, 2.1 Hz), 7.15 – 7.07 (2H, m), 7.07 – 7.02 (1H, m), 6.37 (1H, d, *J* = 7.8 Hz), 6.20 (1H, d, *J* = 8.3 Hz), 5.43 (2H, d, *J* = 2.3 Hz), 4.17 (2H, dt, *J* = 10.5, 4.9 Hz), 2.98 (3H, s), 2.94 (3H, s), 2.68 (2H, ddd, *J* = 18.9, 12.8, 5.9 Hz), 2.17 (2H, dd, *J* = 12.8, 11.2 Hz), 1.46 (6H, d, *J* = 7.6 Hz)

^{13}C{^{1}H} NMR (CDCl$_3$, 151 MHz) δ 165.8, 165.4, 150.3, 149.3, 135.4, 133.0, 131.6, 128.9, 125.7, 122.5, 118.3, 107.3, 106.0, 86.7, 86.7, 60.5, 60.3, 60.2, 50.5, 42.8, 42.8, 33.1, 33.0, 25.6, 25.4, 21.2.

MS (APCI): m/z calc. for $C_{26}H_{28}N_4O_2Br+H^+$: 507.4 [M+H]+; found: 507.4

(3*S*,6*S*)-3-((1H-indol-3-yl)methyl)-6-((5-bromo-1H-indol-3-yl)methyl)piperazine-2,5-dione (10)

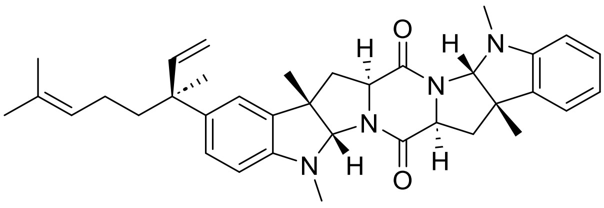

The title compound was prepared from (3*S*,6*S*)-3-((1H-indol-3-yl)methyl)-6-((5-bromo-1H-indol-3-yl)methyl)piperazine-2,5-dione (20 mg, 0.039 mmol) according to the general procedure for asymmetric γ-selective Suzuki-Miyaura coupling using (*Z*)-2-(3,7-dimethylocta-2,6-dien-1-yl)-5,5-dimethyl-1,3,2-dioxaborinane as allylic boronate. The crude product was purified using column chromatography (silica gel; petrol ether:ethyl acetate) to afford the title compound (16.7 mg, 0.029 mmol, 75%) as a colorless amorphous solid. The diastereomeric ratio was determined to be 89:11 (*S*:*R*) concerning the newly generated stereogenic centre, following analysis of the obtained ^{1}H-NMR-spectra comparing to the spectra obtained from a 50:50 mixture of the two diastereomers. The 50:50 diastereomeric mixture was obtained in the same manner as the title compound, with the exception that the general procedure for asymmetric γ-selective Suzuki-Miyaura coupling was performed with SPhos as (achiral) ligand.

^{1}H NMR ($CDCl_3$, 600 MHz) δ 7.09 (1H, td, *J* = 7.7, 1.3 Hz), 7.06 – 7.01 (2H, m), 6.98 (1H, d, *J* = 1.9 Hz), 6.69 (1H, td, *J* = 7.4, 0.9 Hz), 6.34 (1H, d, *J* = 7.8 Hz), 6.27 (1H, d, *J* = 8.2 Hz), 5.96 (1H, dd, *J* = 17.5, 10.3 Hz), 5.44 (1H, s), 5.41 (1H, s), 5.12 – 4.96 (3H, m), 4.14 (2H, dtd, *J* = 13.1, 7.6, 7.1, 3.5 Hz), 2.97 (3H, s), 2.95 (3H, s), 2.70 (2H, dd, *J* = 12.8, 6.0 Hz), 2.16 (2H, ddd, *J* = 12.6, 11.2, 8.3 Hz), 1.89 – 1.68 (4H, m), 1.66 (3H, s), 1.52 (3H, d, *J* = 1.3 Hz), 1.46 (3H, s), 1.45 (3H, s), 1.32 (3H, s)

^{13}C{^{1}H} NMR ($CDCl_3$, 151 MHz) δ 165.9, 165.7, 150.3, 148.3, 147.6, 137.7, 133.1, 132.9, 131.4, 128.9, 126.9, 125.0, 122.5, 120.8, 118.2, 111.4, 106.0, 105.5, 87.1, 86.7, 60.3, 60.3, 50.6, 50.4, 43.9, 42.9, 42.9, 41.4, 33.3, 33.1, 25.8, 25.6, 25.6, 25.1, 23.5, 17.7

HRMS (ESI): m/z calc. for $C_{36}H_{44}N_4O_2+H^+$ 565.3537; found 565.3532

IR (ATR, film) ṽ [cm^{-1}] =1964, 2925, 2860, 1668, 162, 1492, 1417, 1301, 1208, 1083, 995, 914, 810, 744.

$[\alpha]_D^{20} = -192.0$ (c = 0.1, CH_3CN)

m.p.: 141 °C - 145 °C

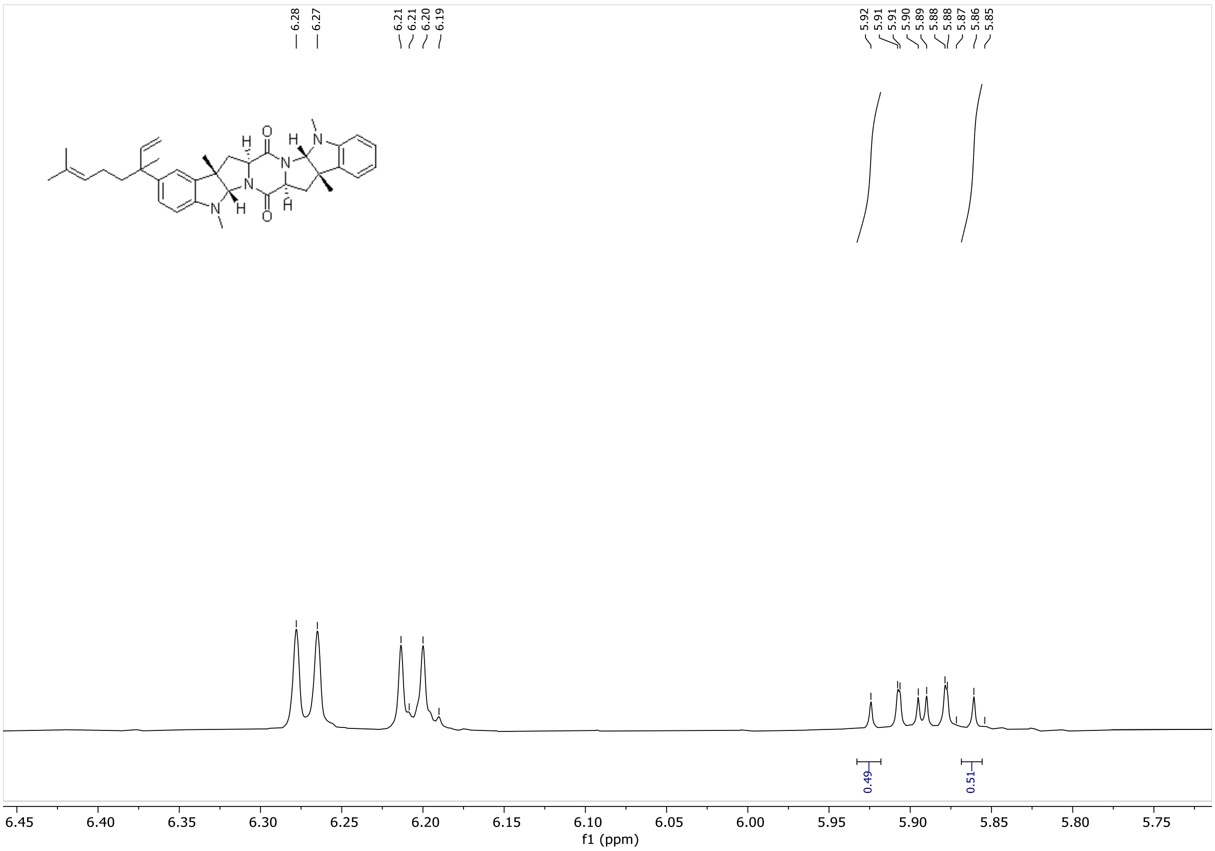

*Figure S5 Cutout from the ^{1}H-NMR of a 50:50 diastereomeric mixture of Compound **10**.*

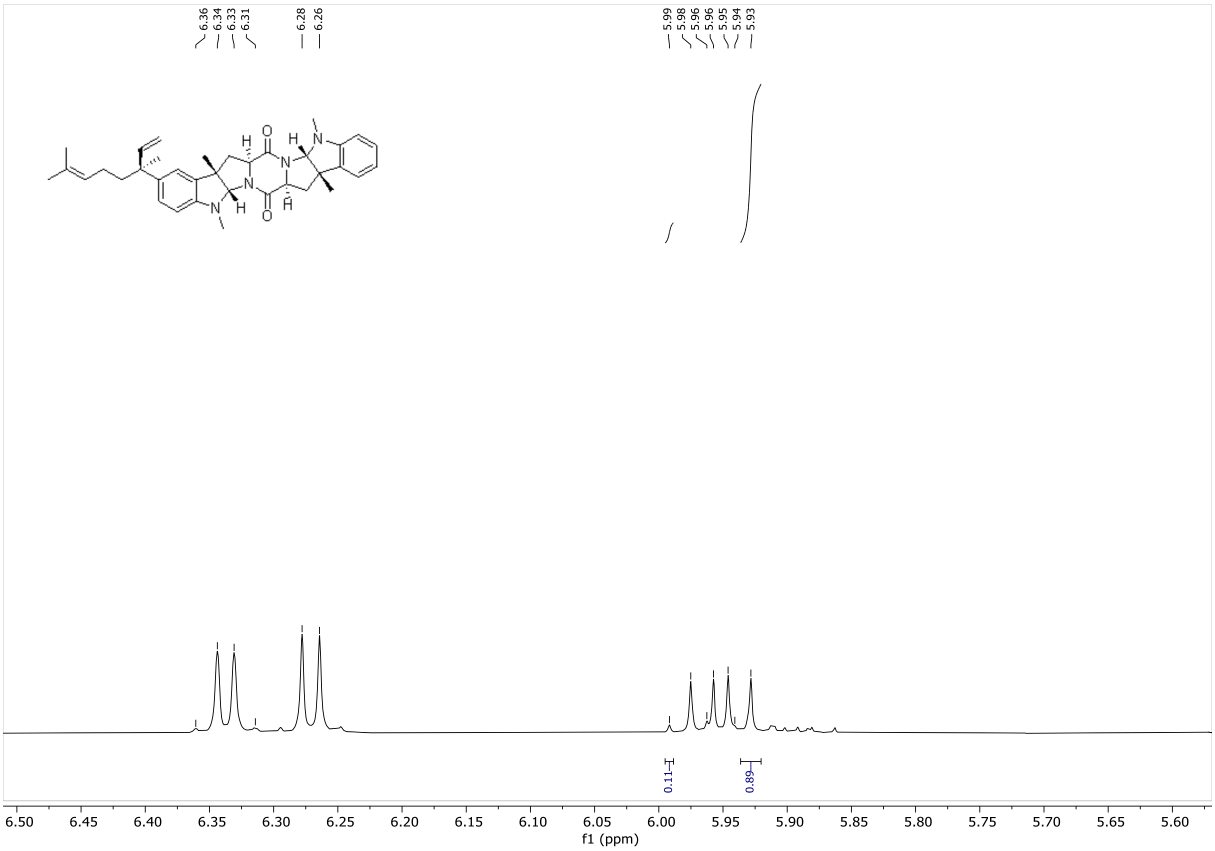

*Figure S6 Cutout from the ^{1}H-NMR of diastereomerically enriched Compound **10**.*

6. Biological work

For completeness, the following work (section 6 Biological work) has already been performed and published[9]:

Protein sequences

The gene sequence encoding the methyltransferase SgMT (GenBank: GGT26788.1) and the tryptophan synthase β-subunit Pf0A9 (mutant yielded via directed evolution of the native parental enzyme TrpB, GenBank: AB080770.1) were optimized for codon usage and synthetically synthesized within a pET28a (SgMT) or pET21a (TrpBPf0A9) expression vector by GenScript (USA).

C-terminal His-tagged SgMT:

(Code adapted from *Haase et al*.[10])

Nucleotide sequence:

ATGGGCCATATGTCGAGCCAGACCGTTACCCCGGATCCGTATGGTAACCTGGCAGAAAGCTATGAT
CGTCTGGCACAGTGGGCAATTGATCAGCAGCAGGAGAGCCCCCGTGATCGTGTTGGGGACTTTC
TGCAGACCTTTTGGCAGAGCCAGGATCGTCCGGTTAGGACCGTTCTGGAAATTTGTTGTGGTACC
GGTCTGATGCTGGCAGAACTGGCTCGTCGTGGGTATGTTGTTACCGGGCTGGATCGTTCGGCAG
CAATGCTGGAGCAGGCTCGTGCACGTATGGGTGGAAAAACCACTCTGATTAGGGCAGAGCTGCC
GGATATTCCGGCACCGGCAGGGGAATTTGATGCAGTTGTTAGCGCAGCAGGGGGACTGAATTATC
TGAGCGAATCGCAGATTAGCGCAACCTTTGGTGCAGTTGCAAGGCTGCTGCCGGCAGGAGGTAC
CTTTACCTTTGATGTTTTTGGTCAGGGGTTTTATGCAAAATTTTTTGATCCGTCGGCACCGCGTGTT
ATGGCACTGGAGCTGGATGATATTAGCTATATTTGGACCTTTACCAAACCGGCAGAAGCACCGTTT
GTTGATATGAGCTATACCCAGTTTAGCCCGGCATCGCGTGCAGTTGATGGGGAACCCGCATTTATT
CGTACCAGGGATCTGCACCGTTATTATCCGCTGCCGCATGCAACCGTTCTGCGTCTGGCAGCAGA
ACATGGGTTTACCGATGCACGTGCACATGATAATTATAGCAGCGATCCGAGCGGTCCGCATACCCT
GTATGATACCTGGACTATGGTTCGTACCGGATCGCTCGAGCACCACCACCACCACCACTGA

Protein sequence:

MGHMSSQTVTPDPYGNLAESYDRLAQWAIDQQQESPRDRVGDFLQTFWQSQDRPVRTVLEICCGT
GLMLAELARRGYVVTGLDRSAAMLEQARARMGGKTTLIRAELPDIPAPAGEFDAVVSAAGGLNYLSES
QISATFGAVARLLPAGGTFTFDVFGQGFYAKFFDPSAPRVMALELDDISYIWTFTKPAEAPFVDMSYTQ
FSPASRAVDGEPAFIRTRDLHRYYPLPHATVLRLAAEHGFTDARAHDNYSSDPSGPHTLYDTWTMVRT
GSLEHHHHHH*

C-terminal His-tagged TrpBPf0A9

(Code adapted from *Dick et al.*[11])

Nucleotide sequence:

ATGTGGTTCGGTGAATTTGGTGGTCAGTACGTGCCAGAAACGCTGGTTGGACCCCTGAAAGAGCT
GGAAAAAGCTTACAAACGTTTCAAAGATGACGAAGAATTCAATCGTCAGCTGAATTACTACCTGAAA
ACCTGGGCAGGTCGTCCAACCCCACTGTACTACGCAAAACGCCTGACTGAAAAAATCGGTGGTG
CTAAAGTCTACCTGAAACGTGAAGACCTGGTTCACGGTGGTGCACACAAGACCAACAACGCCATC
GGTCAGGCACTGCTGGCAAAGCTCATGGGTAAAACTCGTCTGATCGCTGAGACCGGTGCTGGTC
AGCACGGCGTAGCGACTGCAATGGCTGGTGCACTGCTGGGCATGAAAGTGGACATTTACATGGG
TGCTGAGGACGTAGAACGTCAGAAACTGAACGTATTCCGTATGAAGCTGCTGGGTGCAAACGTAA
TTCCAGTTAACTCCGGTTCTCGCACCCTGAAAGACGCATTTGACGAGGCTCTGCGTGATTGGGTG
GCTACTTTTGAATACACCCACTACCTAATCGGTTCCGTGGTCGGTCCACATCCGTATCCGACCATC
GTTCGTGATTTTCAGTCTGTTATCGGTCGTGAGGCTAAAGCGCAGATCCTGGAGGCTGAGGGTCA
GCTGCCAGATGTAATCGTTGCTTGTGTTGGTGGTGGCTCTAACGCGATGGGTATCTTTTACCCGTT
CGTGAACGACAAAAAAGTTAAGCTGGTTGGCGTTGAGGCTGGTGGTAAAGGCCTGGAATCTGGTA
AGCATTCCGCTAGCCTGAACGCAGGTCAGGTTGGTGTGTCCCATGGCATGCTGTCCTACTTTCTG
CAGGACGAAGAAGGTCAGATCAAACCAAGCCACTCCATCGCACCAGGTCTGGATCATCCAGGTGT
TGGTCCAGAACACGCTTACCTGAAAAAAATTCAGCGTGCTGAATACGTGGCTGTAACCGATGAAGA
AGCACTGAAAGCGTTCCATGAACTGAGCCGTACCGAAGGTATCATCCCAGCTCTGGAATCTGCGC
ATGCTGTGGCTTACGCTATGAAACTGGCTAAGGAAATGTCTCGTGATGAGATCATCATCGTAAACCT
GTCTGGTCGTGGTGACAAAGACCTGGATATTGTCCTGAAAGCGTCTGGCAACGTGCTCGAGCAC
CACCACCACCACCACTGAG

Protein sequence:

MWFGEFGGQYVPETLVGPLKELEKAYKRFKDDEEFNRQLNYYLKTWAGRPTPLYYAKRLTEKIGGAK
VYLKREDLVHGGAHKTNNAIGQALLAKLMGKTRLIAETGAGQHGVATAMAGALLGMKVDIYMGAEDVE
RQKLNVFRMKLLGANVIPVNSGSRTLKDAFDEALRDWVATFEYTHYLIGSVVGPHPYPTIVRDFQSVIG
REAKAQILEAEGQLPDVIVACVGGGSNAMGIFYPFVNDKKVKLVGVEAGGKGLESGKHSASLNAGQV
GVSHGMLYFLQDEEGQIKPSHSIAPGLDHPGVGPEHAYLKKIQRAEYVAVTDEEALKAFHELSRTEGII
PALESAHAVAYAMKLAKEMSRDEIIIVNLSGRGDKDLDIVLKASGNVLEHHHHHH*

Bacterial strains and media

The cultivation and protein expression were carried out in E. *coli* BL21(DE3). For the preculture, LB liquid medium (lysogeny broth) containing 10 g/L tryptone, 5 g/L yeast extract, and 2 g/L sodium chloride was used. The main culture was grown in TB liquid medium (terrific broth; Carl Roth, Karlsruhe, Germany), which included 12 g/L casein, 24 g/L yeast extract, 12.54 g/L K_2HPO_4, 2.3 g/L KH_2PO_4, and 4 mL/L glycerol, supplemented with kanamycin (SgMT) or ampicillin (TrpB^{Pf0A9}) at a final concentration of 100 µg/mL. All culture media were prepared using distilled water and sterilized by autoclaving prior to use.

Protein expression

Competent E. *coli* BL21(DE3) cells were transformed with the desired plasmid by heat shock: 100 ng of plasmid DNA was added to 100 µL of competent cells and incubated on ice for 30 minutes. The cells were then heat-shocked in a 42 °C water bath for 90 seconds, followed by the addition of 700 µL of LB medium. The mixture was shaken at 37 °C for 1 hour. Afterward, the cells were centrifuged at 2000 rpm for 2 minutes, and the resulting pellet was resuspended in 100 µL of LB medium before plating on LB agar plates with kanamycin (SgMT) or ampicillin (TrpB^{Pf0A9}). The plates were incubated overnight at 37 °C. To prepare a preculture, a single colony from the transformed cells was inoculated into 5 mL of LB medium containing kanamycin (SgMT) or ampicillin (TrpB^{Pf0A9}) and incubated at 37 °C for 16 hours. This preculture was then used to inoculate the main culture (500 mL of TB medium) at a 1:100 dilution. The main culture was incubated at 37 °C with shaking at 130 rpm until the optical density at 600 nm (OD600) reached 0.5. At this point, protein expression was induced by adding IPTG to a final concentration of 100 µM. The culture was then incubated at 25 °C for 20 hours. Cells were harvested by centrifugation at 7000 g for 35 minutes at 4 °C.

7. Copy of NMR spectra

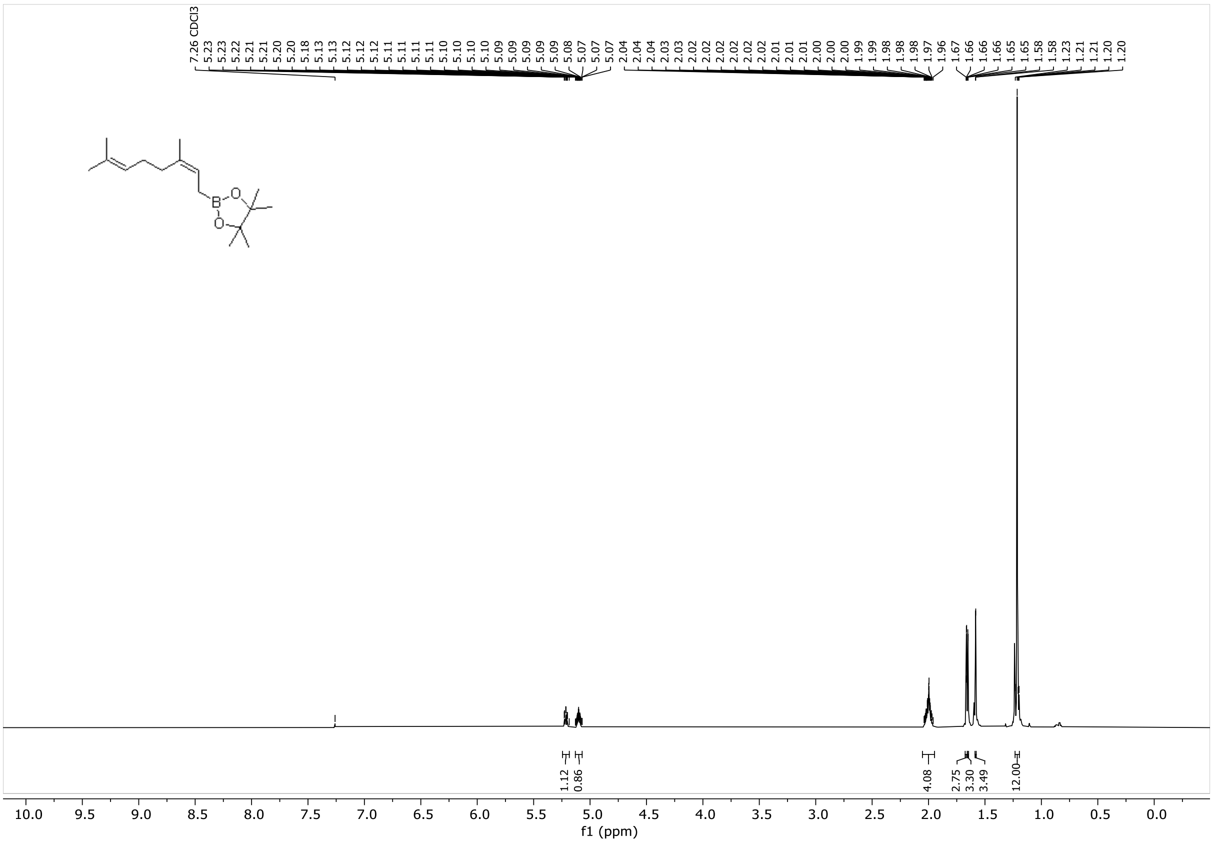

*Figure S7 ^{1}H-NMR(600 MHz) of **B1** in CDCl3.*

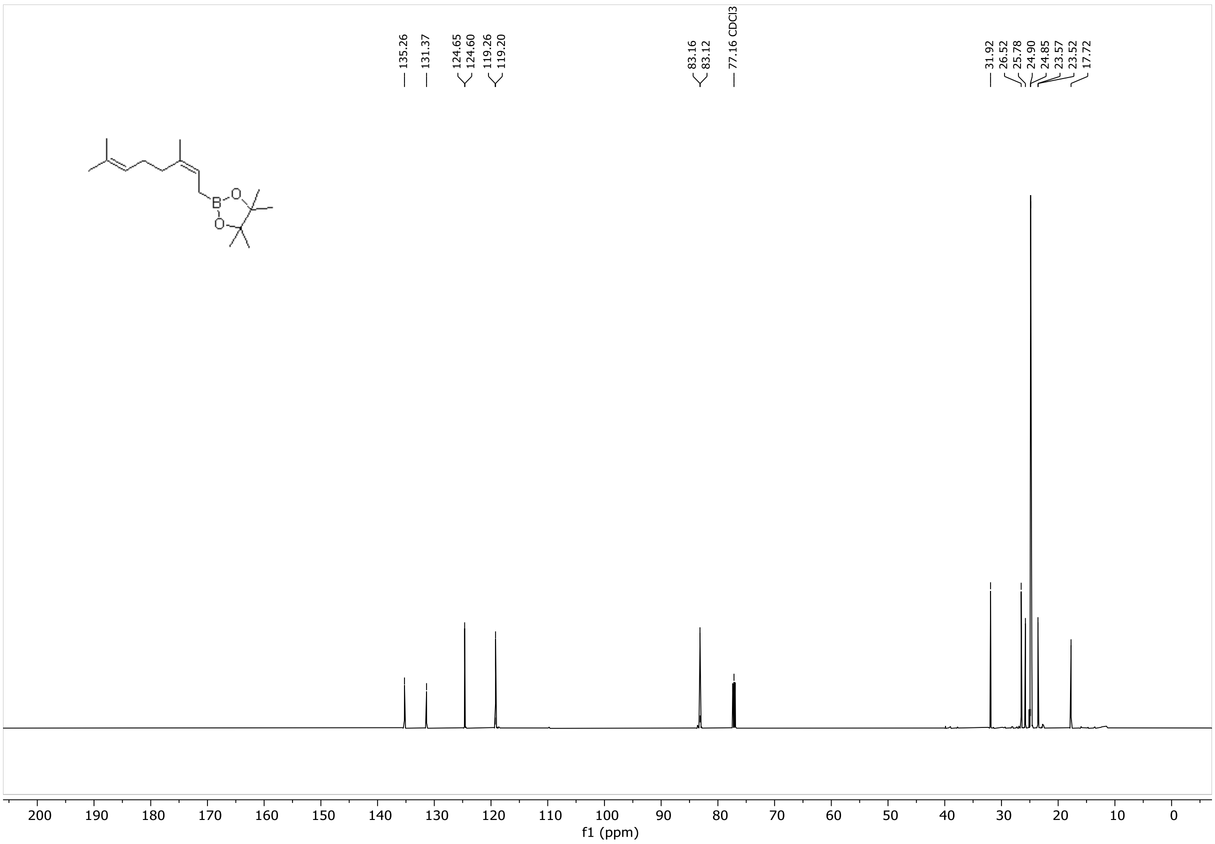

Figure S8 $^{13}C\{^{1}H\}$*-NMR(151 MHz) of* ***B1*** *in* $CDCl_3$.

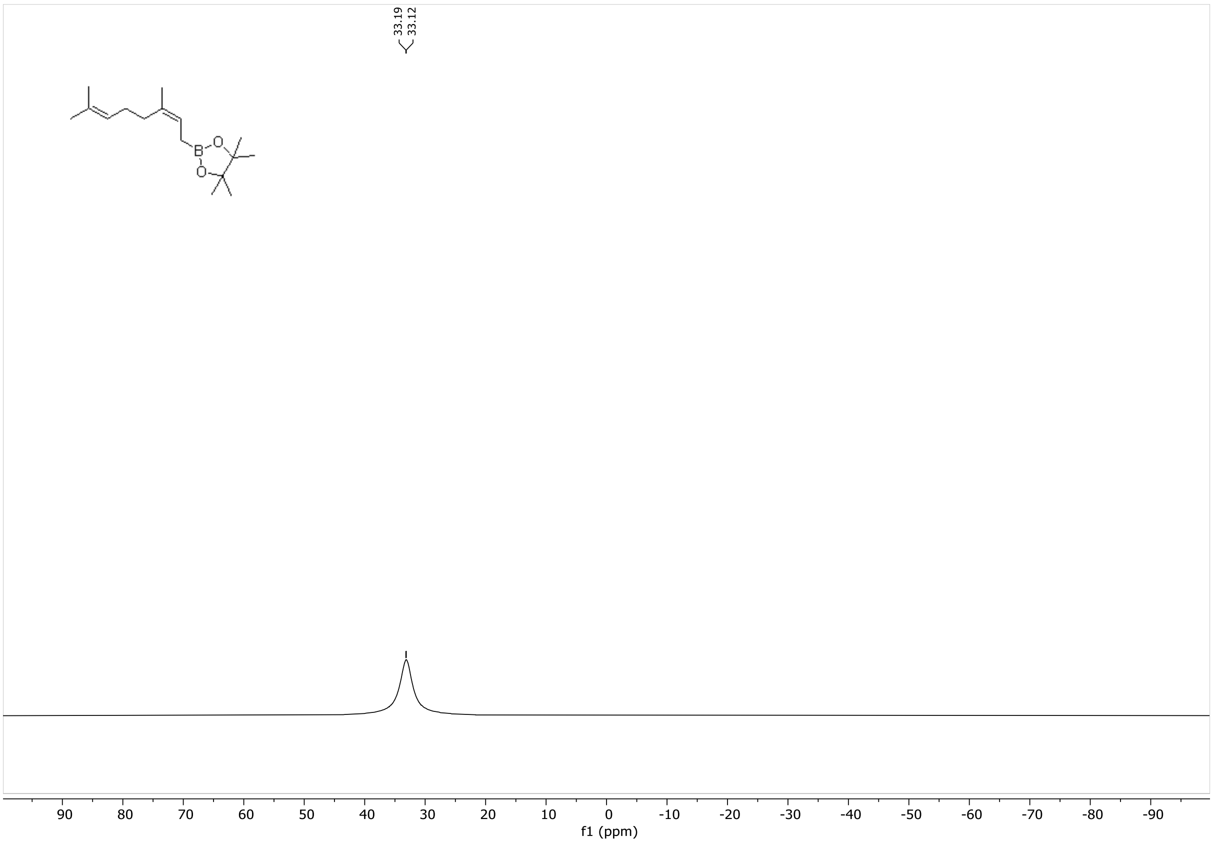

Figure S9 ^{11}B*-NMR(96 MHz) of* ***B1*** *in* $CDCl_3$.

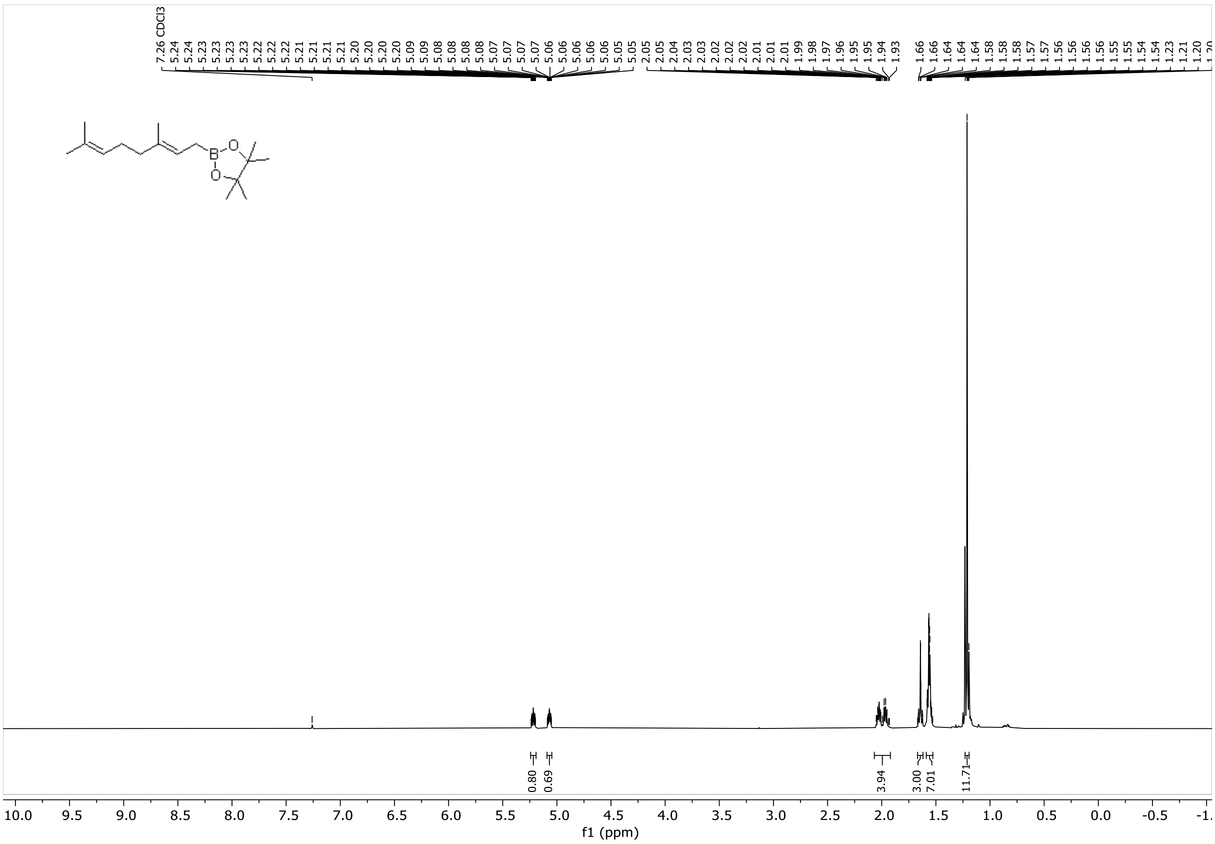

*Figure S10 ^{1}H-NMR(600 MHz) of **B2** in $CDCl_3$.*

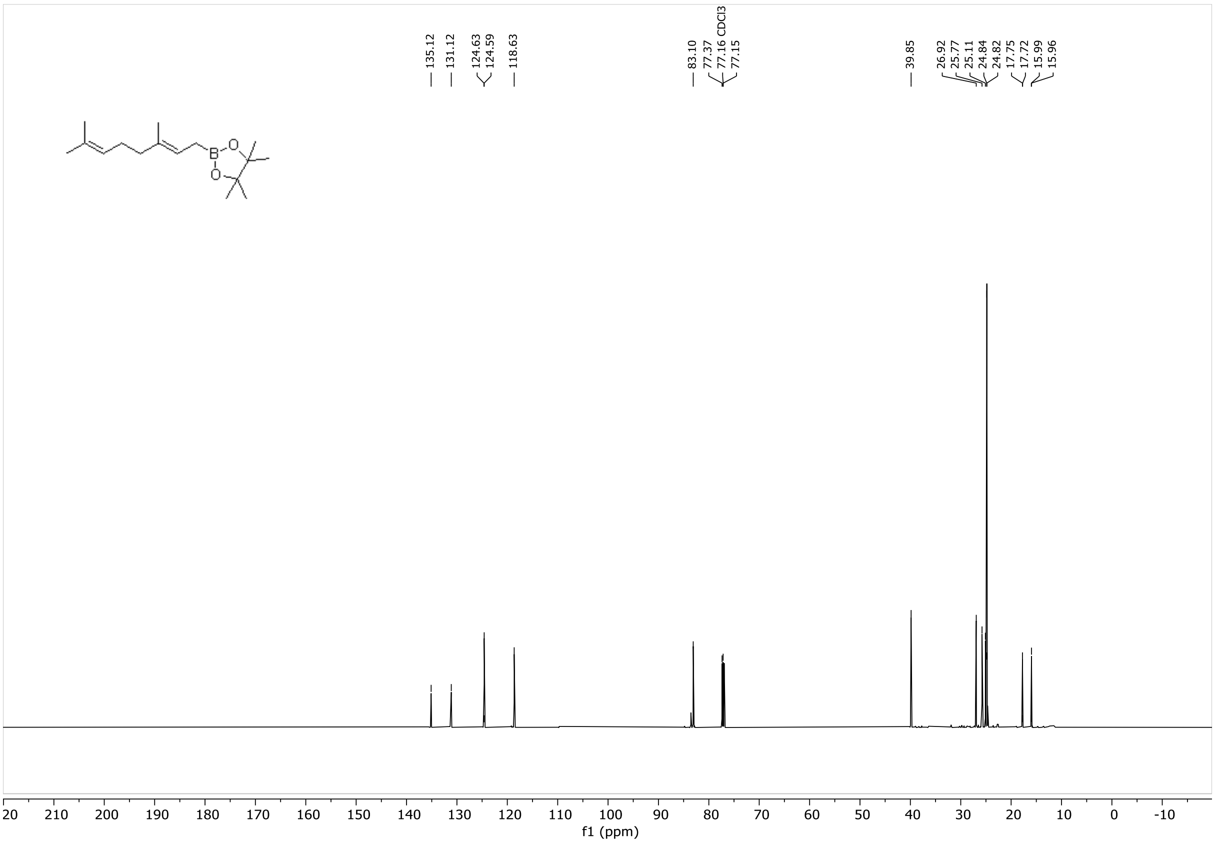

*Figure S11 $^{13}C\{^{1}H\}$-NMR(151 MHz) of **B2** in $CDCl_3$.*

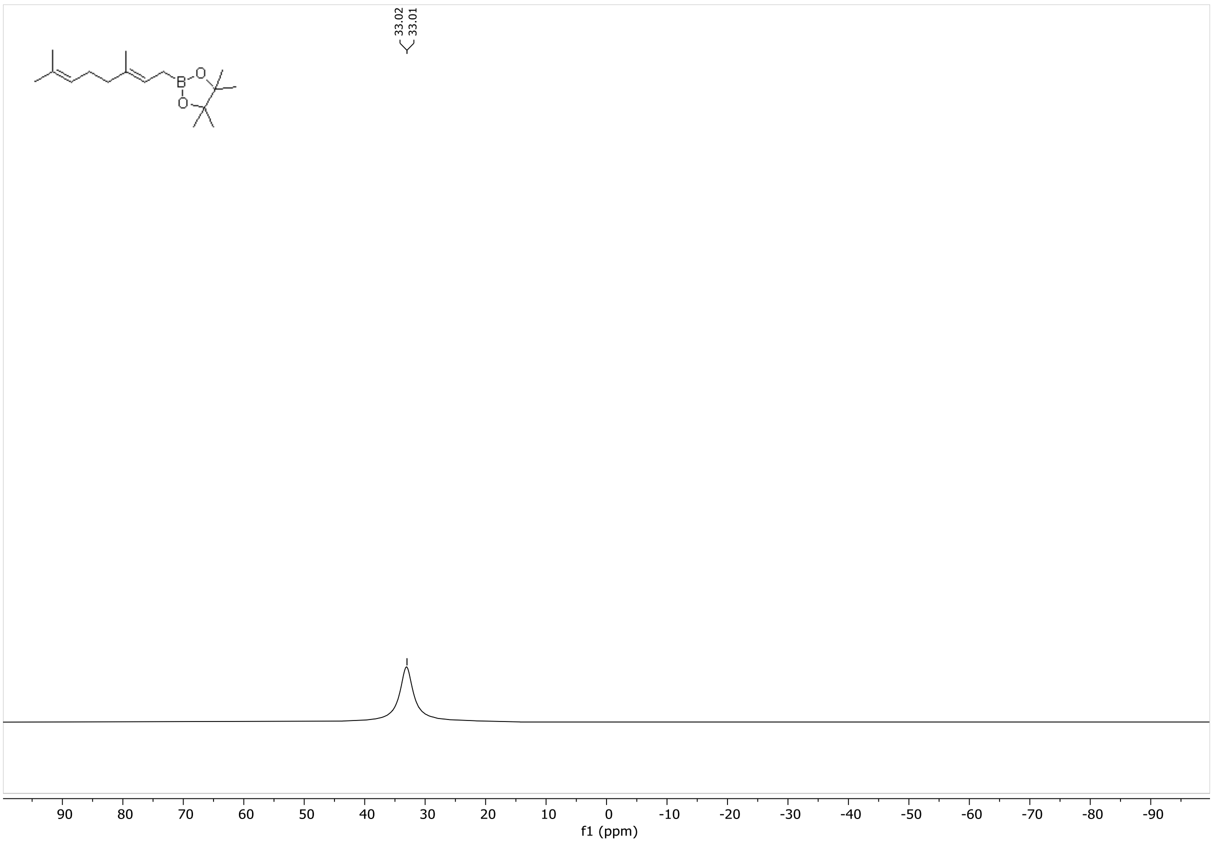

*Figure S12 ^{11}B-NMR(96 MHz) of **B2** in $CDCl_3$.*

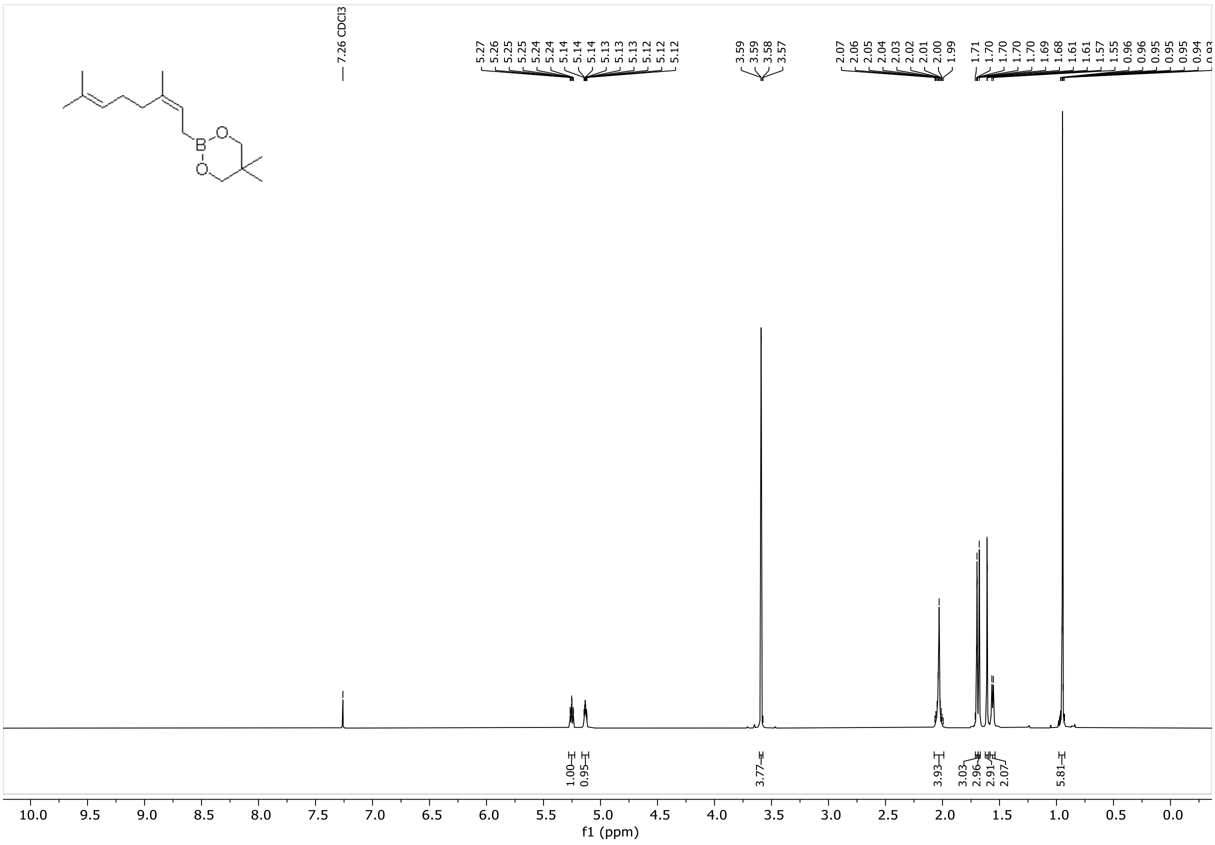

*Figure S13 ^{1}H-NMR(600 MHz) of **B3** in $CDCl_3$.*

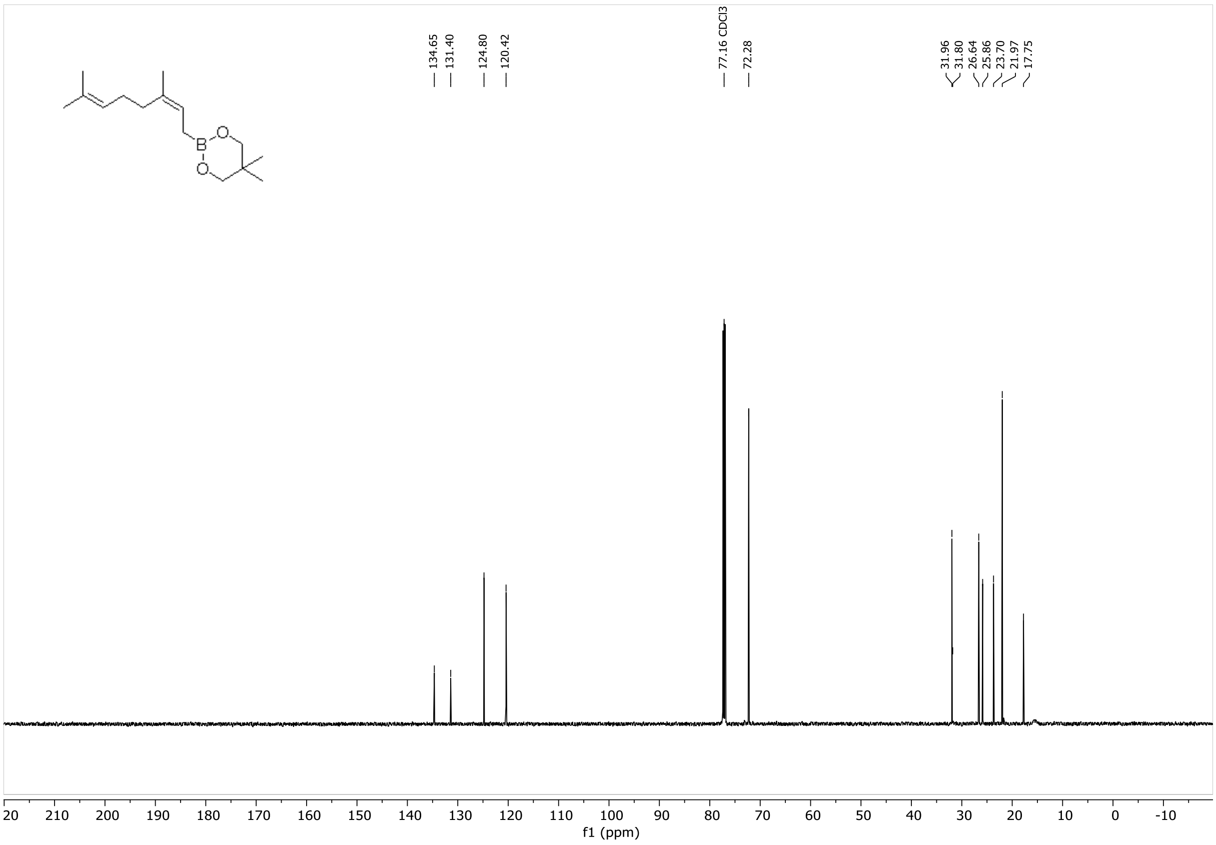

*Figure S14 $^{13}C\{^{1}H\}$-NMR(151 MHz) of **B3** in $CDCl_3$.*

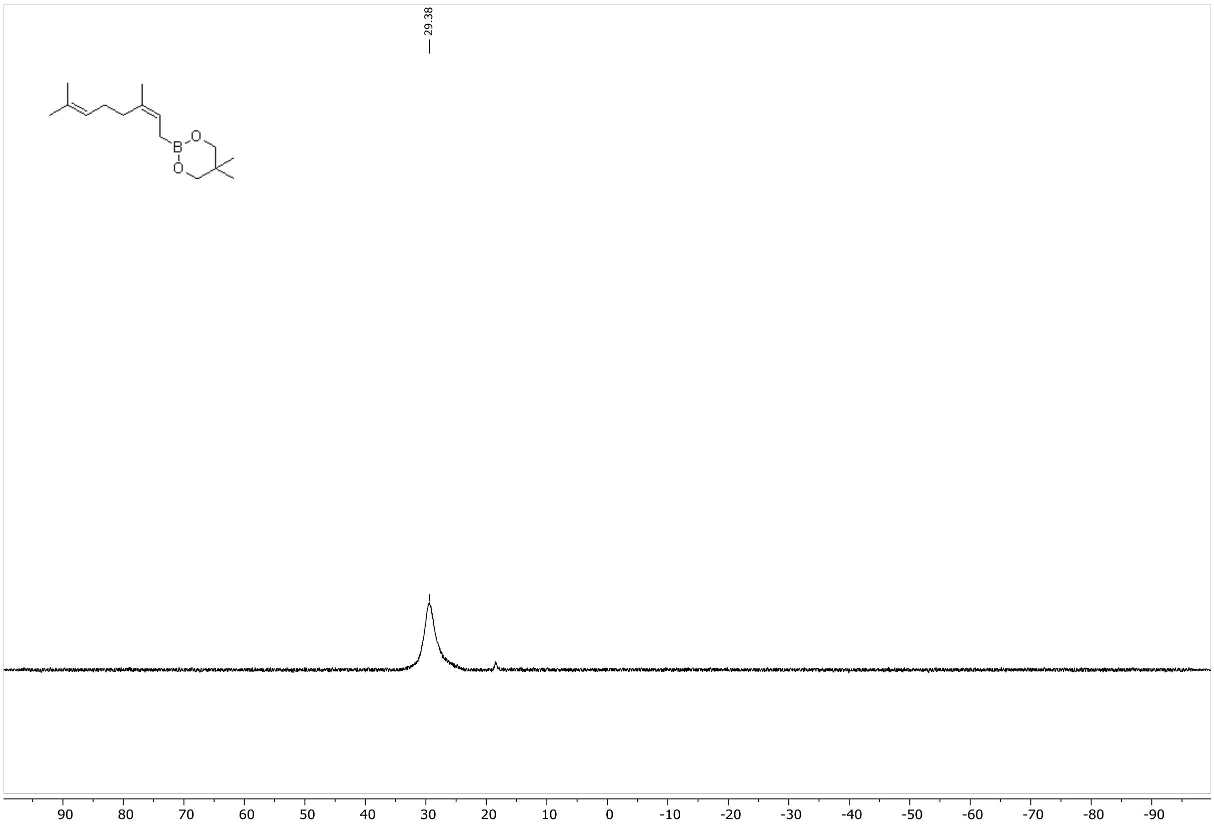

*Figure S15 ^{11}B-NMR(96 MHz) of **B3** in $CDCl_3$.*

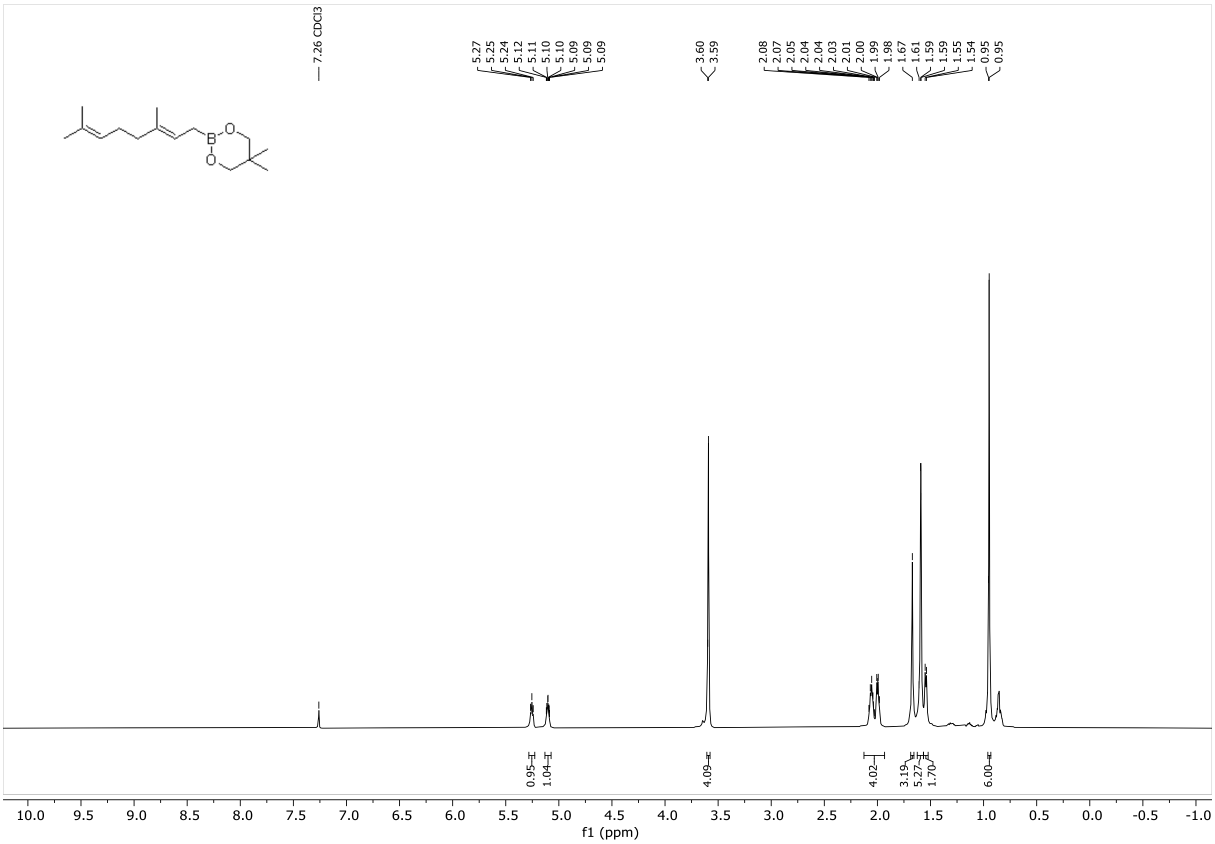

*Figure S16 ^{1}H-NMR(600 MHz) of **B4** in $CDCl_3$.*

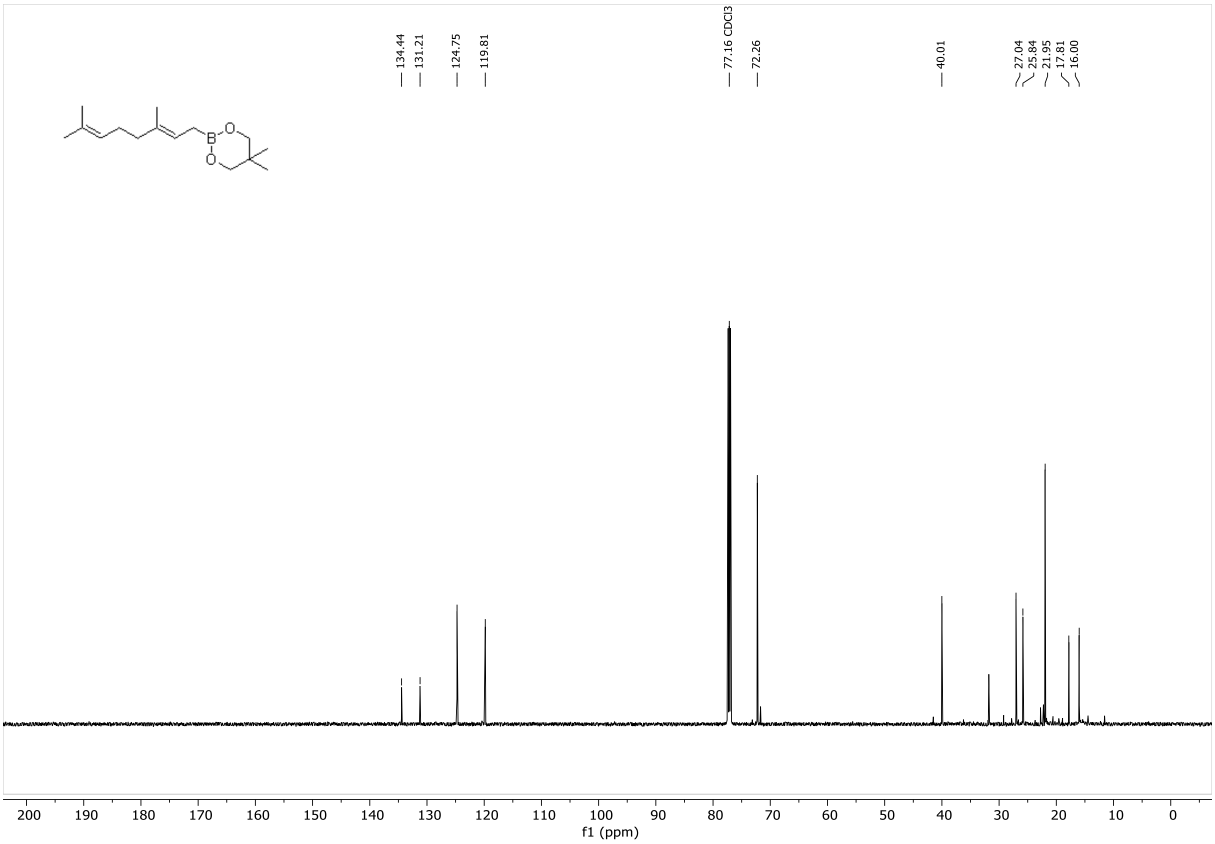

*Figure S17 $^{13}C\{^{1}H\}$-NMR(151 MHz) of **B4** in $CDCl_3$.*

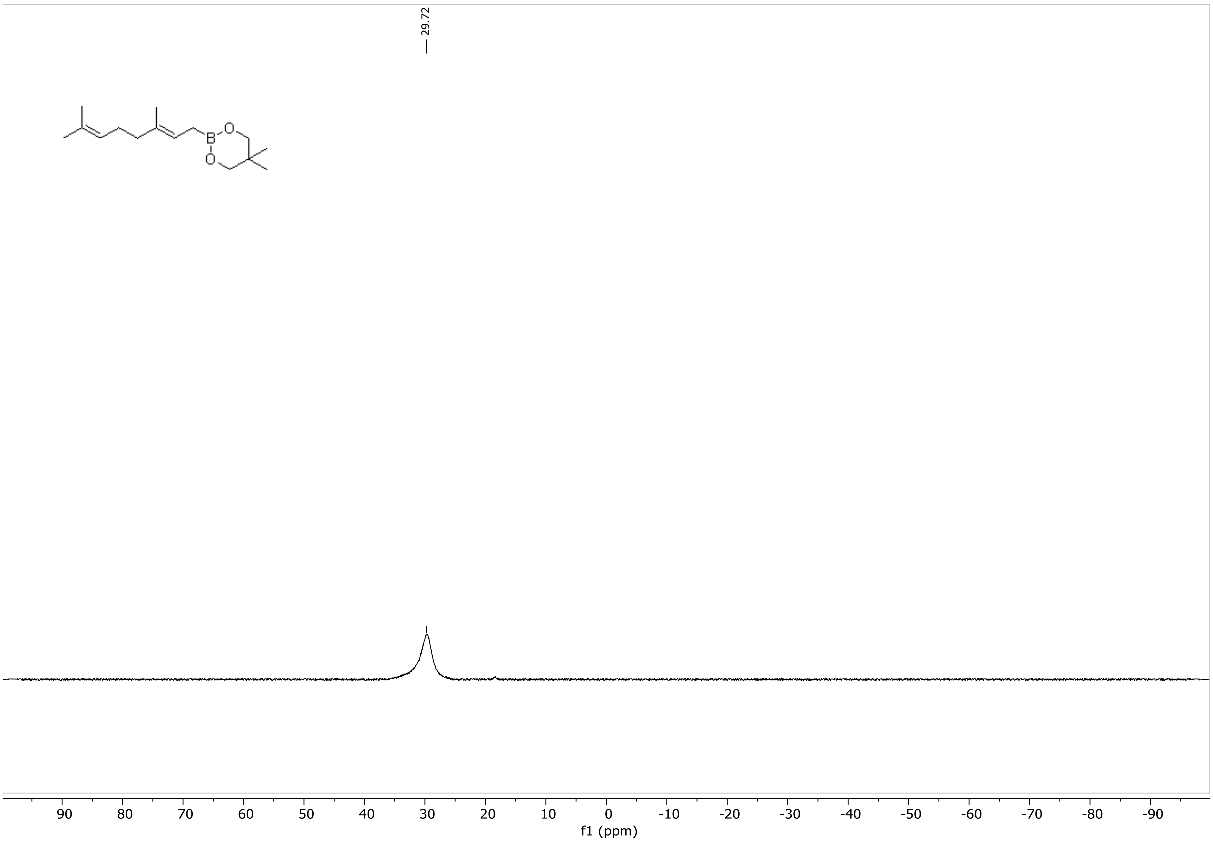

*Figure S18 ^{11}B-NMR(96 MHz) of **B4** in $CDCl_3$.*

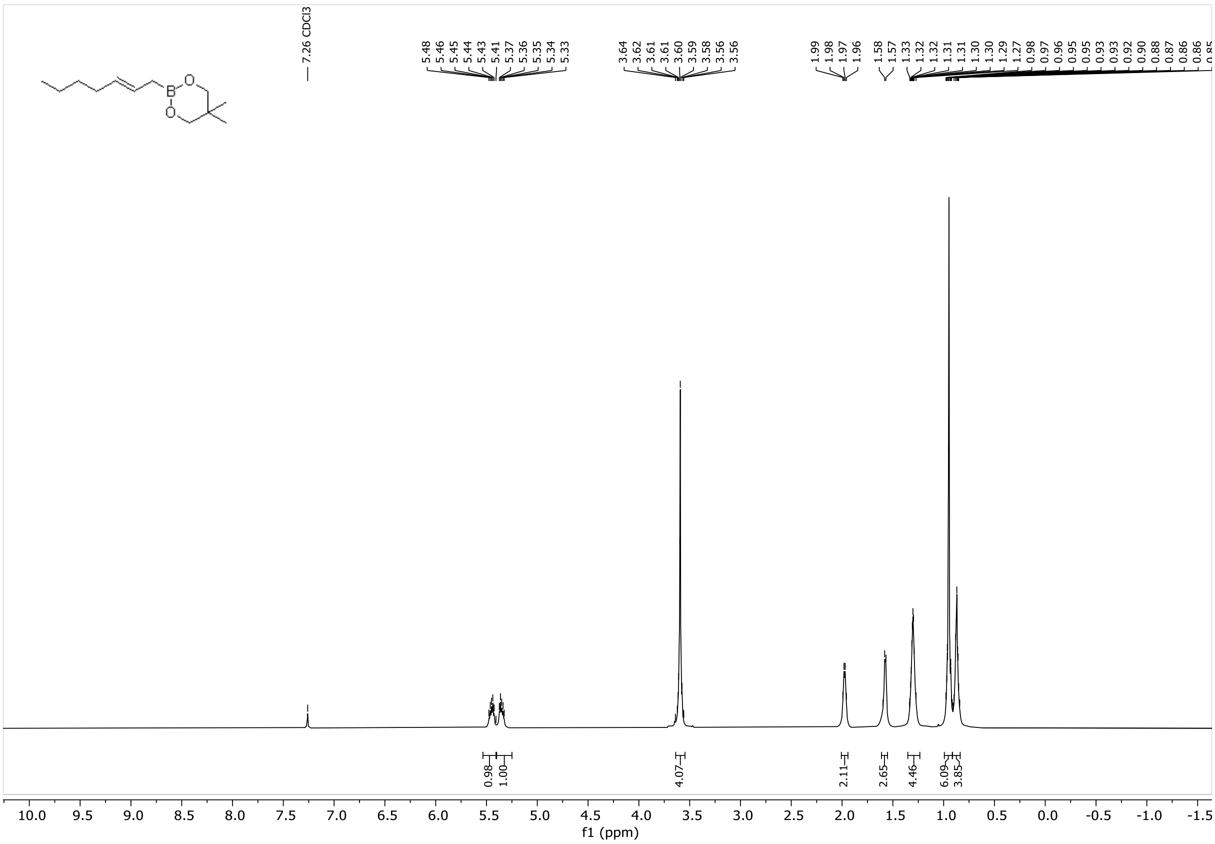

*Figure S19 ^{1}H-NMR(600 MHz) of **B5** in $CDCl_3$.*

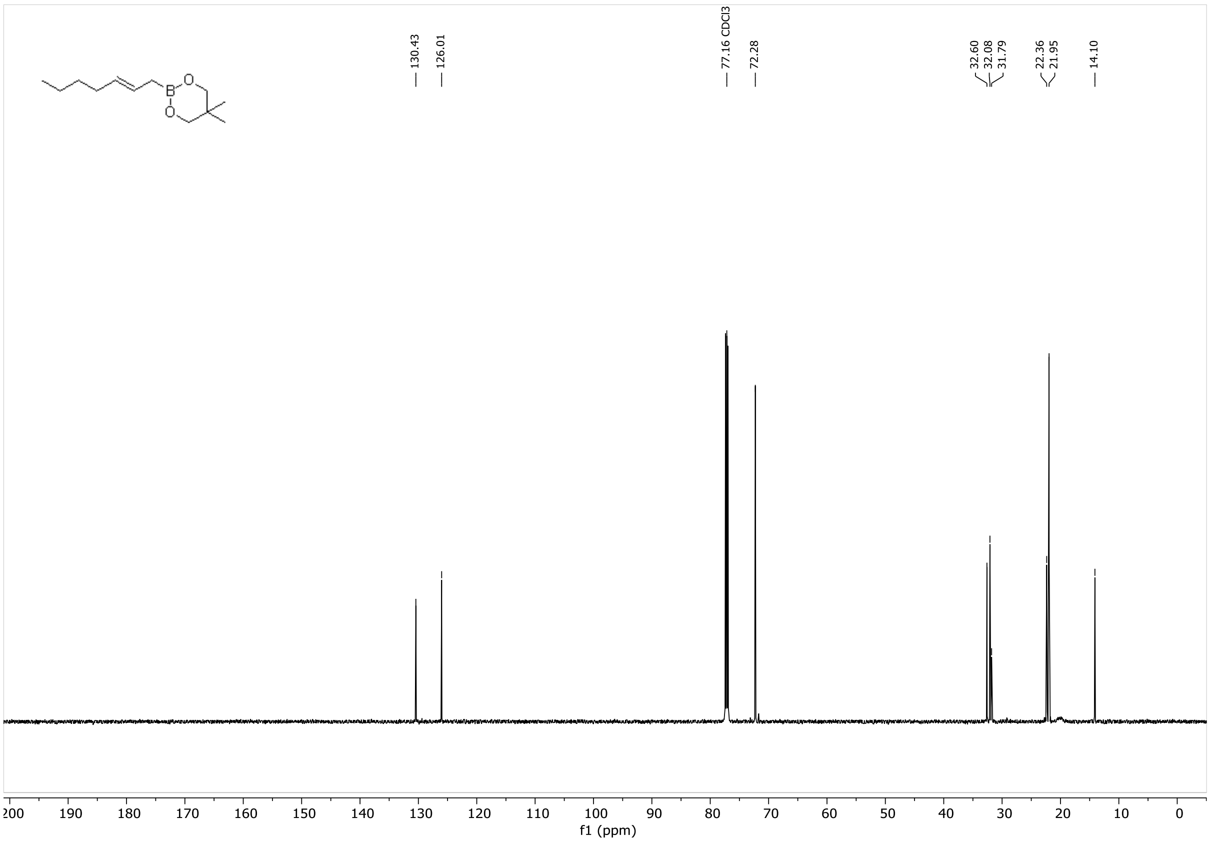

Figure S20 $^{13}C\{^{1}H\}$*-NMR(151 MHz) of* ***B5*** *in* $CDCl_3$*.*

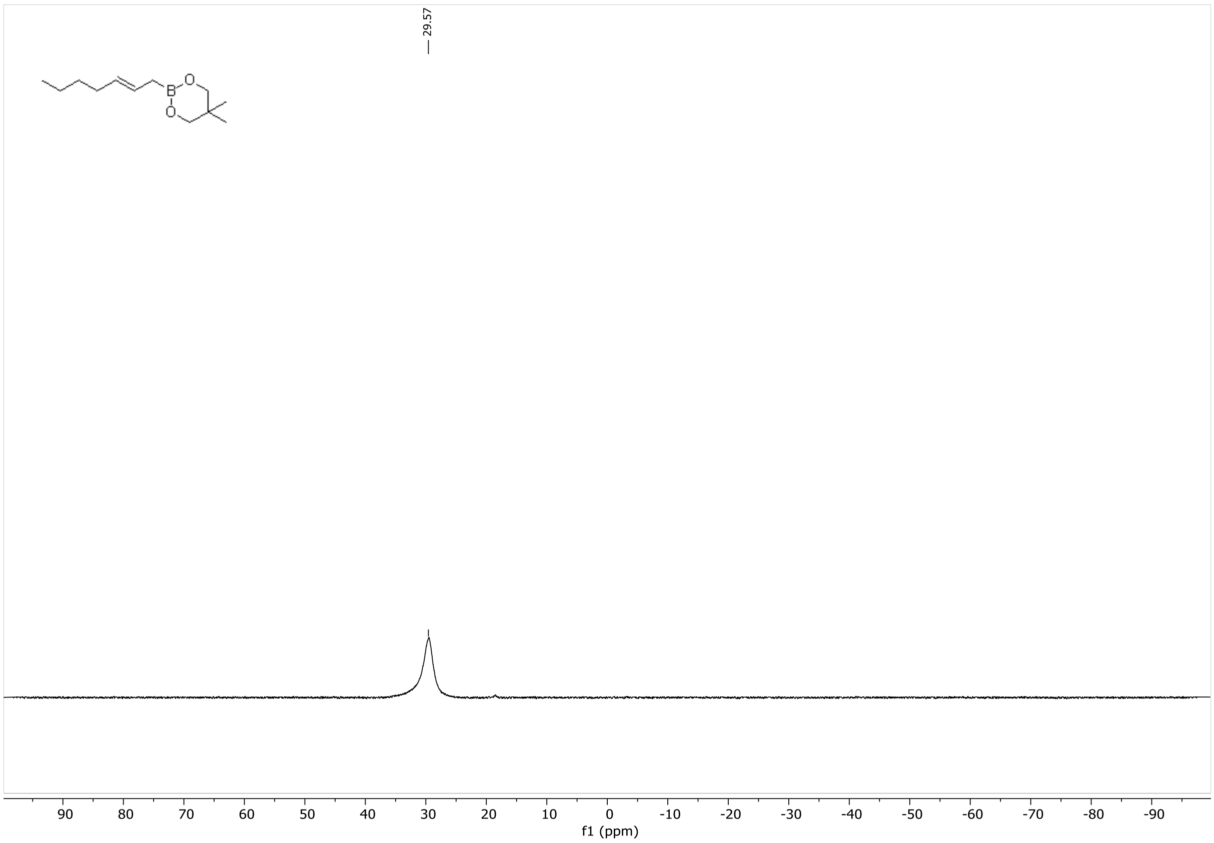

Figure S21 $^{13}C\{^{1}H\}$*-NMR(96 MHz) of* ***B5*** *in* $CDCl_3$*.*

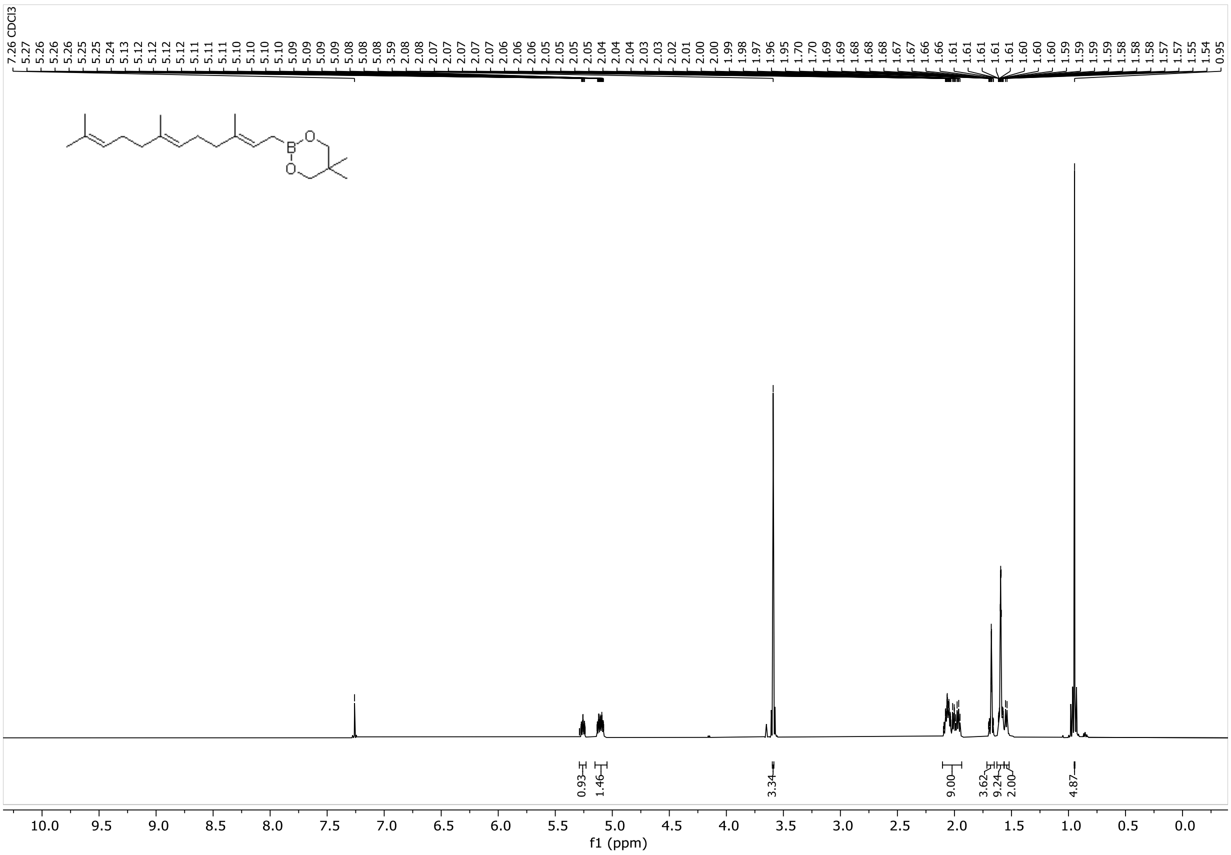

*Figure S22 ¹H-NMR(600 MHz) of **B6** in $CDCl_3$.*

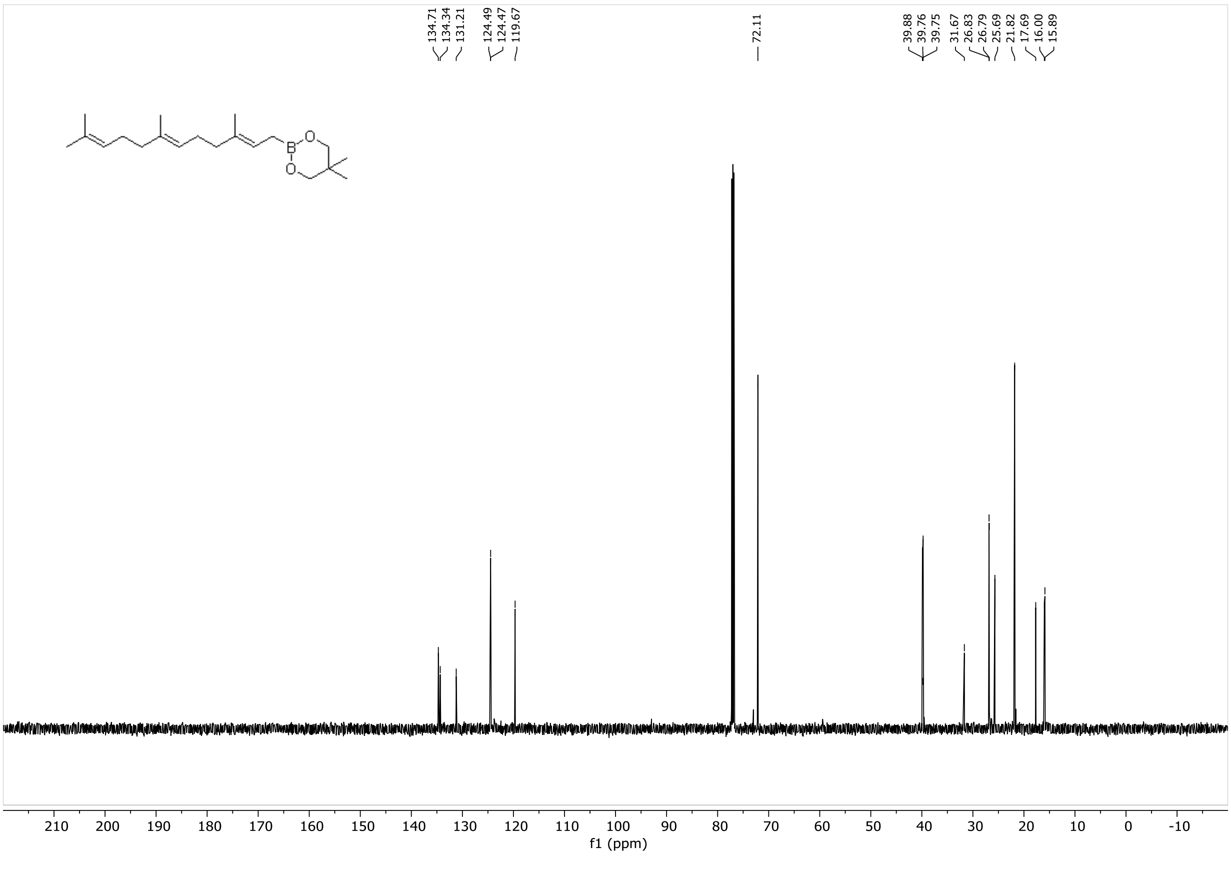

*Figure S23 $^{13}C\{^1H\}$-NMR(151 MHz) of **B6** in $CDCl_3$.*

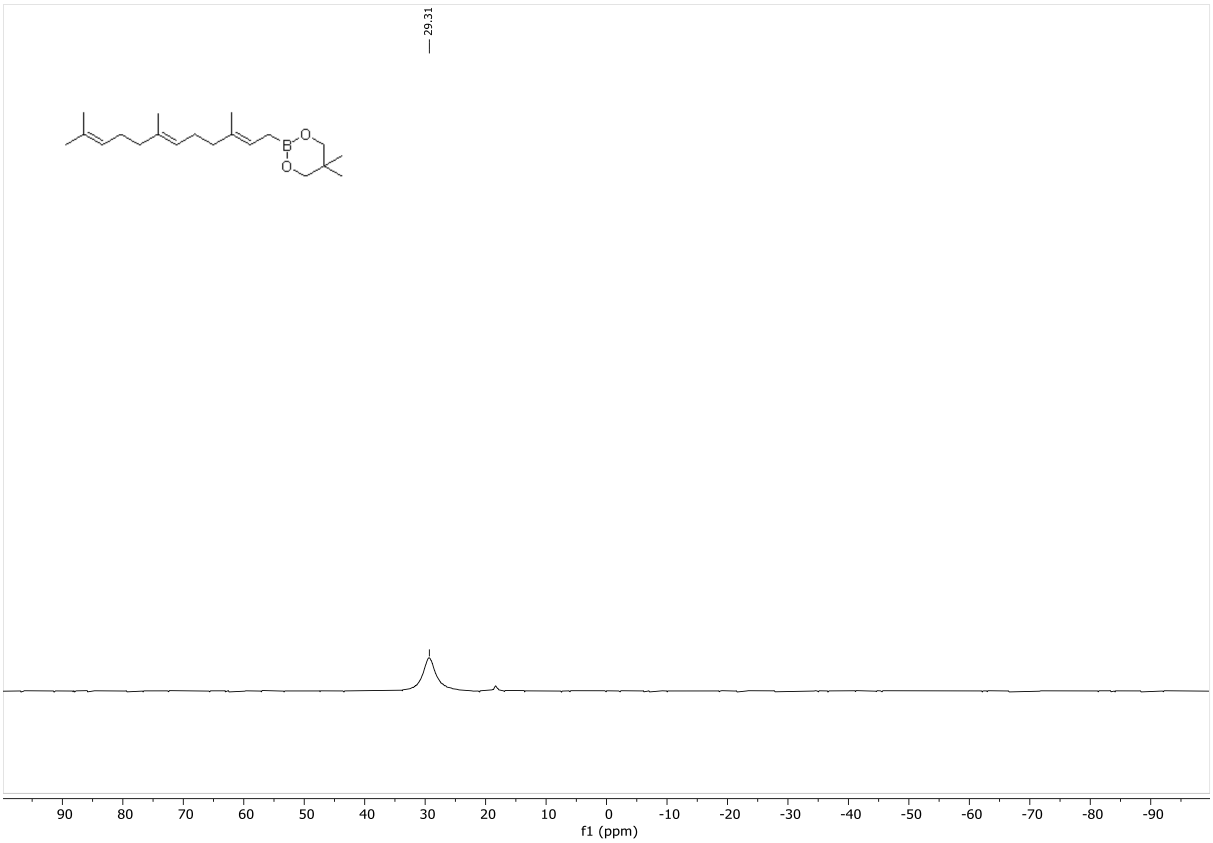

*Figure S24 ^{11}B-NMR(96 MHz) of **B6** in $CDCl_3$.*

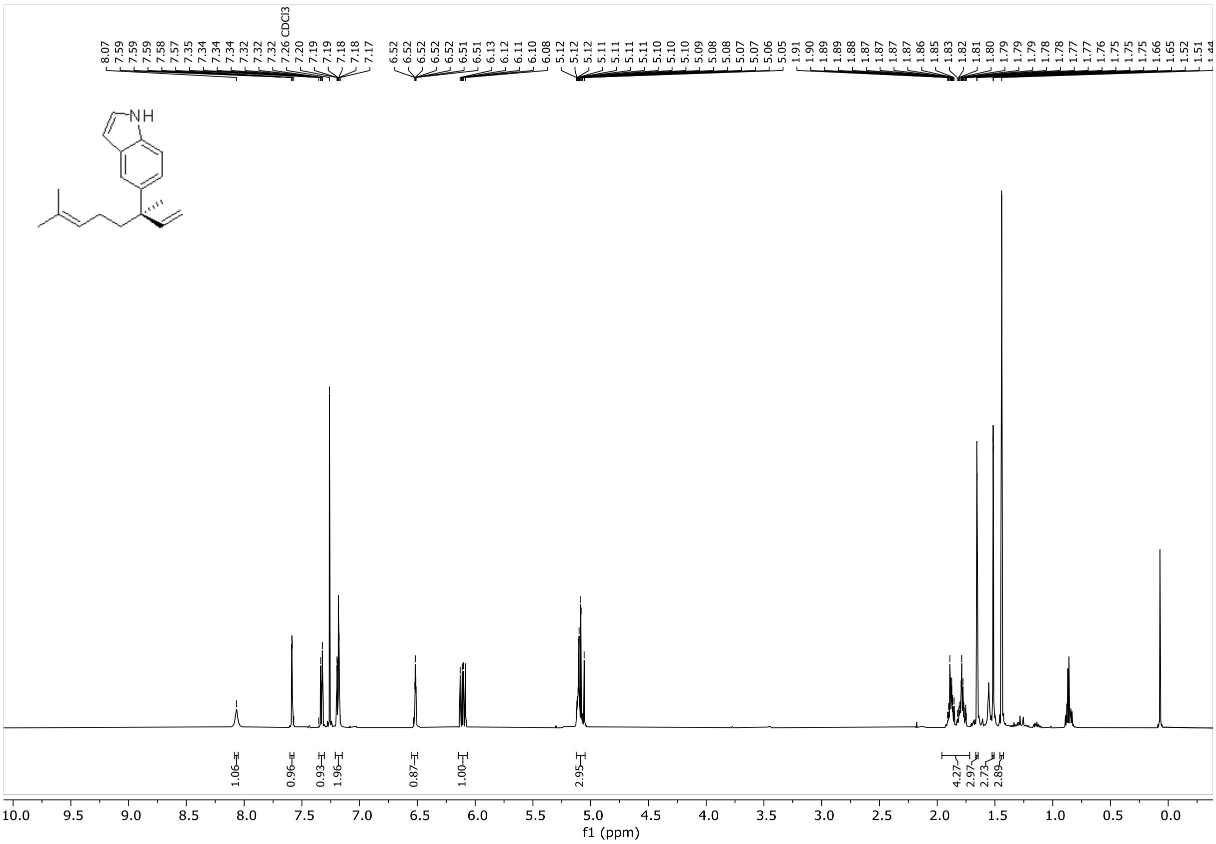

*Figure S25 ^{1}H-NMR(600 MHz) of **5a** in $CDCl_3$.*

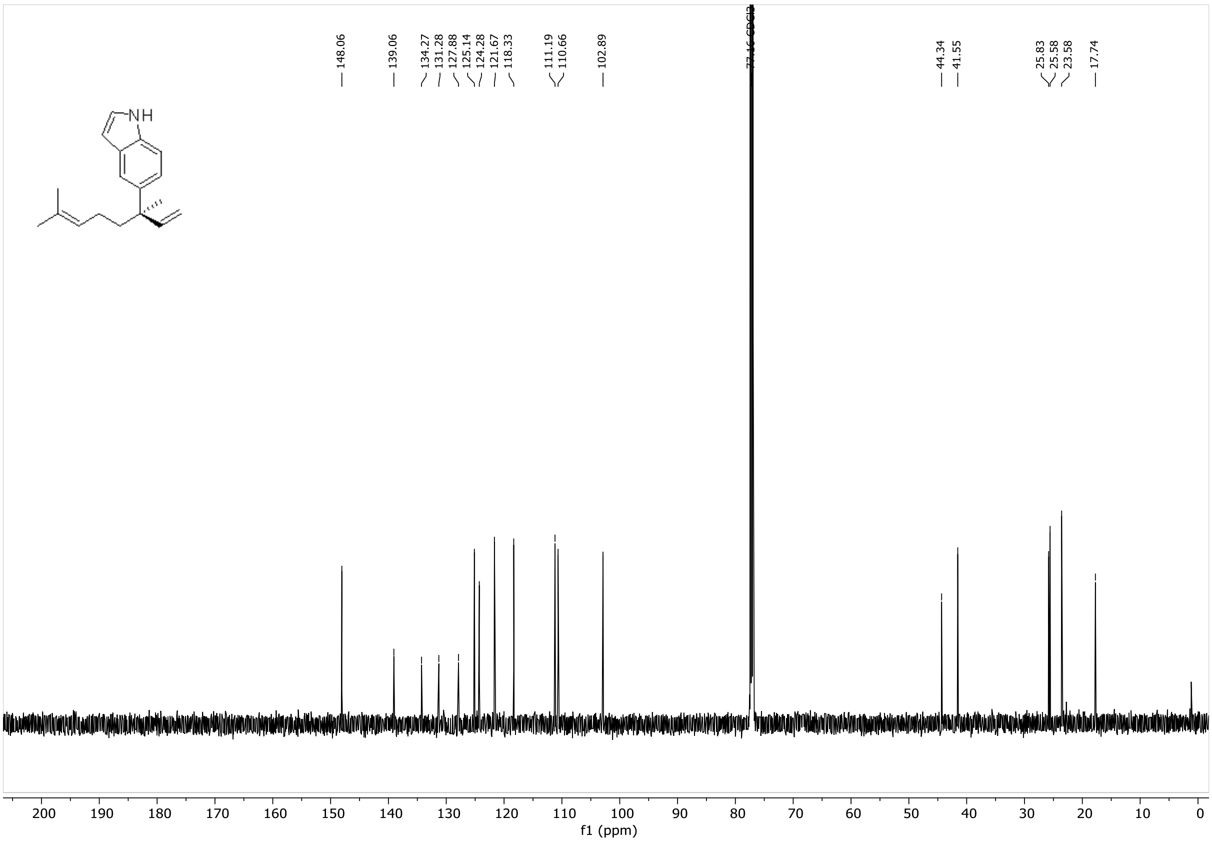

*Figure S26 $^{13}C\{^{1}H\}$-NMR(151 MHz) of **5a** in $CDCl_3$.*

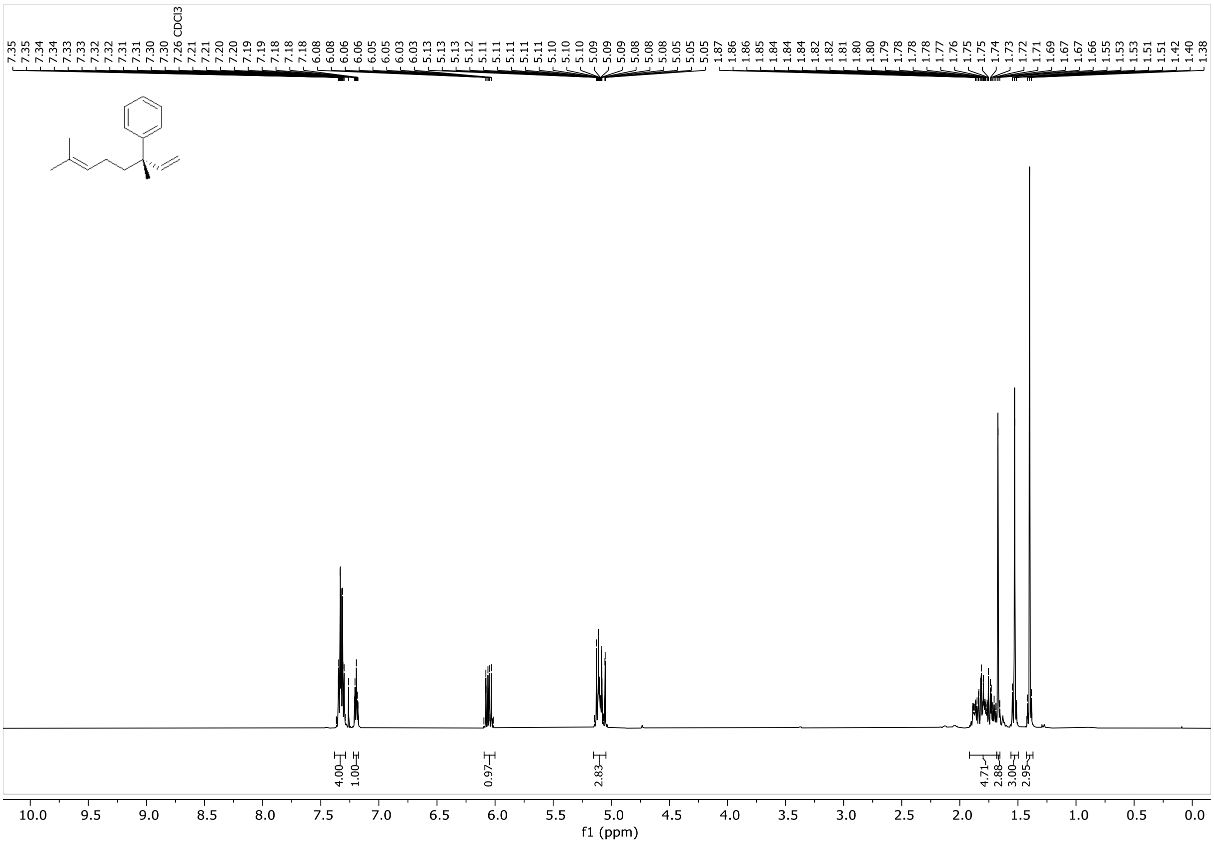

*Figure S27 ^{1}H-NMR(600 MHz) of **5b** in $CDCl_3$.*

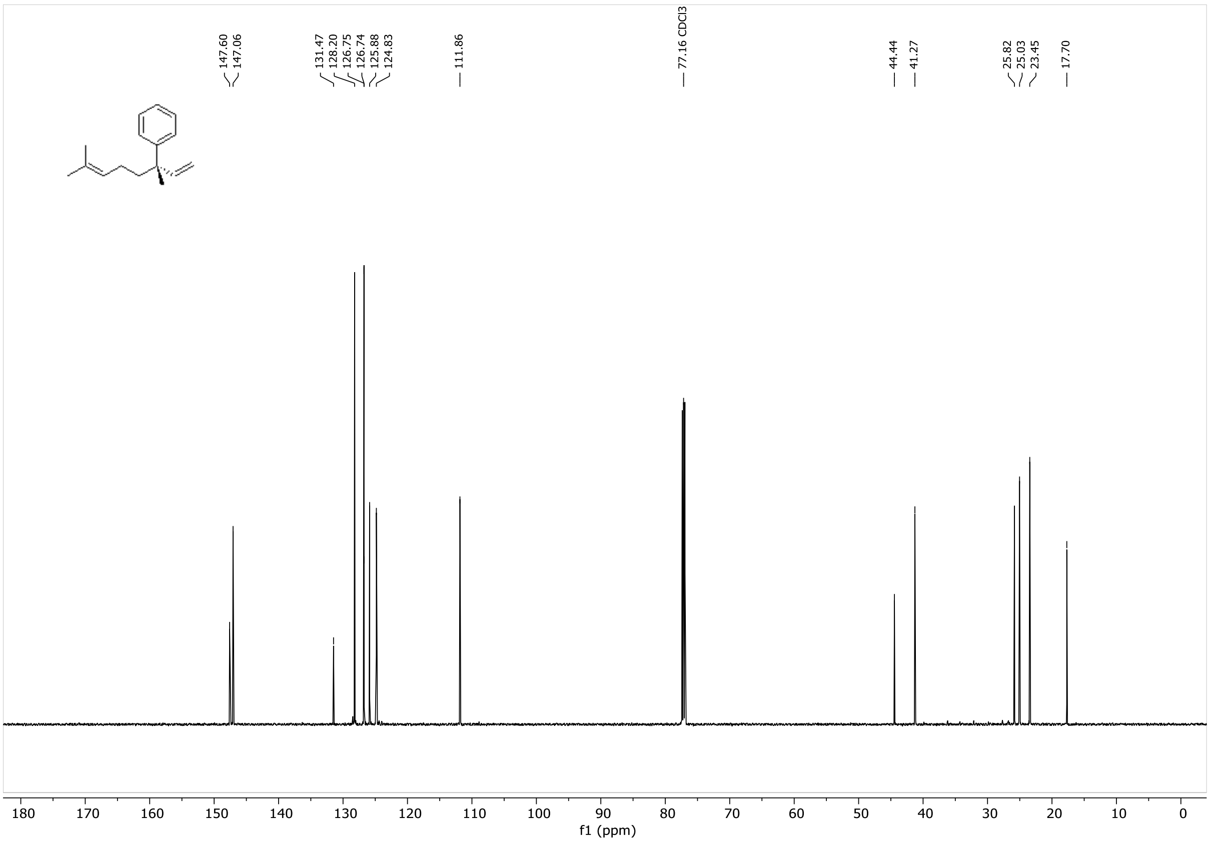

*Figure S28 $^{13}C\{^{1}H\}$-NMR(151 MHz) of **5b** in $CDCl_3$.*

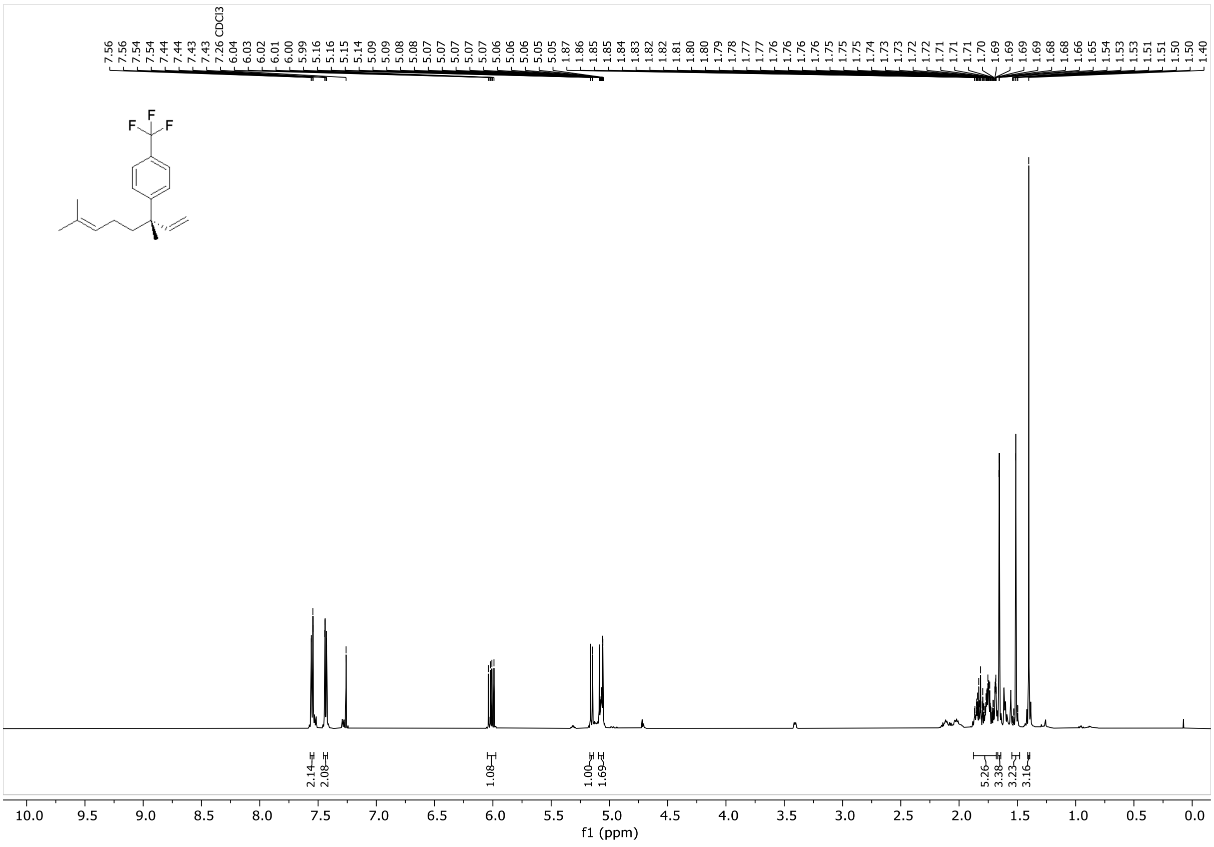

*Figure S29 ^{1}H-NMR(600 MHz) of **5c** in $CDCl_3$.*

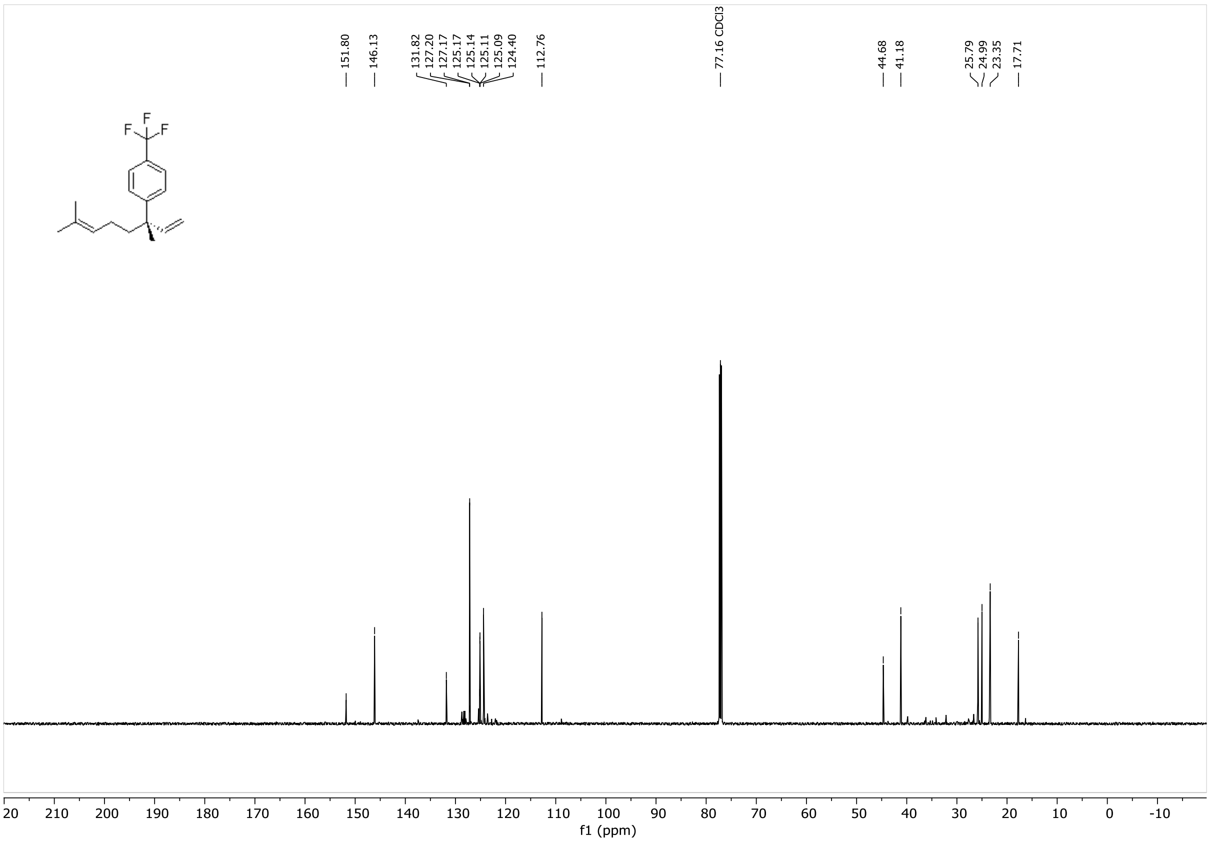

*Figure S30 $^{13}C\{^1H\}$-NMR(151 MHz) of **5c** in $CDCl_3$.*

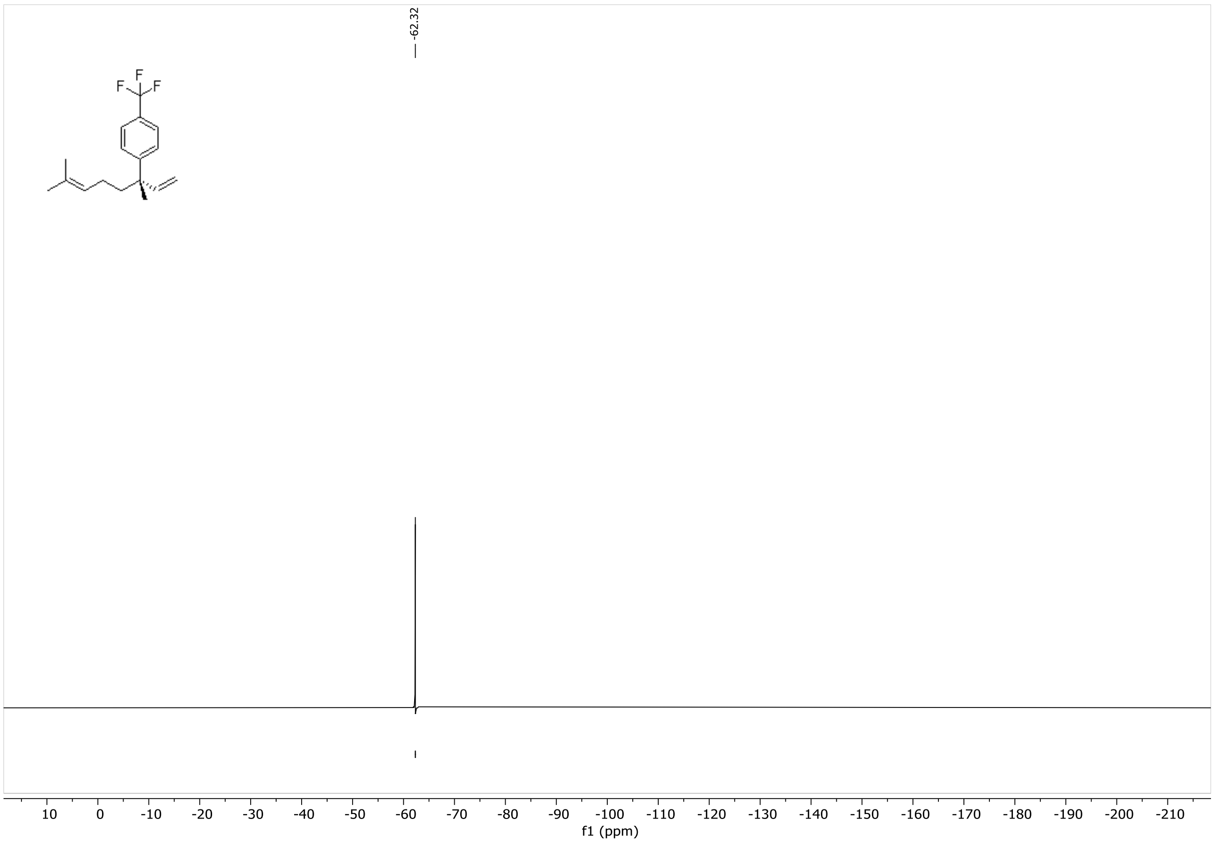

*Figure S31 ^{19}F-NMR(282 MHz) of **5c** in $CDCl_3$.*

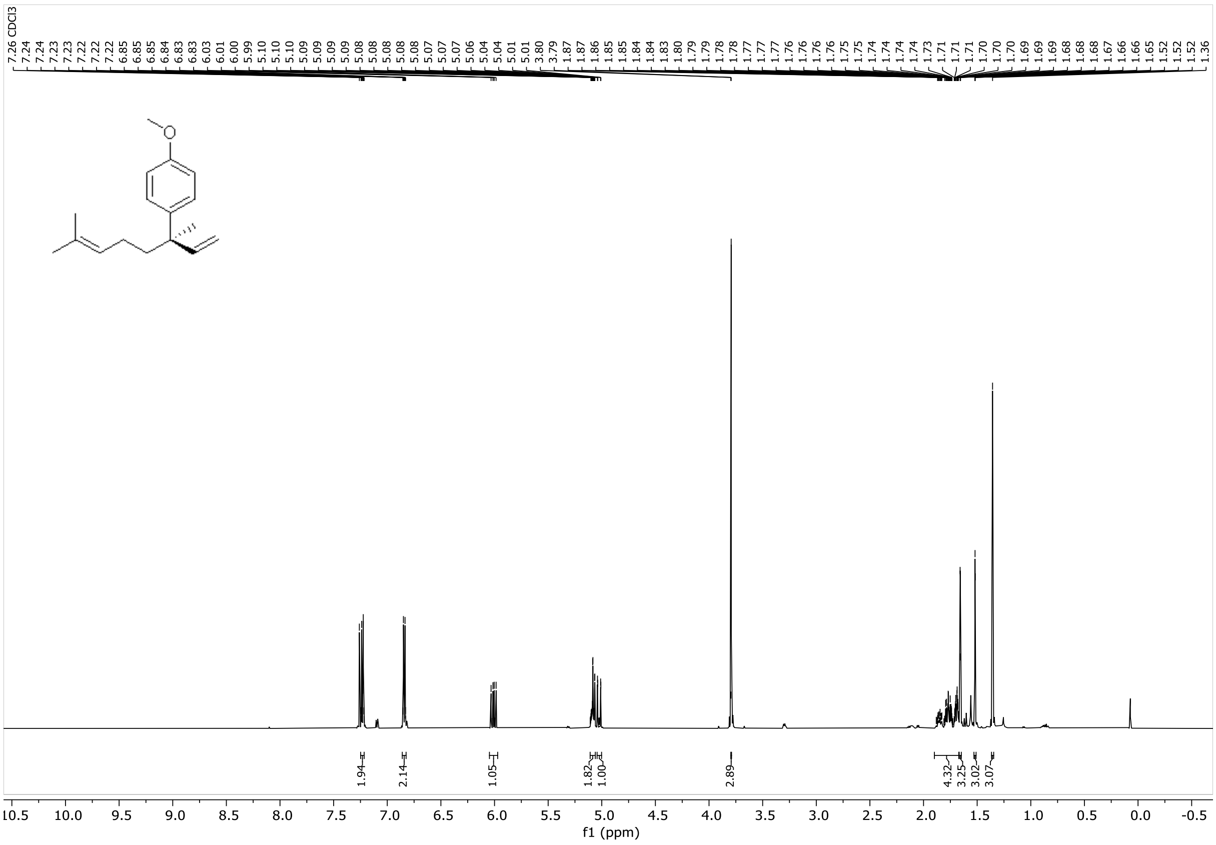

Figure S32 [1]*H-NMR(600 MHz) of* ***5d*** *in* $CDCl_3$.

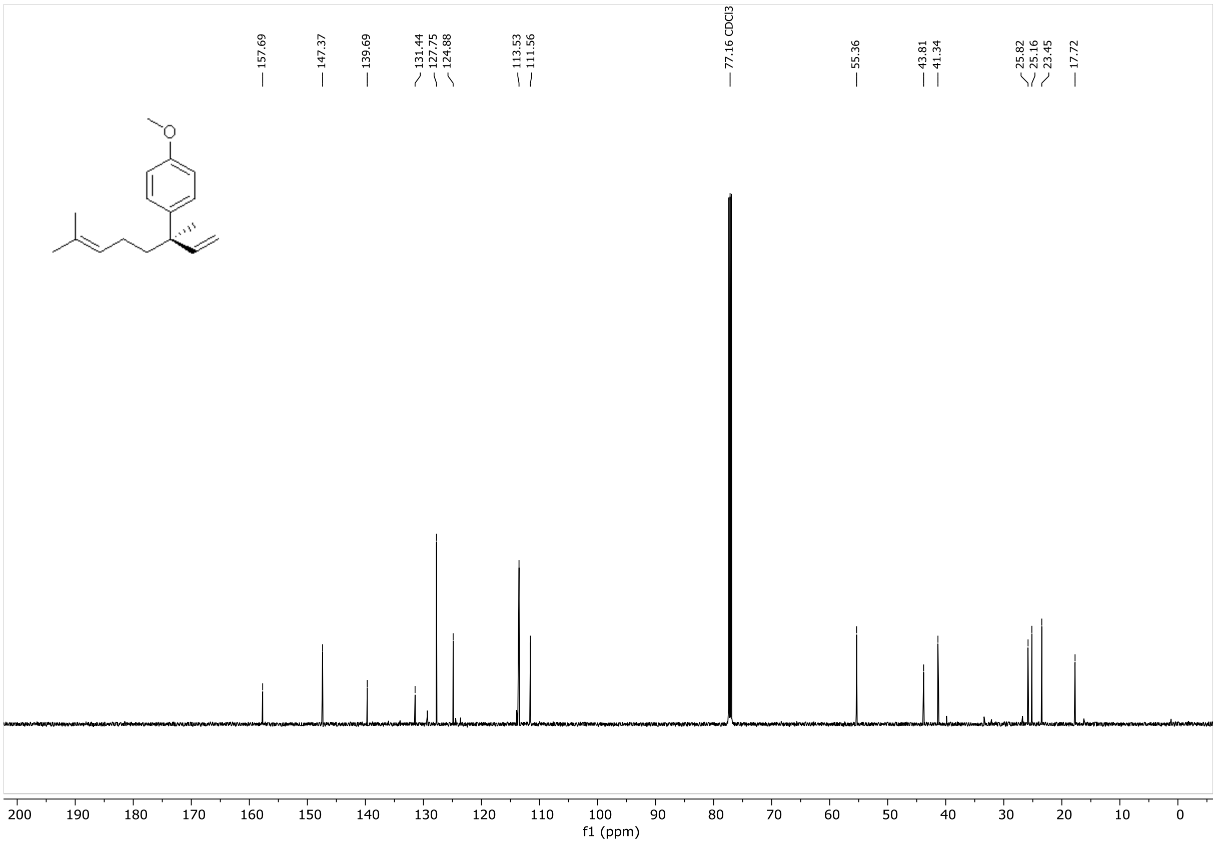

Figure S33 [13]*C{*[1]*H}-NMR(151 MHz) of* ***5d*** *in* $CDCl_3$.

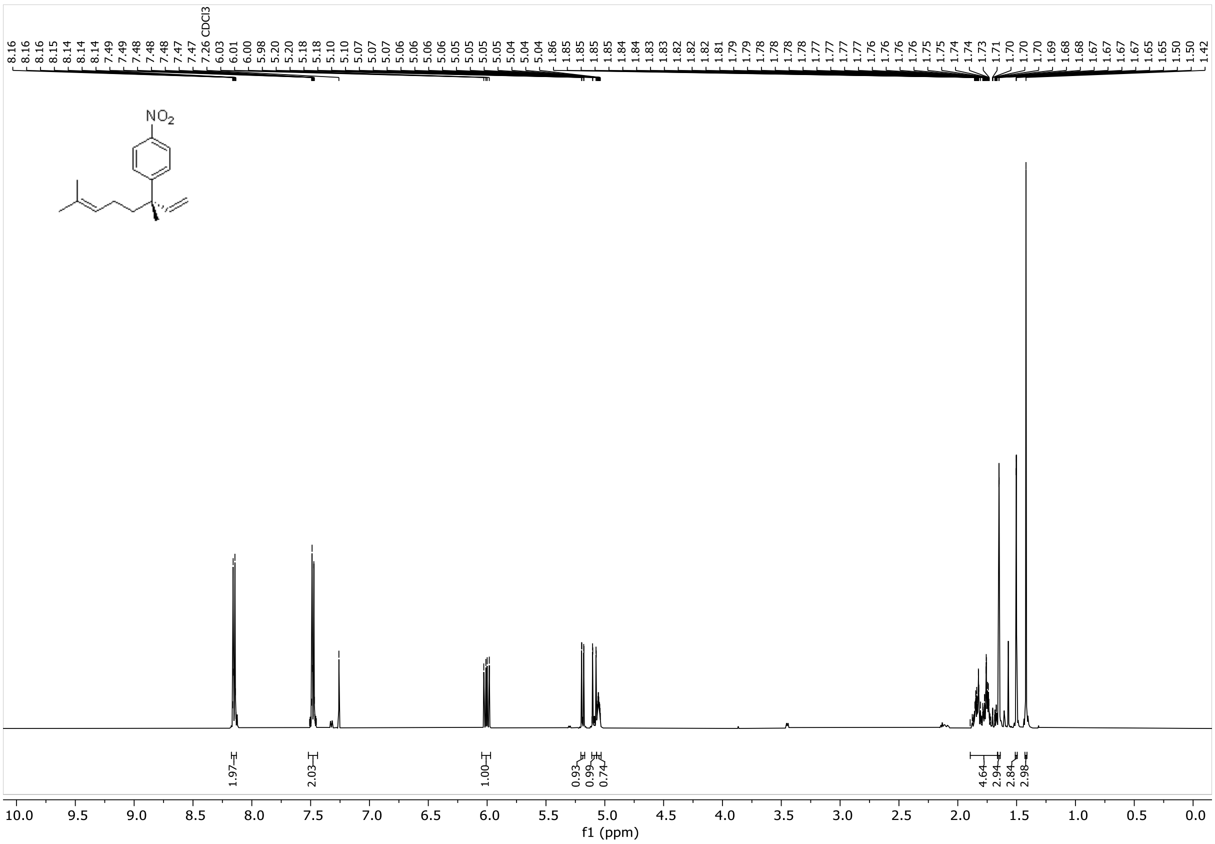

*Figure S34 ^{1}H-NMR(600 MHz) of **5e** in $CDCl_3$.*

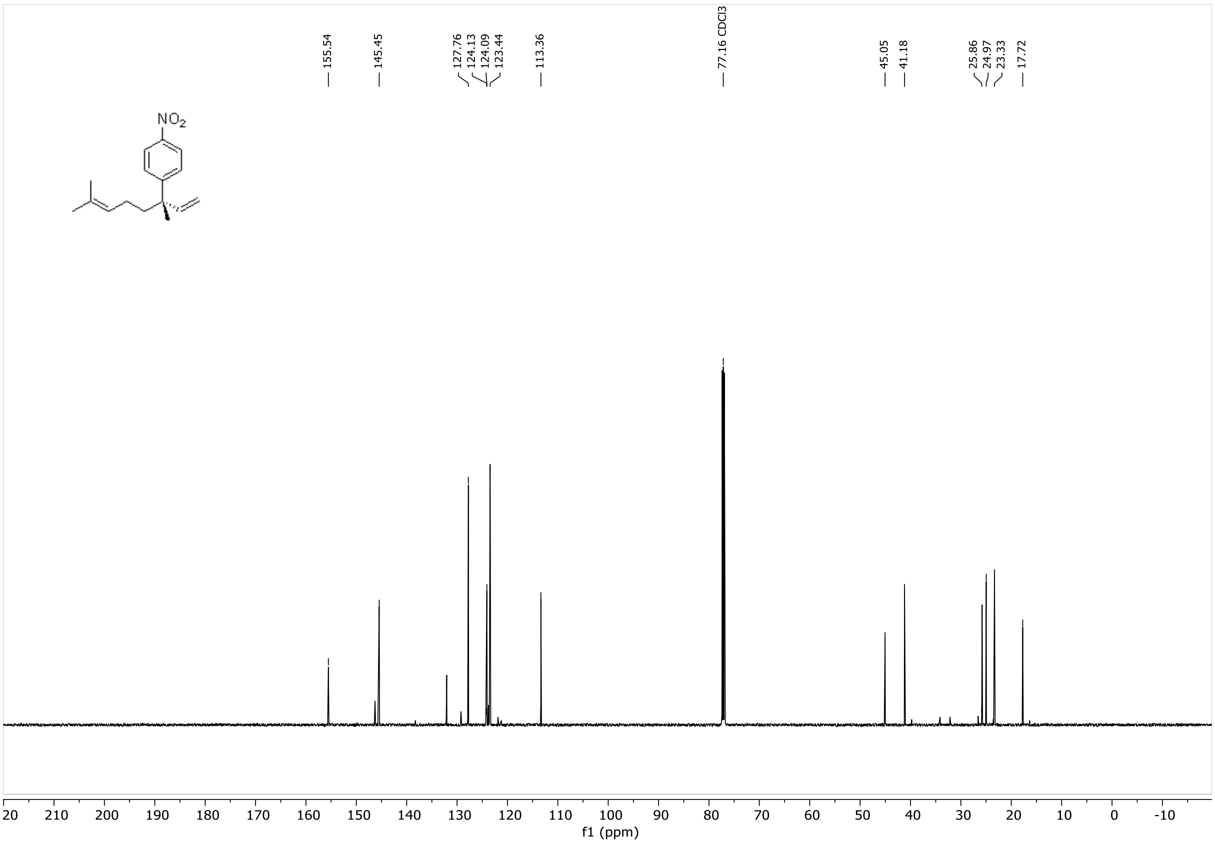

*Figure S35 $^{13}C\{^{1}H\}$-NMR(151 MHz) of **5e** in $CDCl_3$.*

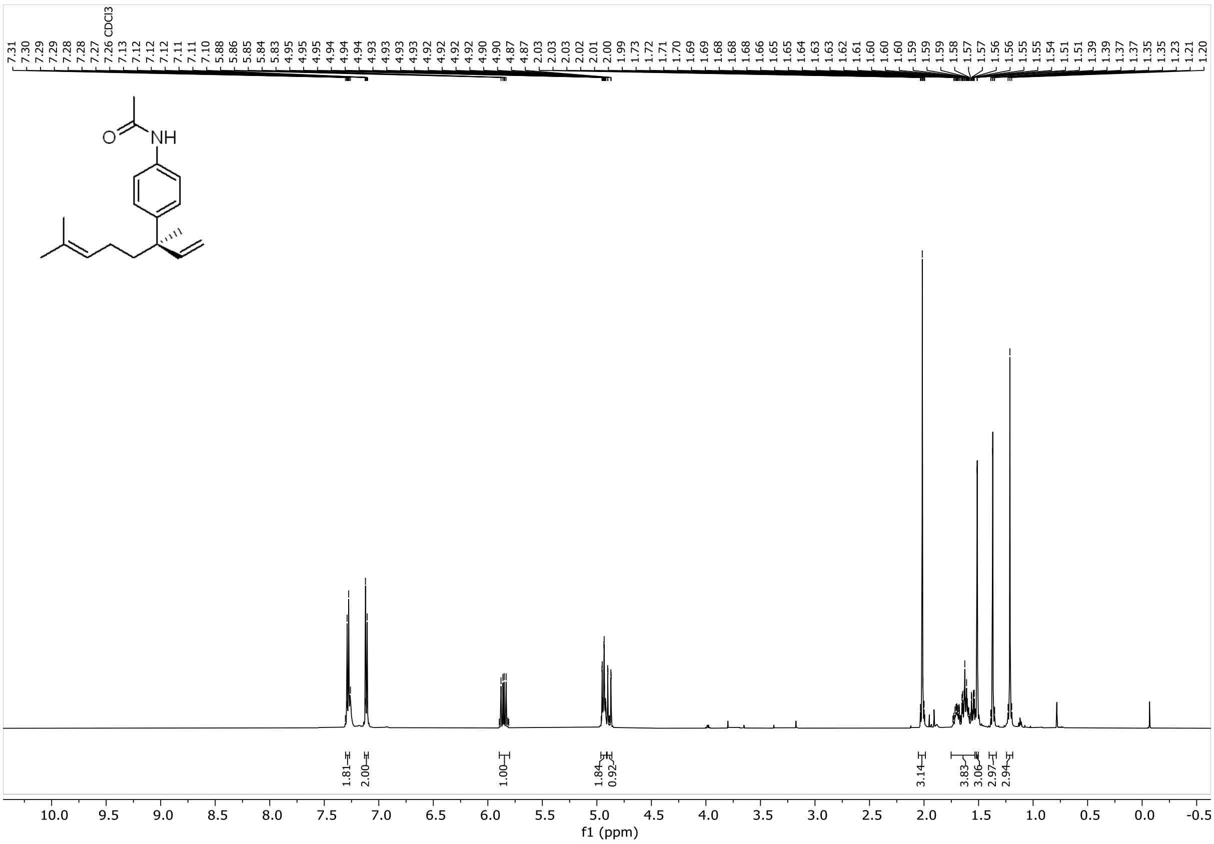

*Figure S36 ^{1}H-NMR(600 MHz) of **5f** in $CDCl_3$.*

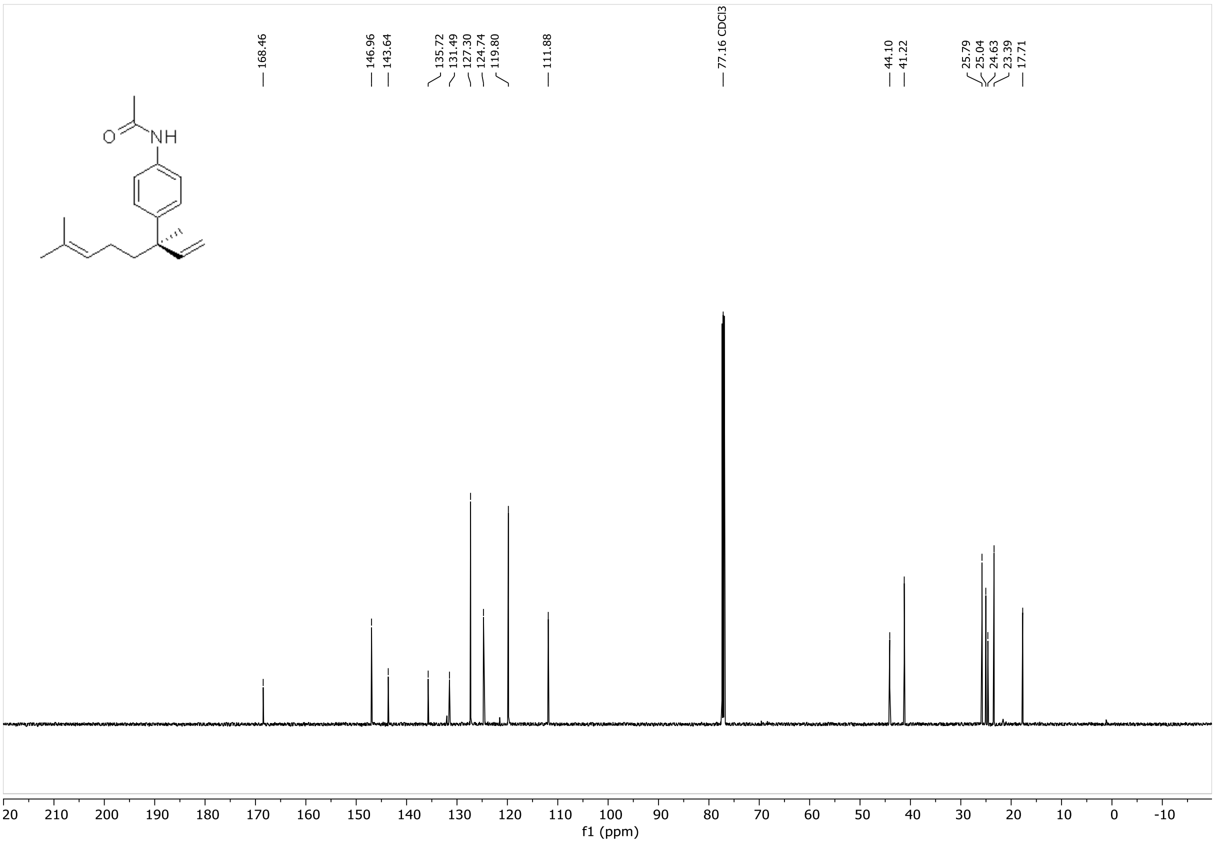

*Figure S37 ^{13}C{^{1}H}-NMR(151 MHz) of **5f** in $CDCl_3$.*

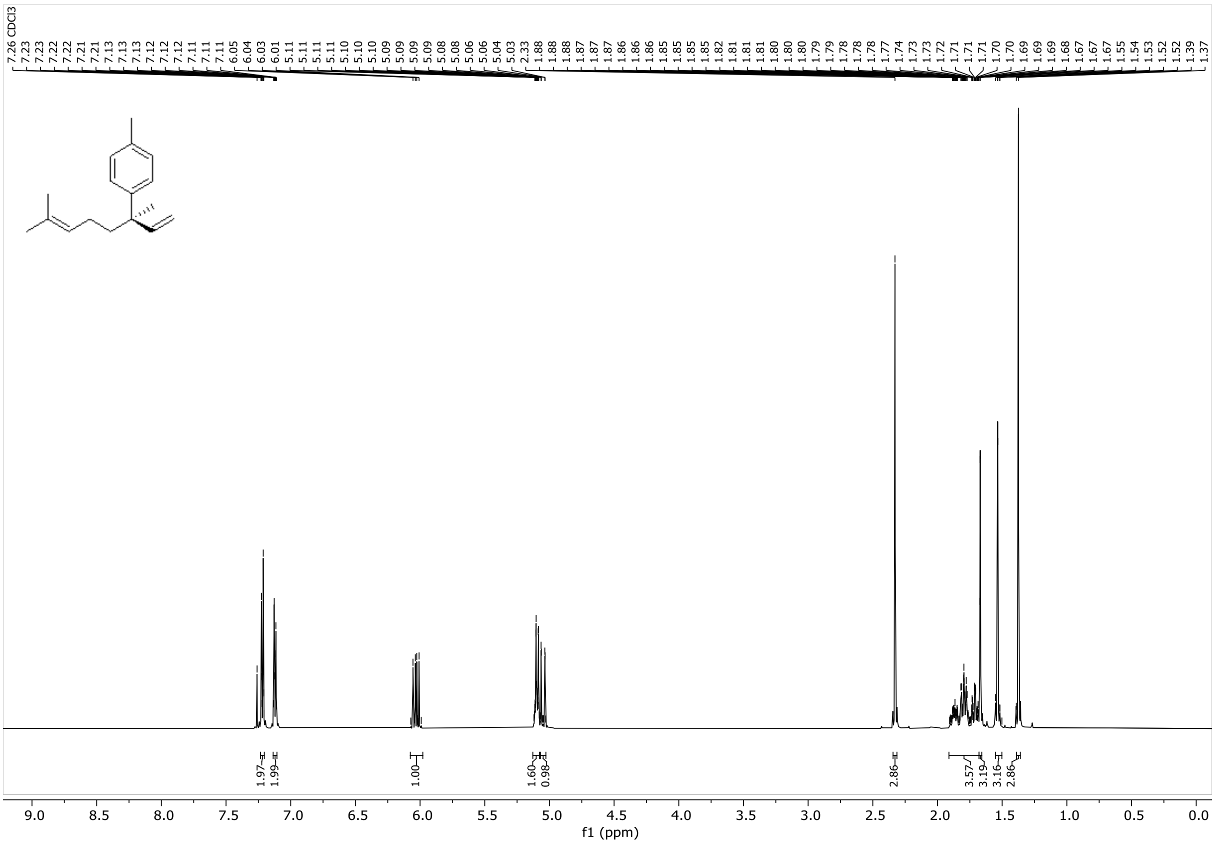

*Figure S38 ^{1}H-NMR(600 MHz) of **5g** in $CDCl_3$.*

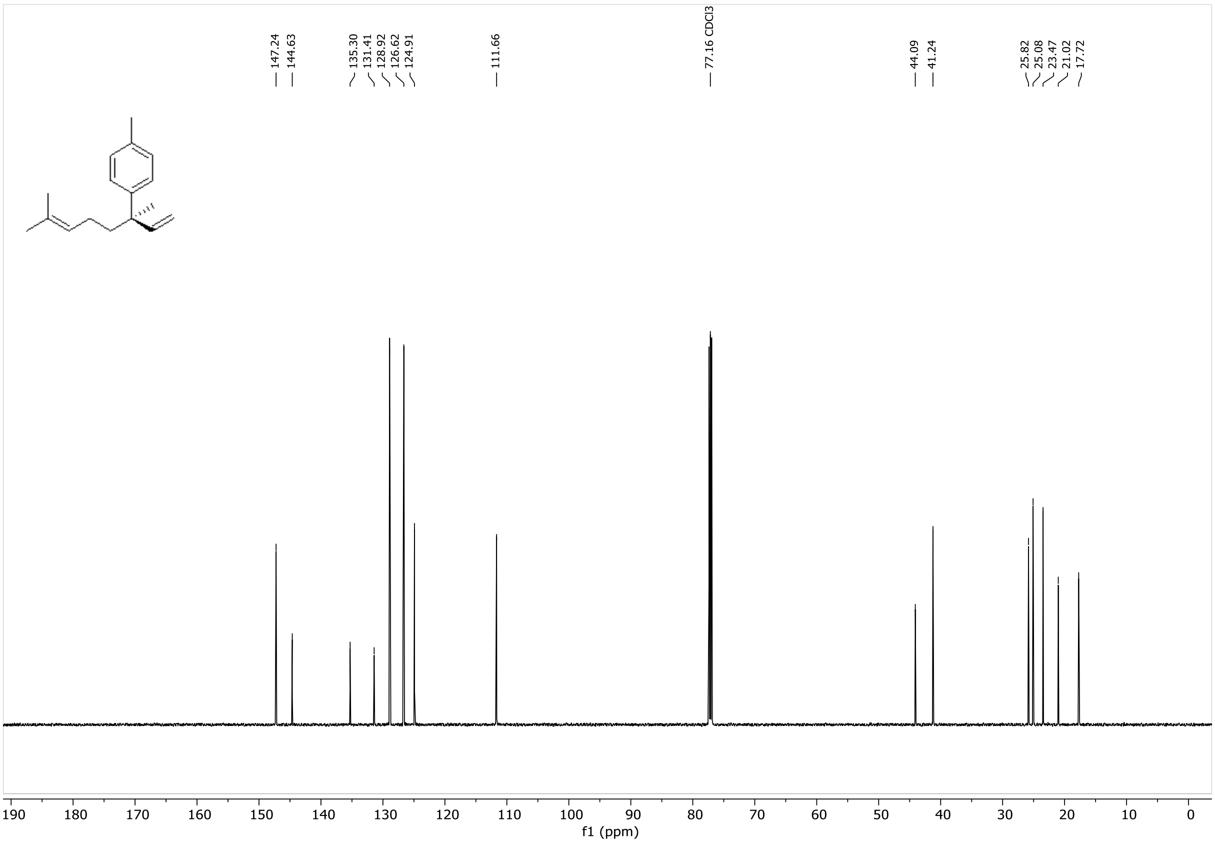

*Figure S39 $^{13}C\{^{1}H\}$-NMR(151 MHz) of **5g** in $CDCl_3$.*

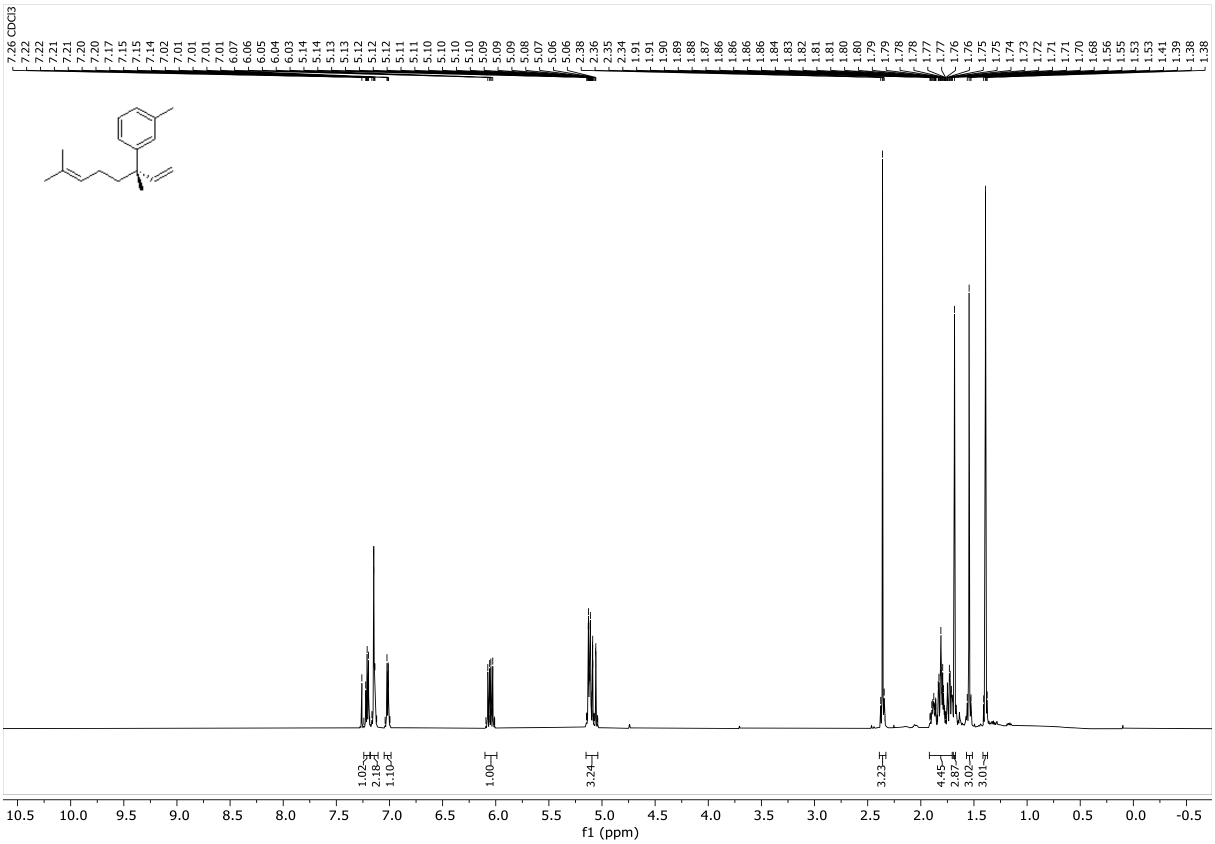

*Figure S40 ^{1}H-NMR(600 MHz) of **5h** in $CDCl_3$.*

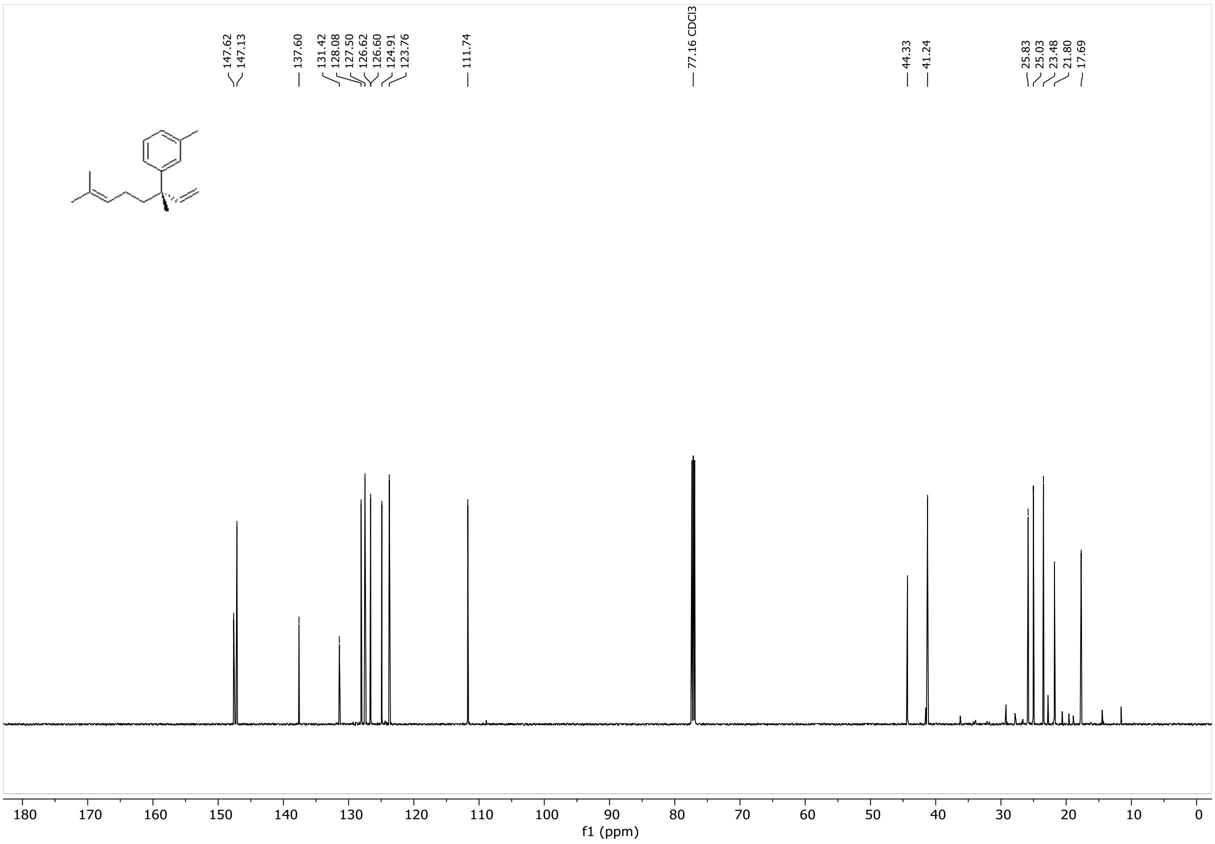

*Figure S41 ^{13}C{^{1}H}-NMR(151 MHz) of **5h** in $CDCl_3$.*

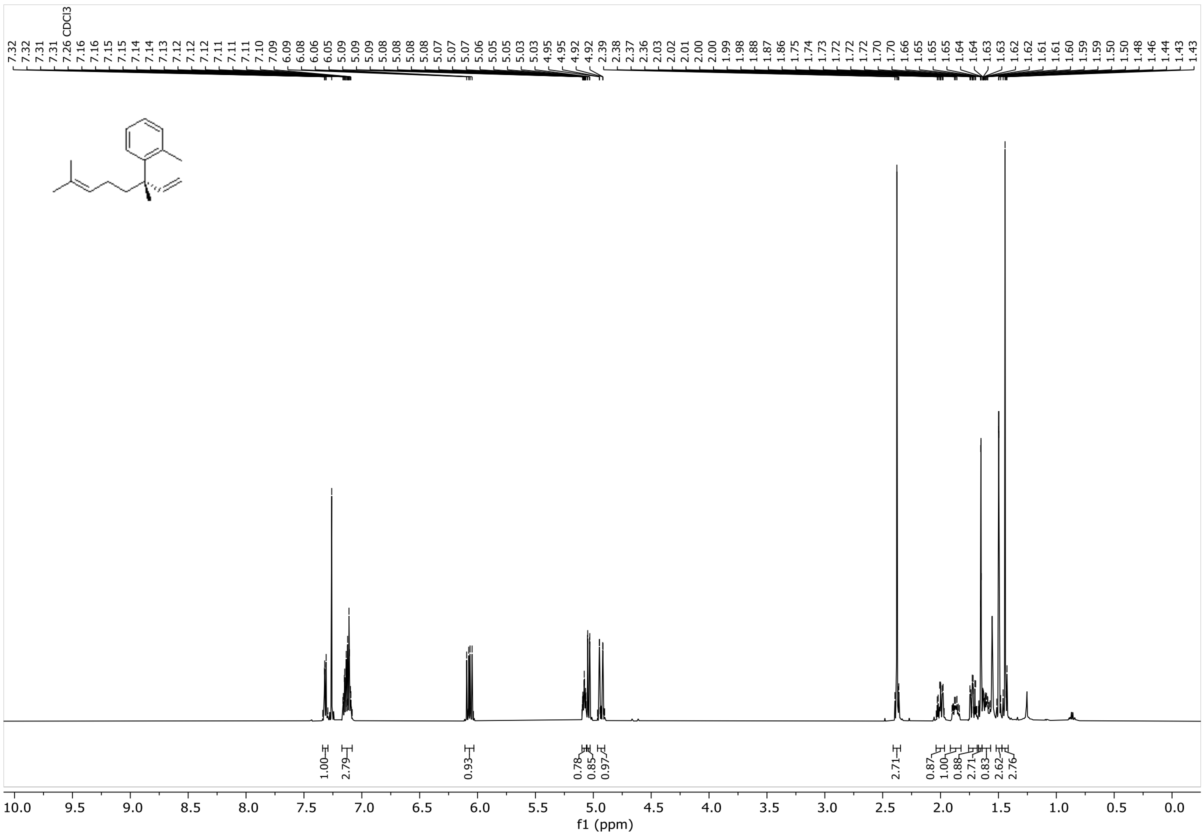

*Figure S42 ^{1}H-NMR(600 MHz) of **5i** in $CDCl_3$.*

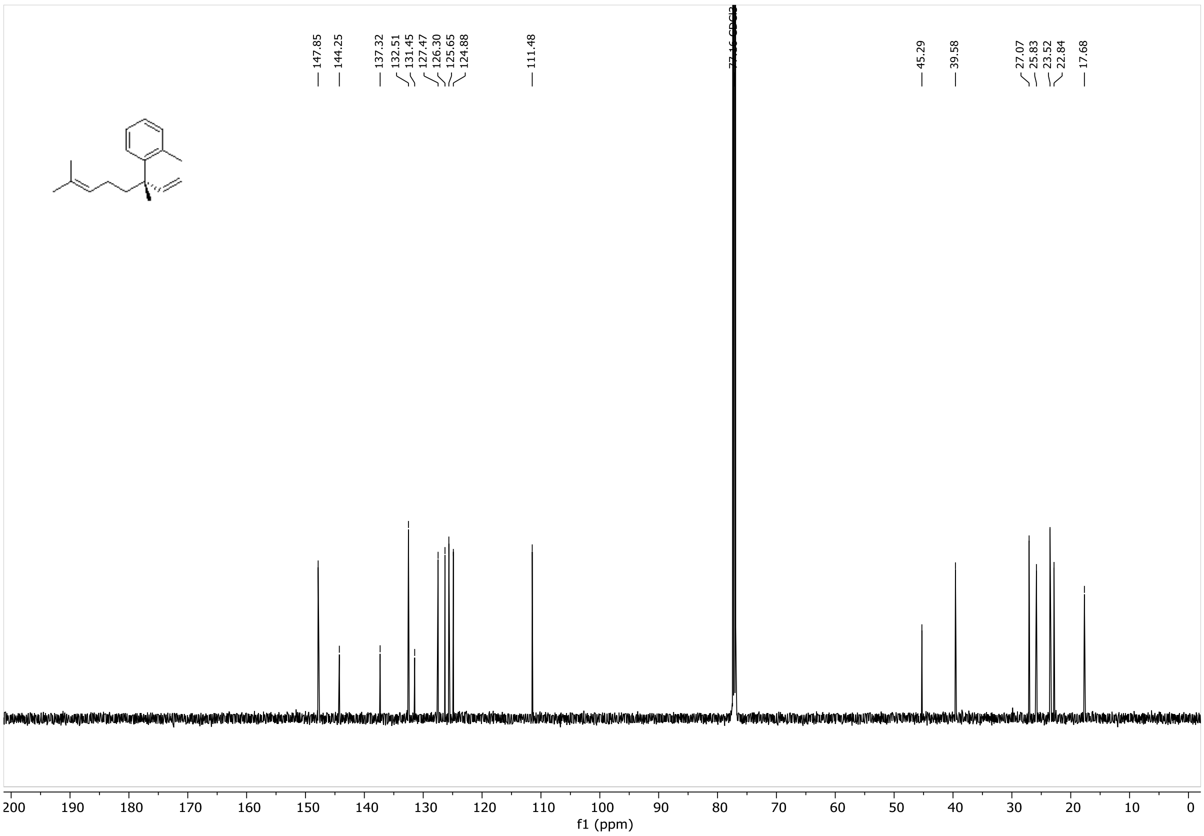

*Figure S43 $^{13}C\{^{1}H\}$-NMR(151 MHz) of **5i** in $CDCl_3$.*

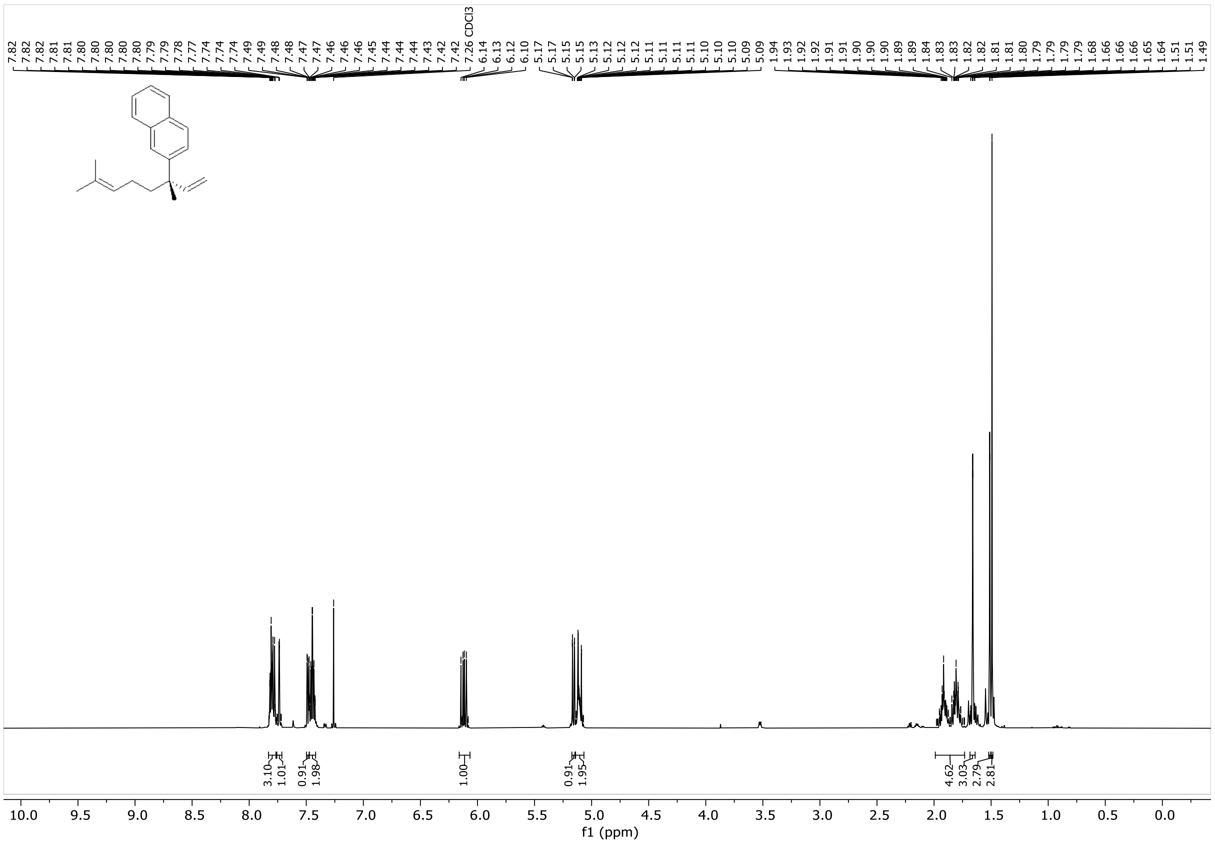

*Figure S44 ^{1}H-NMR(600 MHz) of **5j** in $CDCl_3$.*

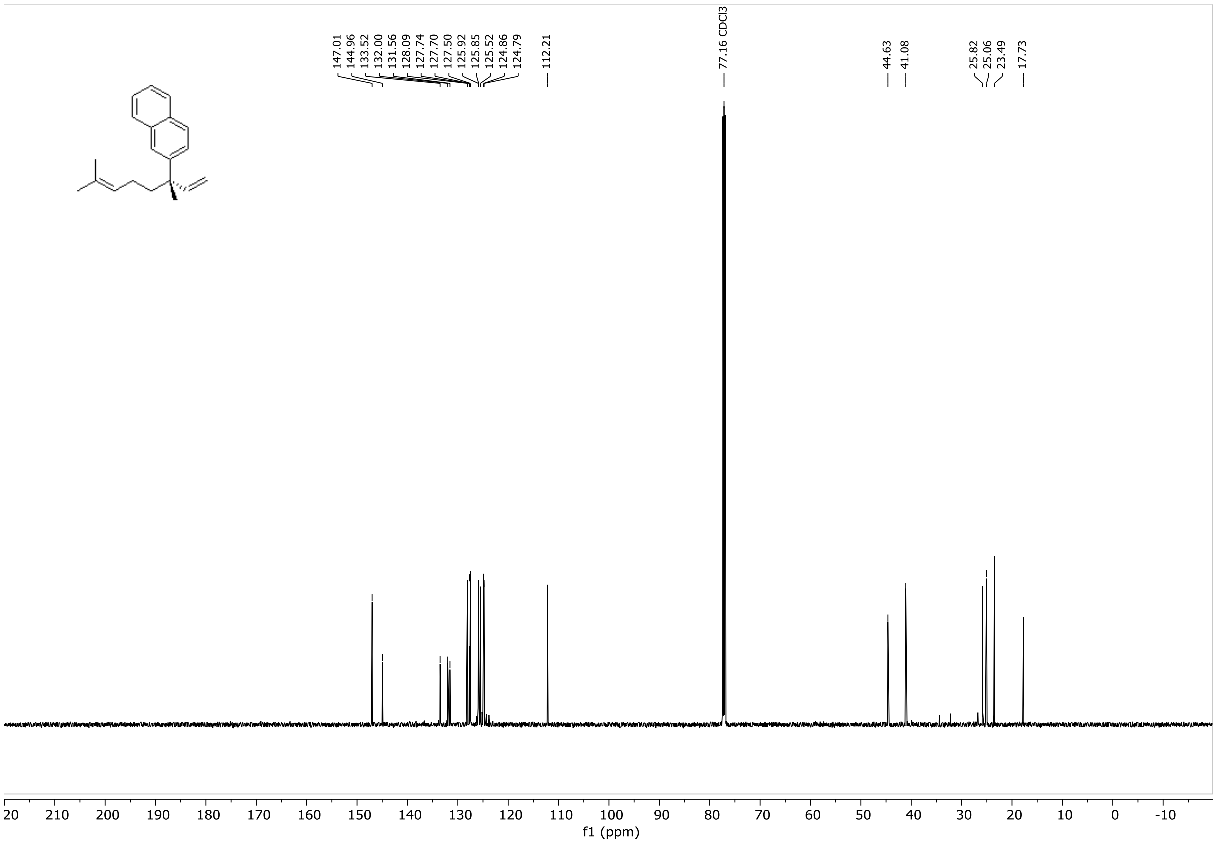

*Figure S45 $^{13}C\{^{1}H\}$-NMR(151 MHz) of **5j** in $CDCl_3$.*

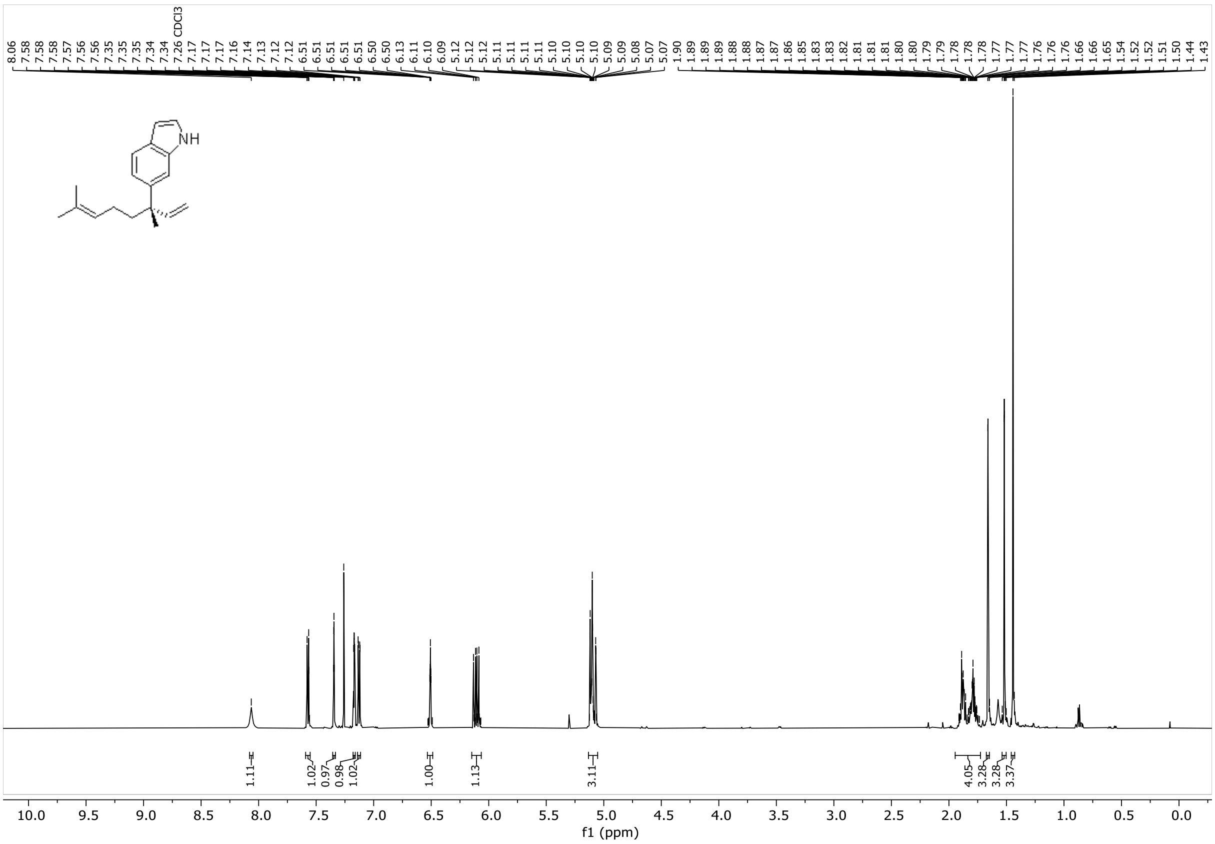

*Figure S46 ¹H-NMR(600 MHz) of **5k** in CDCl₃.*

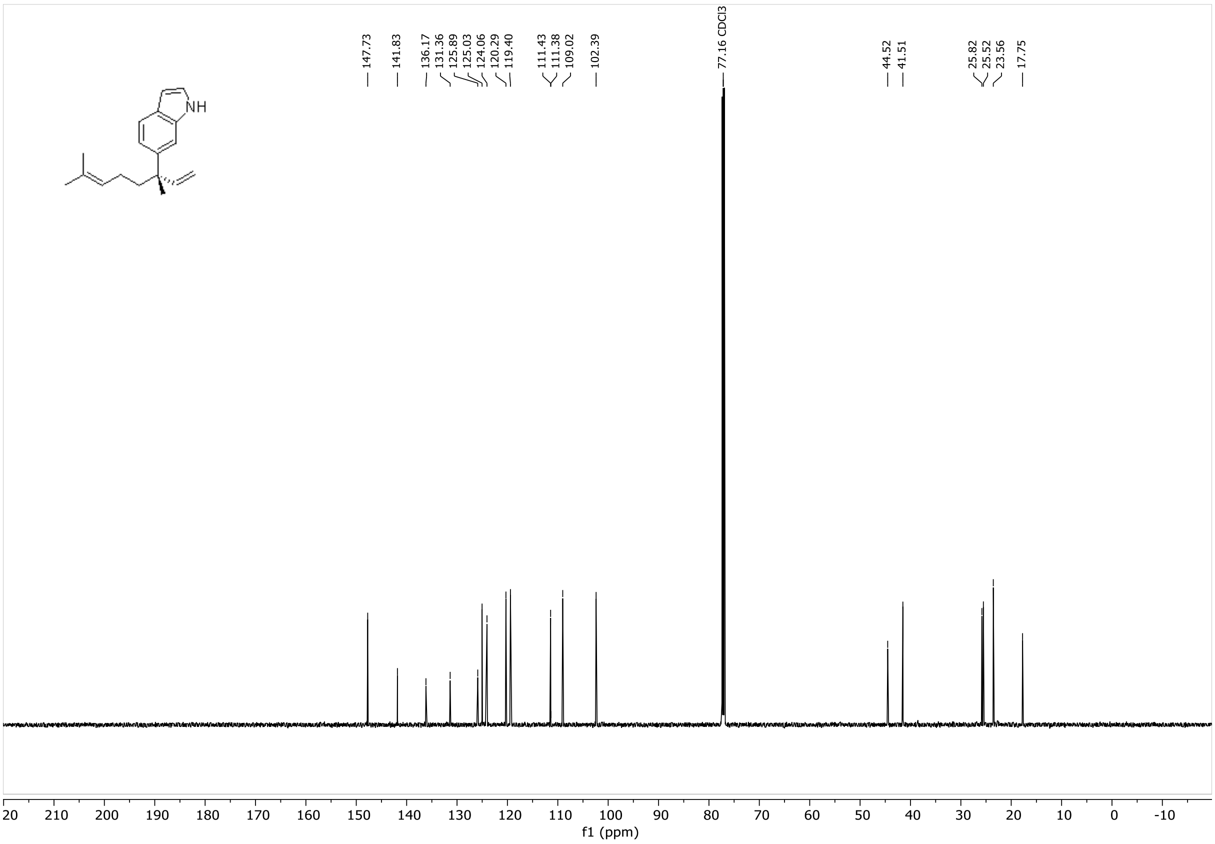

*Figure S47 $^{13}C\{^1H\}$-NMR(151 MHz) of **5k** in CDCl₃.*

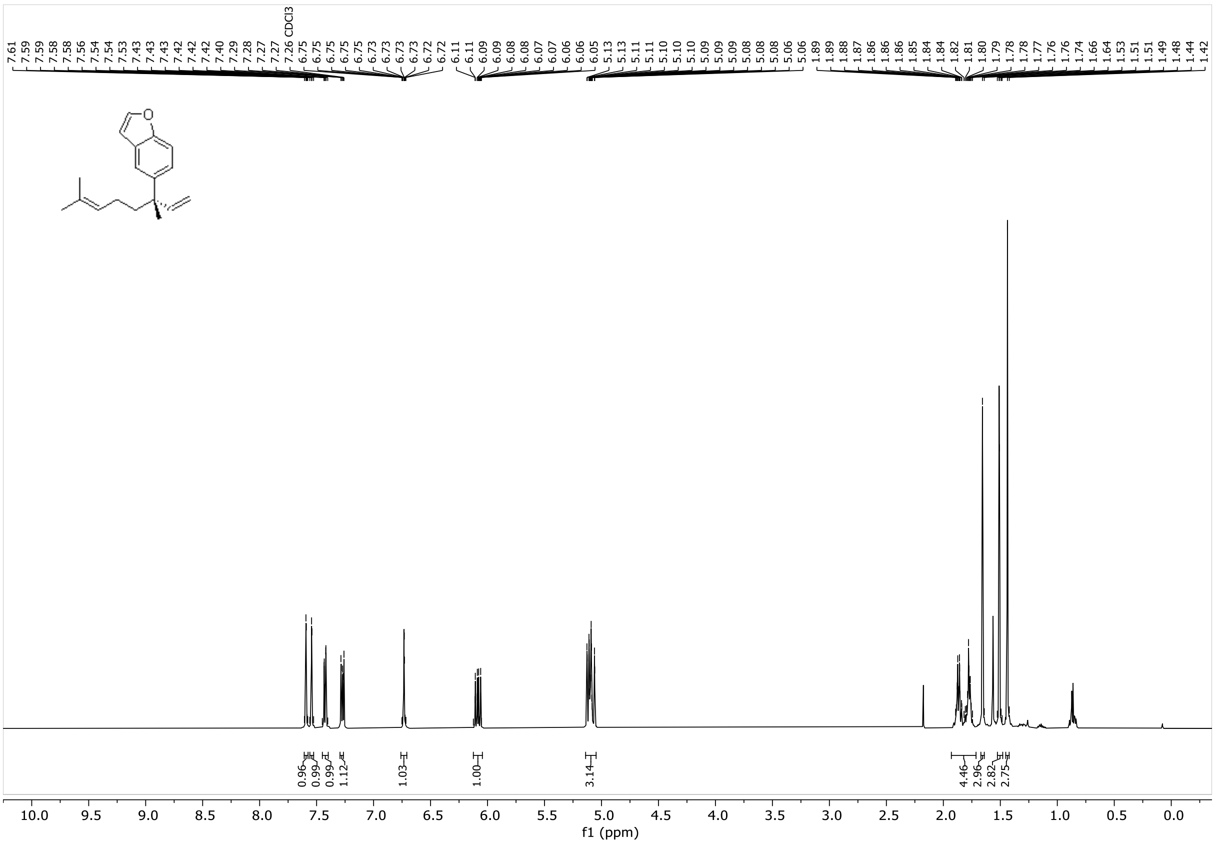

*Figure S48 ^{1}H-NMR(600 MHz) of **5l** in $CDCl_3$.*

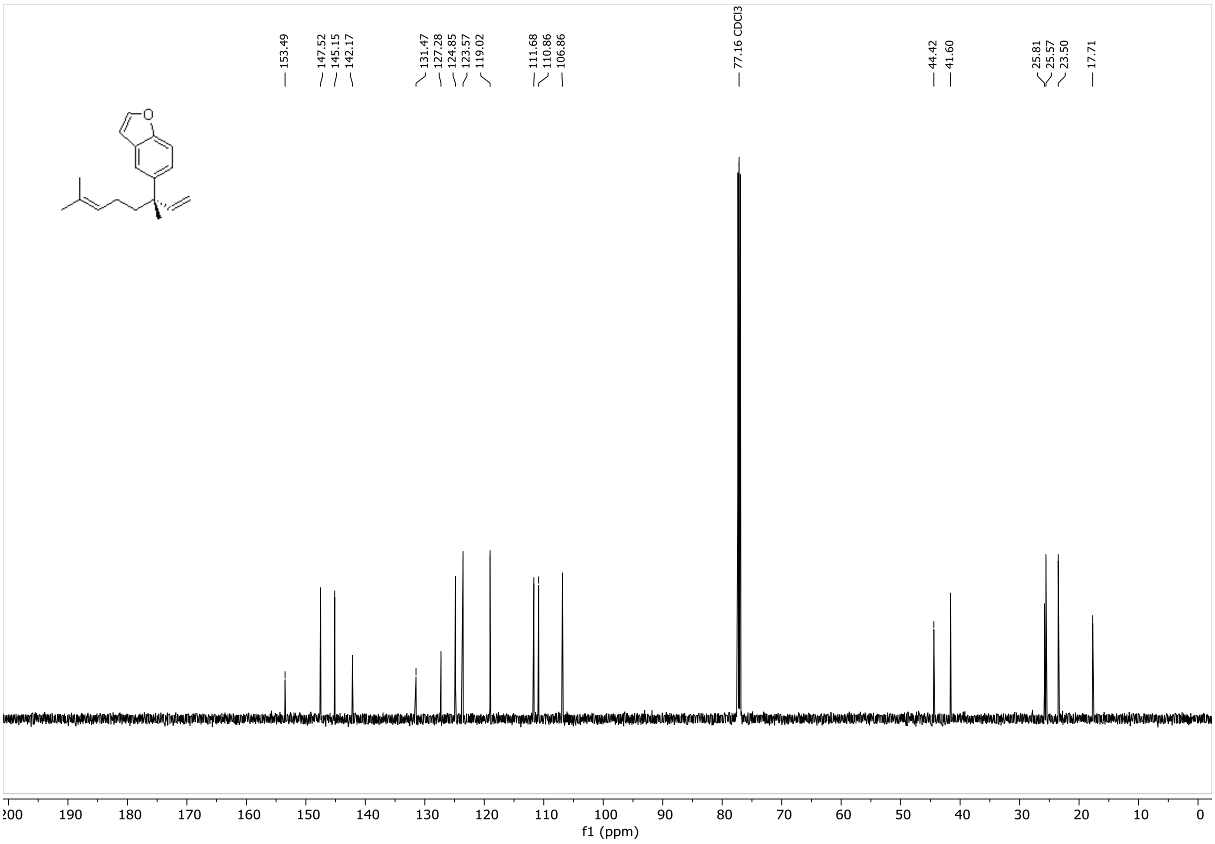

*Figure S49 $^{13}C\{^1H\}$-NMR(151 MHz) of **5l** in $CDCl_3$.*

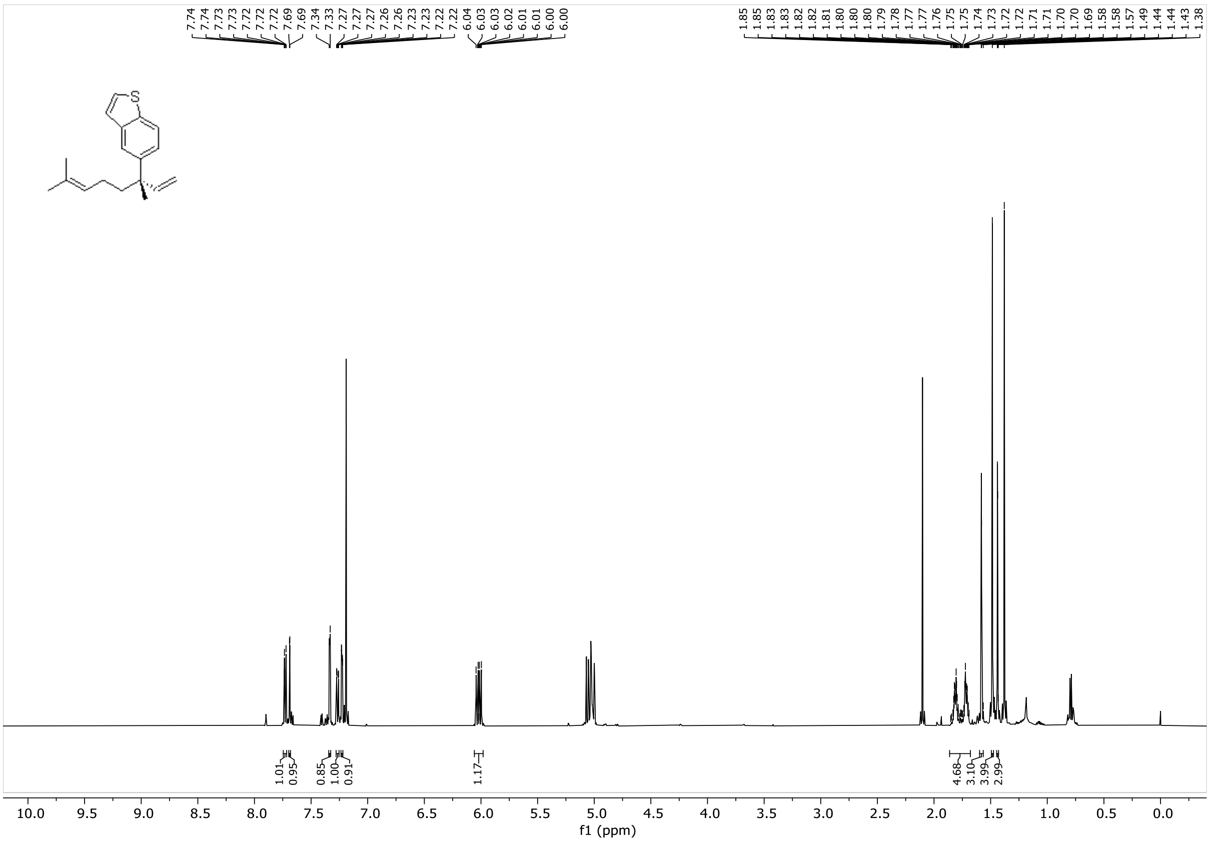

*Figure S50 ¹H-NMR(600 MHz) of **5m** in CDCl₃.*

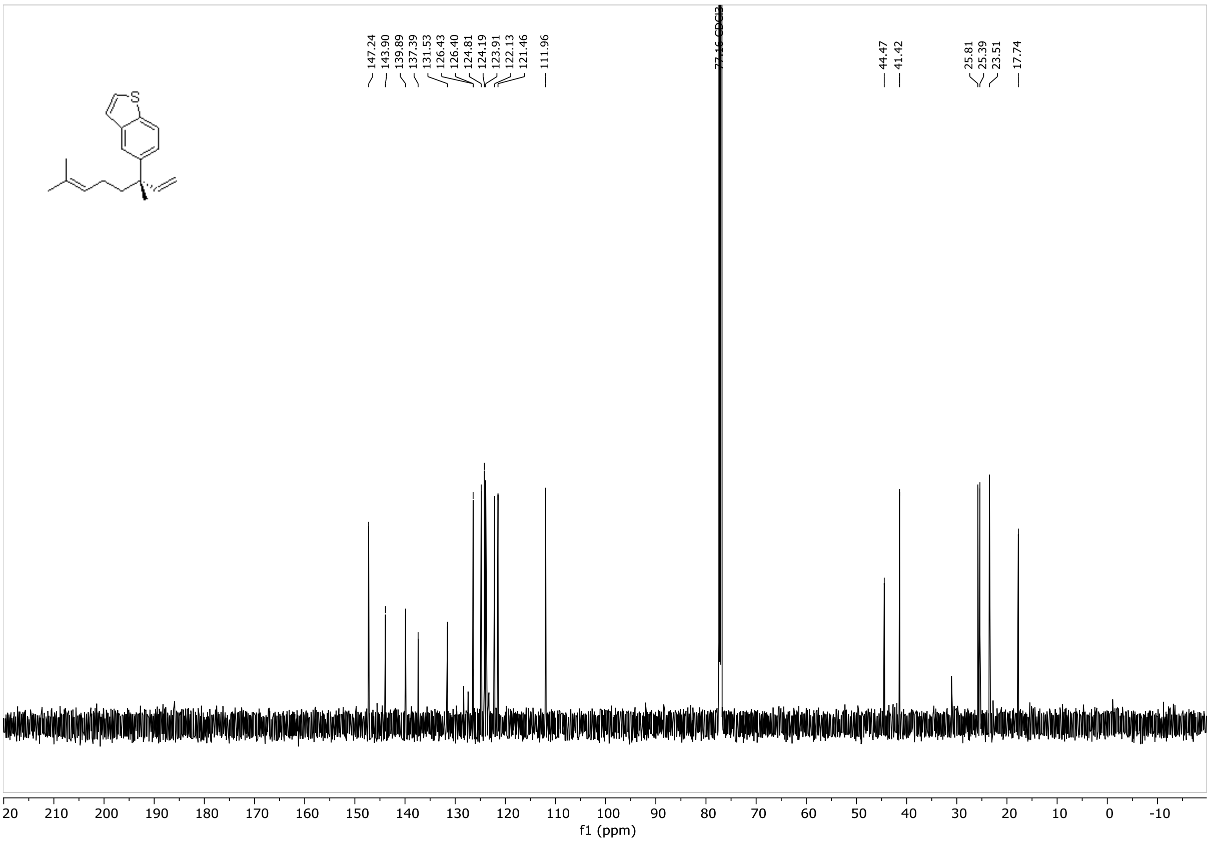

*Figure S51 ¹³C{¹H}-NMR(151 MHz) of **5m** in CDCl₃.*

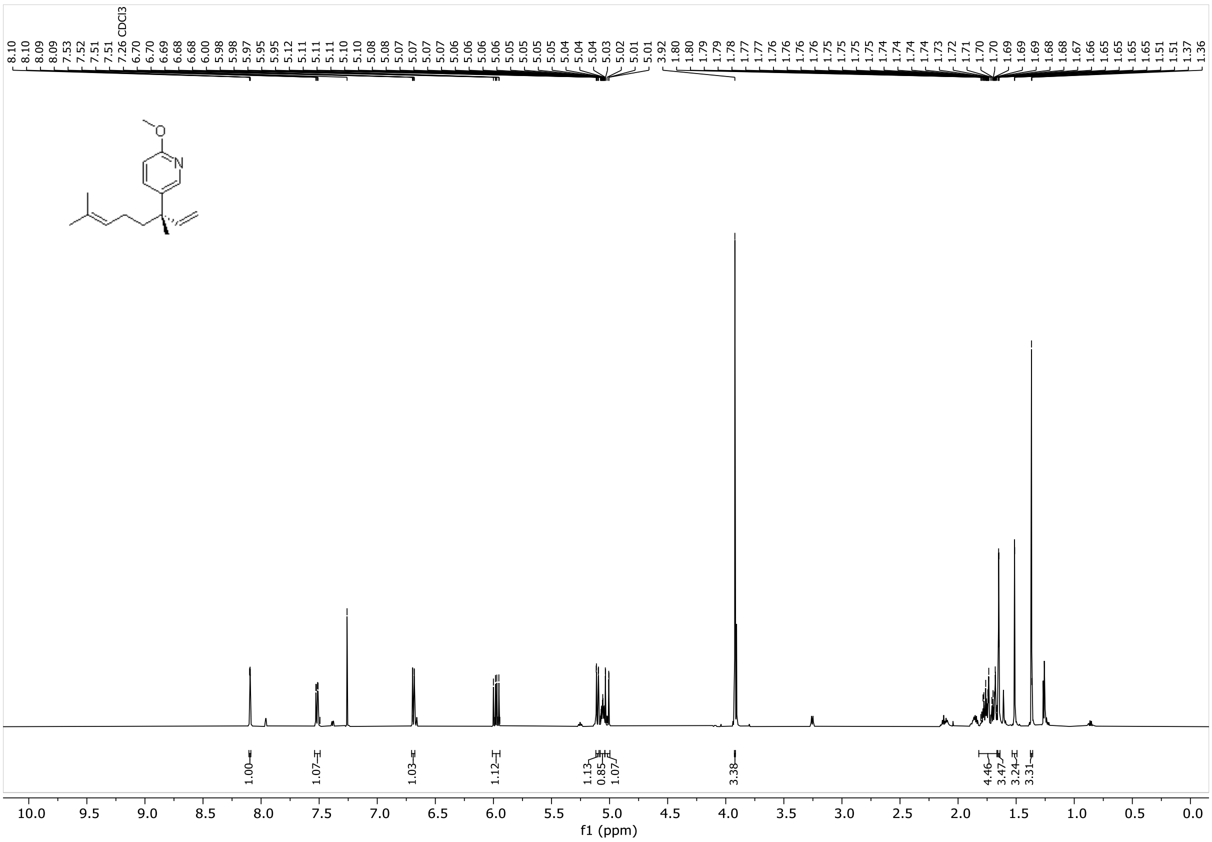

*Figure S52 ^{1}H-NMR(600 MHz) of **5n** in $CDCl_3$.*

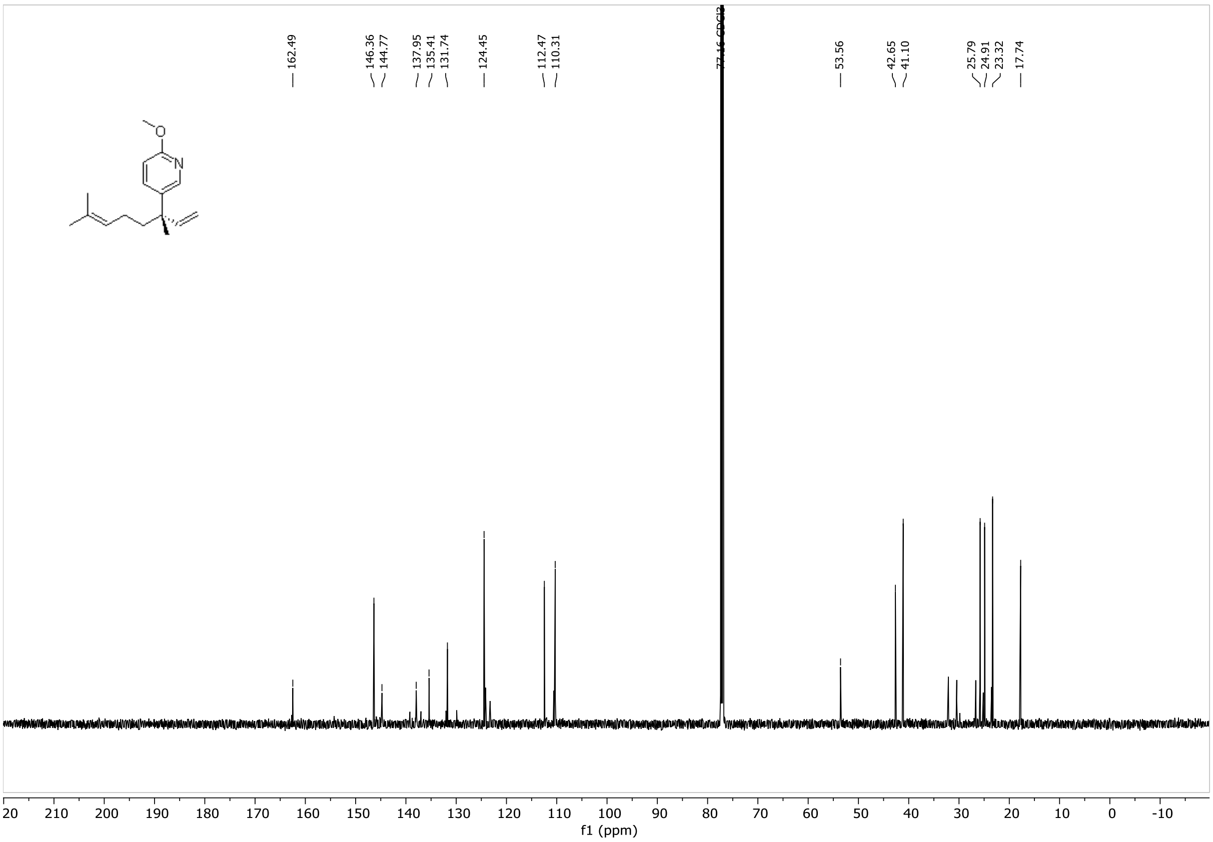

*Figure S53 $^{13}C\{^1H\}$-NMR(151 MHz) of **5n** in $CDCl_3$.*

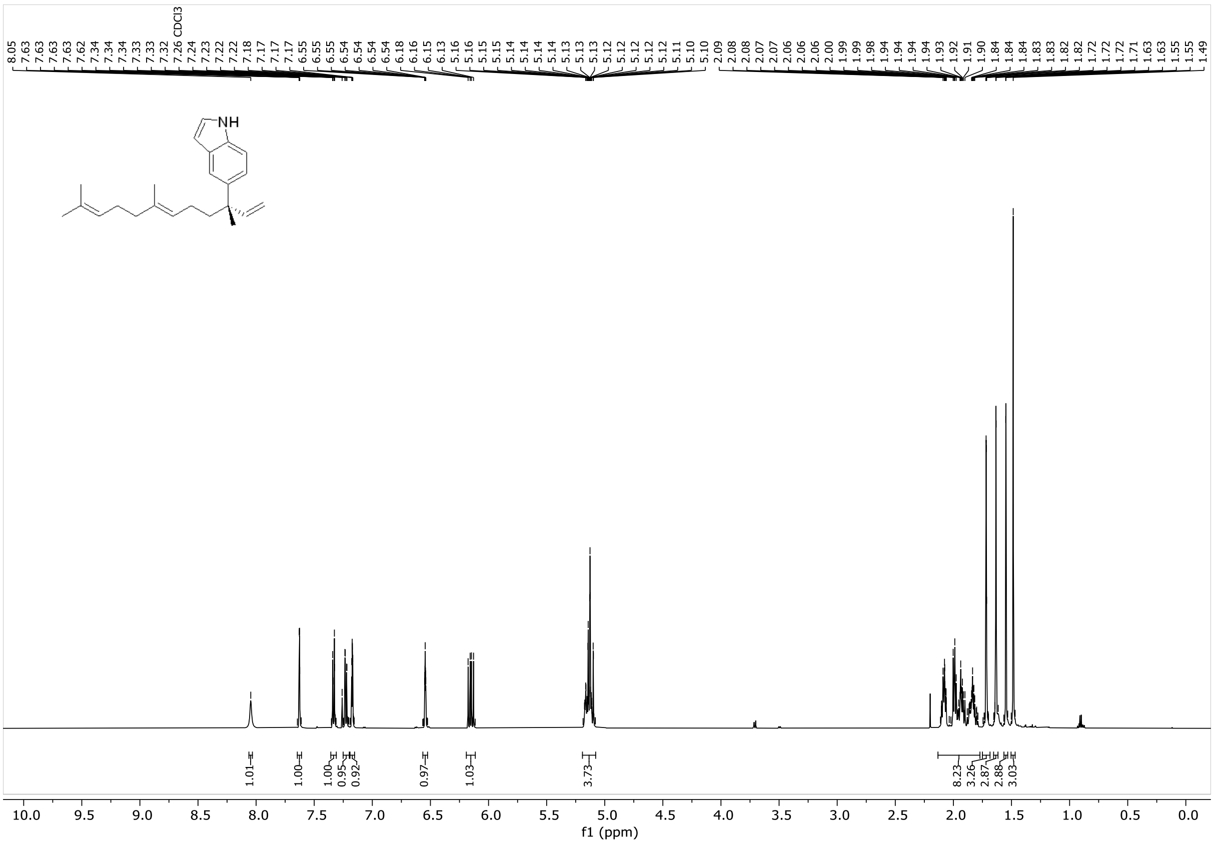

*Figure S54 ^{1}H-NMR(600 MHz) of **5o** in $CDCl_3$.*

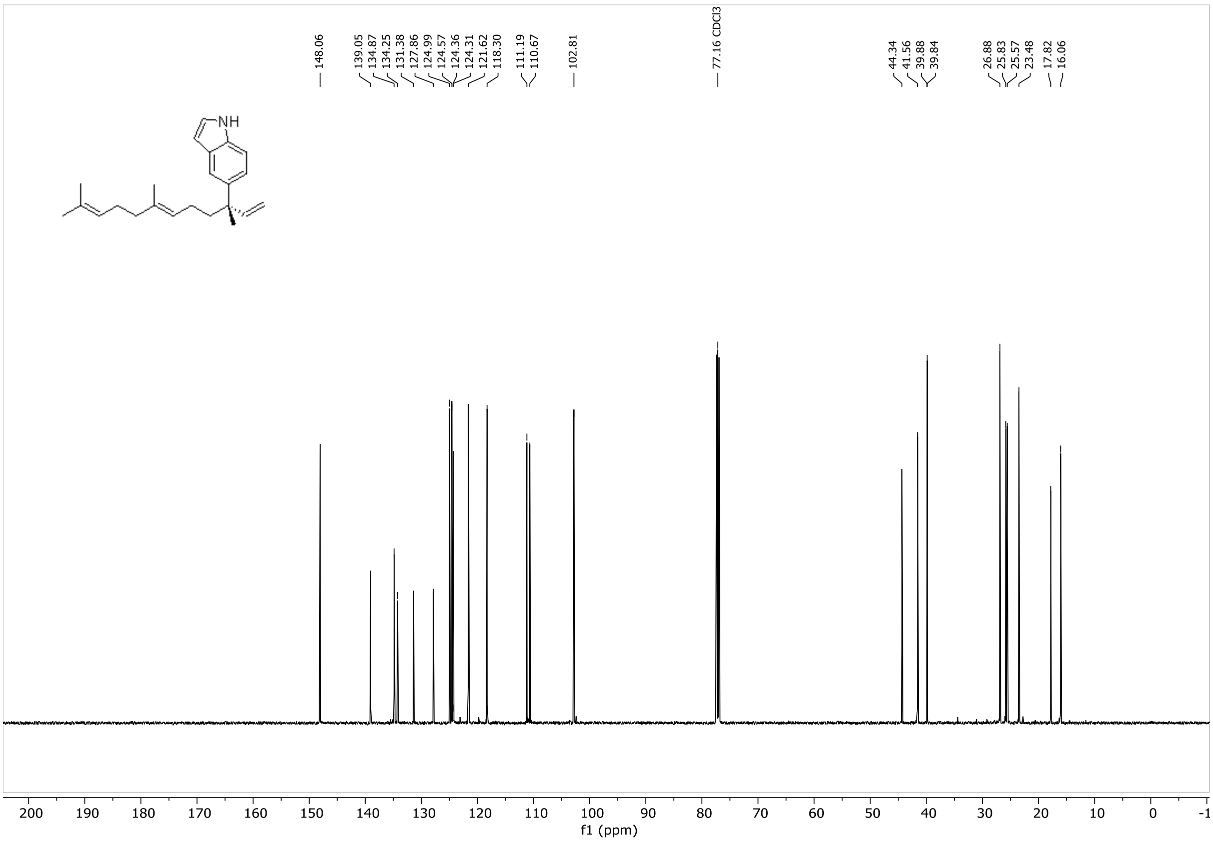

*Figure S55 $^{13}C\{^{1}H\}$-NMR(151 MHz) of **5o** in $CDCl_3$.*

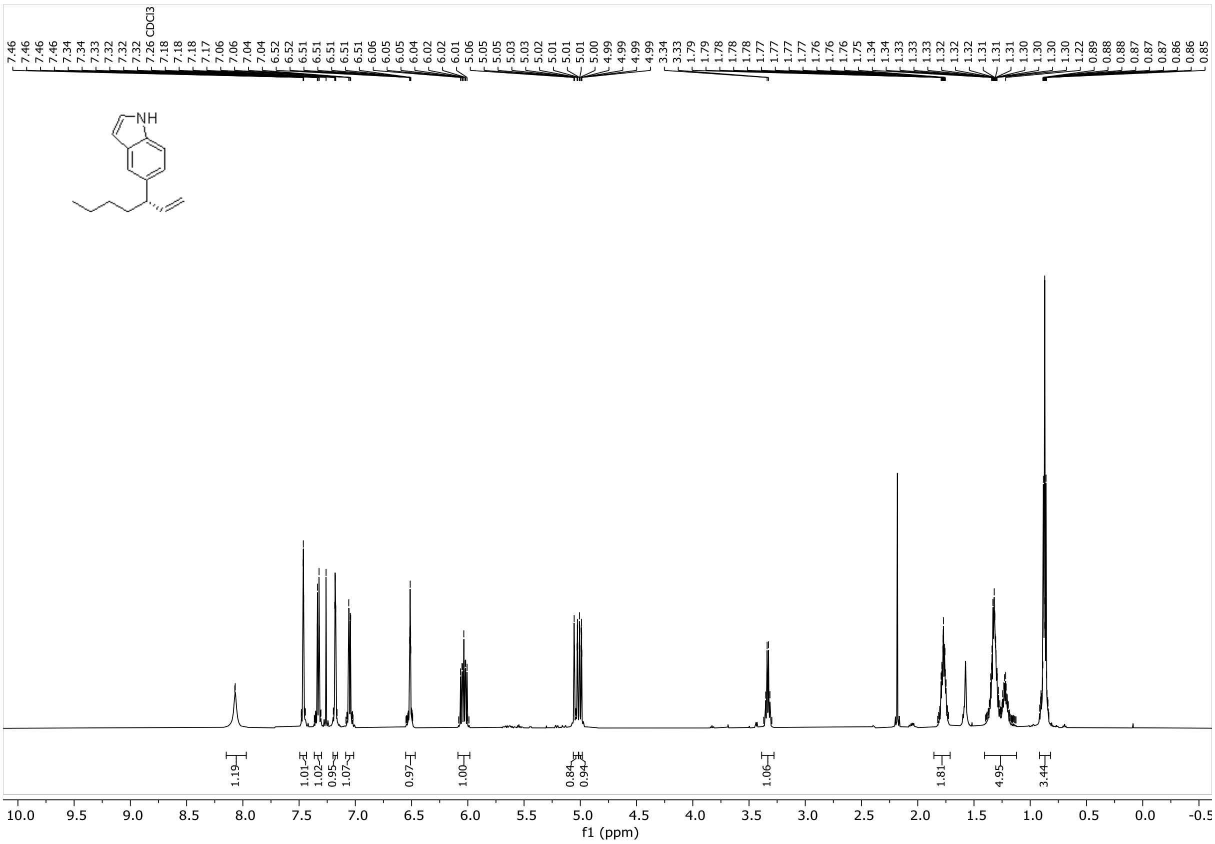

*Figure S56 ¹H-NMR(600 MHz) of **5p** in $CDCl_3$.*

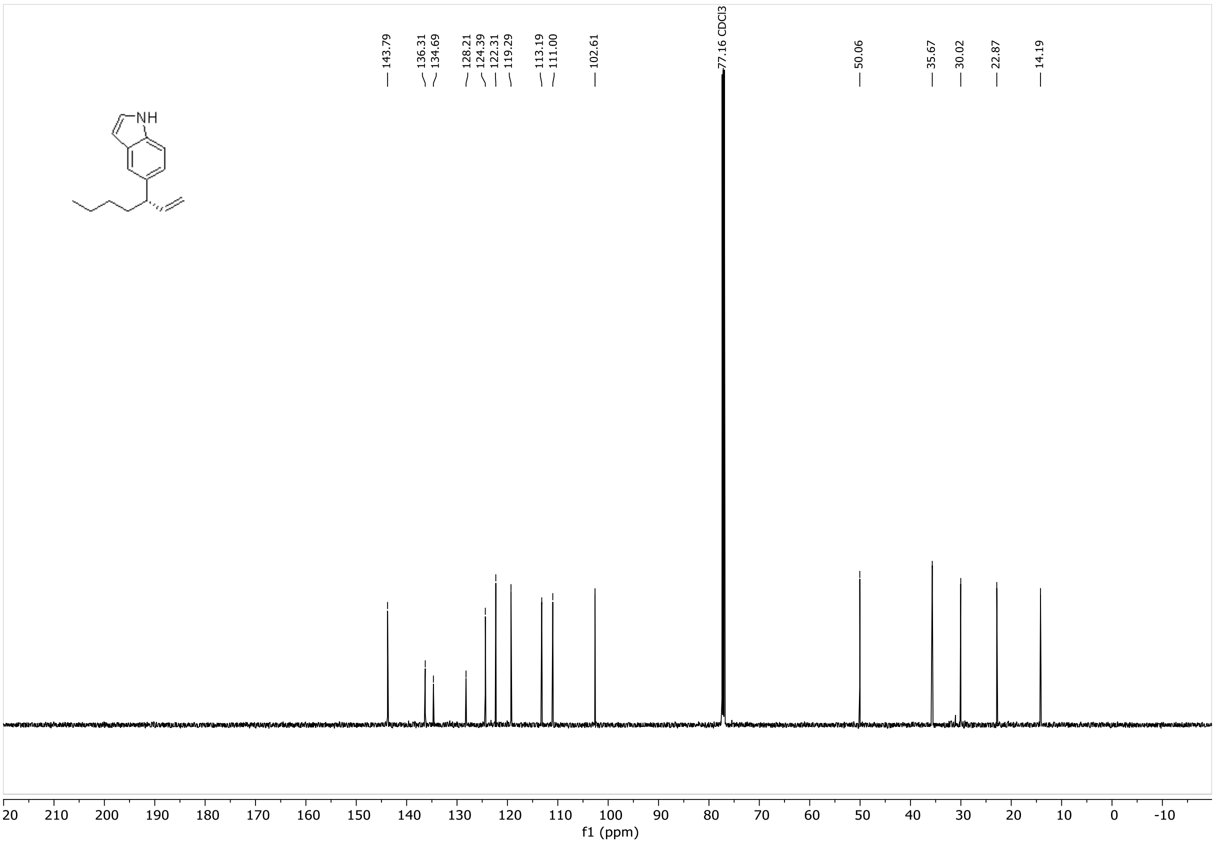

*Figure S57 $^{13}C\{^1H\}$-NMR(151 MHz) of **5p** in $CDCl_3$.*

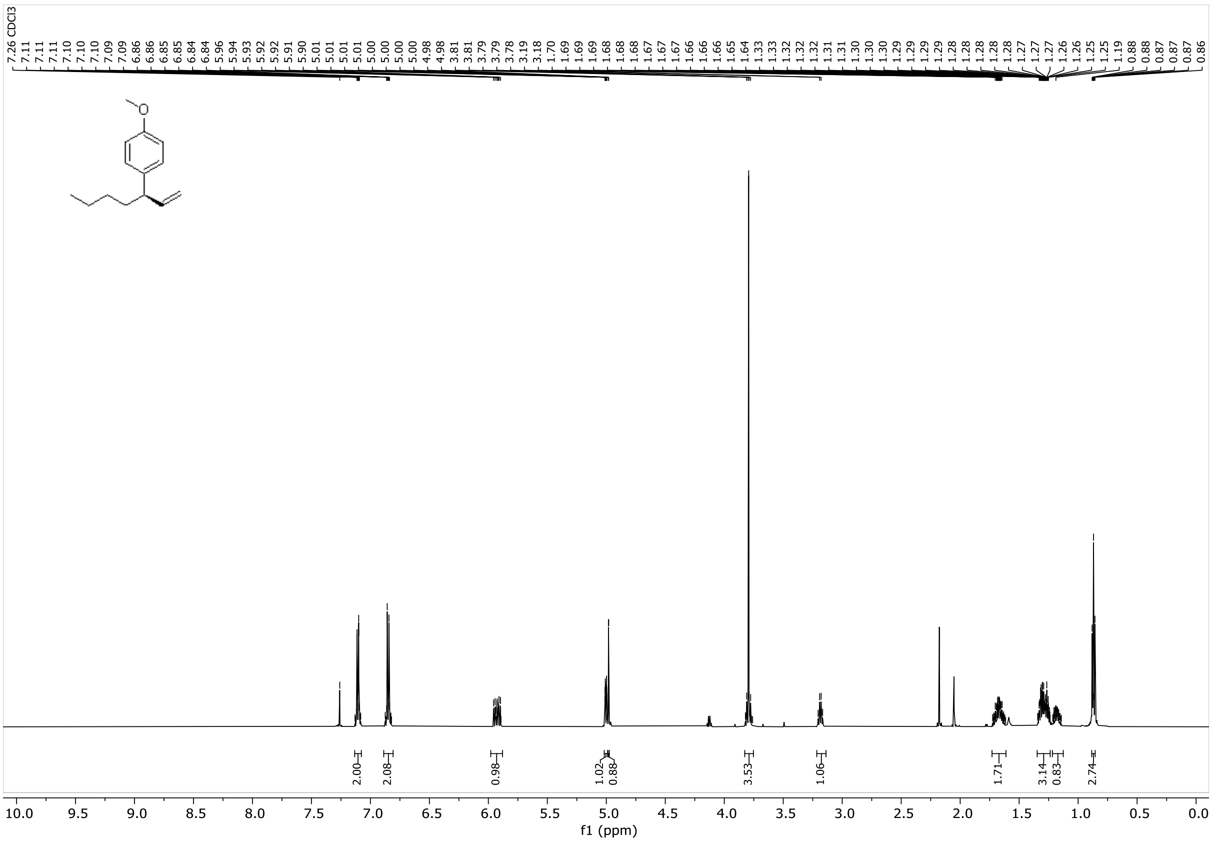

*Figure S58 ^{1}H-NMR(600 MHz) of **5q** in $CDCl_3$.*

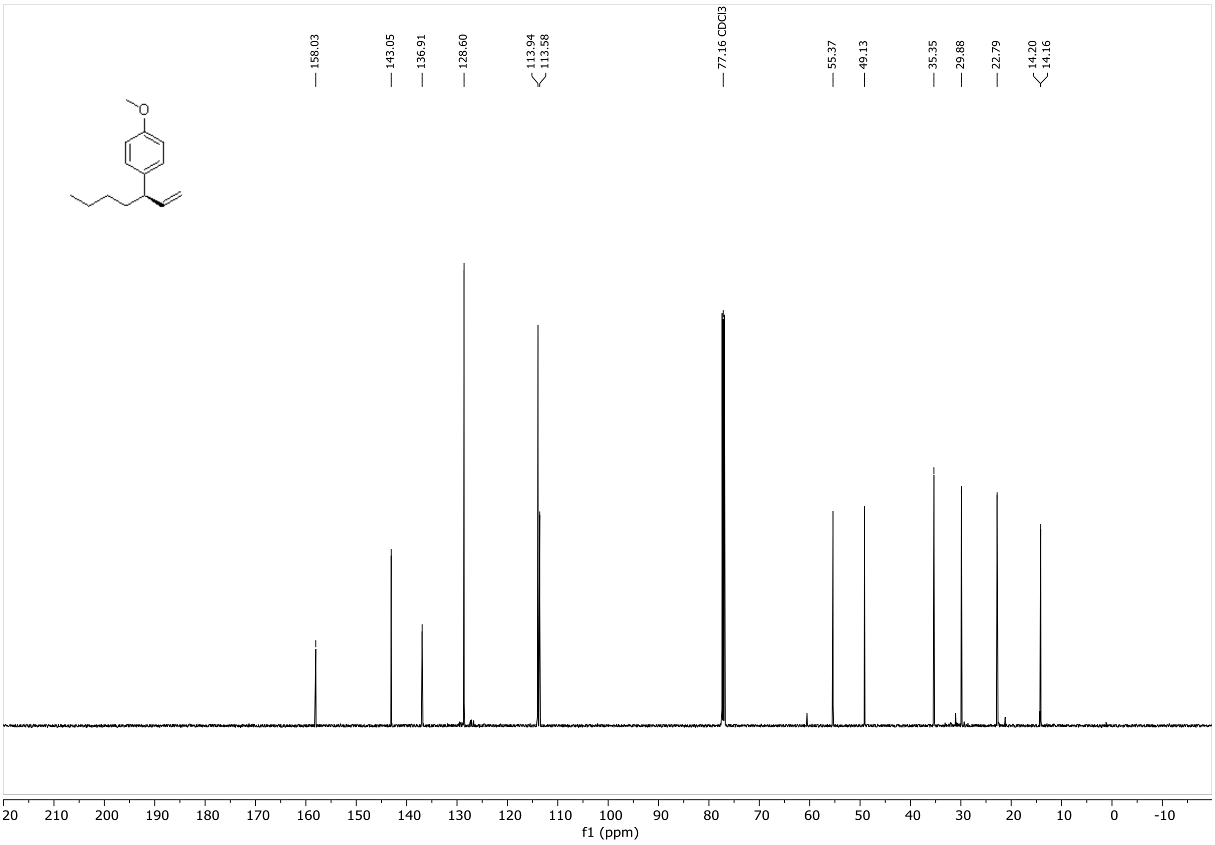

*Figure S59 $^{13}C\{^1H\}$-NMR(151 MHz) of **5q** in $CDCl_3$.*

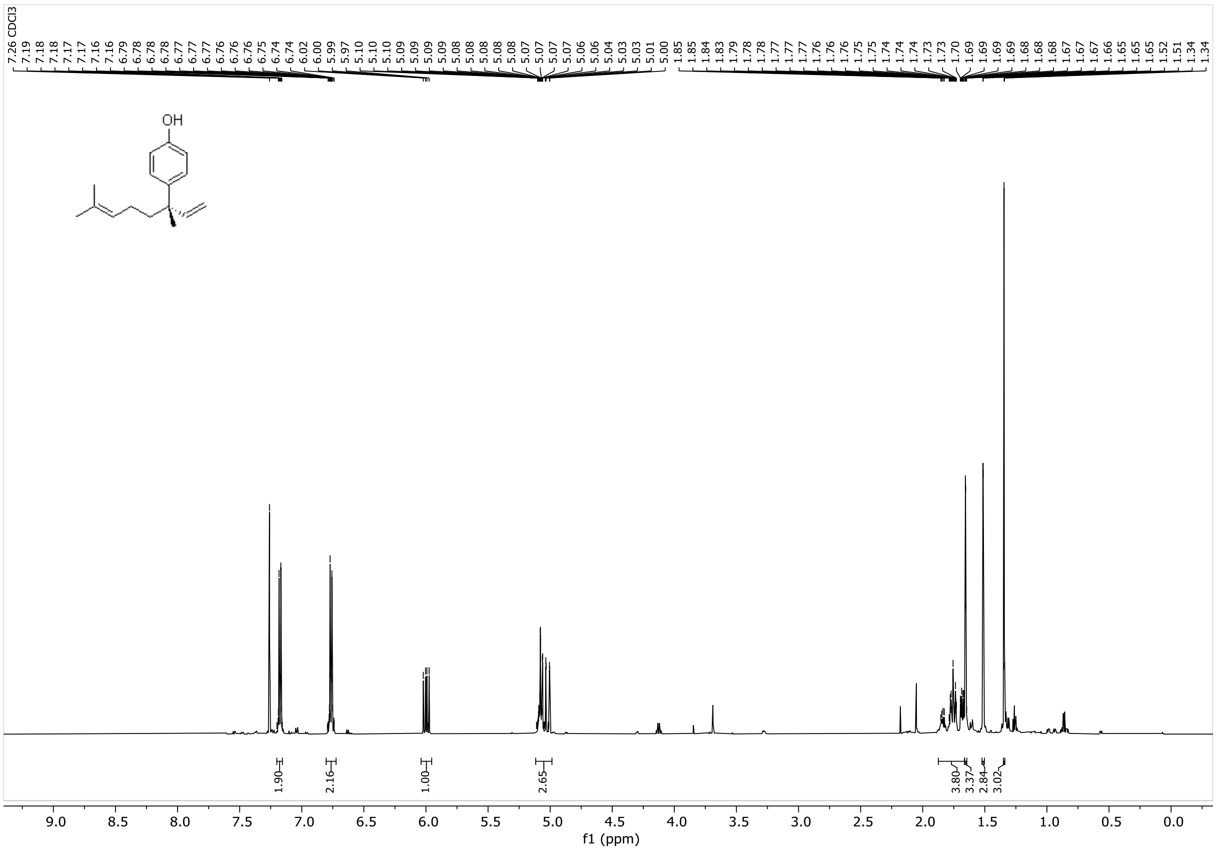

*Figure S60 ^{1}H-NMR(600 MHz) of **2** in $CDCl_3$.*

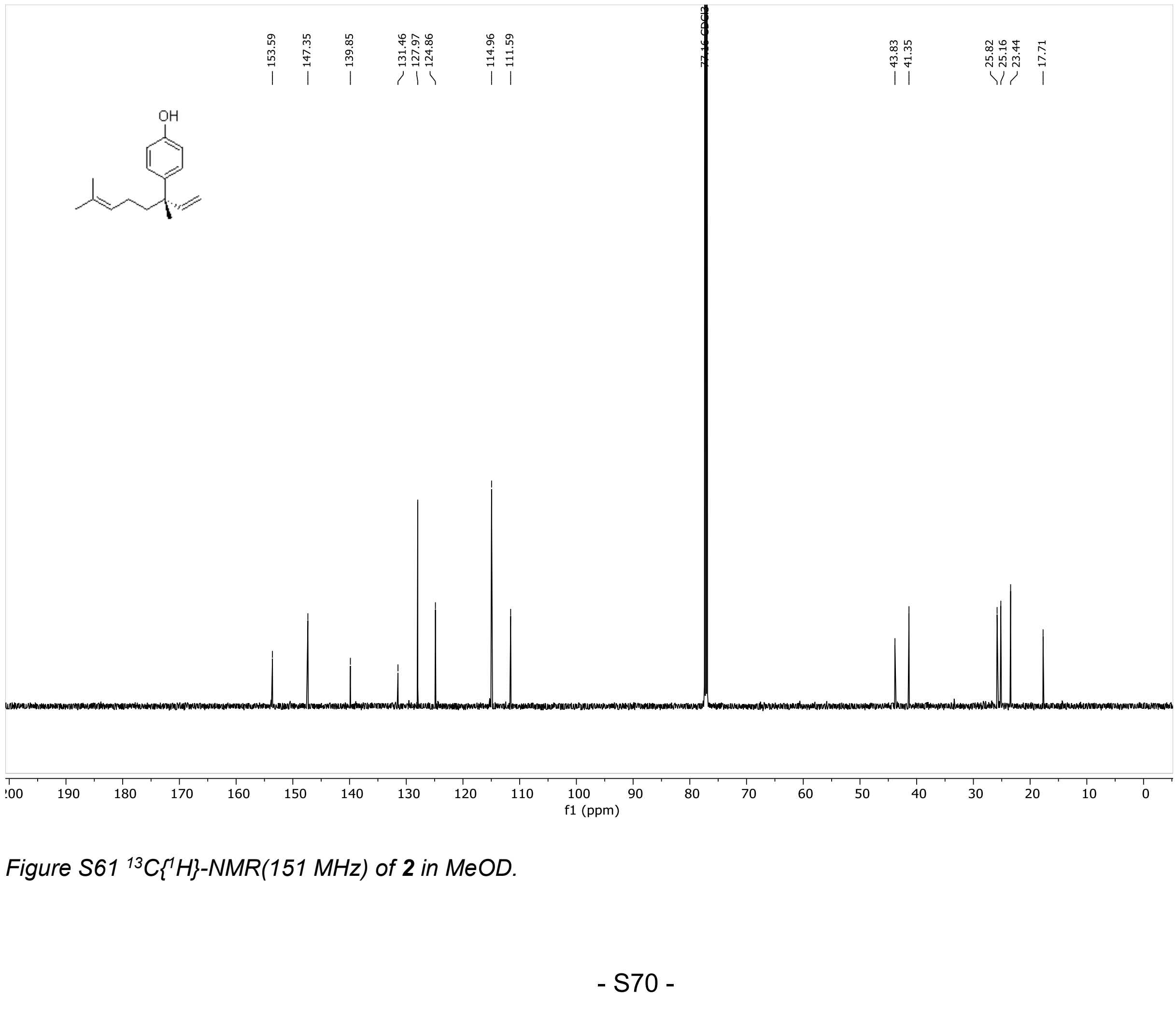

*Figure S61 $^{13}C\{^{1}H\}$-NMR(151 MHz) of **2** in MeOD.*

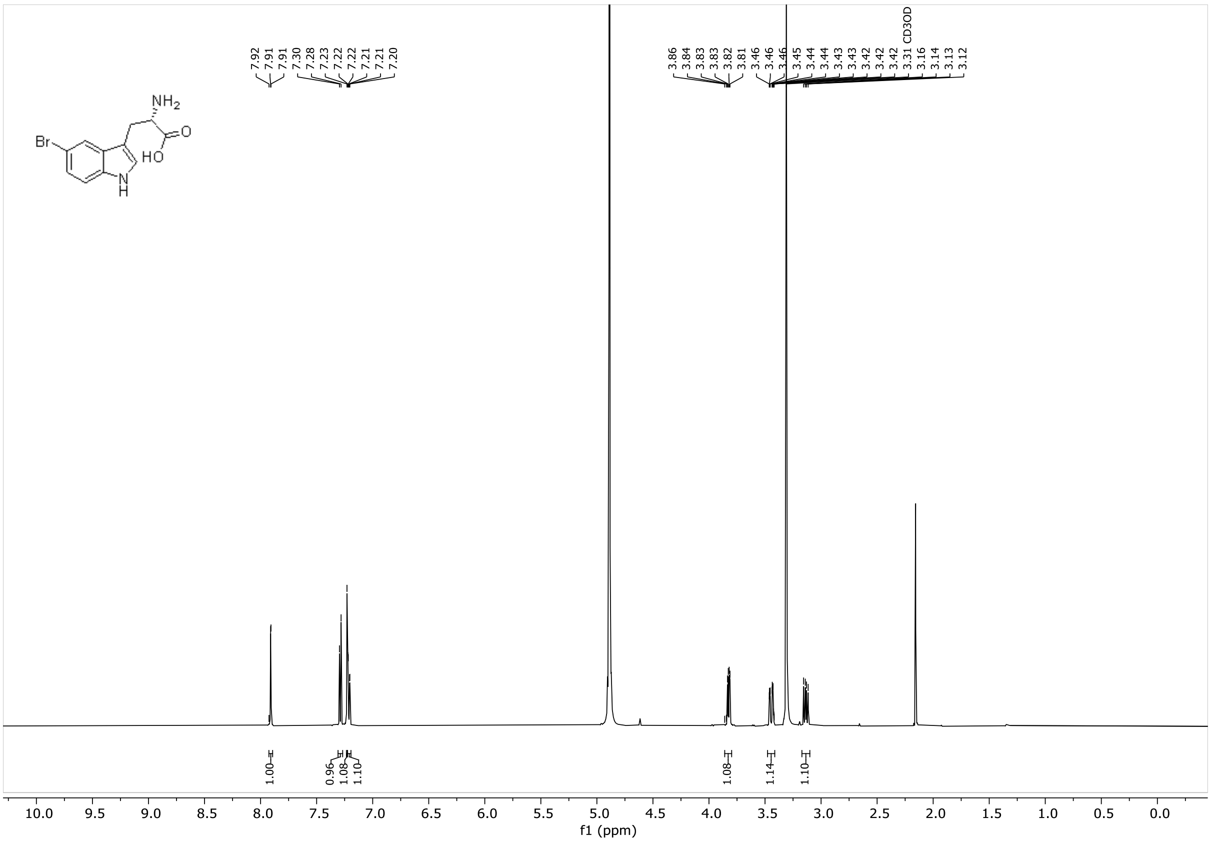

*Figure S62 ^{1}H-NMR(600 MHz) of **11** in MeOD.*

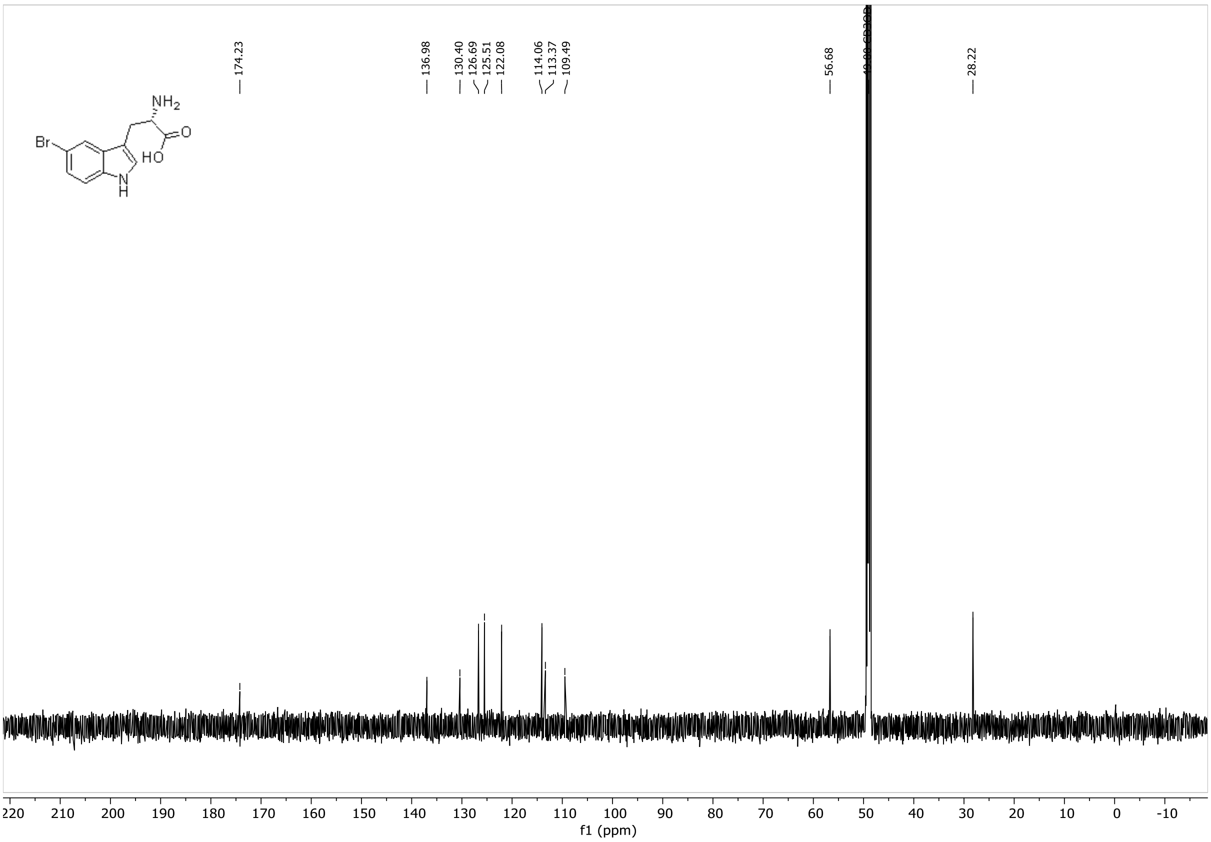

*Figure S63 $^{13}C\{^{1}H\}$-NMR(151 MHz) of **11** in MeOD.*

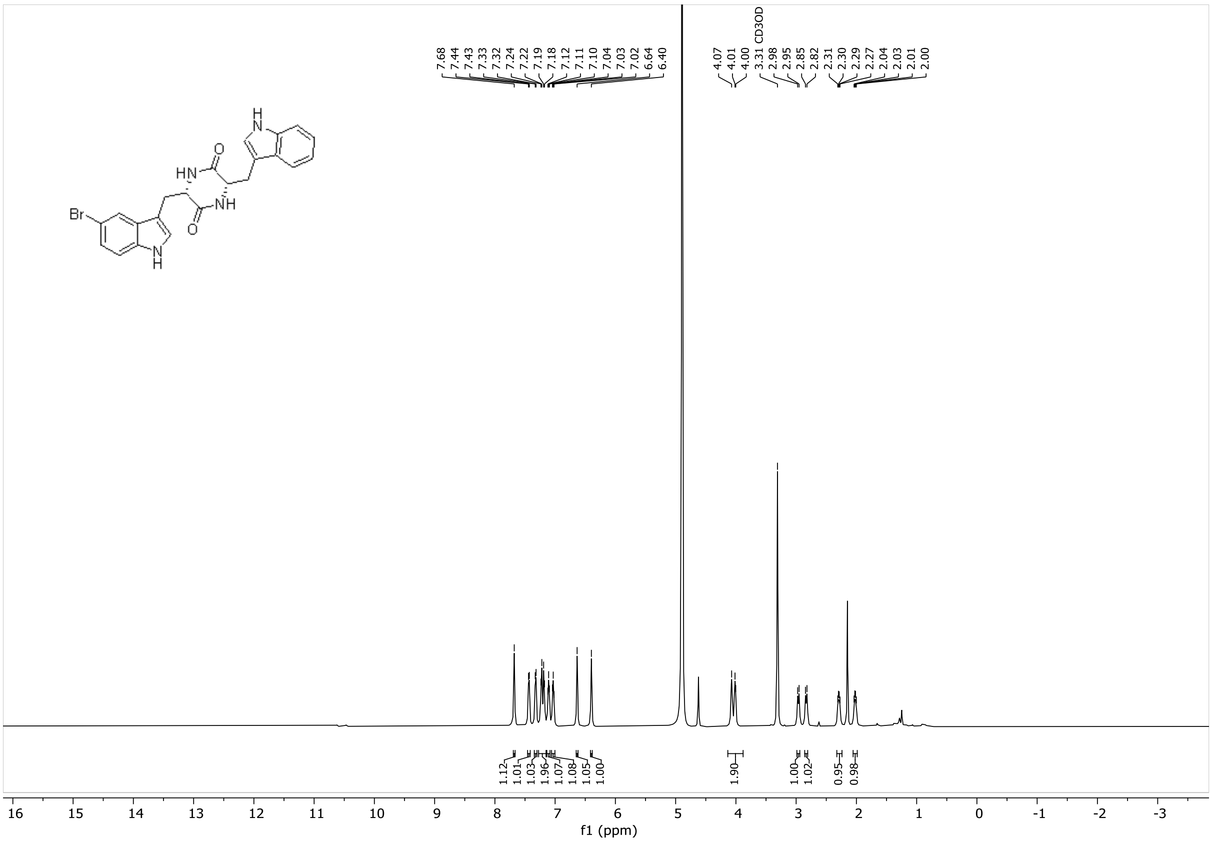

*Figure S64 ^{1}H-NMR(600 MHz) of **7** in MeOD.*

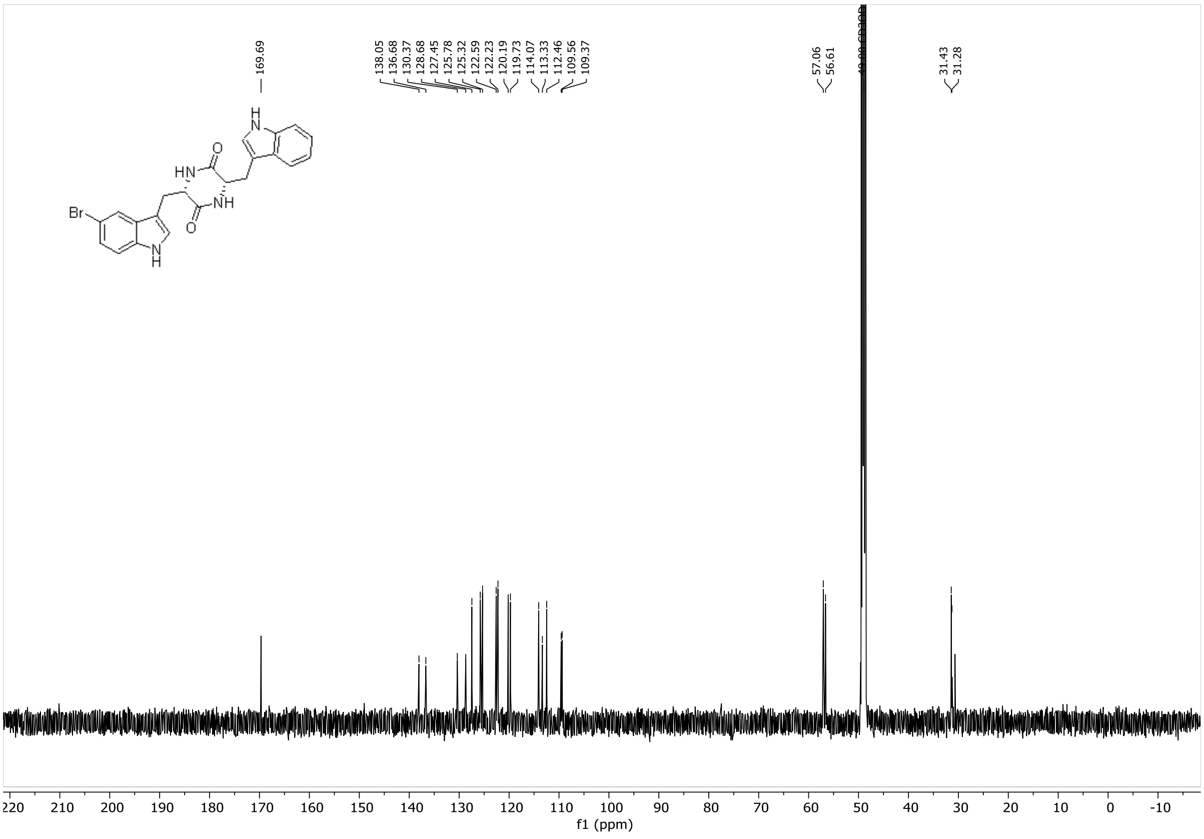

*Figure S65 $^{13}C\{^{1}H\}$-NMR(151 MHz) of **7** in MeOD.*

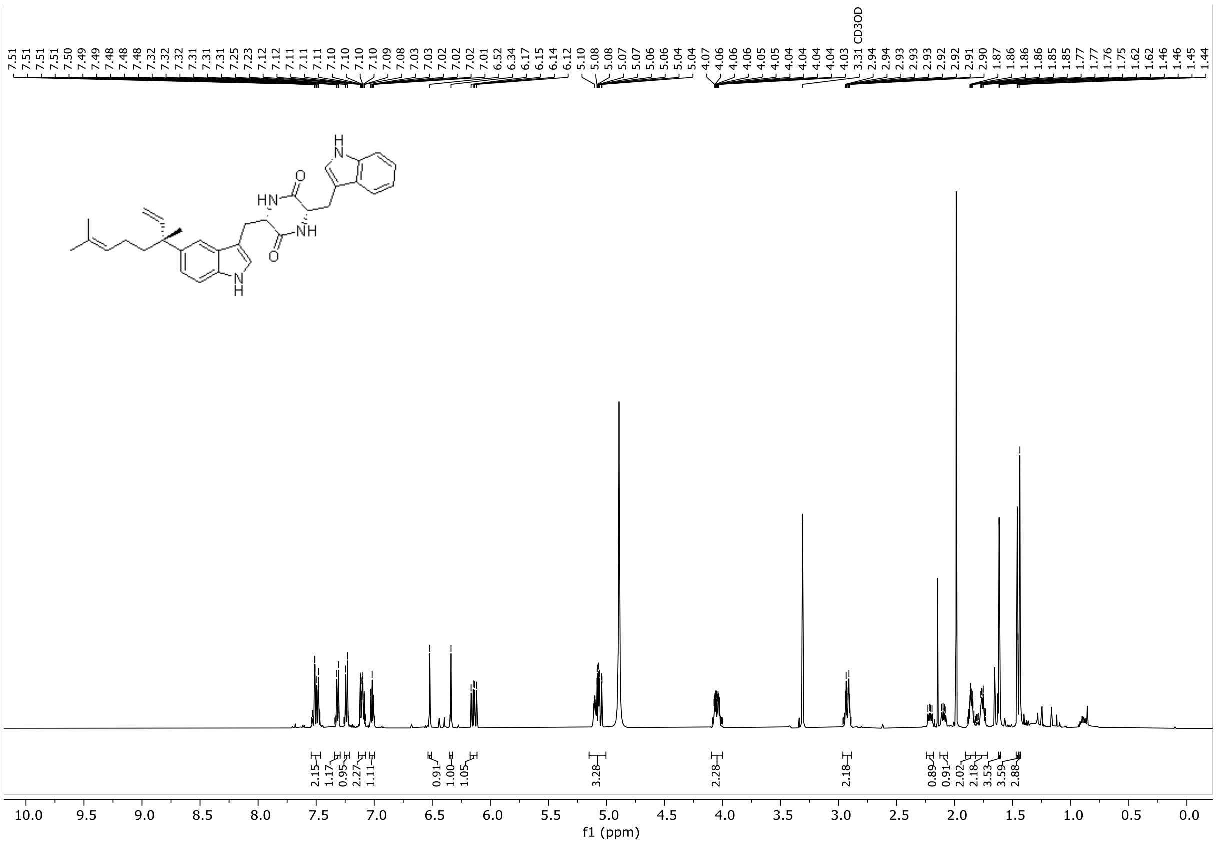

*Figure S66 ^{1}H-NMR(600 MHz) of **8a** in MeOD.*

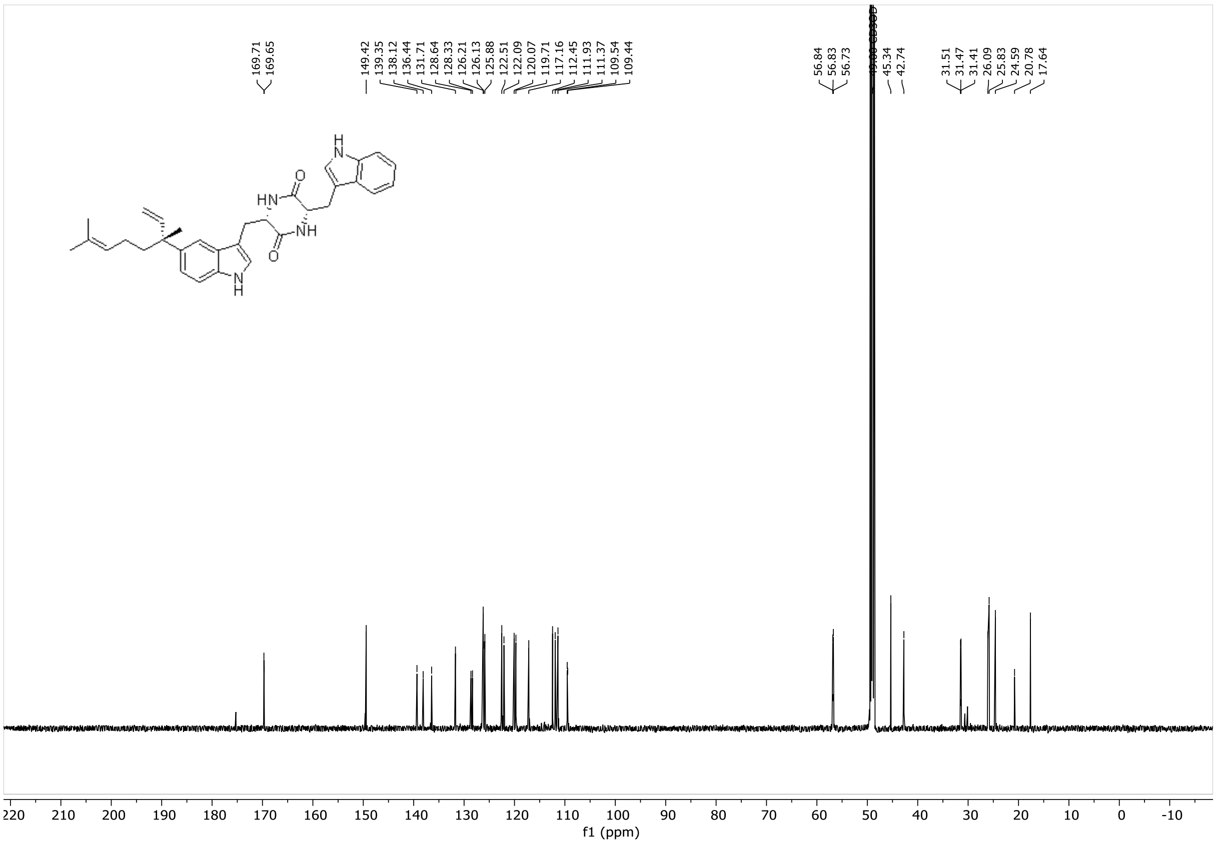

*Figure S67 ^{13}C{^{1}H}-NMR(151 MHz) of **8a** in MeOD.*

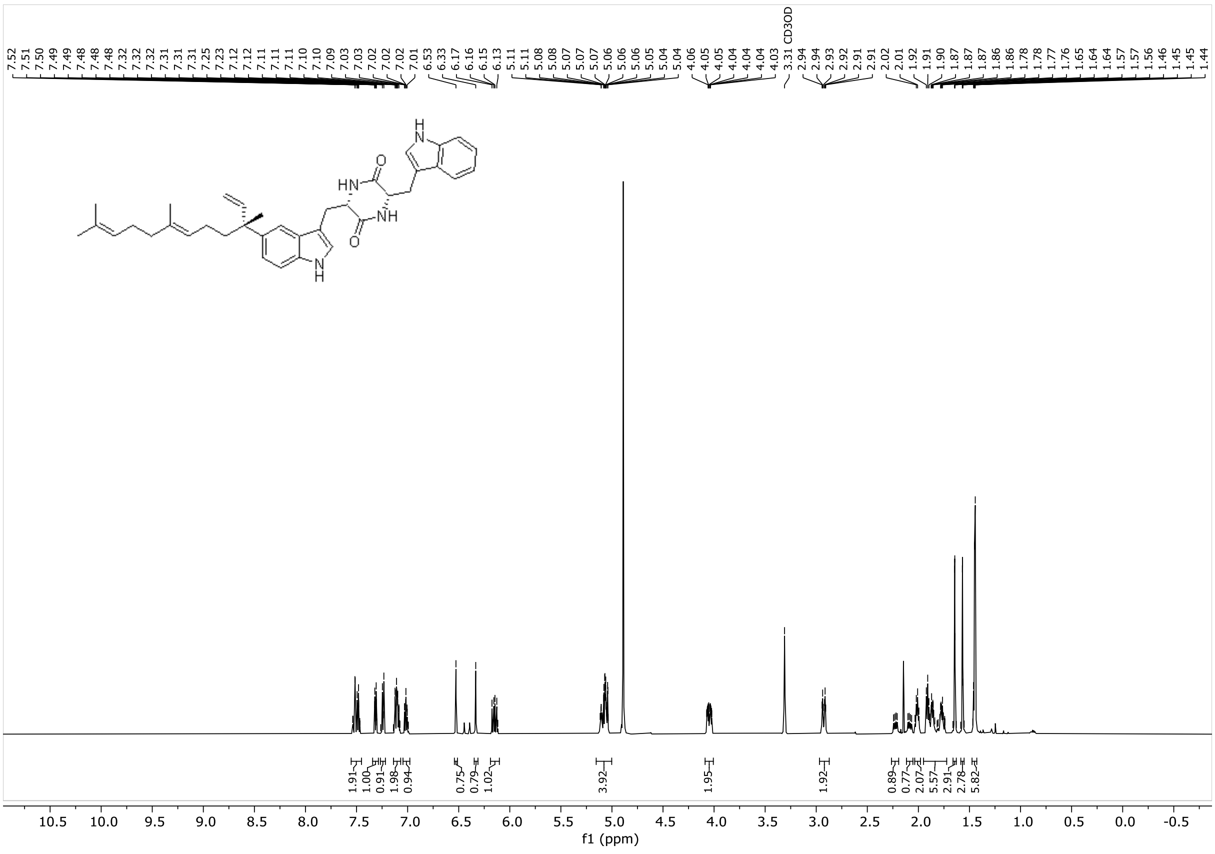

*Figure S68 ^{1}H-NMR(600 MHz) of **8b** in MeOD.*

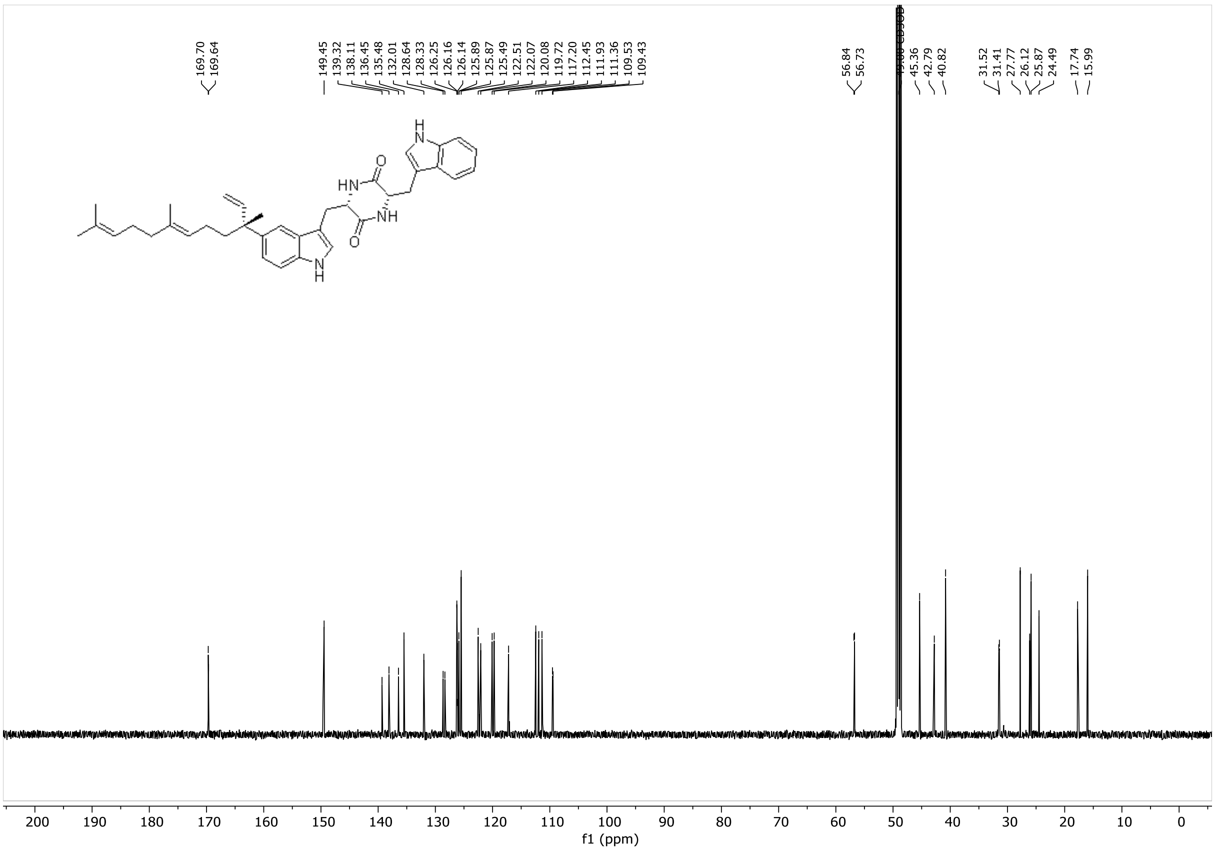

*Figure S69 ^{13}C{^{1}H}-NMR(151 MHz) of **8b** in MeOD.*

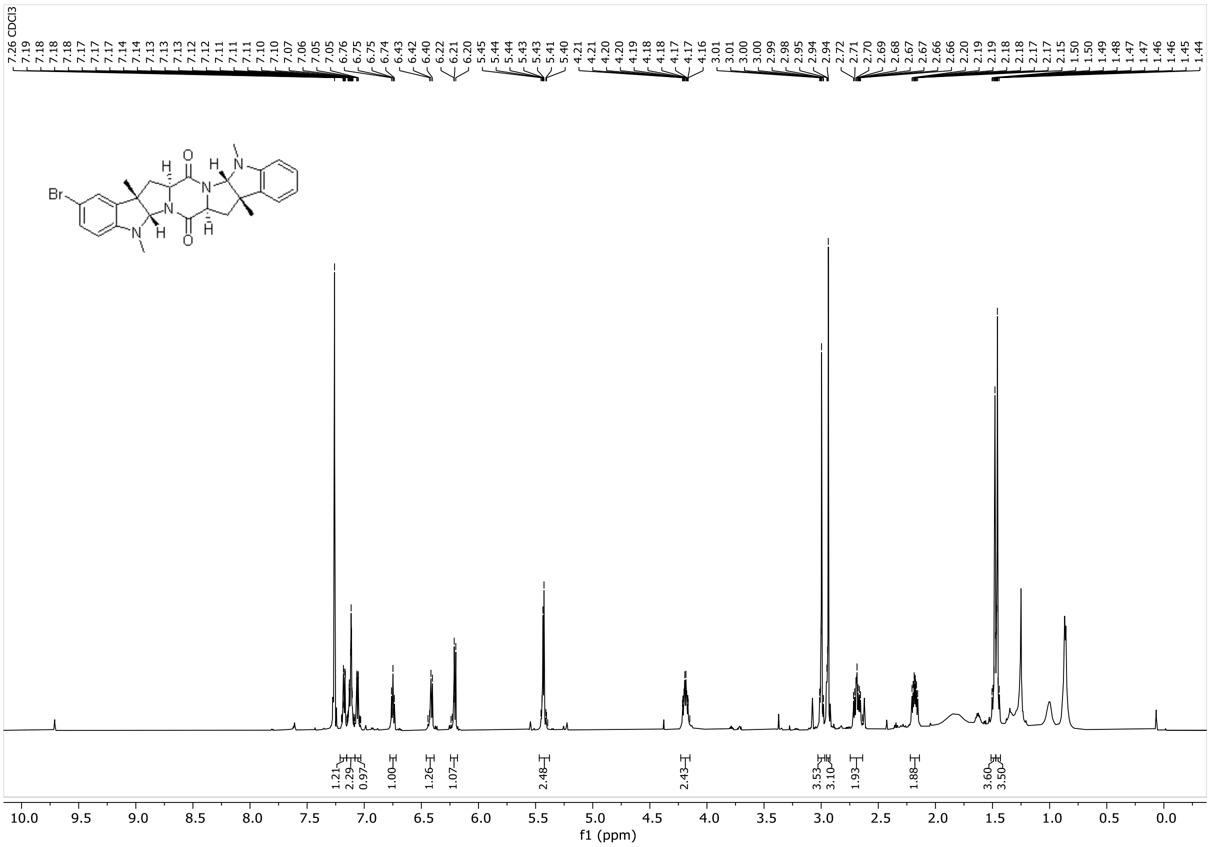

Figure S70 ^{1}H-NMR(600 MHz) of **9** in $CDCl_3$.

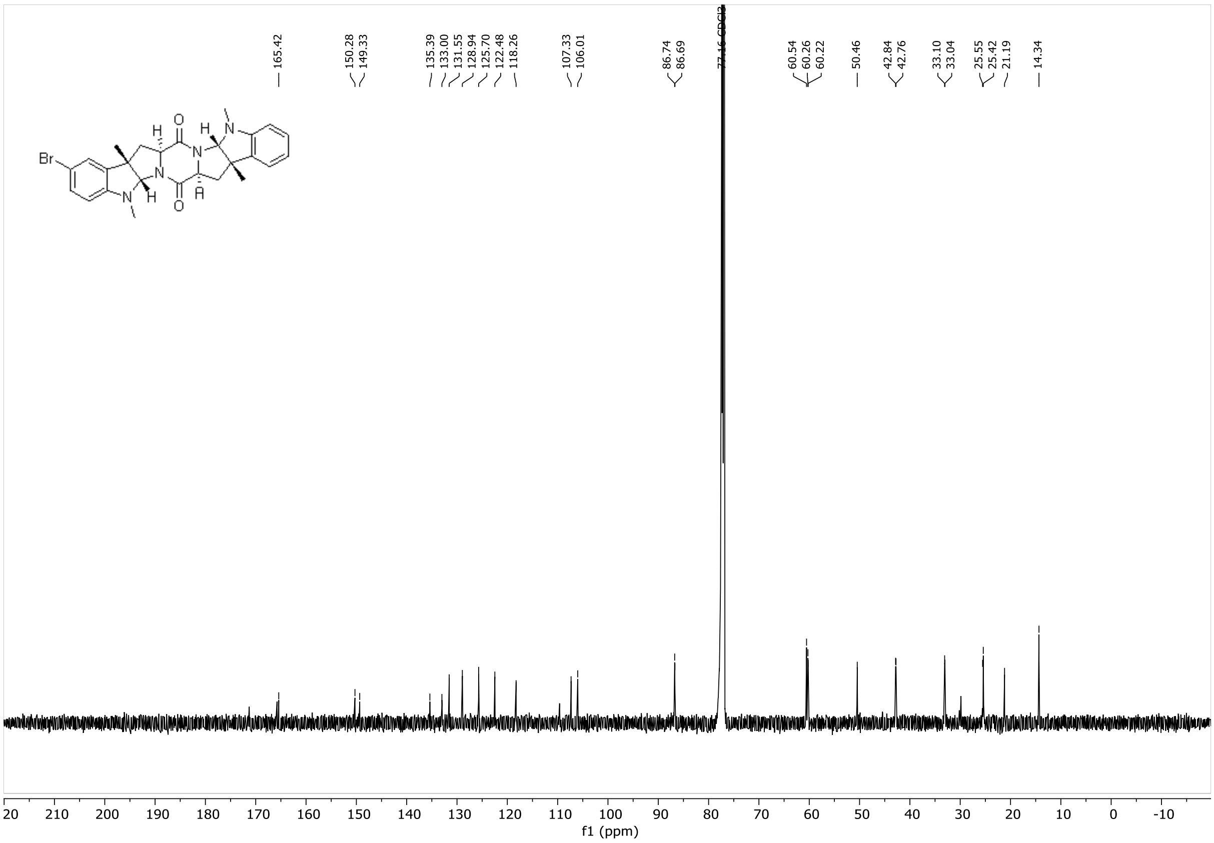

Figure S71 $^{13}C\{^{1}H\}$-NMR(151 MHz) of **9** in $CDCl_3$.

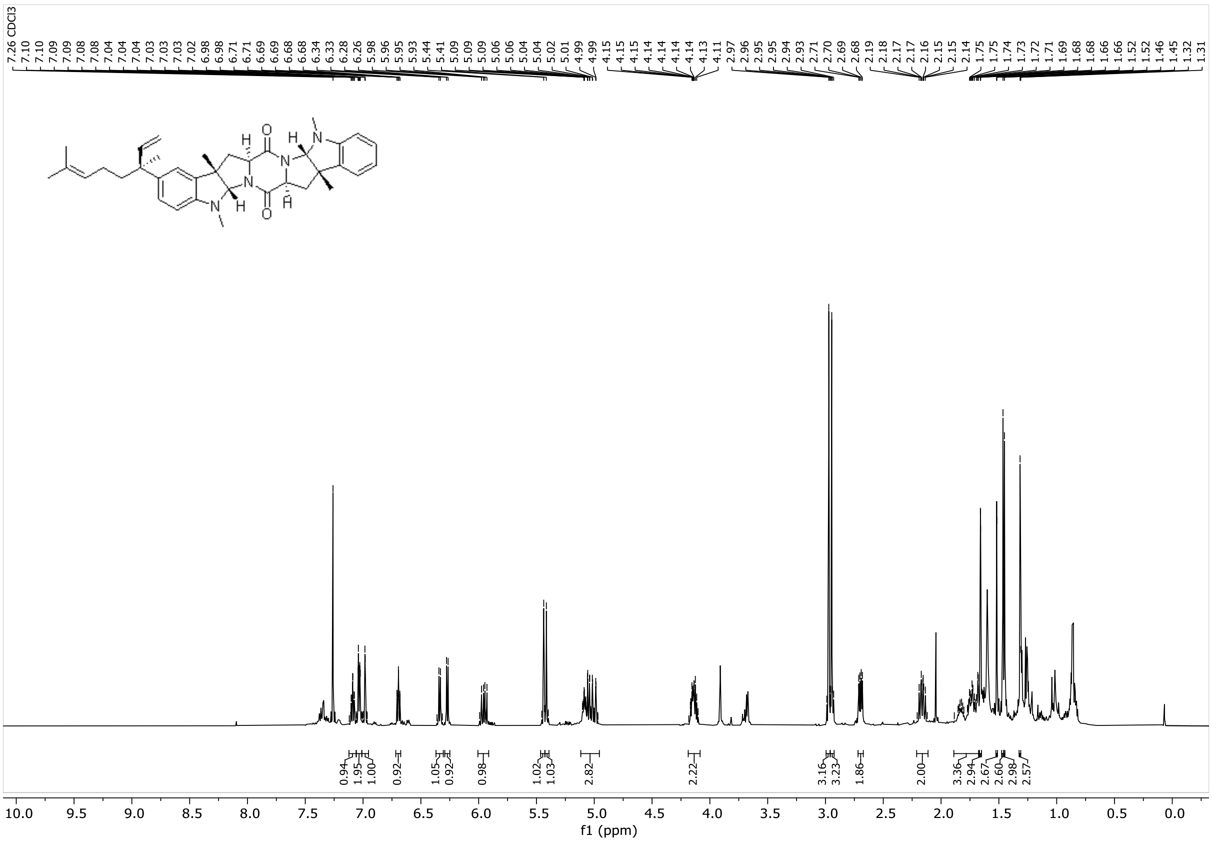

*Figure S72 ^{1}H-NMR(600 MHz) of **10** in MeOD.*

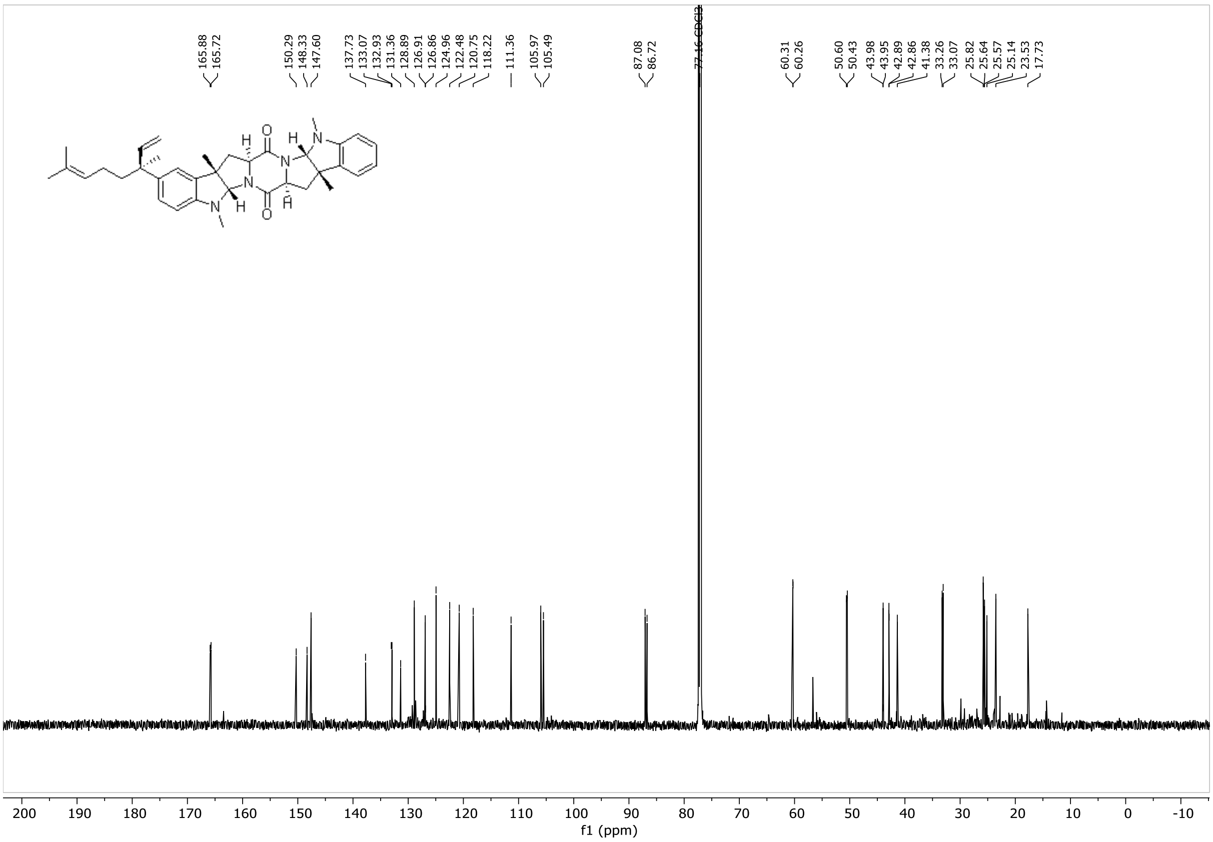

*Figure S73 ^{13}C{^{1}H}-NMR(151 MHz) of **10** in MeOD.*

8. References

[1] G. Dutheuil, N. Selander, K. J. Szabó, V. K. Aggarwal, *Synthesis* **2008**, *2008*, 2293-2297; 'Direct Synthesis of Functionalized Allylic Boronic Esters from Allylic Alcohols and Inexpensive Reagents and Catalysts'; 10.1055/s-2008-1067144.

[2] R. Alam, T. Vollgraff, L. Eriksson, K. J. Szabó, *J. Am. Chem. Soc.* **2015**, *137*, 11262-11265; 'Synthesis of Adjacent Quaternary Stereocenters by Catalytic Asymmetric Allylboration'; 10.1021/jacs.5b07498.

[3] M. Raducan, R. Alam, K. J. Szabó, *Angew. Chem., Int. Ed.* **2012**, *51*, 13050-13053; 'Palladium-Catalyzed Synthesis and Isolation of Functionalized Allylboronic Acids: Selective, Direct Allylboration of Ketones'; 10.1002/anie.201207951.

[4] P. Zhang, I. A. Roundtree, J. P. Morken, *Org. Lett.* **2012**, *14*, 1416-1419; 'Ni- and Pd-Catalyzed Synthesis of Substituted and Functionalized Allylic Boronates'; 10.1021/ol3001552.

[5] F. Gao, K. P. McGrath, Y. Lee, A. H. Hoveyda, *J. Am. Chem. Soc.* **2010**, *132*, 14315-14320; 'Synthesis of Quaternary Carbon Stereogenic Centers through Enantioselective Cu-Catalyzed Allylic Substitutions with Vinylaluminum Reagents'; 10.1021/ja106829k.

[6] A. Trofimova, M. Diamandas, C. Brien, N. Khasanzoda, A. J. Lough, A. K. Yudin, *J. Am. Chem. Soc.* **2024**, *146*, 23365-23375; 'Terpenoid Cyclophanes with Planar Chirality'; 10.1021/jacs.4c06308.

[7] F. Gao, Y. Lee, K. Mandai, A. H. Hoveyda, *Angew. Chem., Int. Ed.* **2010**, *49*, 8370-8374; 'Quaternary Carbon Stereogenic Centers through Copper-Catalyzed Enantioselective Allylic Substitutions with Readily Accessible Aryl-or Heteroaryllithium Reagents and Aluminum Chlorides'; 10.1002/anie.201005124.

[8] D. K. Romney, J. Murciano-Calles, J. E. Wehrmüller, F. H. Arnold, *J. Am. Chem. Soc.* **2017**, *139*, 10769-10776; 'Unlocking Reactivity of TrpB: A General Biocatalytic Platform for Synthesis of Tryptophan Analogues'; 10.1021/jacs.7b05007.

[9] M. Schatton, M. Haase, J. Tenhaef, C. Gronkowsky, S. Noack, J. Pietruszka, *Chem. Eur. J.* **2025**, *31*, e202500740; 'Chemoenzymatic Total Synthesis of Lansai B'; 10.1002/chem.202500740.

[10] M. Haase, O. H. Weiergräber, B. David, E. L. Pfirmann, B. Paschold, H. Gohlke, J. Pietruszka, *Chem. Sci.* **2025**, *16*, 4519-4527; 'Characterization of a C-methyltransferase from Streptomyces griseoviridis – crystal structure, mechanism, and substrate scope'; 10.1039/D4SC07300B.

[11] M. Dick, N. S. Sarai, M. W. Martynowycz, T. Gonen, F. H. Arnold, *J. Am. Chem. Soc.* **2019**, *141*, 19817-19822; 'Tailoring Tryptophan Synthase TrpB for Selective Quaternary Carbon Bond Formation'; 10.1021/jacs.9b09864.
